# High-Yield, Case-Based, Interactive Workshop on Telehealth and Teleneurology With Pediatric Resident Physicians

**DOI:** 10.15766/mep_2374-8265.11340

**Published:** 2023-08-25

**Authors:** Sean K. Wang, Robert C. Goodrich, Lauren D. Strauss, Jaclyn M. Martindale

**Affiliations:** 1 Third-Year Medical Student, Wake Forest University School of Medicine; 2 Chief Resident, Department of Child Neurology, Atrium Health Wake Forest Baptist; 3 Associate Professor, Wake Forest University School of Medicine and Department of Neurology, Atrium Health Wake Forest Baptist; 4 Assistant Professor, Wake Forest University School of Medicine and Department of Neurology, Atrium Health Wake Forest Baptist

**Keywords:** Child Neurology, Telemedicine, Case-Based Learning, Clinical Reasoning/Diagnostic Reasoning, Clinical/Procedural Skills Training, Communication Skills, Online/Distance Learning, Neurology, Pediatrics, Telehealth

## Abstract

**Introduction:**

Increasing prevalence of neurologic disorders with an aging global population and limited availability of neurologists may lead to worse patient outcomes. As a result of the COVID-19 pandemic, telehealth services surged, and despite easing public health measures, the demand has remained. Telehealth technology has the potential to close the physical gaps in expanding the reach of care. This academic half-day workshop sought to provide a learning opportunity in response to these concerns.

**Methods:**

The workshop consisted of small- and large-group case discussions among pediatric resident physicians (PGY 1-PGY 3) moderated by two child neurology faculty physicians over Zoom. Participants received a learner document with prereading articles and questions for each case. PowerPoint presentations with video demonstrations were used to introduce the cases and guide discussions.

**Results:**

Of the 25 attendees, 14 (56% response rate) answered a nonmandatory postsession survey. Eighty-six percent of the respondents were very or extremely satisfied with the content covered and were similarly satisfied with the effectiveness of content delivery. Seventy-nine percent of the respondents found the content helpful or very helpful in preparation for the board, and 93% anticipated applying the content covered occasionally or frequently in their clinical practice.

**Discussion:**

Small-group discussions with video demonstrations are helpful in increasing proficiency with telehealth technology and in examining board-relevant cases on pediatric patients. There is strong interest in subsequent telehealth half-day workshops that incorporate teaching through group discussions on relevant patient case scenarios.

## Educational Objectives

By the end of this activity, learners will be able to:

1. Identify the advantages and disadvantages of telehealth when working with the pediatric population.
2. Adapt to common obstacles faced during a telehealth visit.
3. Conduct an age-appropriate neurological examination using telehealth technology.

## Introduction

Neurological disorders are a common cause of disability, particularly in older adults. As the global population increasingly ages and the average life span lengthens, the prevalence of neurological disorders is likely to increase.^1,2^ Regular access to neurologic care has been shown to reduce acute care hospitalizations and to increase the application of disease-specific treatments.^3^ However, with a progressive decline in national interest in neurology, there is a strong need for non-neurologist physicians to be trained and for neurologists to be able to expand their reach of care by utilizing telehealth to reduce the current mismatch in supply and demand for neurologic sevices.^4,5^

Telehealth, or telemedicine, can be defined as the delivery of medical care without in-person contact. During the COVID-19 pandemic, there was a surge in demand for such remote services to minimize community spread and exposure to patients while maintaining patient care.^6^ Discouragingly, many providers initially felt they lacked sufficient virtual clinical experiences and were concerned about their ability to conduct an effective evaluation and deliver compassionate care virtually.^7^ Even as public health precautions have relaxed, telehealth use has remained. A national study showed an increase in telehealth use from 0.3% of patient-physician contacts in 2019 to 23.6% of contacts in 2020 in a cohort of 36 million Americans.^8^ Furthermore, FAIR Health, an independent nonprofit that compiles a national database of both private and Medicare claims, showed an increase in the overall percentage of telehealth claims from 0.2% in December 2019 to 4.9% in December 2021 to 5.4% in May 2022.^9^ With the increasing prevalence of telehealth, it is essential for health care professionals to be trained in utilizing telehealth accurately and effectively.

In addition to promoting a learning experience with telehealth, another of our goals was to address *neurophobia,* a term coined by Dr. Ralph F. Jozefowicz in 1994 describing the avoidance or even overutilization of the neurological sciences whether from intimidation, boredom, or difficulty understanding the concepts.^5,10,11^ More informed trainees make for more informed practitioners who are comfortable with managing neurological conditions. Because of the limited availability of child neurology physicians, there is a need for pediatricians to have a strong understanding in regard to recognizing and managing patients who present with neurologic findings.

A review of *MedEdPORTAL* publications revealed numerous case-based approaches to developing neurology comprehension or telemedicine use largely targeted towards medical students.^12–17^ Fewer publications targeted either neurology comprehension or telehealth technology use with graduate medical learners.^12^ Moreover, while there were many resources available on utilizing telehealth, there were none on teleneurology. Based on the success seen with clinically relevant case-based learning, we utilized a similar approach to encourage inquiry and discussions.^14–16^

The pediatric residency program at Atrium Health Wake Forest Baptist has weekly academic half-days to promote the educational activities of its residents. The curriculum has an 18-month rotational schedule that includes three child neurology sessions. To address the need in resident education and contribute to the literature, the Department of Child Neurology designed a 3-hour academic half-day workshop that occurred over Zoom in April 2020 in which pediatric resident physicians engaged in group case discussions to increase familiarity and instill confidence with telehealth technology, adapt age-appropriate clinical skills to a virtual setting, and develop the communication skills needed to deliver compassionate care virtually.

## Methods

### Preassignments

Prior to the workshop, prereading of four papers was assigned on the following topics: advantages and limitations of teleneurology, hypotonia in floppy infants, evaluation of children with developmental disabilities, and pediatric neurological examination via telehealth technology.^18–21^ Sufficient preparation on clinical topics laid groundwork for active participation in group discussions (Appendix A).

### Session Preparation

The intended audience for this workshop was pediatric resident physicians (PGY 1-PGY 3) who might not feel comfortable conducting a telehealth visit efficiently and effectively for a neurologic complaint. Medical students rotating on pediatrics were also invited to the academic half-day sessions. The workshop was conducted in the early COVID-19 pandemic when familiarity with telehealth was limited; however, these skills have continued to remain applicable and relevant to clinical examination skills, knowledge, and board preparation. A virtual-based platform with small-group (breakout room) capability was required to successfully offer this workshop virtually, but it could also be reproduced in the in-person setting. The session was designed in a case-based interactive format, with each case focusing on a key component of the pediatric neurological exam while incorporating a high-yield, boards-related, clinically relevant topic. Prior to the workshop, the facilitators performed focused teleneurology exams on other faculty members, residents, and their children. These were recorded and incorporated into the PowerPoint sessions.

The overall session was subdivided into two sessions moderated by two child neurology faculty physicians: an overview of telehealth with group discussions on general telehealth cases followed by group discussions of high-yield teleneurology cases. The resident physicians were split into four breakout groups, with each group assigned a general telehealth exam case and two teleneurological exam cases. The exact number of residents in a group would vary depending on the size of the program; however, we had five to six residents per group, which allowed for sufficient organic discussion amongst all residents. The small groups were facilitated by both a child neurology resident and an attending.

The facilitators followed a document guide (Appendix A) for facilitating the workshop overall, including the small- and whole-group discussions, and presented didactic Powerpoint presentations that included videos demonstrating proper physical exam maneuvers and findings (not included in this publication), such as testing for infantile reflexes through a virtual platform. Due to privacy concerns, the original videos have not been included here, but we do provide references to optional sample videos in the speaker notes for relevant slides in Appendix C. Learners received their own guide to the session (Appendix B).

A pediatric residency program coordinator was available throughout the workshop to facilitate breakout rooms and offer technical support.

### Workshop

#### Overview of telehealth

The first session started off with a 30-minute overview of a general telehealth experience featuring an interactive discussion on advantages, disadvantages, and adaptations to unexpected challenges (Appendix D). Following this overview, attendees broke off into small-group sessions and reviewed general telehealth exam cases for 10 minutes. Each of the four general exam cases covered different areas of a physical exam, such as an overall, HEENT (head, eyes, ears, nose, and throat), cardiopulmonary, or abdominal exam. The larger group reconvened for a 20-minute review discussion on teaching points from each of the general telehealth cases.

#### Teleneurology cases

After a 10-minute break, there was a brief teleneurology introduction (Appendix C). Resident physicians returned to their previously assigned groups for a 15-minute breakout discussion on their first teleneurology case. Each case was designed to focus on a specific aspect of the neurological examination and to discuss adjustments to the clinical exam according to age and modality, treatment considerations for the diagnosis including suitability of telehealth, and board relevance. The larger group reconvened for a 45-minute group discussion to review the cases. This process was repeated for the second teleneurology case.

Following all pediatric academic half-days, the resident physicians completed an evaluation form online about the session as well as individual facilitators. Residents evaluated satisfaction and effectiveness of content delivery for the presentation, conference material, and relevance to board preparation and clinical practice on Likert scales. Additionally, resident physicians provided open feedback on what they liked best and what could be improved. Each faculty member was rated on their effectiveness of facilitation (Appendix E).

## Results

Fourteen of 25 attendees (56% response rate) filled out the optional anonymous postsession survey online. This was a standard feedback form the pediatric residency utilized for all its academic half-day sessions.

The workshop was one of the most highly rated academic half-days for pediatric residents. Twelve respondents (86%) were very or extremely satisfied with the workshop content covered and were similarly very or extremely satisfied with the effectiveness of content delivery. Ten respondents (79%) found the content helpful or very helpful in their preparation for the board, and 13 respondents (93%) expected to apply the content covered occasionally or frequently in their clinical practice (Figure).

**Figure.**
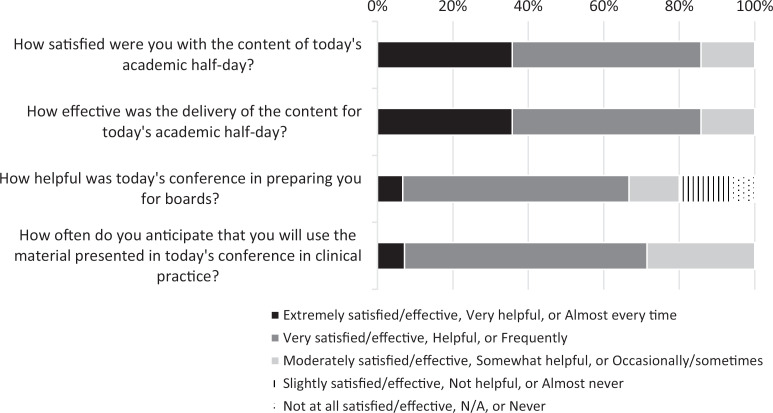
Postsession evaluation responses.

The pediatric resident physicians shared comments expressing their enjoyment with the telehealth workshop, interacting as a small group, having video demonstrations as resources, and having moderators who were neurologists. Comments on what they liked included the following:

• “Good review of neurology exam on telemedicine. I liked the video exams of real kids.”
• “Loved the cases and the fact that there was a neurology authority to help guide us through the cases rather than talking about it amongst ourselves.”
• “I enjoyed the format of today's conference. The case-based format was interactive and engaging.”

Comments on areas for improvement included the following:

• “I would remove the first hour of this talk on how to do a telehealth visit. This was too general. We ran out of time to talk about the more clinically relevant neurology content.”
• “More board related questions.”
• “Since we each had different cases, when we came back together, we didn't spend as much time on each so I feel like you learned a lot about your case and not as much about others so it would have been better as one talk going through all the cases rather than breakout rooms.”

## Discussion

Overall, this academic half-day workshop received strong ratings based on the postsession survey sent out. The survey results showed that the resident physicians were extremely satisfied with the workshop material and format and that they found the content helpful in board preparation. Although adherence was not measured, the results strongly showed residents could foresee applying the knowledge gained from this workshop in their clinical practice. These positive ratings suggest our accomplishment of reaching the first two levels of Kirkpatrick's pyramid.^22^ We used the standard feedback form our pediatric residency utilizes for all its academic half-day sessions, which did limit the type of feedback assessed. Longitudinal feedback for behavioral change might prove more feasibly challenging in our specific setting but would show more application of the knowledge obtained from the workshop.

From the written feedback, the small-group, case-based discussions in which each attendee could actively participate were engaging and well received. Another strongly positive feature was the incorporation of video demonstration recordings after case discussions moderated by a knowledgeable facilitator. This success has continued to be modeled in our other neurology academic half-days for pediatric resident physicians. We have incorporated other feedback such as shortening introductory slides, offering more board-pertinent cases, and extending large-group discussions to learn about the other cases. Future directions include more time to discuss cases in the smaller groups combined with an increase in participation in the large-group discussions to facilitate the educational experience. To balance each of these aspects, six total cases have been an effective number to facilitate in-depth discussions and large-group learning. This has successfully been replicated in three other academic half-days with 15 minutes of small-group discussion per case. Interactive worksheets have also been incorporated, allowing residents to benefit equally from all case discussions.

Feasibility may provide some challenges depending on the institution or setting. While the workshop was conducted in a single 3-hour didactic session, it could be divided into two shorter sessions. Additionally, our workshop was implemented during the early stages of the COVID-19 pandemic. Providers with experience in telemedicine can remove the introduction to telehealth and prioritize teleneurology cases and group discussions. Furthermore, while all institutions may not have pediatric neurology attendings or residents readily available, an educator with sufficient knowledge of telehealth and of adapting the neurologic exam to the virtual setting could easily facilitate the same workshop with any primary care specialty. To demonstrate the teleneurology exam, we filmed some of our own videos, which may not be feasible at other institutions. For such a situation, we have included references in the speaker notes in Appendix C to optional sample videos that can be found online.

Lastly, although this workshop focuses on pediatric neurology, its structure can be translated to other disciplines by incorporating small-group discussions with a whole-group summary, facilitators with backgrounds in the material covered, and video demonstrations. While the workshop was completed virtually due to the pandemic, it can also be reproduced in an in-person educational setting.

Even though telehealth came to prominence thanks to public health concerns during the COVID-19 pandemic, its role in neurology and in medicine has become better established in health care delivery and in addressing social determinants of health, such as reducing the travel burden and time away from employment or school for either patients or caregivers. With the increasing prevalence and advantages of telehealth technology, it is relevant to continue teaching the skills useful for delivering compassionate care virtually that can also be applied in an in-person setting.

## Appendices


Facilitator Guide.docxLearner Guide.docxTeleneurology Cases.pptxTelehealth Introduction.pptxConference Evaluation.docx
*All appendices are peer reviewed as integral parts of the Original Publication.*
