# Supplementary material for: High-Yield, Case-Based, Interactive Workshop on Telehealth and Teleneurology With Pediatric Resident Physicians
Source: MedEdPORTAL. 2023 Aug 25;19:11340. doi: 10.15766/mep_2374-8265.11340 (PMC10450098; doi:10.15766/mep_2374-8265.11340)
Supplement: Supplementary file 1 — Facilitator Guide.docxLearner Guide.docxTeleneurology Cases.pptxTelehealth Introduction.pptxConference Evaluation.docx [file mep_2374-8265.11340-s001.zip › D. Telehealth Introduction.pptx]

## Slide 1
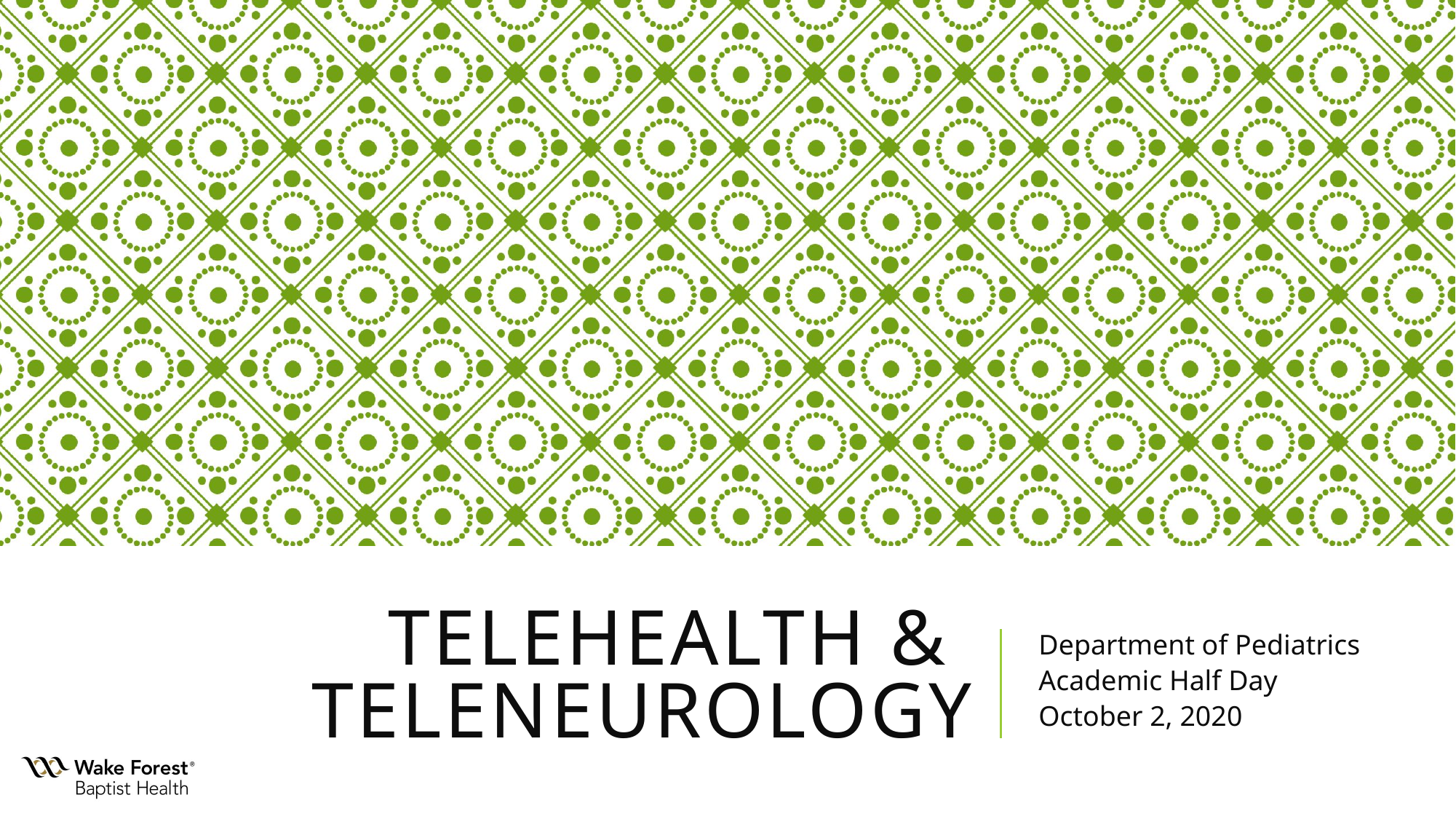

# Telehealth & Teleneurology
Department of Pediatrics
Academic Half Day
October 2, 2020

## Slide 2
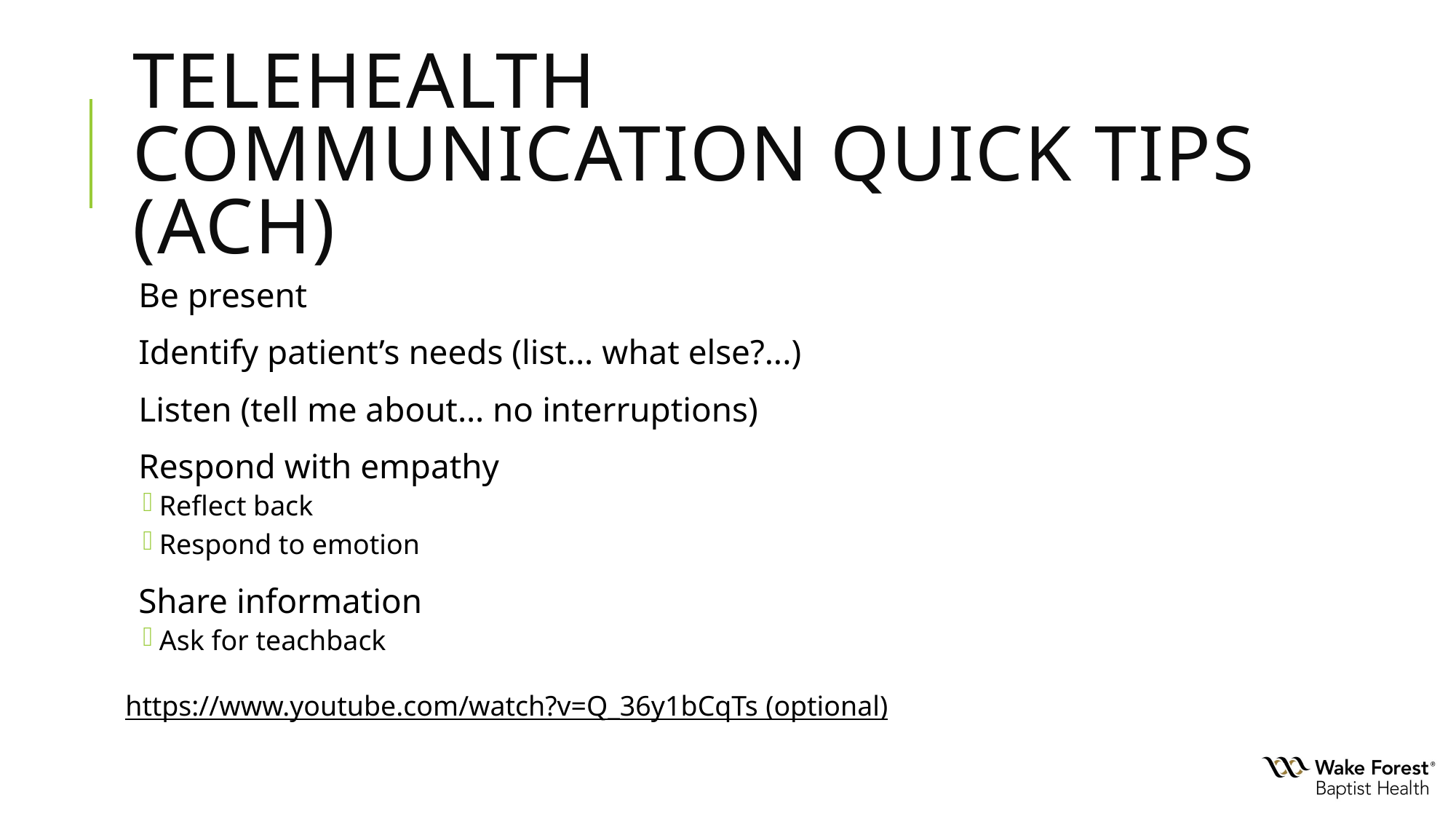

# Telehealth Communication Quick Tips (ACH)
Be present
Identify patient’s needs (list… what else?...)
Listen (tell me about… no interruptions)
Respond with empathy
Reflect back
Respond to emotion
Share information
Ask for teachback
https://www.youtube.com/watch?v=Q_36y1bCqTs (optional)

## Slide 3
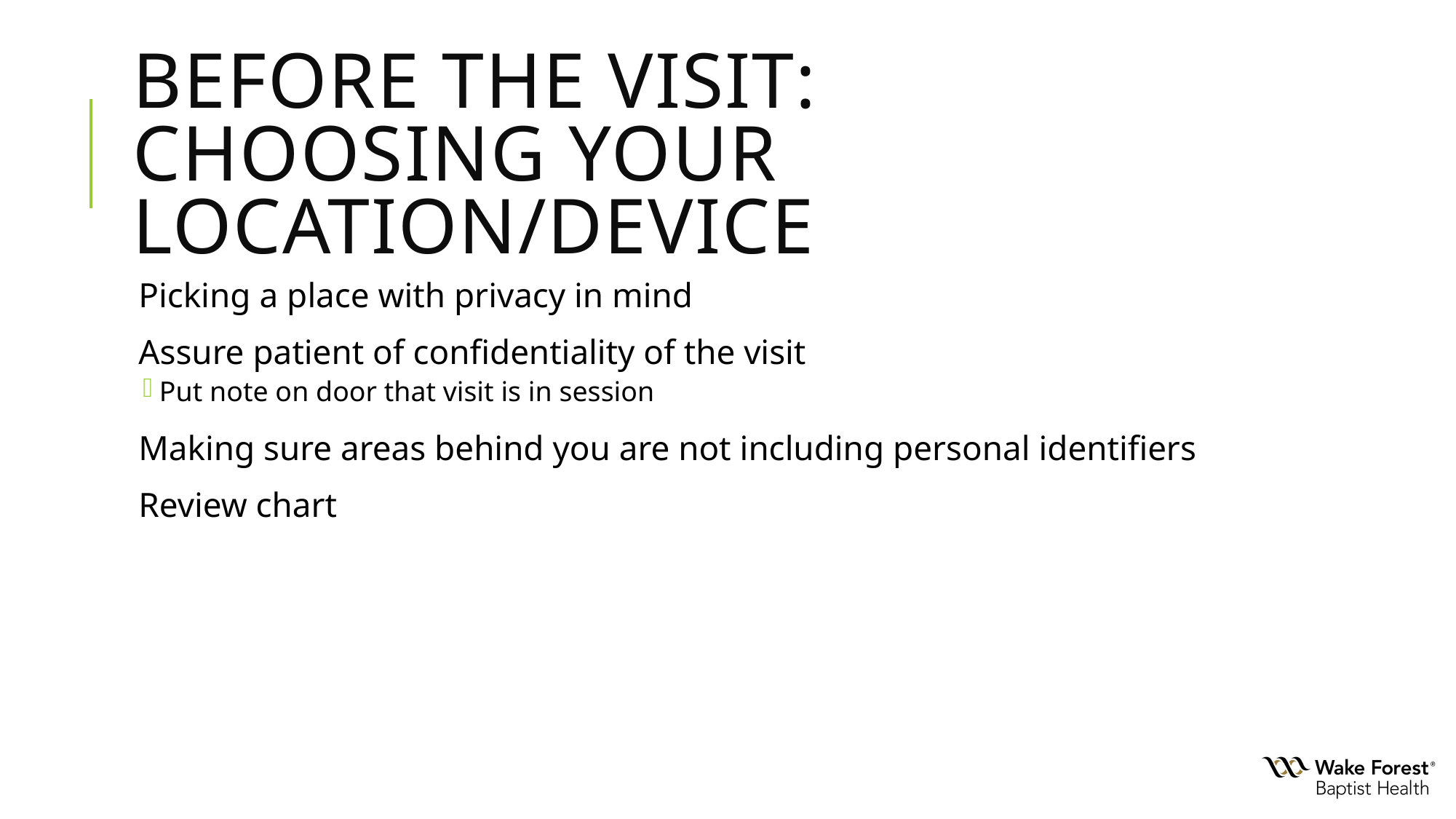

# Before the Visit: Choosing Your Location/Device
Picking a place with privacy in mind
Assure patient of confidentiality of the visit
Put note on door that visit is in session
Making sure areas behind you are not including personal identifiers
Review chart
3

## Slide 4
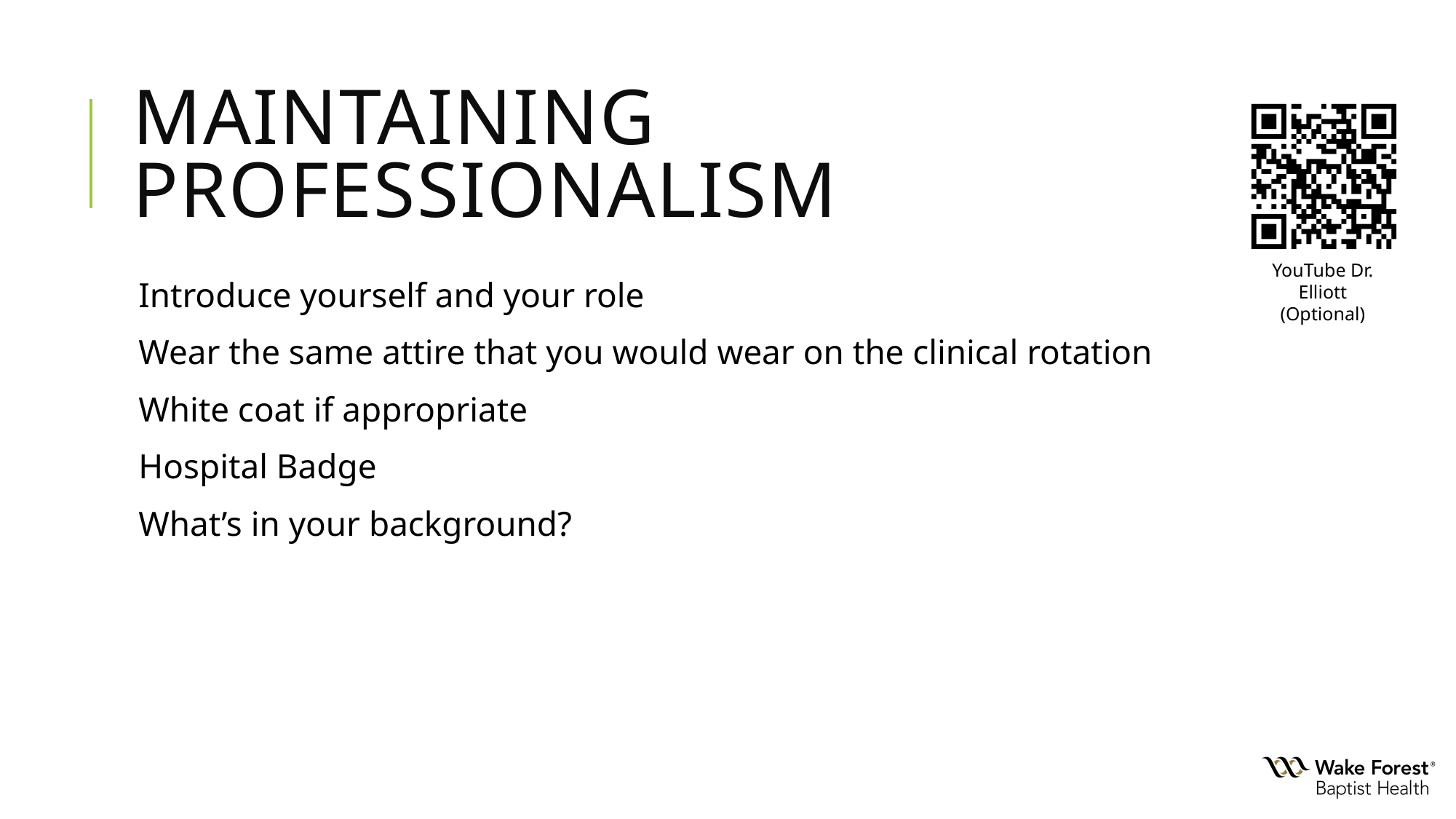

# Maintaining Professionalism
YouTube Dr. Elliott
(Optional)
Introduce yourself and your role
Wear the same attire that you would wear on the clinical rotation
White coat if appropriate
Hospital Badge
What’s in your background?
4

## Slide 5
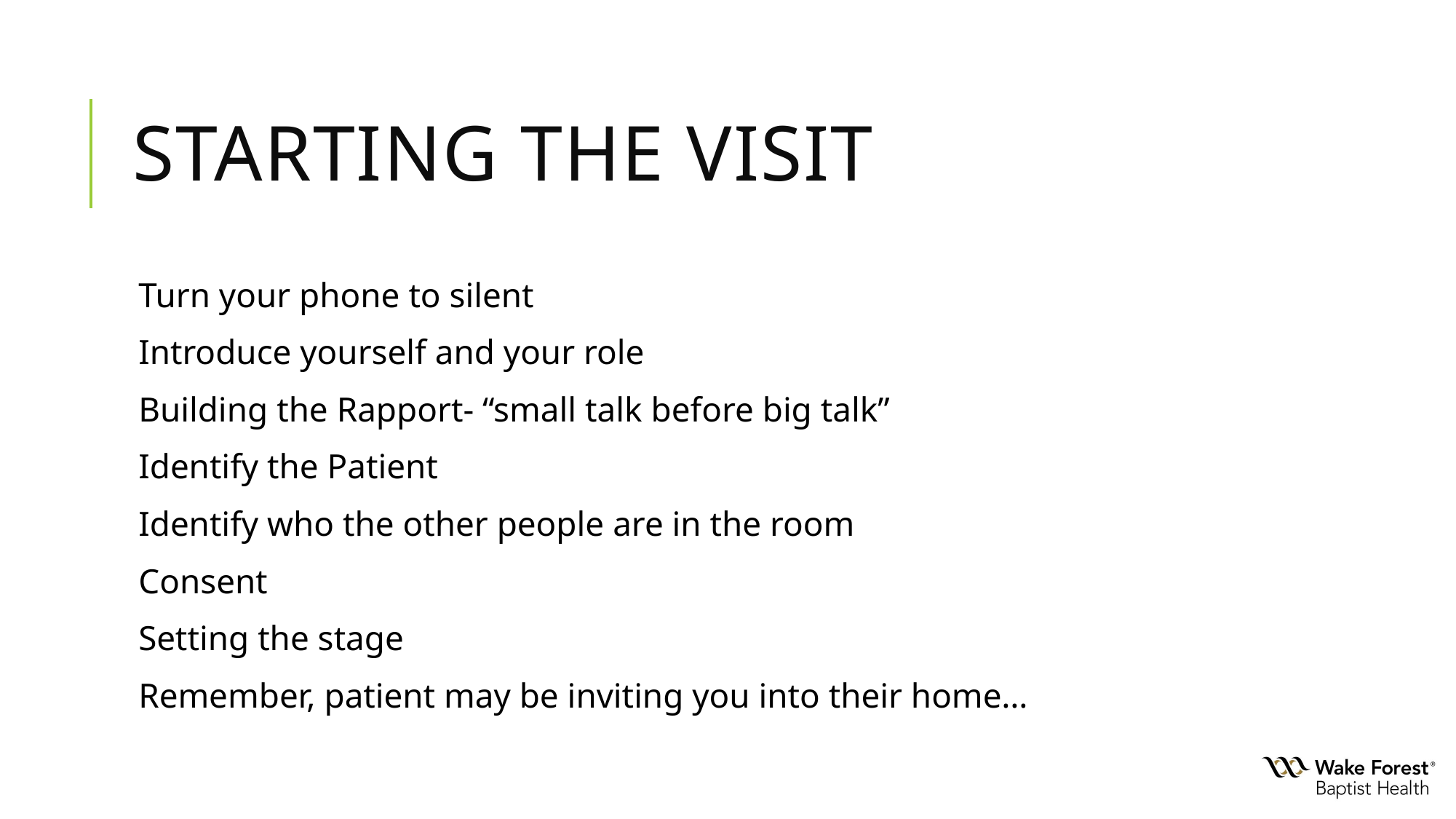

# Starting the Visit
Turn your phone to silent
Introduce yourself and your role
Building the Rapport- “small talk before big talk”
Identify the Patient
Identify who the other people are in the room
Consent
Setting the stage
Remember, patient may be inviting you into their home…
5

## Slide 6
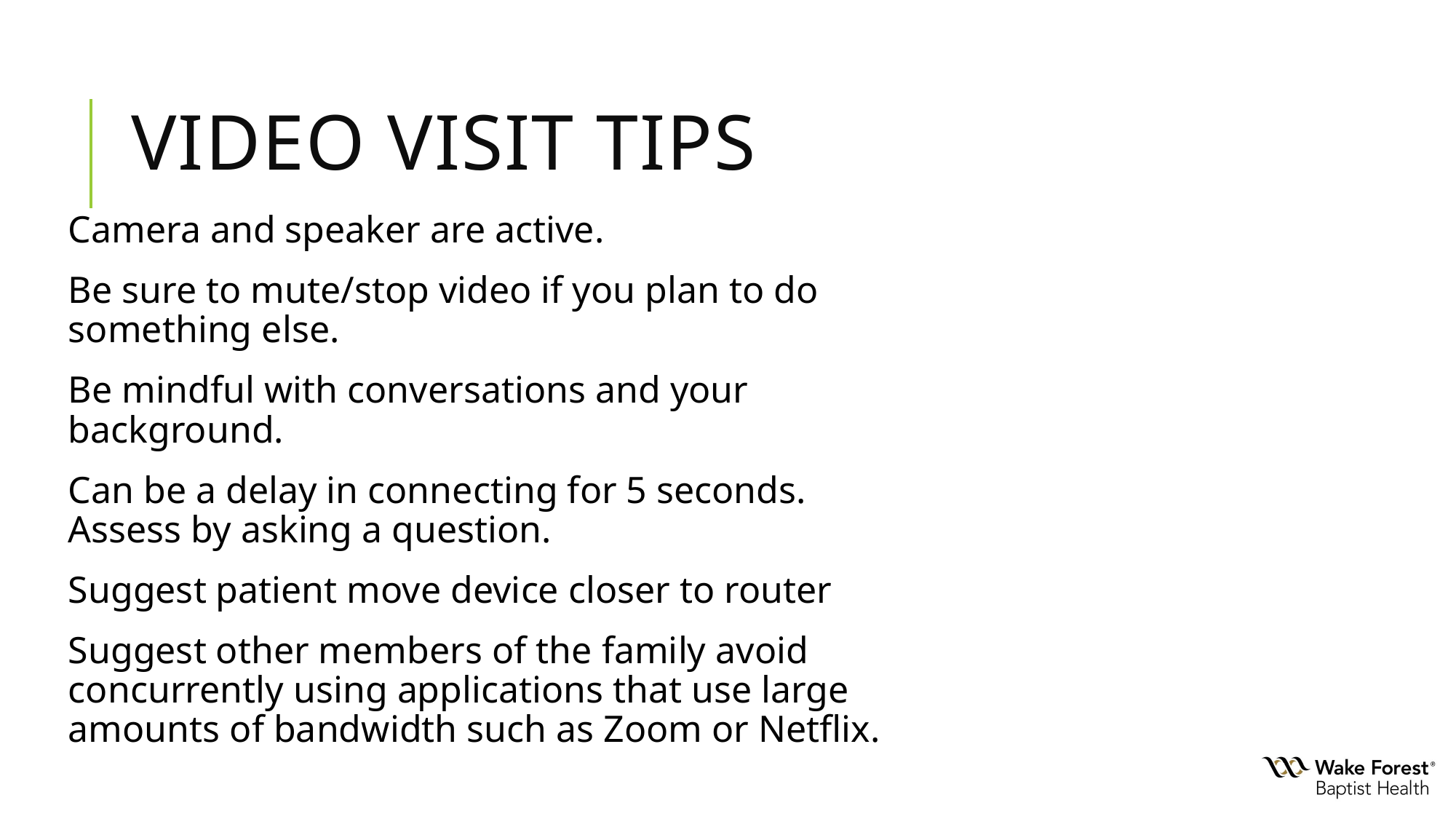

# Video Visit Tips
Camera and speaker are active.
Be sure to mute/stop video if you plan to do something else.
Be mindful with conversations and your background.
Can be a delay in connecting for 5 seconds. Assess by asking a question.
Suggest patient move device closer to router
Suggest other members of the family avoid concurrently using applications that use large amounts of bandwidth such as Zoom or Netflix.
6

## Slide 7
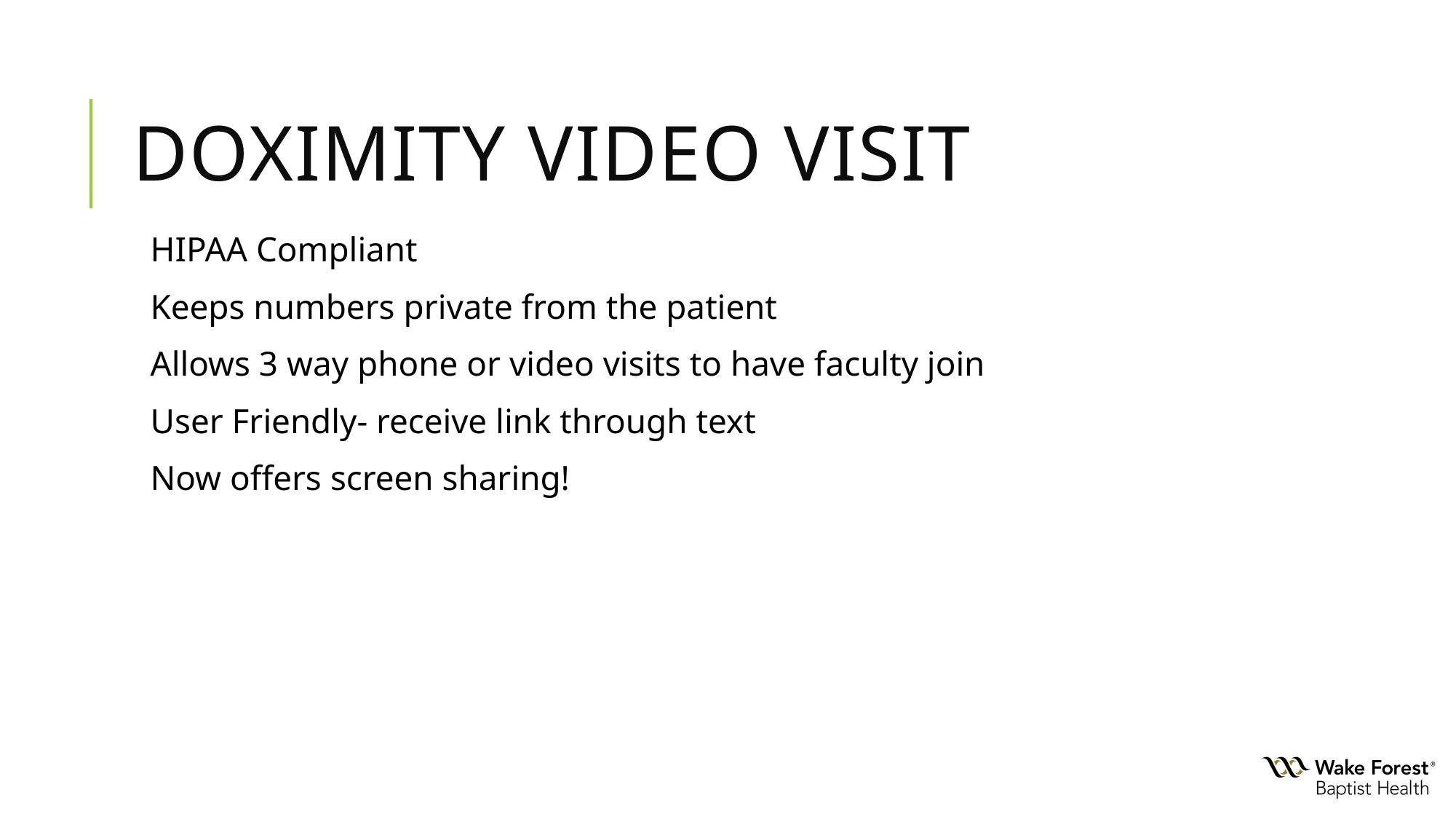

# DOXIMITY VIDEO VISIT
HIPAA Compliant
Keeps numbers private from the patient
Allows 3 way phone or video visits to have faculty join
User Friendly- receive link through text
Now offers screen sharing!
7

## Slide 8
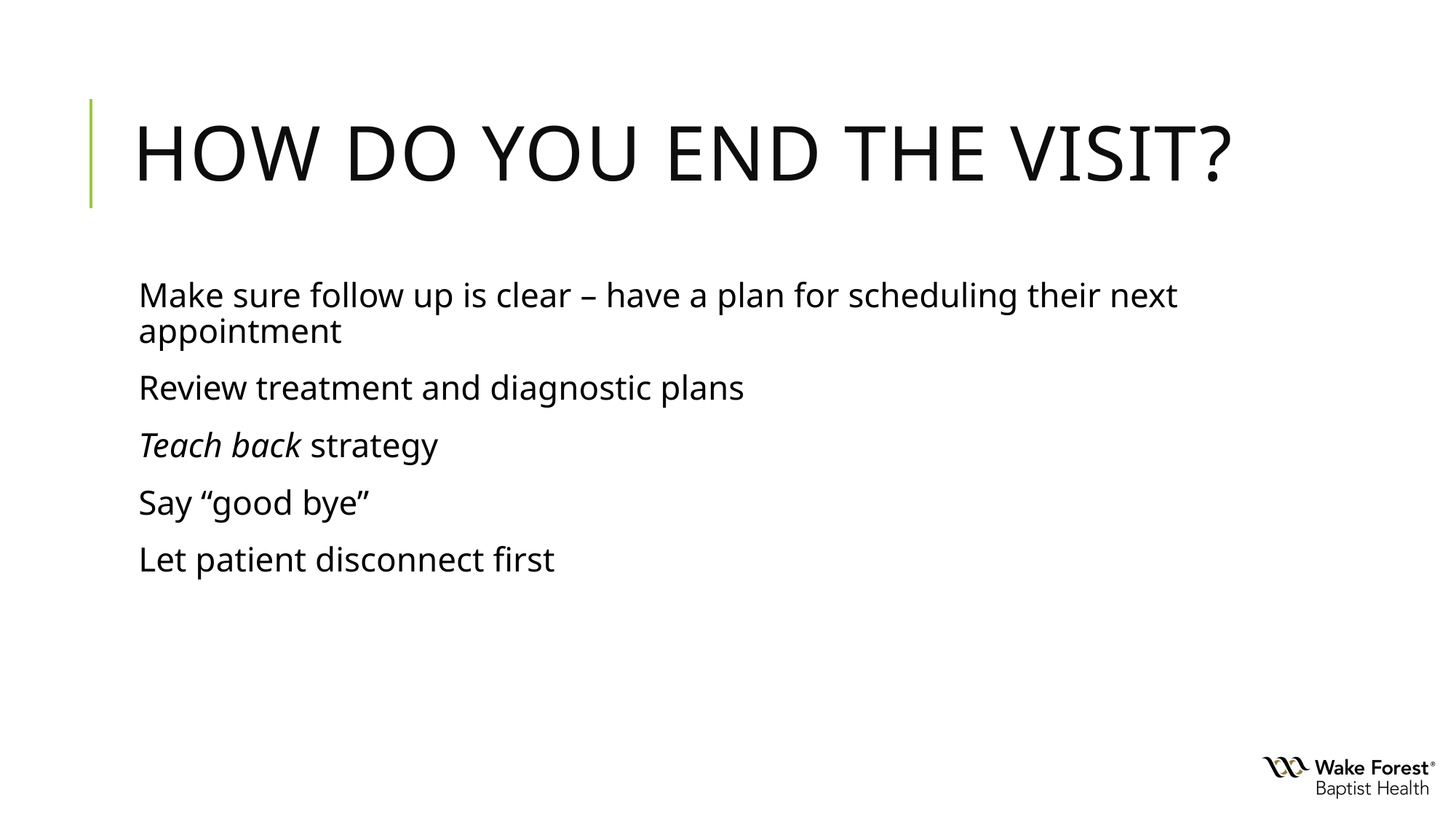

# How Do You End the Visit?
Make sure follow up is clear – have a plan for scheduling their next appointment
Review treatment and diagnostic plans
Teach back strategy
Say “good bye”
Let patient disconnect first
8

## Slide 9
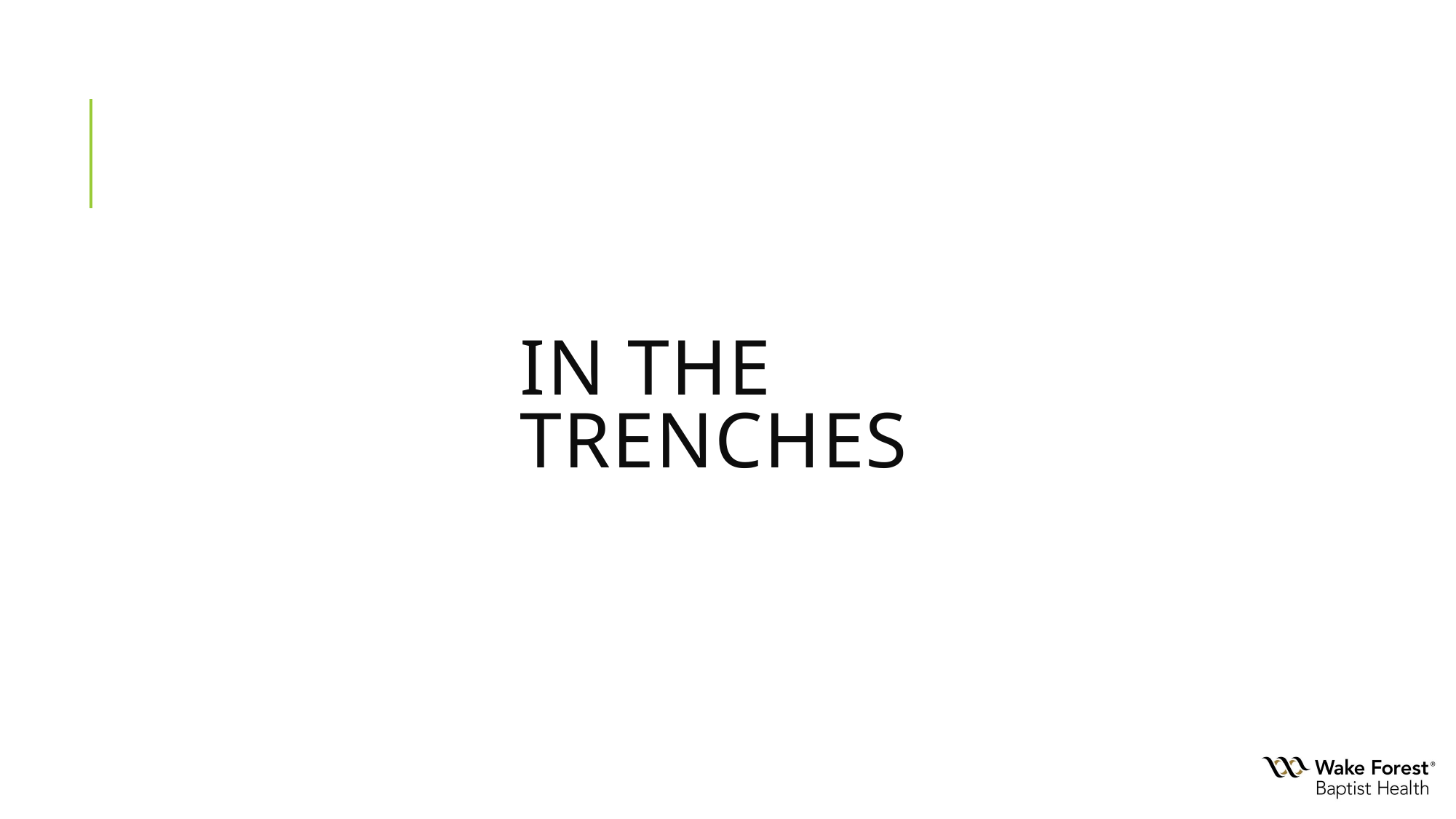

# In the Trenches
9

## Slide 10
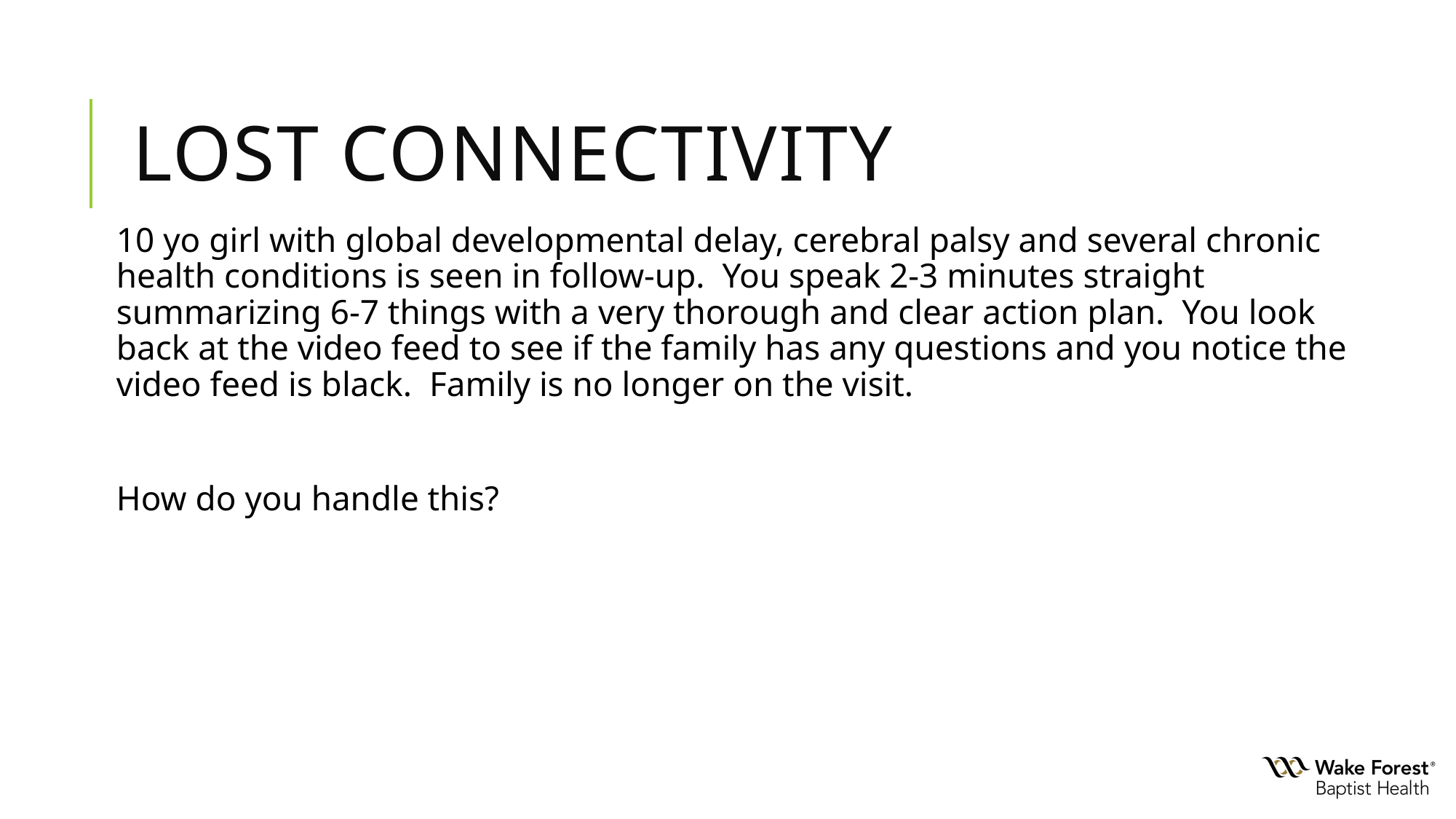

# Lost connectivity
10 yo girl with global developmental delay, cerebral palsy and several chronic health conditions is seen in follow-up. You speak 2-3 minutes straight summarizing 6-7 things with a very thorough and clear action plan. You look back at the video feed to see if the family has any questions and you notice the video feed is black. Family is no longer on the visit.
How do you handle this?

## Slide 11
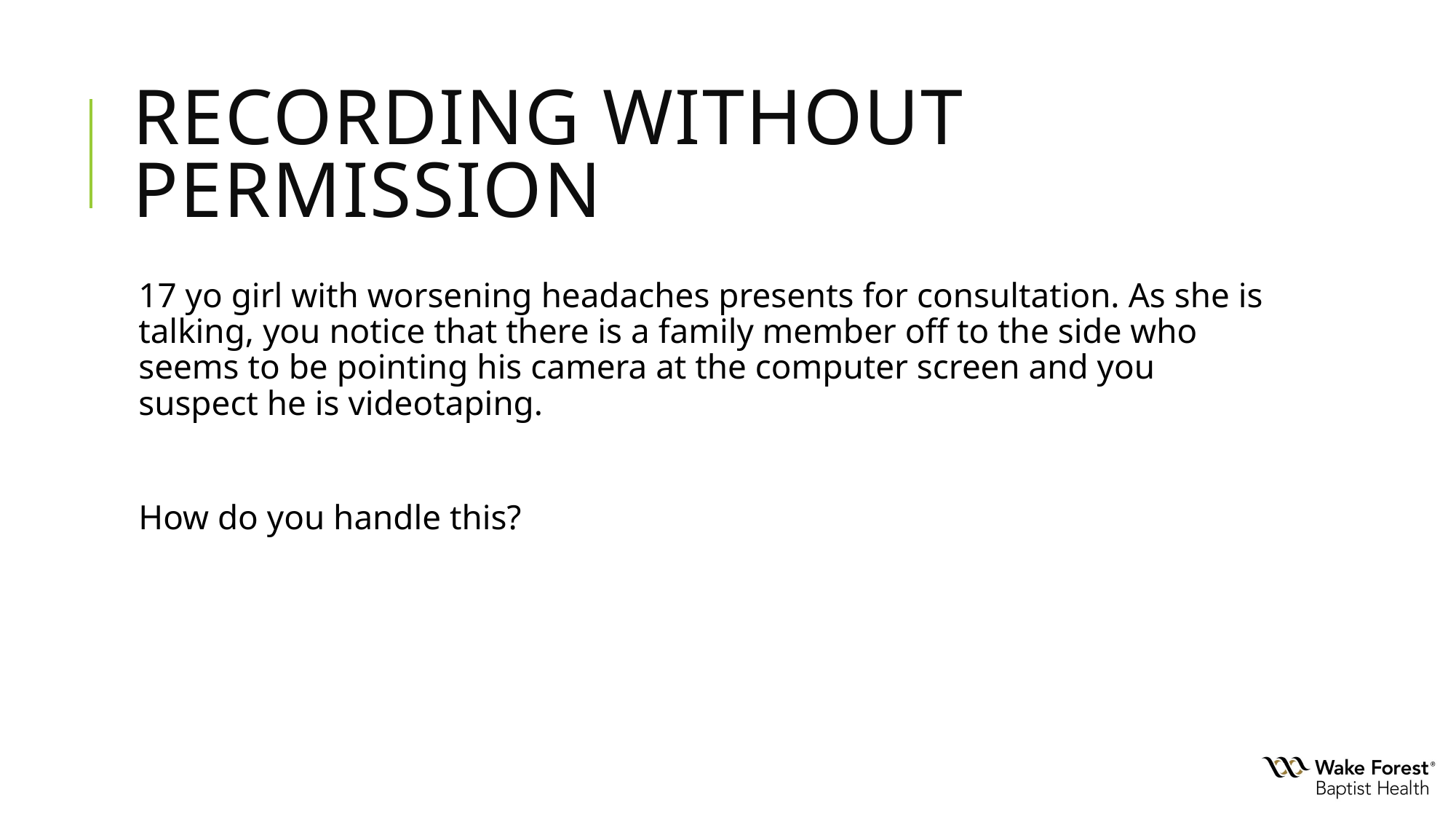

# Recording without Permission
17 yo girl with worsening headaches presents for consultation. As she is talking, you notice that there is a family member off to the side who seems to be pointing his camera at the computer screen and you suspect he is videotaping.
How do you handle this?
11

## Slide 12
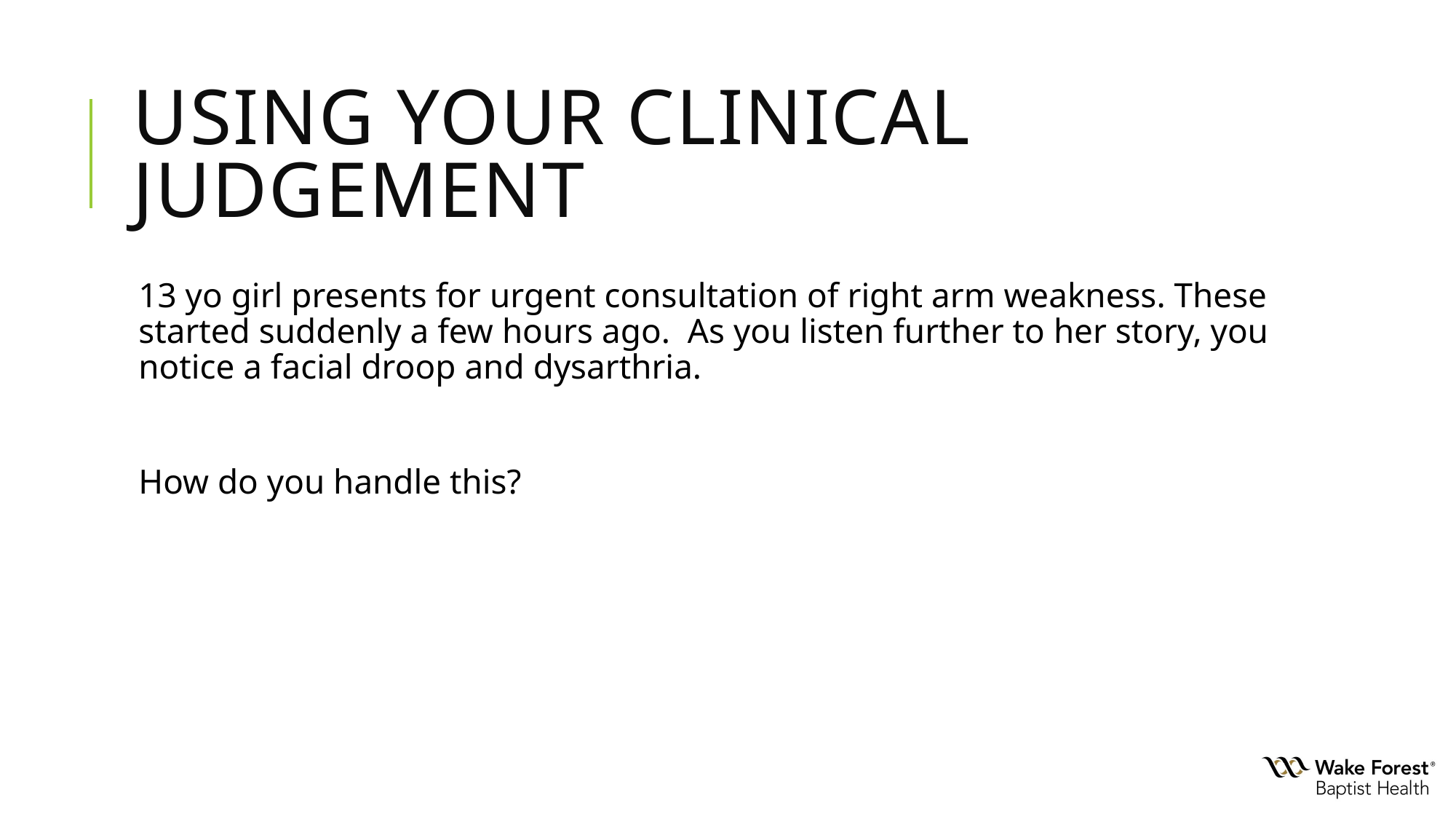

# Using your clinical judgement
13 yo girl presents for urgent consultation of right arm weakness. These started suddenly a few hours ago. As you listen further to her story, you notice a facial droop and dysarthria.
How do you handle this?
12

## Slide 13
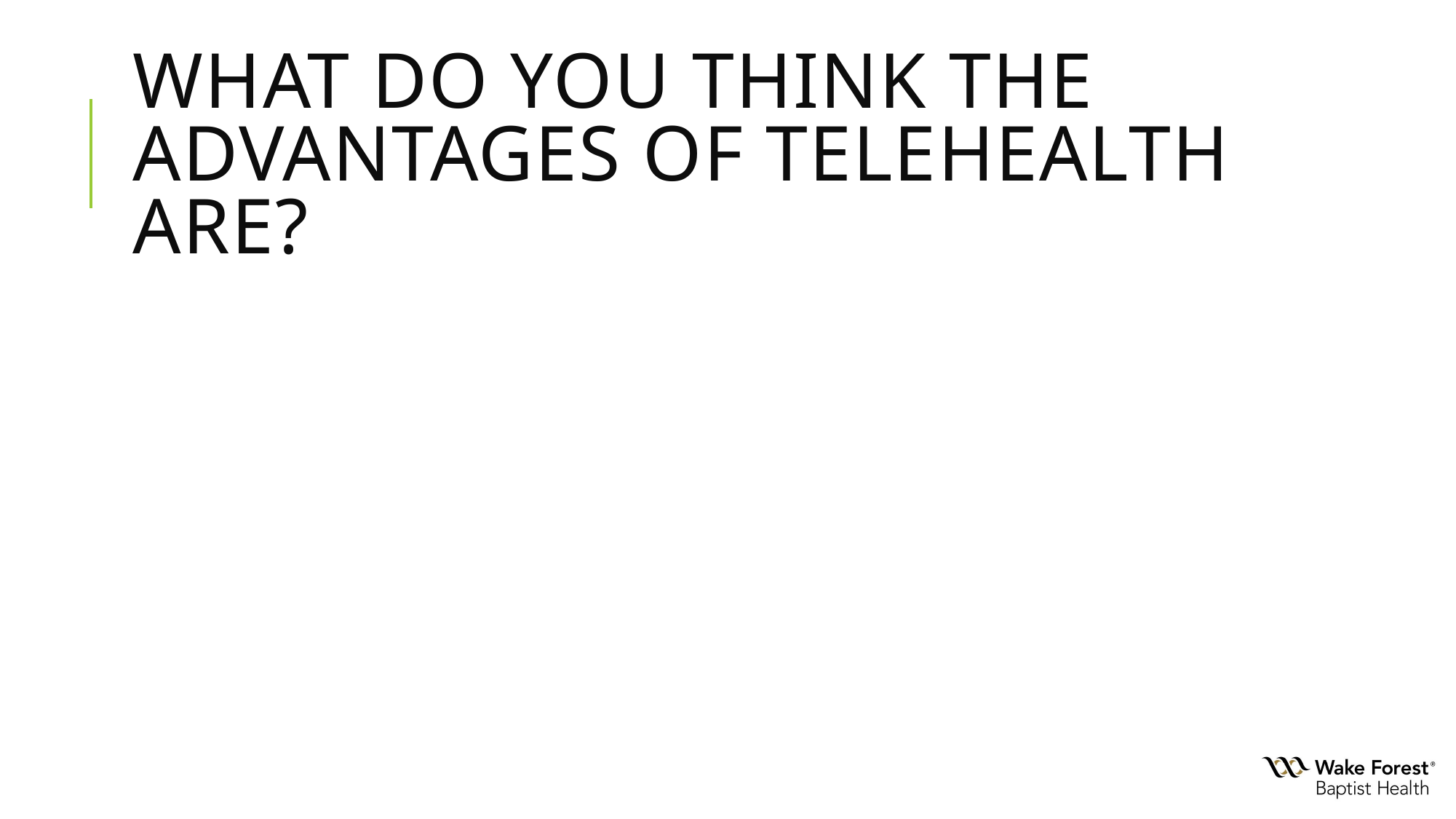

# What do you think the Advantages of Telehealth are?
13

## Slide 14
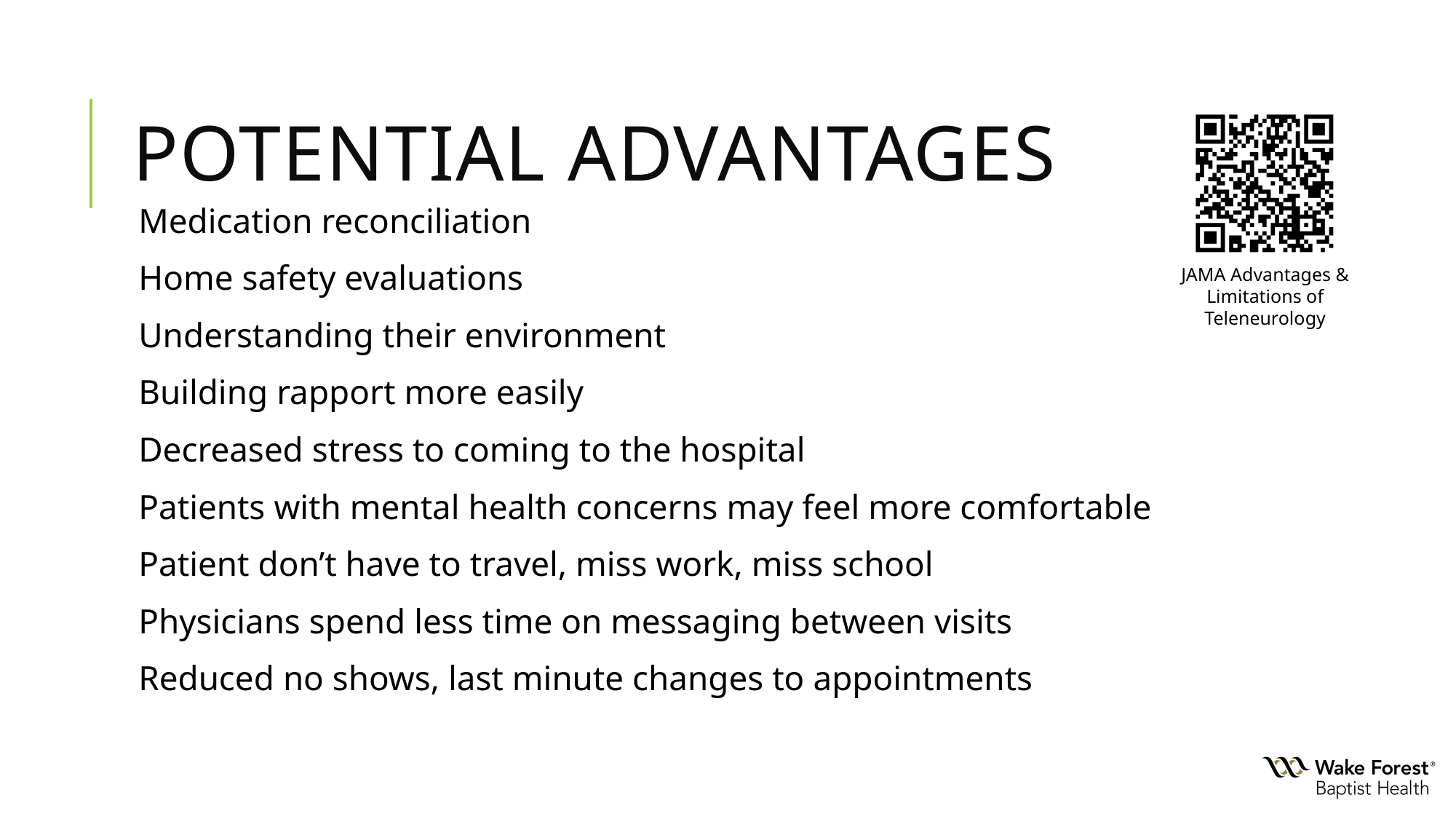

# Potential Advantages
Medication reconciliation
Home safety evaluations
Understanding their environment
Building rapport more easily
Decreased stress to coming to the hospital
Patients with mental health concerns may feel more comfortable
Patient don’t have to travel, miss work, miss school
Physicians spend less time on messaging between visits
Reduced no shows, last minute changes to appointments
JAMA Advantages & Limitations of Teleneurology
14

## Slide 15
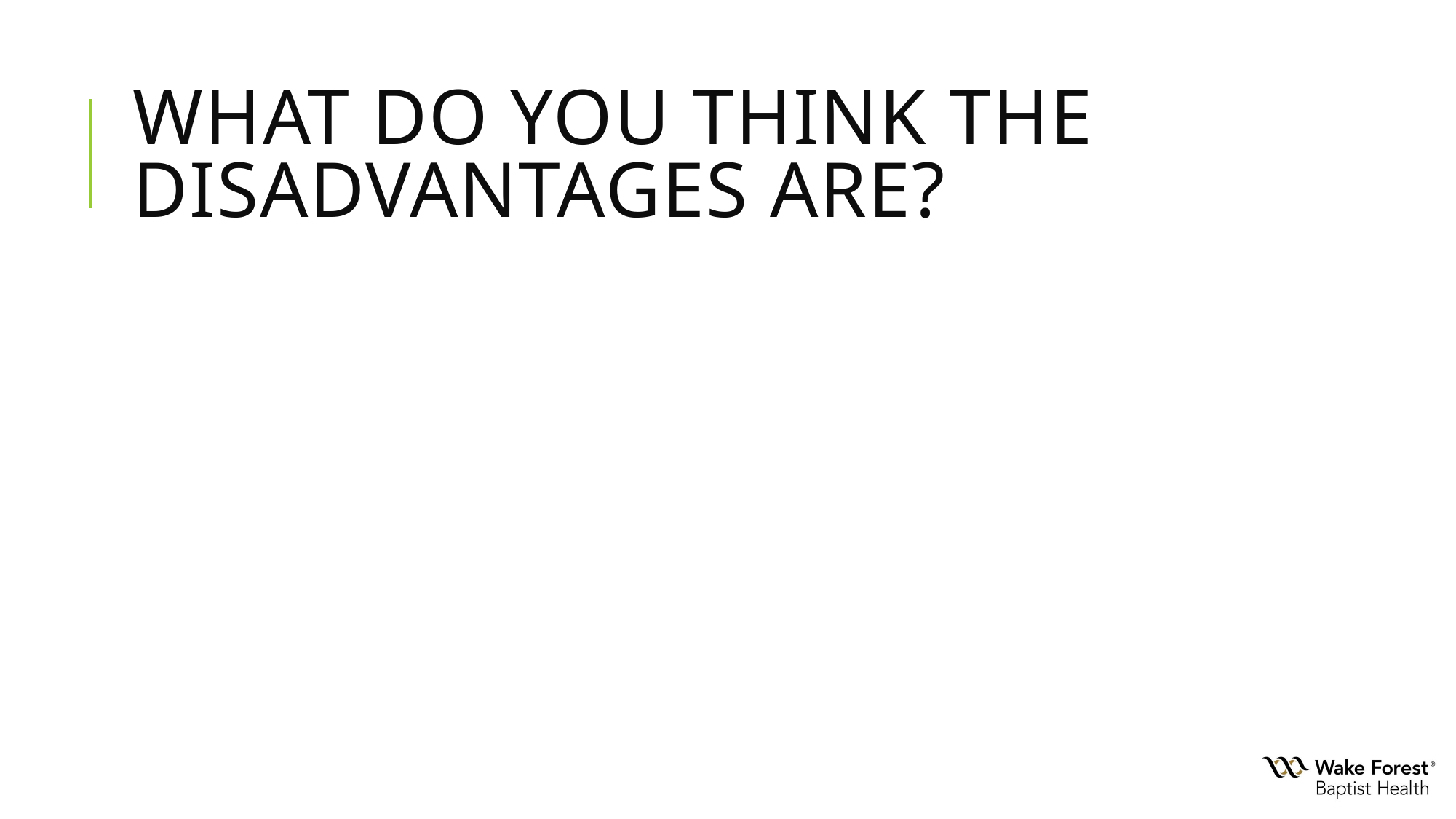

# What do you think the disadvantages are?
15

## Slide 16
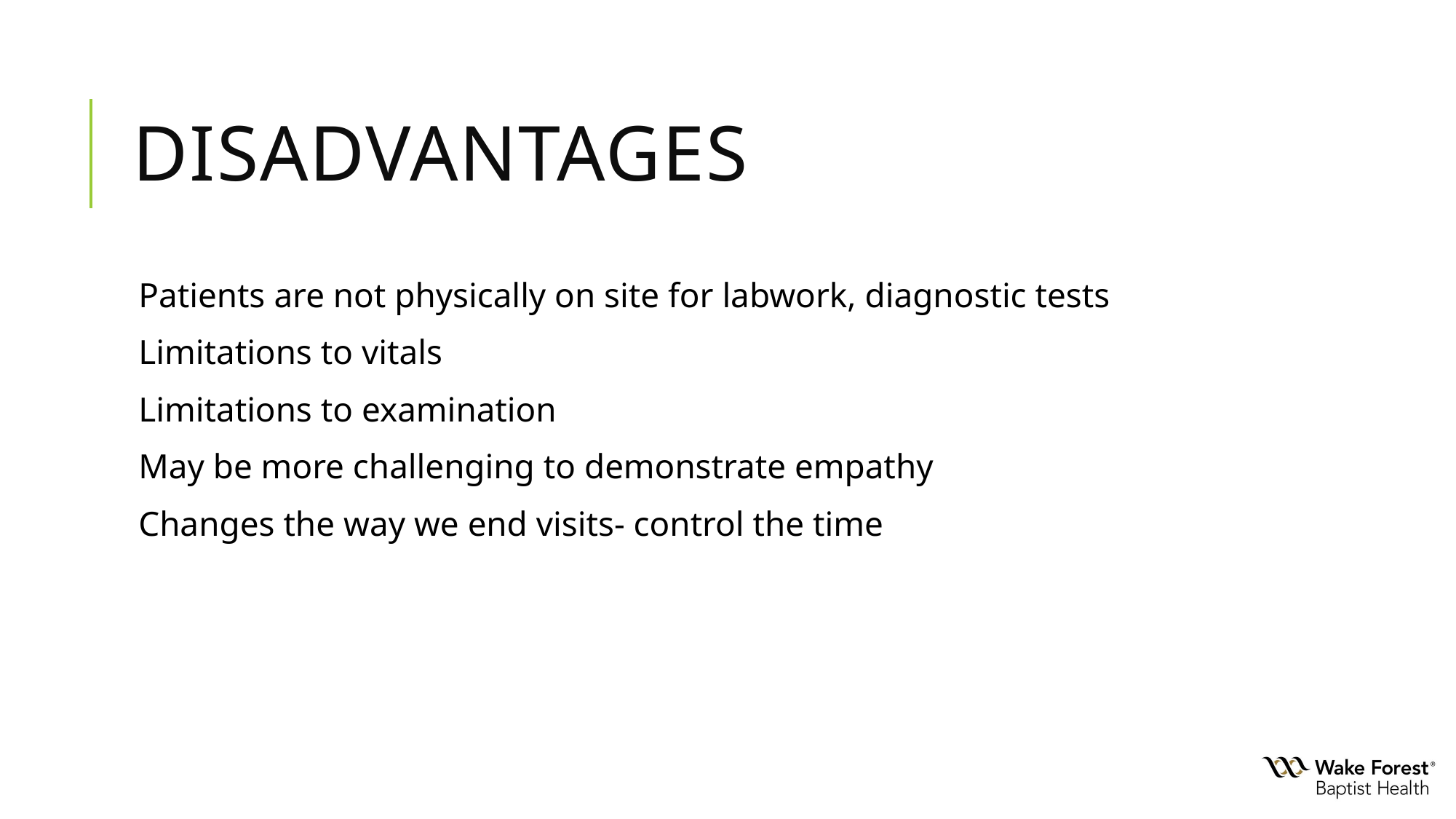

# Disadvantages
Patients are not physically on site for labwork, diagnostic tests
Limitations to vitals
Limitations to examination
May be more challenging to demonstrate empathy
Changes the way we end visits- control the time
16

## Slide 17
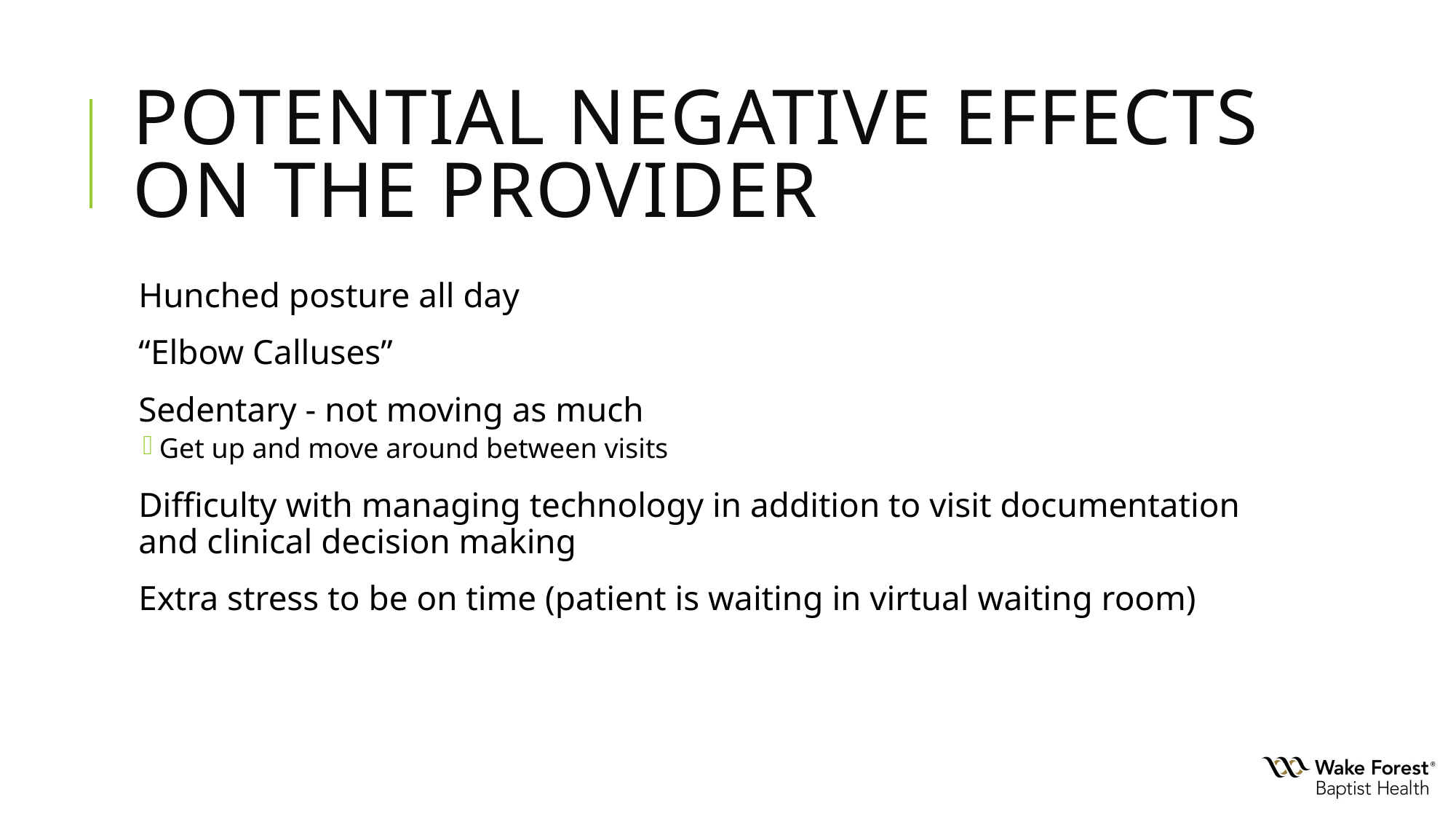

# Potential Negative Effects on the Provider
Hunched posture all day
“Elbow Calluses”
Sedentary - not moving as much
Get up and move around between visits
Difficulty with managing technology in addition to visit documentation and clinical decision making
Extra stress to be on time (patient is waiting in virtual waiting room)

## Slide 18
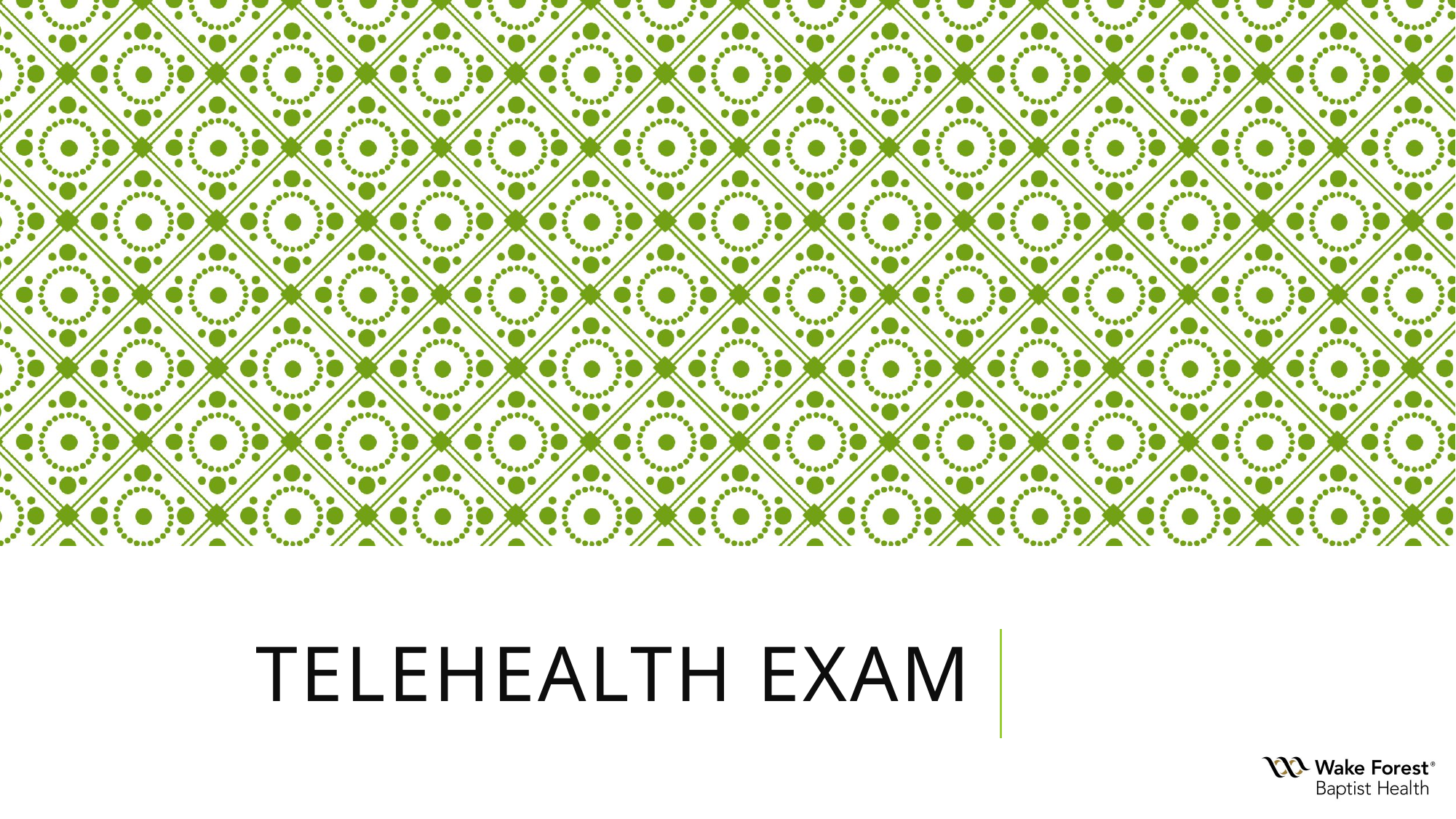

# Telehealth exam

## Slide 19
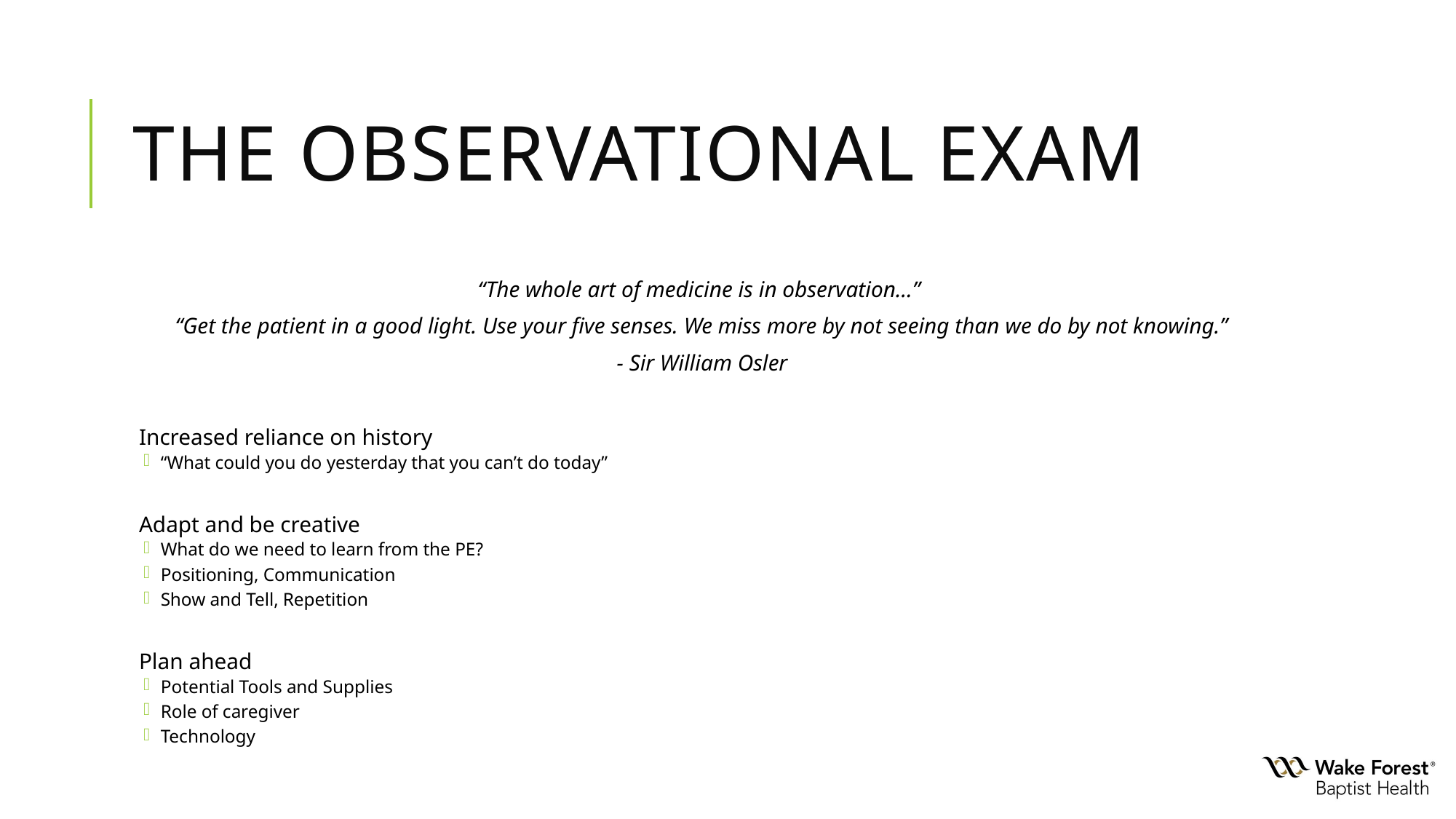

# The Observational Exam
“The whole art of medicine is in observation…”
“Get the patient in a good light. Use your five senses. We miss more by not seeing than we do by not knowing.”
- Sir William Osler
Increased reliance on history
“What could you do yesterday that you can’t do today”
Adapt and be creative
What do we need to learn from the PE?
Positioning, Communication
Show and Tell, Repetition
Plan ahead
Potential Tools and Supplies
Role of caregiver
Technology
19

## Slide 20
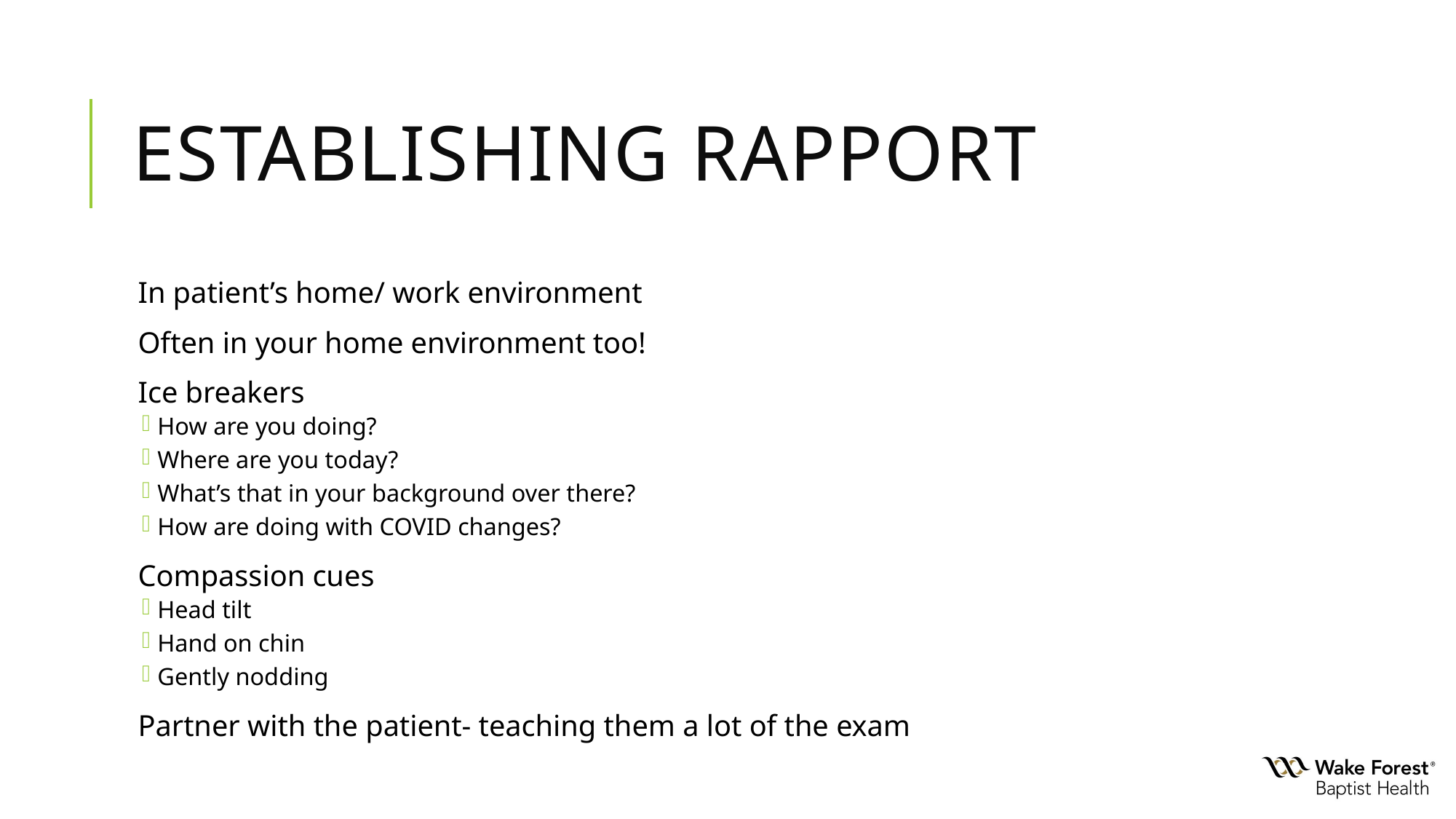

# Establishing Rapport
In patient’s home/ work environment
Often in your home environment too!
Ice breakers
How are you doing?
Where are you today?
What’s that in your background over there?
How are doing with COVID changes?
Compassion cues
Head tilt
Hand on chin
Gently nodding
Partner with the patient- teaching them a lot of the exam
20

## Slide 21
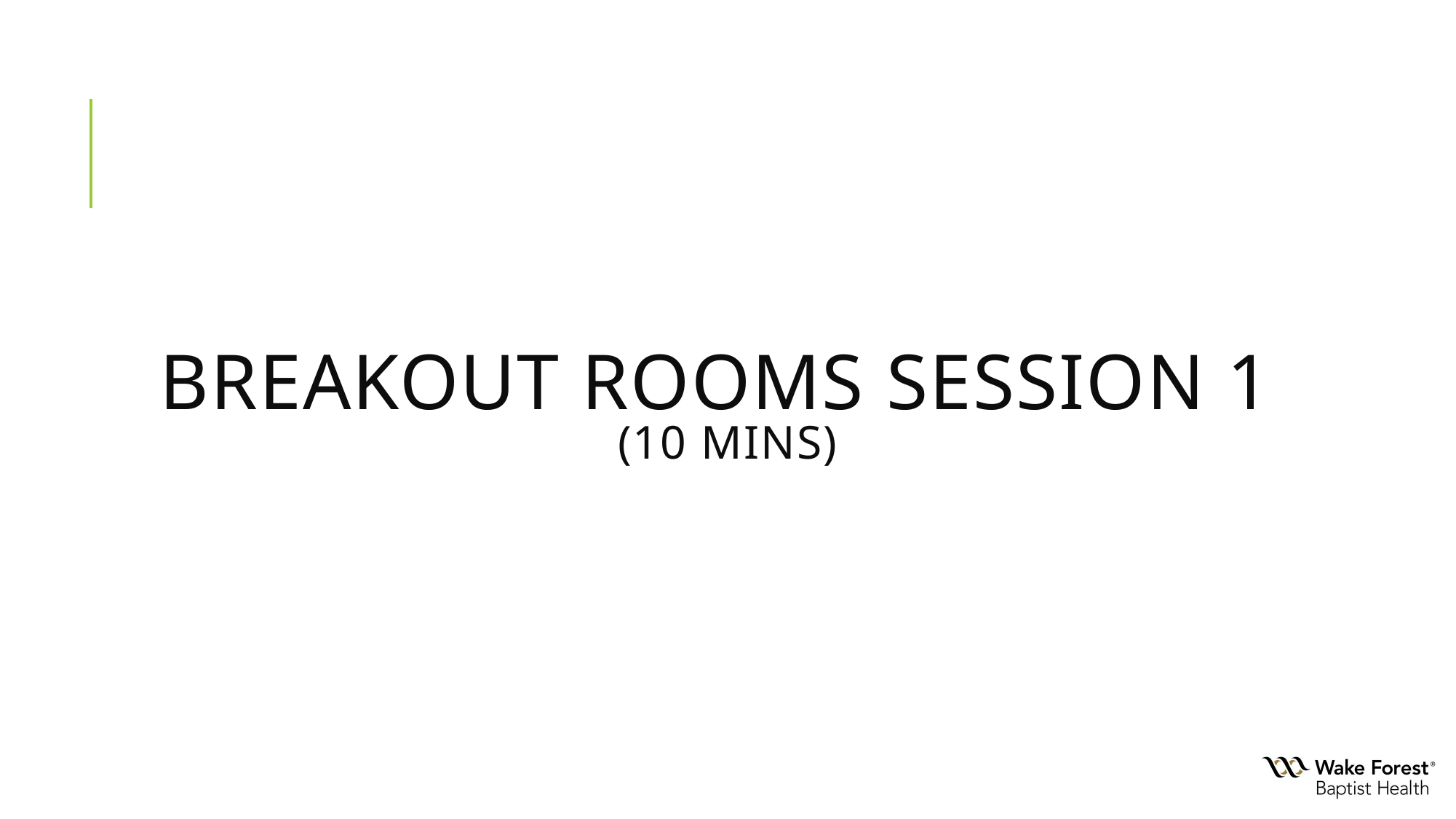

# Breakout rooms session 1 (10 mins)

## Slide 22
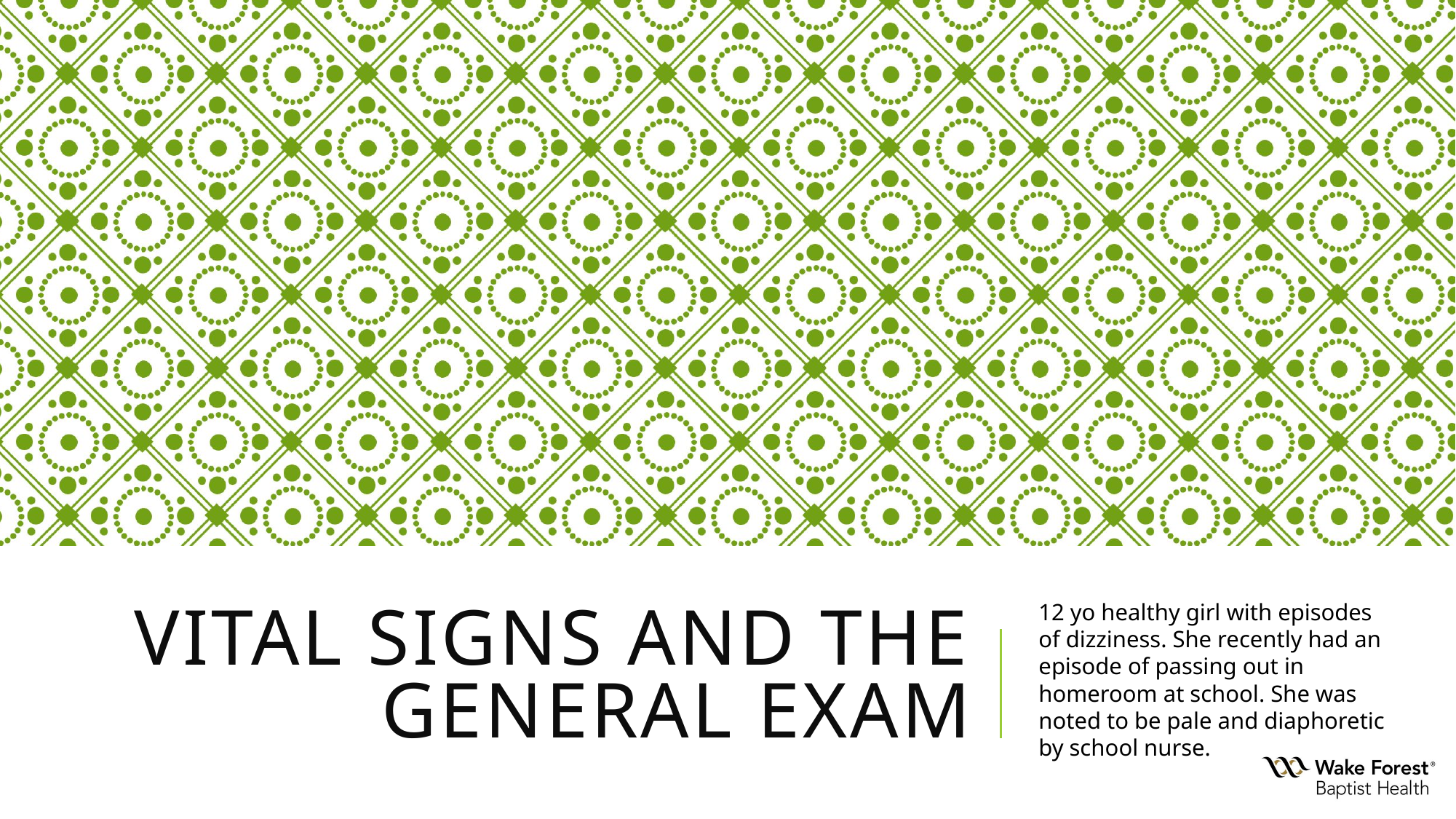

# Vital Signs and the General Exam
12 yo healthy girl with episodes of dizziness. She recently had an episode of passing out in homeroom at school. She was noted to be pale and diaphoretic by school nurse.

## Slide 23
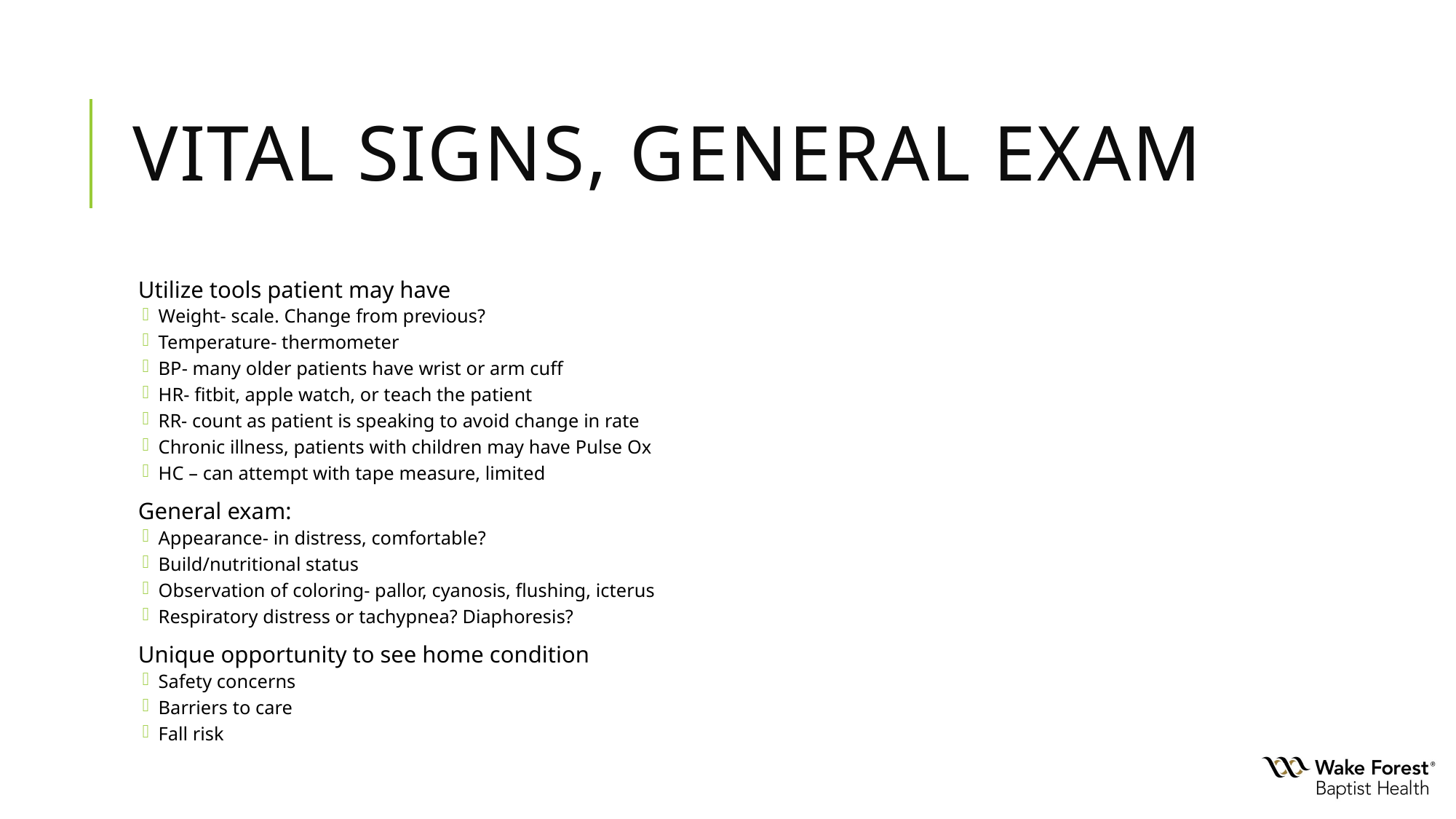

# Vital Signs, General Exam
Utilize tools patient may have
Weight- scale. Change from previous?
Temperature- thermometer
BP- many older patients have wrist or arm cuff
HR- fitbit, apple watch, or teach the patient
RR- count as patient is speaking to avoid change in rate
Chronic illness, patients with children may have Pulse Ox
HC – can attempt with tape measure, limited
General exam:
Appearance- in distress, comfortable?
Build/nutritional status
Observation of coloring- pallor, cyanosis, flushing, icterus
Respiratory distress or tachypnea? Diaphoresis?
Unique opportunity to see home condition
Safety concerns
Barriers to care
Fall risk

## Slide 24
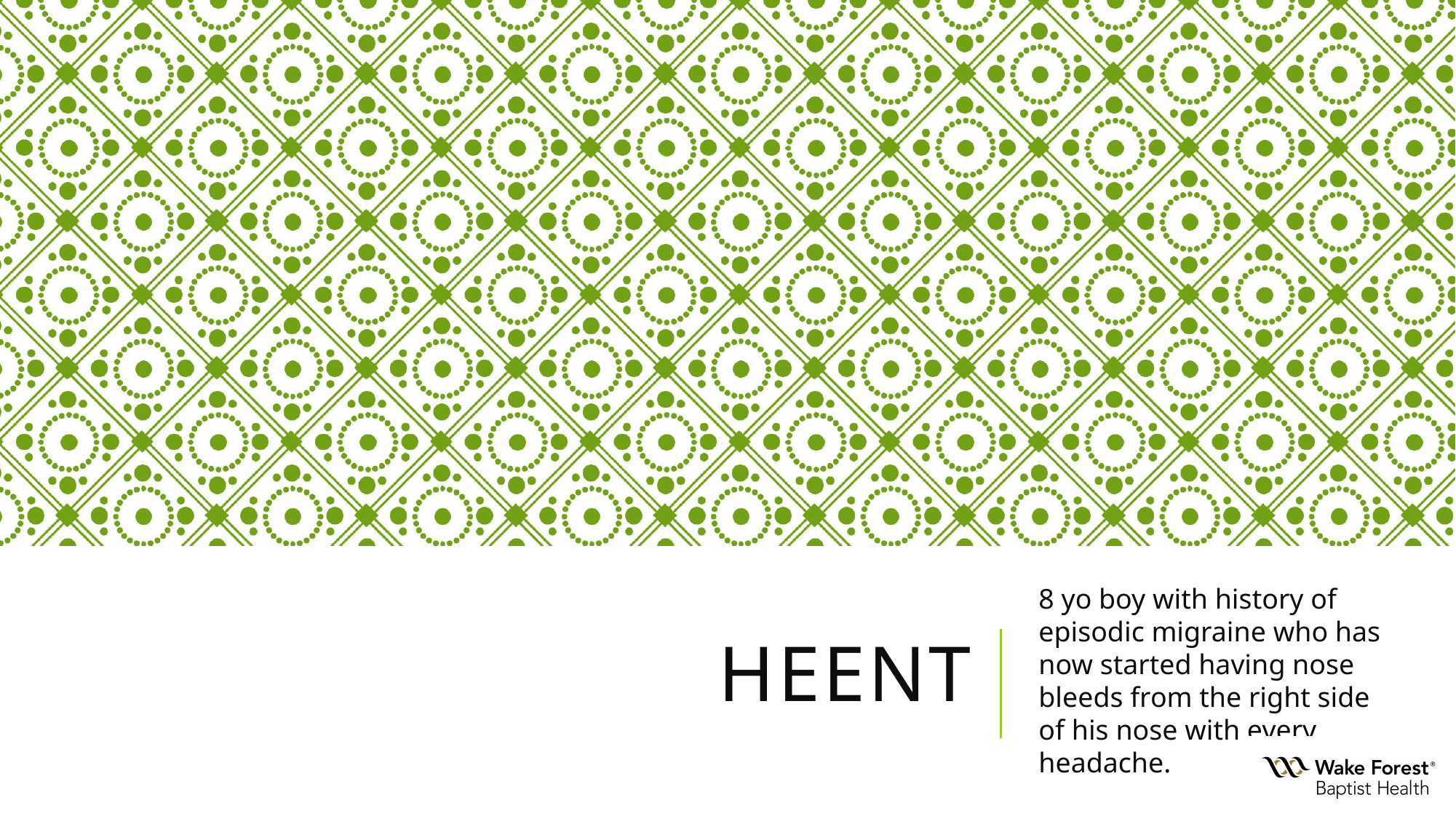

# HEENT
8 yo boy with history of episodic migraine who has now started having nose bleeds from the right side of his nose with every headache.
24

## Slide 25
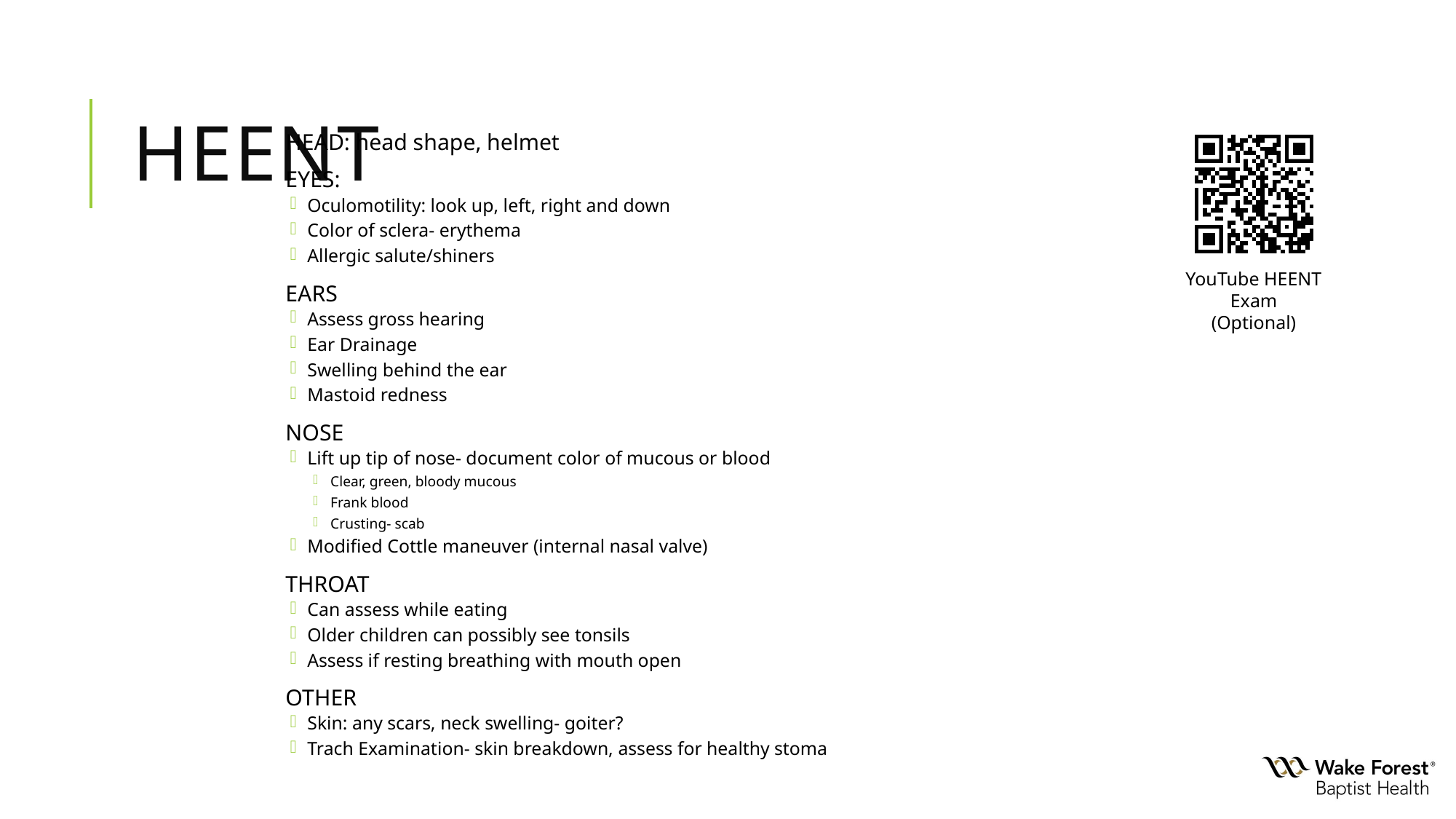

# HEENT
HEAD: head shape, helmet
EYES:
Oculomotility: look up, left, right and down
Color of sclera- erythema
Allergic salute/shiners
EARS
Assess gross hearing
Ear Drainage
Swelling behind the ear
Mastoid redness
NOSE
Lift up tip of nose- document color of mucous or blood
Clear, green, bloody mucous
Frank blood
Crusting- scab
Modified Cottle maneuver (internal nasal valve)
THROAT
Can assess while eating
Older children can possibly see tonsils
Assess if resting breathing with mouth open
OTHER
Skin: any scars, neck swelling- goiter?
Trach Examination- skin breakdown, assess for healthy stoma
YouTube HEENT Exam
(Optional)
25

## Slide 26
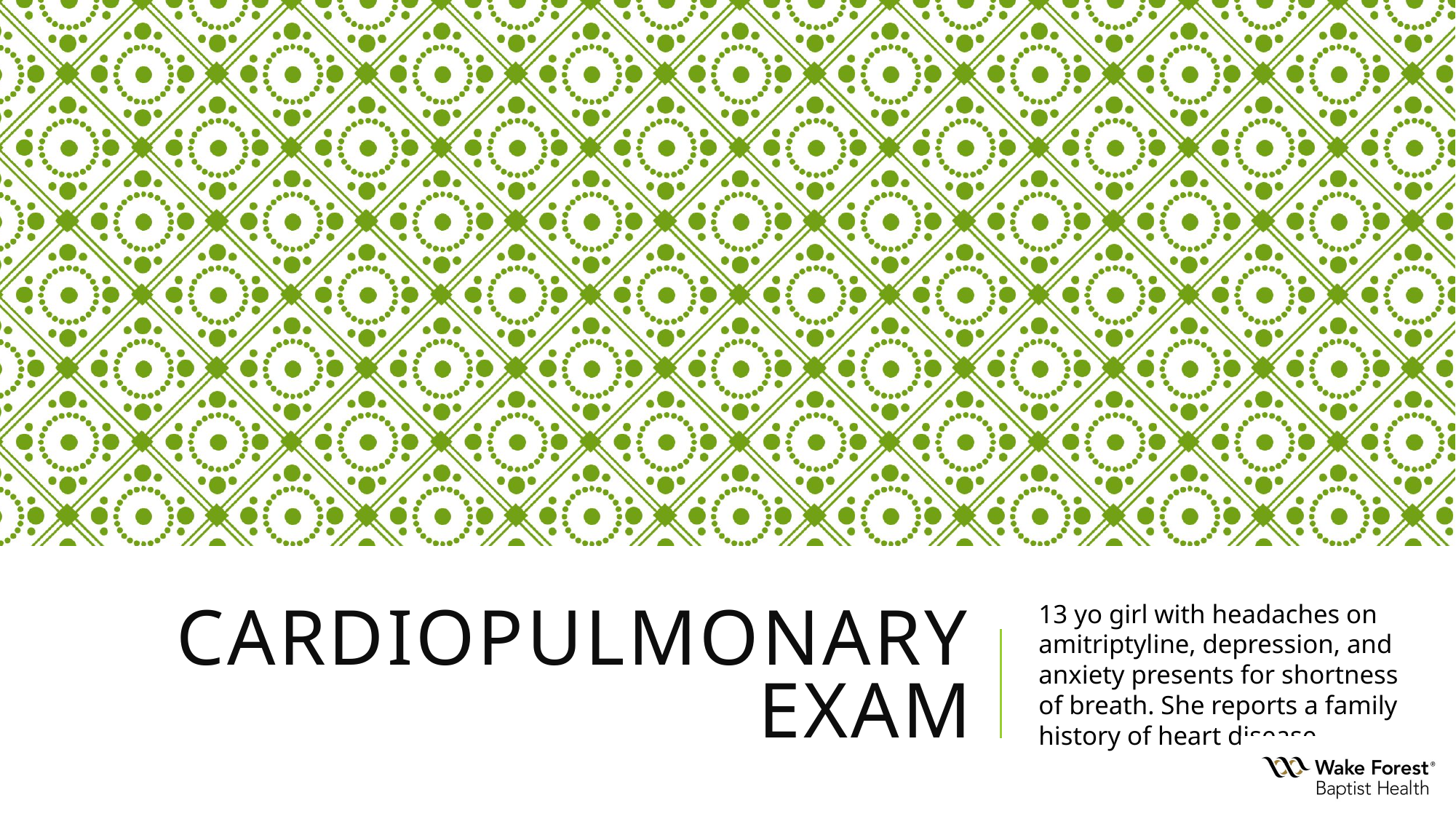

# Cardiopulmonary Exam
13 yo girl with headaches on amitriptyline, depression, and anxiety presents for shortness of breath. She reports a family history of heart disease.

## Slide 27
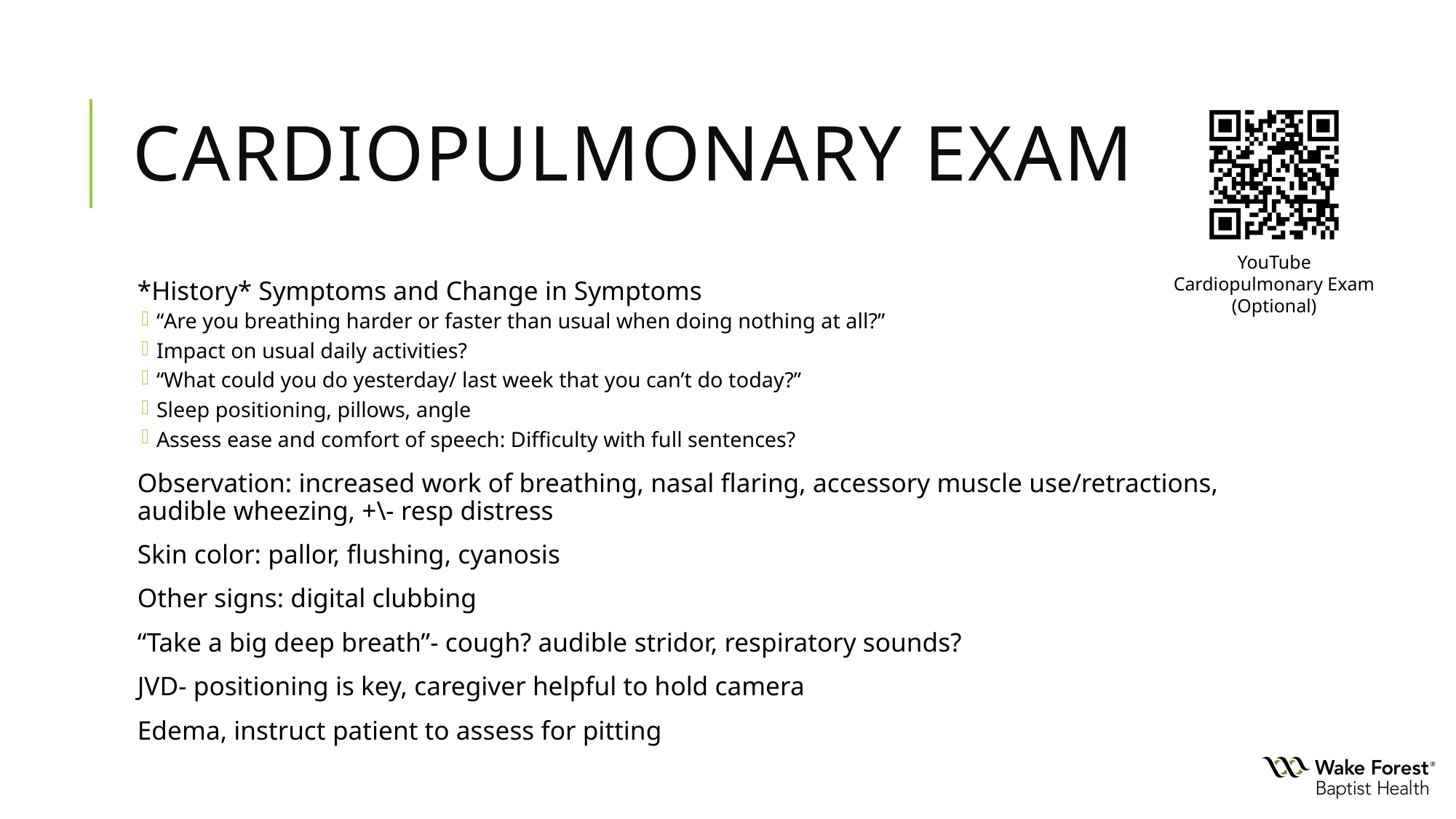

# Cardiopulmonary Exam
YouTube Cardiopulmonary Exam
(Optional)
*History* Symptoms and Change in Symptoms
“Are you breathing harder or faster than usual when doing nothing at all?”
Impact on usual daily activities?
“What could you do yesterday/ last week that you can’t do today?”
Sleep positioning, pillows, angle
Assess ease and comfort of speech: Difficulty with full sentences?
Observation: increased work of breathing, nasal flaring, accessory muscle use/retractions, audible wheezing, +\- resp distress
Skin color: pallor, flushing, cyanosis
Other signs: digital clubbing
“Take a big deep breath”- cough? audible stridor, respiratory sounds?
JVD- positioning is key, caregiver helpful to hold camera
Edema, instruct patient to assess for pitting

## Slide 28
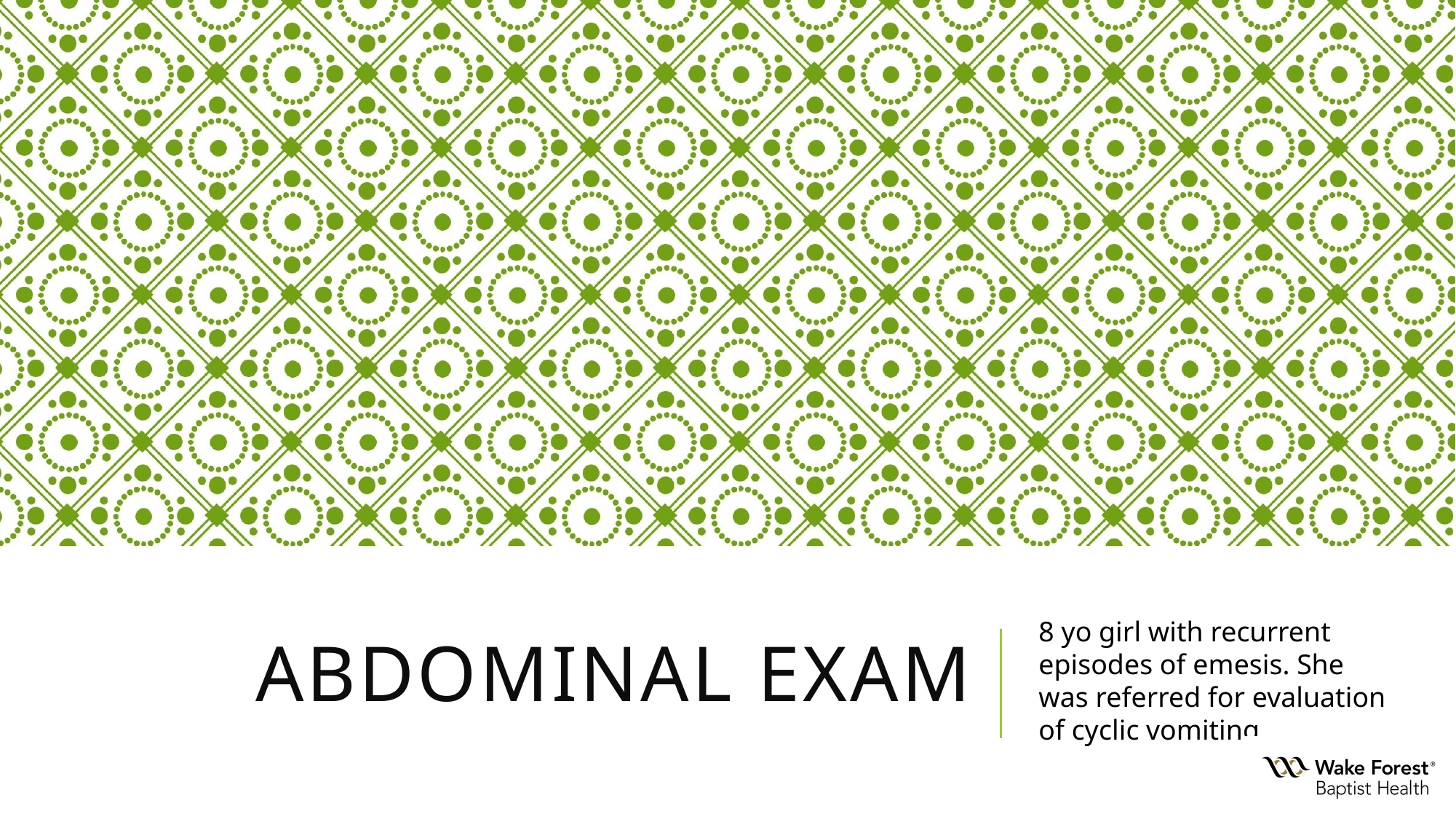

# Abdominal Exam
8 yo girl with recurrent episodes of emesis. She was referred for evaluation of cyclic vomiting.
28

## Slide 29
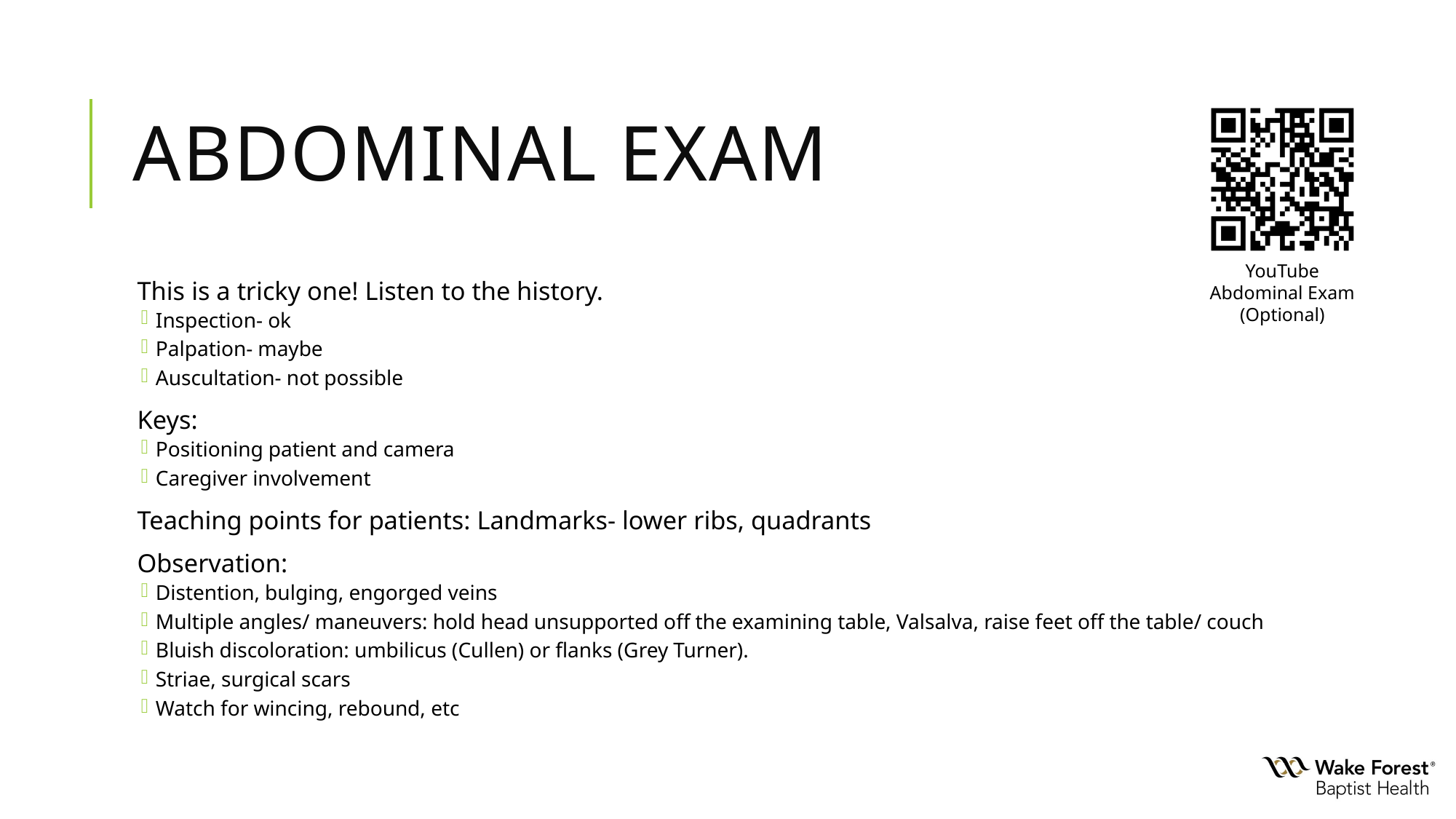

# Abdominal Exam
YouTube Abdominal Exam
(Optional)
This is a tricky one! Listen to the history.
Inspection- ok
Palpation- maybe
Auscultation- not possible
Keys:
Positioning patient and camera
Caregiver involvement
Teaching points for patients: Landmarks- lower ribs, quadrants
Observation:
Distention, bulging, engorged veins
Multiple angles/ maneuvers: hold head unsupported off the examining table, Valsalva, raise feet off the table/ couch
Bluish discoloration: umbilicus (Cullen) or flanks (Grey Turner).
Striae, surgical scars
Watch for wincing, rebound, etc
