# Supplementary material for: High-Yield, Case-Based, Interactive Workshop on Telehealth and Teleneurology With Pediatric Resident Physicians
Source: MedEdPORTAL. 2023 Aug 25;19:11340. doi: 10.15766/mep_2374-8265.11340 (PMC10450098; doi:10.15766/mep_2374-8265.11340)
Supplement: Supplementary file 1 — Facilitator Guide.docxLearner Guide.docxTeleneurology Cases.pptxTelehealth Introduction.pptxConference Evaluation.docx [file mep_2374-8265.11340-s001.zip › E. Conference Evaluation.docx]

| **WAKE FOREST** |  |  |  |  |  |  |  |  |  |  |  |  |
| --- | --- | --- | --- | --- | --- | --- | --- | --- | --- | --- | --- | --- |
| Evaluation: **Academic Half-Day** |  |  |  |  |  |  |  |  |  |  |  |  |
| Conference(s): | | | | |  |  |  |  |  |  |  |  |
| Responses: |  |  |  |  |  |  |  |  |  |  |  |  |
| **Presentation Evaluation (entire AHD)** | | | | | | | | | | | | |
|  | 1 | | 2 | | 3 | | 4 | | 5 | | Avg: | Std: |
| 1. How satisfied were you with the content of today's Academic Half Day* | *Not at all satisfied* | | *Slightly satisfied* | | *Moderately satisfied* | | *Very satisfied* | | *Extremely satisfied* | |  |  |
|  |  | |  | |  | |  | |  | |  |  |
|  | 1 | | 2 | | 3 | | 4 | | 5 | | Avg: | Std: |
| 2. How effective was the delivery of the content for today's academic half-day?* | *Not at all effective* | | *Not so effective* | | *Somewhat effective* | | *Very effective* | | *Extremely effective* | |  |  |
|  |  | |  | |  | |  | |  | |  |  |
| **Conference Material (entire AHD)** | | | | | | | | | | | | |
|  | 1 | | 2 | | 3 | | 4 | |  | | Avg: | Std: |
| 3. How helpful was today's conference in preparing you for boards?* | *Not at all helpful* | | *Somewhat helpful* | | *Helpful* | | *Very helpful* | | *N/A* | |  |  |
|  |  | |  | |  | |  | |  | |  |  |
|  | 1 | | 2 | | 3 | | 4 | | 5 | | Avg: | Std: |
| 4. How often do you anticipate that you will use the material presented in today's conference in clinical practice?* | *Never* | | *Almost never* | | *Occasionally/Sometimes* | | *Frequently* | | *Almost every time* | |  |  |
|  |  | |  | |  | |  | |  | |  |  |
| **Conference Feedback (entire AHD)** | | | | | | | | | | | | |
| What did you like best about today's conference? (Please feel free to comment on content, delivery, location/environment, speakers, etc.) |  | | | | | | | | | | | |
|  |  | | | | | | | | | | | |
|  |  | | | | | | | | | | | |
|  |  | | | | | | | | | | | |
|  |  | | | | | | | | | | | |
|  |  | | | | | | | | | | | |
|  |  | | | | | | | | | | | |
|  |  | | | | | | | | | | | |
|  |  | | | | | | | | | | | |
|  |  | | | | | | | | | | | |
|  |  | | | | | | | | | | | |
|  |  | | | | | | | | | | | |
|  |  | | | | | | | | | | | |
|  |  | | | | | | | | | | | |
|  |  | | | | | | | | | | | |
|  |  |  |  |  |  |  |  |  |  |  |  |  |
|  |  |  |  |  |  |  |  |  |  |  |  |  |
|  |  |  |  |  |  |  |  |  |  |  |  |  |
| How could this conference be improved? (Please feel free to comment on content, delivery, location/environment, speakers, etc.) |  | | | | | | | | | | | |
|  |  | | | | | | | | | | | |
|  |  | | | | | | | | | | | |
|  |  | | | | | | | | | | | |
|  |  | | | | | | | | | | | |
|  |  | | | | | | | | | | | |
|  |  |  |  |  |  |  |  |  |  |  |  |  |
| **Evaluation of Facilitators** | | | | | | | | | | | | |
| 6.How effective was the faculty member in facilitating today's conference? | 1 | | 2 | | 3 | | 4 | | 5 | | Avg: | # of evals |
|  | *Not at all effective* | | *Not so effective* | | *Somewhat effective* | | *Very effective* | | *Extremely effective* | |  |  |
| **Faculty** |  |  |  |  |  | |  | |  | |  |  |
| **Comments** |  |  |  |  |  |  |  |  |  |  |  |  |
|  |  | | | | | | | | | | | |
|  |  |  |  |  |  |  |  |  |  |  |  |  |
|  |  |  |  |  |  |  |  |  |  |  |  |  |
| **Rec Improvements** |  | | | | | | | |  |  |  |  |
|  |  |  |  |  |  |  |  |  |  |  |  |  |
| **Evaluation of Facilitators** | | | | | | | | | | | | |
| 6.How effective was the faculty member in facilitating today's conference? | 1 | | 2 | | 3 | | 4 | | 5 | | Avg: | # of evals |
|  | *Not at all effective* | | *Not so effective* | | *Somewhat effective* | | *Very effective* | | *Extremely effective* | |  |  |
| **Faculty** |  |  |  |  |  | |  | |  | |  |  |
| **Comments** |  |  |  |  |  |  |  |  |  |  |  |  |
|  |  |  |  |  |  |  |  |  |  |  |  |  |
|  |  | | | | | | | | | | | |
|  |  |  |  |  |  |  |  |  |  |  |  |  |
|  |  |  |  |  |  |  |  |  |  |  |  |  |
| **Rec Improvements** |  | | | | |  |  |  |  |  |  |  |
|  |  |  |  |  |  |  |  |  |  |  |  |  |
| **Evaluation of Facilitators** | | | | | | | | | | | | |
| 6.How effective was the faculty member in facilitating today's conference? | 1 | | 2 | | 3 | | 4 | | 5 | | Avg: | # of evals |
|  | *Not at all effective* | | *Not so effective* | | *Somewhat effective* | | *Very effective* | | *Extremely effective* | |  |  |
| **Faculty** |  |  |  |  |  | |  | |  | |  |  |
| **Comments** |  |  |  |  |  |  |  |  |  |  |  |  |
|  |  |  |  |  |  |  |  |  |  |  |  |  |
|  |  | | | | | | | | | | | |
|  |  |  |  |  |  |  |  |  |  |  |  |  |
|  |  |  |  |  |  |  |  |  |  |  |  |  |
| **Rec Improvements** |  | | | | | | | | | | | |
|  |  |  |  |  |  |  |  |  |  |  |  |  |
| **Total Average:** |  |  |  |  |  |  |  |  |  |  |  |  |
