# Supplementary material for: High-Yield, Case-Based, Interactive Workshop on Telehealth and Teleneurology With Pediatric Resident Physicians
Source: MedEdPORTAL. 2023 Aug 25;19:11340. doi: 10.15766/mep_2374-8265.11340 (PMC10450098; doi:10.15766/mep_2374-8265.11340)
Supplement: Supplementary file 1 — Facilitator Guide.docxLearner Guide.docxTeleneurology Cases.pptxTelehealth Introduction.pptxConference Evaluation.docx [file mep_2374-8265.11340-s001.zip › A. Facilitator Guide.docx]

**Appendix A:**

**Telehealth and Teleneurology**

**Facilitator Guide**

## Session Overview:

This is an academic half-day workshop designed to review advantages and disadvantages of telehealth while developing confidence in telehealth adaptation, overcoming challenges, adapting the neurology exam to both age and complaint virtually. This session will be delivered through high-yield neurology cases and small group sessions.

## Session Objectives

By the end of this session, residents will be able to:

1) Identify the advantages and disadvantages of telehealth when working with the pediatric population.

2) Adapt to common obstacles faced during a telehealth visit.

3) Conduct an age-appropriate neurological examination using telehealth technology.

## Session Outline

| **Time** | **Topic** |
| --- | --- |
| 30 min | **Introduction to Telehealth**  Optimizing the Experience  In the Trenches: Adapting to Unexpected Challenges  Discussing the Advantages & Disadvantages of Telehealth |
| 10 min | **Breakout Groups: General Telehealth Examination Cases** |
| 20 min | **Group Discussion** |
| **10 min** | **BREAK** |
| 15 min | **Breakout Groups: Teleneurology Cases 1-4** |
| 45 min | **Group Discussion** |
| 15 min | **Breakout Groups: Teleneurology Cases 5-8** |
| 45 min | **Group Discussion** |
| 5 min | **Wrap-up** |

**Pre-reading for attendees:**

1. Wechsler LR. Advantages and limitations of teleneurology. JAMA Neurol. 2015 Mar;72(3):349-54. doi: 10.1001/jamaneurol.2014.3844. PMID: 25580942.
2. Peredo DE, Hannibal MC. The floppy infant: evaluation of hypotonia. Pediatr Rev. 2009 Sep;30(9):e66-76. doi: 10.1542/pir.30-9-e66. PMID: 19726697.
3. John B. Moeschler, Michael Shevell, COMMITTEE ON GENETICS, John B. Moeschler, Michael Shevell, Robert A. Saul, Emily Chen, Debra L. Freedenberg, Rizwan Hamid, Marilyn C. Jones, Joan M. Stoler, Beth Anne Tarini; Comprehensive Evaluation of the Child With Intellectual Disability or Global Developmental Delays. Pediatrics September 2014; 134 (3): e903–e918. 10.1542/peds.2014-1839. PMID: **25157020.**
4. Grefe A, Hsieh D, Joshi C, Joshi S, Martindale J, et al. Pediatric Neurological Examination via Telemedicine v2. Child Neurology Society. <https://www.childneurologysociety.org/wp-content/uploads/2021/08/Pediatric-Neuro-Exam-via-Telemedicine-Oct2020-v2.pdf>.

**Small Groups: General Exam Cases [10 minutes]**

**Moderator answers are in italics*

For the case listed below answer the following questions within your group: What aspects of the exam can be easily done over telemedicine and what components are difficult? Give one or more examples of adaptations to your physical exam to obtain the information virtually. Be prepared to share your answers with the larger group.

**General Exam Case 1 (Group 1):** A 12 year old healthy girl with episodes of dizziness. She recently had an episode of passing out in homeroom at school. She was noted to be pale and diaphoretic by school nurse. You plan to assess vital signs and assess general appearance and health by telehealth.

- *Utilize the tools patient’s may have. They can check their weight on home scale, temperature with a thermometer, blood pressure if they have a blood pressure cuff at home. Heart rate can be assessed by many smart watches or manually which can be taught to the patient or caregiver. Respiratory rate can be assessed manually with the patient quiet and counting their respirations. Some patients may have a pulse oximeter at home to assess oxygen saturation. Head circumference is challenging but can be attempted with a soft measuring tape.*
- *General exam: the appearance can be assessed, level of comfort or distress, body habitus, nutritional status, observation of any pallor, cyanosis, flushing or icterus.*
- *In the home environment you can also assess safety concerns, barriers to care and fall risks in their surrounding environment if able*

**General Exam Case 2 (Group 2):** An 8 year old boy with history of episodic migraine who has now started having nose bleeds from the right side of his nose with every headache. You plan to assess the HEENT exam by telehealth.

- *Head: can assess shape, relative proportion to body size*
- *Eyes: oculomotility, can they look in all directions? Color of sclera?*
- *Ears: Assess gross hearing, visual ear drainage, swelling behind ear or redness*
- *Nose: Can lift up tip of nose to assess mucous, blood or drainage.*
- *Throat: Older children can open their mouth to allow you to see their tonsils*

**General Exam Case 3 (Group 3)**: A 13 year old girl with headaches on amitriptyline, depression and anxiety presents for shortness of breath. She reports a family history of heart disease. You plan to assess the cardiopulmonary exam by telehealth.

- *Observation of work of breathing, nasal flaring, accessory muscle use, audible wheezing*
- *Skin color: pallor, flushing, diaphoresis, or cyanosis*
- *Any digital clubbing? Any swelling or edema? Will need older patient or caregiver assistance to check*
- *Assess heart rate and respiratory rate as above*

**General Exam Case 4 (Group 4)**: An 8 year old girl with recurrent episodes of emesis. She was referred for evaluation of cyclic vomiting. Y**ou plan to assess the Abdominal Exam by telehealth.**

- ***This is tricky by telehealth and likely will need caregiver involvement. Observe for distention, bulging or engorged veins. Assess for umbilical discoloration, striae or surgical scares. Try to have the patient hold their head up in a laying supine position. Caregiver can palpate with instruction. Watch for wincing, tenderness or rebound tenderness***

**Group Discussion [20 minutes]**

**Review these cases together with the larger group. A representative from each group should bring their discussion to the larger group.**

***Refer to telehealth powerpoint.***

**Small Groups: Neurology Exam Cases [15 minutes]**

Review your assigned neurology with your small group. Take 15 minutes. For your specific exam area listed below answer the following questions: What aspects of the exam can be easily done over telemedicine and what components are difficult? Give one or more examples of adaptations to your physical exam to obtain the information virtually.

**Neurology Case 1 (Group 1):** A 9 month old child is here to see you for a telehealth visit. In conversation, the mother tells you that the child is left handed, just like her father. *Concepts: Deep tendon and infantile reflexes, cerebral palsy*

- - 1. What additional history may you want to know? *Birth history, perinatal risk factors (perinatal depression, prematurity, intraventricular hemorrhage), developmental history, family history of genetic or developmental abnormalities*
    2. Is early handedness normal? *Hand preference before the age of 2 years old may be a sign of weakness*
    3. You plan to assess reflexes by telehealth.
       1. *You can check infantile reflexes. Have learners describe how they would have parents or caregivers complete the following reflexes by telehealth: Moro, root, suck, atonic neck, plantar and palmar grasp, Galant if examiner is comfortable.*
       2. *Deep tendon reflexes are difficult without a trained examiner. You can check Babinski and clonus – have learners describe how they would have parents or caregivers do this. This is less reliable and they may induce a plantar grasp reflex.*
    4. When is grasp reflex considered normal or abnormal? *Grasp reflex is considered* *normal up until 6 months of age. If grasp reflex is persistent or asymmetric, this may be a sign of corticospinal track dysfunction*
    5. Is clonus normal? When is clonus considered abnormal in an infant? *Infants can have several beats of clonus bilaterally up until 2 months of age. Without other signs of upper motor neuron (UMN) dysfunction, this may be considered normal. If clonus is asymmetric, sustained, or there are other signs of UMN dysfunction present such as hyperreflexia, spasticity, or persistent infantile reflexes, further evaluation is required.*
    6. When are upgoing toes considered normal? Abnormal? *There is no clear consensus but typically considered normal until 12 months of age. If persistent beyond 12 months or asymmetric, may be pathologic and further evaluation is required.*
    7. What is on your differential diagnosis? *Structural abnormalities would be highest on the differential including perinatal stroke, intraventricular haemorrhage, periventricular leukomalacia, cortical malformations*
    8. What further work-up would be recommended*? If no imaging has been previously completed such as a head ultrasound in the perinatal period with supporting findings or if the results do not support the exam findings, further imaging is required. A MRI brain without contrast is preferred. Keep in mind children at this age will need to be sedated for a MRI. If the MRI is normal, genetic studies may be warranted for disorders that may mimic cerebral palsy.*

**Neurology Case 2 (Group 2):** A 3 month old infant presents via telehealth for consultation of newly noted hypotonia. The mother feels he has been weaker and more "floppy" over the past few days. She first noticed severe constipation and him unable to lift his head up well. Now he isn’t moving his arms and legs much. On history, more recently they are also noted to have poor feeding, ptosis, facial weakness, and dry mouth. *Concepts: cranial nerves in an infant, hypotonia evaluation, infantile botulism*

1. You plan to assess cranial nerves. *Smell is typically deferred. You can assess visual acuity, extraocular movements, visual fields, facial expressions, facial sensation possible (stroke side of the face can also check rooting for infants), hearing with toy or voice (do they sooth to parent’s voice?). Pupils are more challenging but can be checked with a parent/caregiver using the flashlight. Also can observe for excessive drooling. Can watch them drink a bottle/feed to assess suck, swallow and for dysphagia. Sternocleidomastoid muscles can be assessed by observing head control. Parent/caregiver can pull to sit and allow you to assess head lag (this also is an indicator of tone). If able, try to visualize tongue to assess if midline, appropriate bulk or if fasciculations. Unable to assess fundi/red reflex adequately via telehealth, palate difficult to visualize at this age*
2. What is a good work-up to start for a suddenly floppy baby? *The time course of onset and progression of symptoms are important for the differential considerations. Complete blood count, comprehensive metabolic panel and urine drug screen. Infectious etiologies should be considered such as polio, west nile virus, enterovirus D68, parainfluenza, metapneumovirus, sepsis, etc.* *Acquired etiologies such as acute flaccid myelitis, guillane-barre syndrome or its variants, acute demyelinating encephalomyelitis (ADEM), transverse myelitis (less likely with cranial nerve involvement). Spinal muscular atrophy expected to be more subacute but possible and important for early treatment. A variety of metabolic disorders are also on the differential.*
3. What is highest on your differential? *Note the pattern of weakness (descending) rather than ascending, and time course (acute-subacute, progressive) helps guide the differential. Botulism given the constellation of symptoms with bilateral cranial nerve involvement would be highest on the differential.*
4. You are seeing this patient by telehealth, what should you do next? *These all need to be evaluated urgently. Patient should be sent to the emergency room for further evaluation and treatment*
5. How do you confirm the suspected diagnosis? *Stool samples for C. botulinum (toxin may not been present in stool for up to several months)*

**Neurology Case 3 (Group 3):** A 6 month old boy has had recurrent hiccups then startles and cries afterward. This started when he was 4 months old. They have worsened over time and now happen multiple times/day. His mother has a video of this to show you via telemedicine visit. [review video 1*, video of infantile spasms*] *Concepts: dermatologic exam, neurocutaneous findings, infantile spasms*

1. You plan to assess a dermatologic exam. *Be mindful of their space and privacy (where are they doing the telehealth appointment?). If able have clothing removed from child. Source of light is important. Use flashlight as a tool. Turn camera around to not “selfie” view (better camera). Family can take pictures and upload to patient portal. Can show family examples on internet by sharing your screen to confirm what you are seeing if necessary. Limitations to consider include camera quality, lack of wood’s lamp, challenging lighting, and location/privacy.*
2. Why might this be important in this child? *Neurocutaneous stigmata can help you clue in to a diagnosis. In a child with concerns for infantile spasms, tuberous sclerosis complex would be high on the differential*
3. What is highest on your differential diagnosis? *Infantile spasms*
4. What should you do next? *Urgent referral to neurology and/or send them to the emergency room for evaluation. Talk with the neurology on-call attending*
5. How do you confirm your suspected diagnosis and what is the expected finding? *EEG. It is particularly important to capture sleep (higher yield). Early EEG can be falsely reassuring and may need to be repeated if clinically concerned. Looking for characteristic hypsarrythmia pattern.*
6. Why should this be treated early? *Higher risk of neurodevelopmental sequela if not treated early. Every 2 weeks of delayed treatment leads to a 4 point drop in VABS (Vineland Adaptive Behavior Scale) scores at 4 years*
7. What work-up would you consider? *MRI brain epilepsy protocol, chromosome microarray and karyotype, epilepsy panel genetic testing, and considering metabolic testing*
8. BONUS: What is the triad of West Syndrome? *Infantile spasms, developmental delay,* and *hypsarrythmia*

**Neurology Case 4 (Group 4):** A 5 month old boy is being evaluated for growth failure, severe muscle weakness, delayed motor development, and global hypotonia. He was developing normally until 2 months of age. He is not dysmorphic and has tongue fasciculations. *Concept: tone exam, hypotonia, and spinal muscular atrophy*

- - 1. You plan to assess tone. *You can observe bulk and posture. Is the baby in a flexed curled up position or frog leg position? Are they moving their extremities? Are movements symmetric, left to right and upper to lower? Is there a paucity of movements? Can ask family to flex heels, wiggle arms and legs. Family can also help pull to sit for head lag and truncal tone. Family can touch feet to nose, scarf sign, horizontal and vertical suspension, and assess head/arm position in prone position. Subtle differences are difficult to pick up. Hypertonia may pose more of a problem but can observe for scissoring, stiffness with movements, clenched fists, resistance to ankle flexion.*
    2. What disorder is highest on your differential? *Spinal muscular atrophy given tongue fasciculation, severity of the hypotonia, progression of weakness, and failure to thrive.*
    3. You are seeing this patient by telehealth, what should you do next? *Urgent referral to neurology, call the on-call attending if necessary. Curative treatments are available and the earlier the better!*
    4. How is this diagnosed? *Typically by genetic testing. Spinal muscular atrophy is caused by defects in the survival motor neuron 1 gene (SMN1) on chromosome 5q. This leads to deficits in SMN protein which is important for the function of motor neurons. Blood tests checks the number of copies of SMN1 and SMN2 genes. The number of SMN2 copies helps determine the expected type of SMA, with more copies leading to better motor neuron function and milder types. Some states have this on their standard newborn screen, where others have an opt-in option and others do not offer it. In this case, there is rapid testing through different companies with turnaround times between 2-4 days.*
    5. Is this disorder treatable? What is available? *There are now three different FDA approved treatments available!*
       1. *Spinraza (nusinersen): This is a gene therapy which targets the SMN2 gene to increase protein function. There are both loading and maintenance dose requirements which are given intrathecally. This is available for all ages.*
       2. *Zolegnsma (onasemnogene abeparvovec-xioi): This is a gene therapy which replaces the SMN1 gene using a viral (AAV9) vector. This is a one time dose, approved for use in children under the age of 2.*
       3. *Evrysdi (risdiplam): This is a daily oral medication which is an SMN2-splicing modifier that increases the production of the SMN protein. This is used for all ages and has weight-based dosing.*

**Group Discussion [45 minutes]**

**Review these cases together with the larger group. A representative from each group should bring their discussion to the larger group.**

***Refer to Teleneurology PowerPoint for case review and teaching points***

**Small Groups: Neurology Exam Cases [15 minutes]**

Return to your small group. Review your assigned neurology with your small group. Take 15 minutes. For your specific exam area listed below answer the following questions: What aspects of the exam can be easily done over telemedicine and what components are difficult? Give one or more examples of adaptations to your physical exam to obtain the information virtually.

**Neurology Case 5 (Group 1):** A 5-year-old boy has progressive muscular weakness, has been falling frequently and has increasing difficulty with climbing stairs, running, jumping and rising from a squatting position. He sat at 10 months and walked at 18 months. The maternal uncle has a disorder that began similarly and has required him to use a wheelchair since his early teens. *Concepts: motor and strength exam, muscular dystrophy*

1. You plan to assess a motor/strength exam. *Assess bulk with visual inspection. Look at posture, calves, feet arches, and for scoliosis. Strength can be tailored to age. A 5 year old should be able to do most of a normal neurological exam. Can do antigravity testing in all 4 extremities (hold up for 10 sec, assess for drift), pronator drift, orbiting, scapular winging. Functionally, can assess standing, hop on each leg, jump up and down, heel walking, and toe walking. Can have them do squats and push ups if able. Get up off the floor (gowers) or the chair without using arms. Fine motor – can ask to draw, write, pick up something small, button/zippers. Myotonia – can ask to grip family member’s hand and let go (assess for getting stuck). Formal strength testing is limited but if they can do all these things they are probably pretty strong. Very athletic people may be difficult to pick up subtle findings/weakness.*
2. What is highest on your differential? *Family history, progressive nature, and history lead to concern for a muscular dystrophy, particularly Duchenne Muscular Dystrophy (DMD) at this age. Remember “Becker’s (muscular dystrophy) do better”*
3. What initial test can you order to help support your diagnosis? *Creatine Kinase (CK)! Cheap and relatively easy to get.*
4. BONUS: What are the expected findings? *CK can be 10-100x normal in muscular dystrophies*
5. How is DMD inherited? *X linked (dystrophin gene)*

**Neurology Case 6 (Group 2):** A mother mentions in a telehealth visit that her 6-year-old child’s academic performance has declined during the last year. The teacher notices the child staring frequently throughout the day. Sometimes, he seems “off in her own world” and does not respond to questions. *Concepts: Mental status and behavior exam, staring spells, and absence seizures*

1. You plan to assess mental status/behavior. *Observation is important. You can assess their level of alertness, interaction with the environment/caregiver. Play games to engage reciprocal play (which also gives you clues to social interactions). Attention – are they able to attend, sit still? Do they require repetition/reminders? Fund of knowledge (age appropriate) – body parts, colors, name, people in the room, animal sounds for younger children. Is there speech content and fluency normal? What is their affect like (are they shy, anxious, withdrawn)? Mood – you can ask. Behavior – do they pick up on social cues, are they impulsive, etc. Seeing them in their home environment may be helpful – it can also have more distractions.*
2. What is highest on your differential? *Absence seizures, particularly childhood absence epilepsy at this age*
3. What else is on your differential diagnosis for staring spells? *Attention-deficit hyperactivity disorder (ADHD), obstructive sleep apnea (OSA) or poor sleep pattern/quality, learning disability, developmental delay, or behavioral. Frequency and duration of staring spells can be helpful. If there is situational context that can also provide historical clues. Absence seizures occur hundreds of time per day and often interrupt activities (ask about behavioral arrest – ie, do they ever do it in the middle of a task such as eating, talking, walking where they stop what they are doing and stare?). Less frequent but longer staring spells could also represent atypical absence or focal seizures. Assess for automatisms, motor symptoms or incontinence with spells.*
4. What test can you do during your visit that may help establish a diagnosis*? Hyperventilation – ask parent to get a tissue. Explain to the child to blow the tissue repetitively, slow and steady for at least 2 minutes with good effort. Enlist the parent or caregiver’s help. Tell the parent what you are doing and what may happen.*
5. What test would you order and what is the expected finding? *EEG, 3Hz spike and slow wave*
6. What is the treatment of choice? *ethosuxamide*

**Neurology Case 7 (Group 3):** 3: A 13-year-old girl with history of scoliosis presents for consultation of worsening unsteadiness and increased falls. Family notes her speech has changed over the last year but thought this was from her braces. *Concepts: coordination and gait exams, ataxia, Friedreich’s ataxia*

1. You plan to assess coordination and gait*. You can assess movements – is the speed appropriate? Is there too little, too much or extra movements? You can check finger to nose (demonstrate, point to examiners nose on screen or family members nose, or touch a toy/object). Finger tapping or hand flicking symmetry and rhythm. Heel to shin. Truncal stability – sitting steady? Require support? Swaying? Have fingers outstretched to look for tremor. Can have them hold fingers close together. Have them draw a spiral with both hands or write a sentence. Have them pour cups outside or over a sink (watch for spilling). Can they button or zipper (other fine motor tasks). Assess Romberg (if concerned about steadiness make sure a family member is there or they are safe from falls). Casual gait/stance, heel and toe walking, tandem. Can they run? Can you see them go up/down the stairs?*
2. You are concerned she has ataxia. What is the most common hereditary ataxia in childhood? *Friedreich’s ataxia*
3. How is this inherited? *Autosomal recessive (family history may be negative)*
4. BONUS: What type of genetic mutation? *trinucleotide repeat*
5. What systemic findings can be seen with this disorder? *Scoliosis, diabetes, cardiomyopathy. Hearing and vision loss are late findings*

**Neurology Case 8 (Group 4):** A 16 year old girl with history of polycystic ovarian syndrome on an oral contraceptive and episodic headaches is returning for evaluation of worsening headaches via telehealth. Previously occurring 1-2 times per month, now happening constantly over the last 2 weeks. No photophobia, phonophobia, nausea or vomiting. Headaches are worse in the morning. Today, she does report some double vision. She does endorse tinnitus. *Concepts: cranial nerve exam in an adolescent, increased intracranial pressure, idiopathic intracranial hypertension*

1. You plan to cranial nerves. *At this age, most of the CN exam can be completed virtually. Smell is typically deferred but possible if concerned (get coffee grounds, identify with eyes closed). Visual acuity – have them close one eye, what do they see (then the other eye). You can hold a picture up, what do they see? Visual fields – use family member to bring something into the periphery in all four quadrants. Pupils – have them get close to the camera (close their eyes tight, then open, look for response) or have family use flashlight and see if pupils constrict. Fundi limited. Facial expressions. Facial sensation – have them or a family member touch face in dermatomal distributions, does it feel symmetric? Can get an ice cube for cold if necessary. Hearing – intact to voice? Have family finger rub or finger click on each side. Assess speech fluency, guttural sounds, content. Palate – can be difficult to see, have them get close and say ahh – is the uvula midline? Palate elevation? Palate abnormalities? SCM – shoulder shrug. Have them stick their tongue out, observe midline/bulk/deviation/fasciculations. Use light if necessary*. *Unable to assess fundi adequately via telehealth*
2. What is highest on your differential diagnosis? *Increased intracranial pressure. Differential is broad but can include idiopathic intracranial hypertension (IIH), tumor, cerebral venous thrombosis*
3. What do you expect to see on your cranial nerve exam? *6^th^ nerve palsy can be associated with IIH*
4. You are seeing this patient by telehealth, what should you do next? *Bring them in urgently to check fundi, refer to neuro urgently (Call) or refer to ER*
5. What type of imaging does this patient need? *MRI brain and MR venogram*
6. What is the treatment of choice for IIH? *Acetazolamide and 10% weight loss.*

**Group Discussion [45 minutes]**

**Review these cases together with the larger group. A representative from each group should bring their discussion to the larger group.**

***Refer to Teleneurology PowerPoint for case review and teaching points***

**Wrap up [5 minutes]**

***Refer to Teleneurology PowerPoint for summary points***
