# Supplementary material for: High-Yield, Case-Based, Interactive Workshop on Telehealth and Teleneurology With Pediatric Resident Physicians
Source: MedEdPORTAL. 2023 Aug 25;19:11340. doi: 10.15766/mep_2374-8265.11340 (PMC10450098; doi:10.15766/mep_2374-8265.11340)
Supplement: Supplementary file 1 — Facilitator Guide.docxLearner Guide.docxTeleneurology Cases.pptxTelehealth Introduction.pptxConference Evaluation.docx [file mep_2374-8265.11340-s001.zip › B. Learner Guide.docx]

**Appendix B:**

**Telehealth and Teleneurology**

**Learner Guide**

## Session Overview:

This is an academic half-day workshop designed to review advantages and disadvantages of telehealth while developing confidence in telehealth adaptation, overcoming challenges, adapting the neurology exam to both age and complaint virtually. This session will be delivered through high-yield neurology cases and small group sessions.

## Session Objectives

By the end of this session, residents will be able to:

1) Identify the advantages and disadvantages of telehealth when working with the pediatric population.

2) Adapt to common obstacles faced during a telehealth visit.

3) Conduct an age-appropriate neurological examination using telehealth technology.

## Session Outline

| **Time** | **Topic** |
| --- | --- |
| 30 min | **Introduction to Telehealth**  Optimizing the Experience  In the Trenches: Adapting to Unexpected Challenges  Discussing the Advantages & Disadvantages of Telehealth |
| 10 min | **Breakout Groups: General Telehealth Examination Cases** |
| 20 min | **Group Discussion** |
| **10 min** | **BREAK** |
| 15 min | **Breakout Groups: Teleneurology Cases 1-4** |
| 45 min | **Group Discussion** |
| 15 min | **Breakout Groups: Teleneurology Cases 5-8** |
| 45 min | **Group Discussion** |
| 5 min | **Wrap-up** |

**Pre-reading for attendees:**

1. Wechsler LR. Advantages and limitations of teleneurology. JAMA Neurol. 2015 Mar;72(3):349-54. doi: 10.1001/jamaneurol.2014.3844. PMID: 25580942.
2. Peredo DE, Hannibal MC. The floppy infant: evaluation of hypotonia. Pediatr Rev. 2009 Sep;30(9):e66-76. doi: 10.1542/pir.30-9-e66. PMID: 19726697.
3. John B. Moeschler, Michael Shevell, COMMITTEE ON GENETICS, John B. Moeschler, Michael Shevell, Robert A. Saul, Emily Chen, Debra L. Freedenberg, Rizwan Hamid, Marilyn C. Jones, Joan M. Stoler, Beth Anne Tarini; Comprehensive Evaluation of the Child With Intellectual Disability or Global Developmental Delays. Pediatrics September 2014; 134 (3): e903–e918. 10.1542/peds.2014-1839
4. Grefe A, Hsieh D, Joshi C, Joshi S, Martindale J, et al. Pediatric Neurological Examination via Telemedicine v2. Child Neurology Society. <https://www.childneurologysociety.org/wp-content/uploads/2021/08/Pediatric-Neuro-Exam-via-Telemedicine-Oct2020-v2.pdf>.

**Small Groups: General Exam Cases [10 minutes]**

For the case listed below answer the following questions within your group: What aspects of the exam can be easily done over telemedicine and what components are difficult? Give one or more examples of adaptations to your physical exam to obtain the information virtually. Be prepared to share your answers with the larger group.

**General Exam Case 1 (Group 1):** A 12 year old healthy girl with episodes of dizziness. She recently had an episode of passing out in homeroom at school. She was noted to be pale and diaphoretic by school nurse. You plan to assess vital signs and assess general appearance and health by telehealth.

**General Exam Case 2 (Group 2):** An 8 year old boy with history of episodic migraine who has now started having nose bleeds from the right side of his nose with every headache. You plan to assess the HEENT exam by telehealth.

**General Exam Case 3 (Group 3)**: A 13 year old girl with headaches on amitriptyline, depression and anxiety presents for shortness of breath. She reports a family history of heart disease. You plan to assess the cardiopulmonary exam by telehealth.

**General Exam Case 4 (Group 4)**: An 8 year old girl with recurrent episodes of emesis. She was referred for evaluation of cyclic vomiting. Y**ou plan to assess the Abdominal Exam by telehealth.**

**Group Discussion [20 minutes]**

**Review these cases together with the larger group. A representative from each group should bring their discussion to the larger group.**

**Small Groups: Neurology Exam Cases [15 minutes]**

Review your assigned neurology with your small group. Take 15 minutes. For your specific exam area listed below answer the following questions: What aspects of the exam can be easily done over telemedicine and what components are difficult? Give one or more examples of adaptations to your physical exam to obtain the information virtually.

**Neurology Case 1 (Group 1):** A 9 month old child is here to see you for a telehealth visit. In conversation, the mother tells you that the child is left handed, just like her father.

- - 1. What additional history may you want to know?
    2. Is early handedness normal?
    3. You plan to assess reflexes by telehealth. Discuss with your group.
    4. When is grasp reflex considered normal or abnormal?
    5. Is clonus normal? When is clonus considered abnormal in an infant?
    6. When are upgoing toes considered normal? Abnormal?
    7. What is on your differential diagnosis?
    8. What further work-up would be recommended*?*

**Neurology Case 2 (Group 2):** A 3 month old infant presents via telehealth for consultation of newly noted hypotonia. The mother feels he has been weaker and more "floppy" over the past few days. She first noticed severe constipation and him unable to lift his head up well. Now he isn’t moving his arms and legs much. On history, more recently they are also noted to have poor feeding, ptosis, facial weakness, and dry mouth.

1. You plan to assess cranial nerves. Discuss with your group
2. What is a good work-up to start for a suddenly floppy baby?
3. What is highest on your differential?
4. You are seeing this patient by telehealth, what should you do next?
5. How do you confirm the suspected diagnosis?

**Neurology Case 3 (Group 3):** A 6 month old boy has had recurrent hiccups then startles and cries afterward. This started when he was 4 months old. They have worsened over time and now happen multiple times/day. His mother has a video of this to show you via telemedicine visit. [review video 1]

1. You plan to assess a dermatologic exam.
2. Why might this be important in this child?
3. What is highest on your differential diagnosis?
4. What should you do next?
5. How do you confirm your suspected diagnosis and what is the expected finding?
6. Why should this be treated early?
7. What work-up would you consider?
8. BONUS: What is the triad of West Syndrome?

**Neurology Case 4 (Group 4):** A 5 month old boy is being evaluated for growth failure, severe muscle weakness, delayed motor development, and global hypotonia. He was developing normally until 2 months of age. He is not dysmorphic and has tongue fasciculations.

- - 1. You plan to assess tone.
    2. What disorder is highest on your differential?
    3. How is this diagnosed?*.*
    4. Is this disorder treatable? What is available?

**Group Discussion [45 minutes]**

**Review these cases together with the larger group. A representative from each group should bring their discussion to the larger group.**

***Refer to Teleneurology PowerPoint for case review and teaching points***

**Small Groups: Neurology Exam Cases [15 minutes]**

Return to your small group. Review your assigned neurology with your small group. Take 15 minutes. For your specific exam area listed below answer the following questions: What aspects of the exam can be easily done over telemedicine and what components are difficult? Give one or more examples of adaptations to your physical exam to obtain the information virtually.

**Neurology Case 5 (Group 1):** A 5-year-old boy has progressive muscular weakness, has been falling frequently and has increasing difficulty with climbing stairs, running, jumping and rising from a squatting position. He sat at 10 months and walked at 18 months. The maternal uncle has a disorder that began similarly and has required him to use a wheelchair since his early teens.

1. You plan to assess a motor/strength exam.
2. What is highest on your differential?
3. What initial test can you order to help support your diagnosis?
4. BONUS: What are the expected findings?
5. How is this disorder inherited?

**Neurology Case 6 (Group 2):** A mother mentions in a telehealth visit that her 6-year-old child’s academic performance has declined during the last year. The teacher notices the child staring frequently throughout the day. Sometimes, he seems “off in her own world” and does not respond to questions.

1. You plan to assess mental status/behavior.
2. What is highest on your differential?
3. What else is on your differential diagnosis for staring spells?
4. What test can you do during your visit that may help establish a diagnosis*?*
5. What test would you order and what is the expected finding?
6. What is the treatment of choice?

**Neurology Case 7 (Group 3):** 3: A 13-year-old girl with history of scoliosis presents for consultation of worsening unsteadiness and increased falls. Family notes her speech has changed over the last year but thought this was from her braces.

1. You plan to assess coordination and gait*.*
2. You are concerned she has ataxia. What is the most common hereditary ataxia in childhood?
3. How is this inherited?
4. BONUS: What type of genetic mutation?
5. What systemic findings can be seen with this disorder?

**Neurology Case 8 (Group 4):** A 16 year old girl with history of polycystic ovarian syndrome on an oral contraceptive and episodic headaches is returning for evaluation of worsening headaches via telehealth. Previously occurring 1-2 times per month, now happening constantly over the last 2 weeks. No photophobia, phonophobia, nausea or vomiting. Headaches are worse in the morning. Today, she does report some double vision. She does endorse tinnitus.

1. You plan to test cranial nerves.
2. What is highest on your differential diagnosis?
3. What do you expect to see on your cranial nerve exam?
4. You are seeing this patient by telehealth, what should you do next?
5. What type of imaging does this patient need?
6. What is the treatment of choice for this disorder?

**Group Discussion [45 minutes]**

**Review these cases together with the larger group. A representative from each group should bring their discussion to the larger group.**
