# Supplementary material for: High-Yield, Case-Based, Interactive Workshop on Telehealth and Teleneurology With Pediatric Resident Physicians
Source: MedEdPORTAL. 2023 Aug 25;19:11340. doi: 10.15766/mep_2374-8265.11340 (PMC10450098; doi:10.15766/mep_2374-8265.11340)
Supplement: Supplementary file 1 — Facilitator Guide.docxLearner Guide.docxTeleneurology Cases.pptxTelehealth Introduction.pptxConference Evaluation.docx [file mep_2374-8265.11340-s001.zip › C. Teleneurology Cases.pptx]

## Slide 1
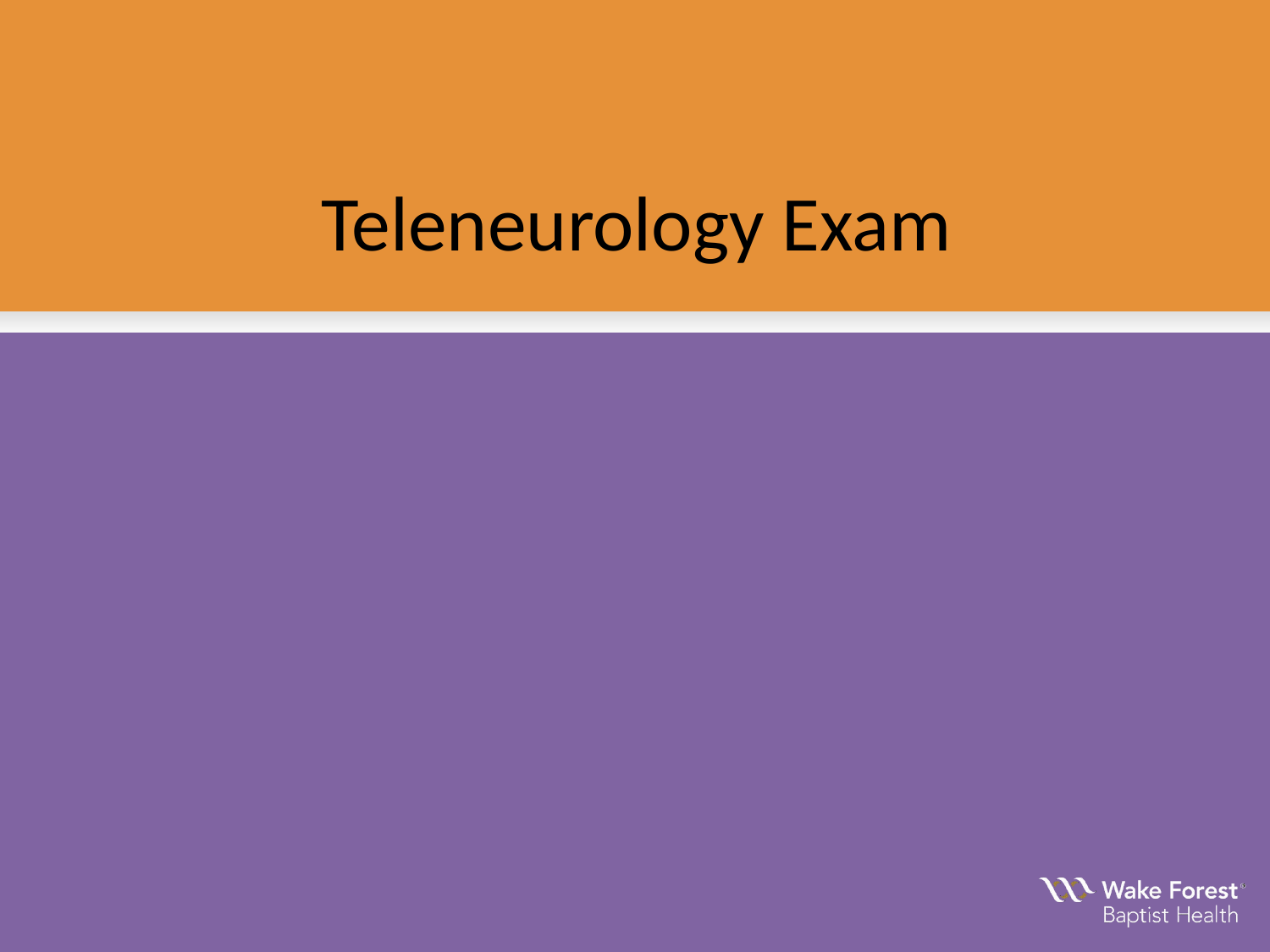

# Teleneurology Exam

## Slide 2
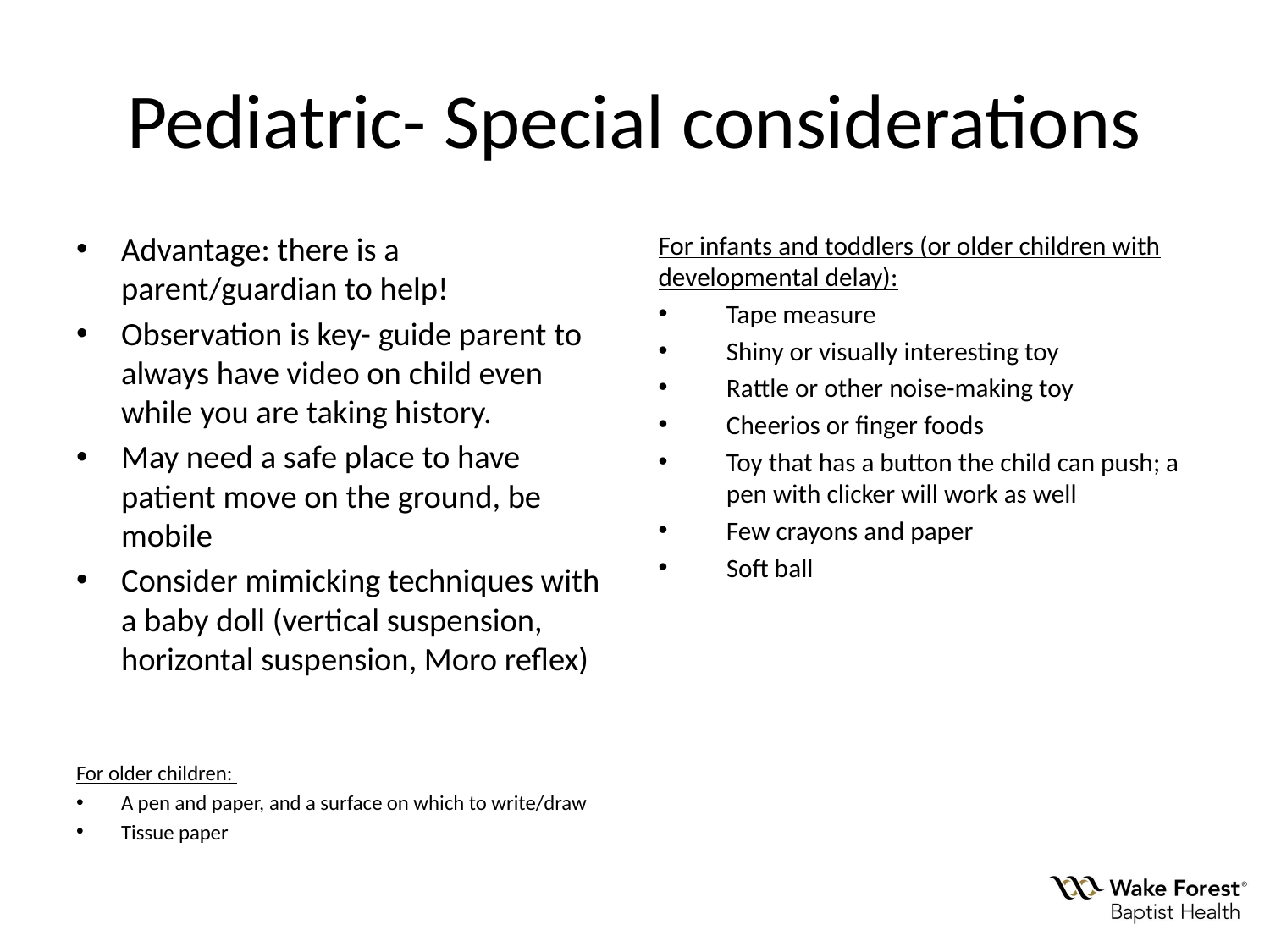

# Pediatric- Special considerations
Advantage: there is a parent/guardian to help!
Observation is key- guide parent to always have video on child even while you are taking history.
May need a safe place to have patient move on the ground, be mobile
Consider mimicking techniques with a baby doll (vertical suspension, horizontal suspension, Moro reflex)
For older children:
A pen and paper, and a surface on which to write/draw
Tissue paper
For infants and toddlers (or older children with developmental delay):
Tape measure
Shiny or visually interesting toy
Rattle or other noise-making toy
Cheerios or finger foods
Toy that has a button the child can push; a pen with clicker will work as well
Few crayons and paper
Soft ball
2

## Slide 3
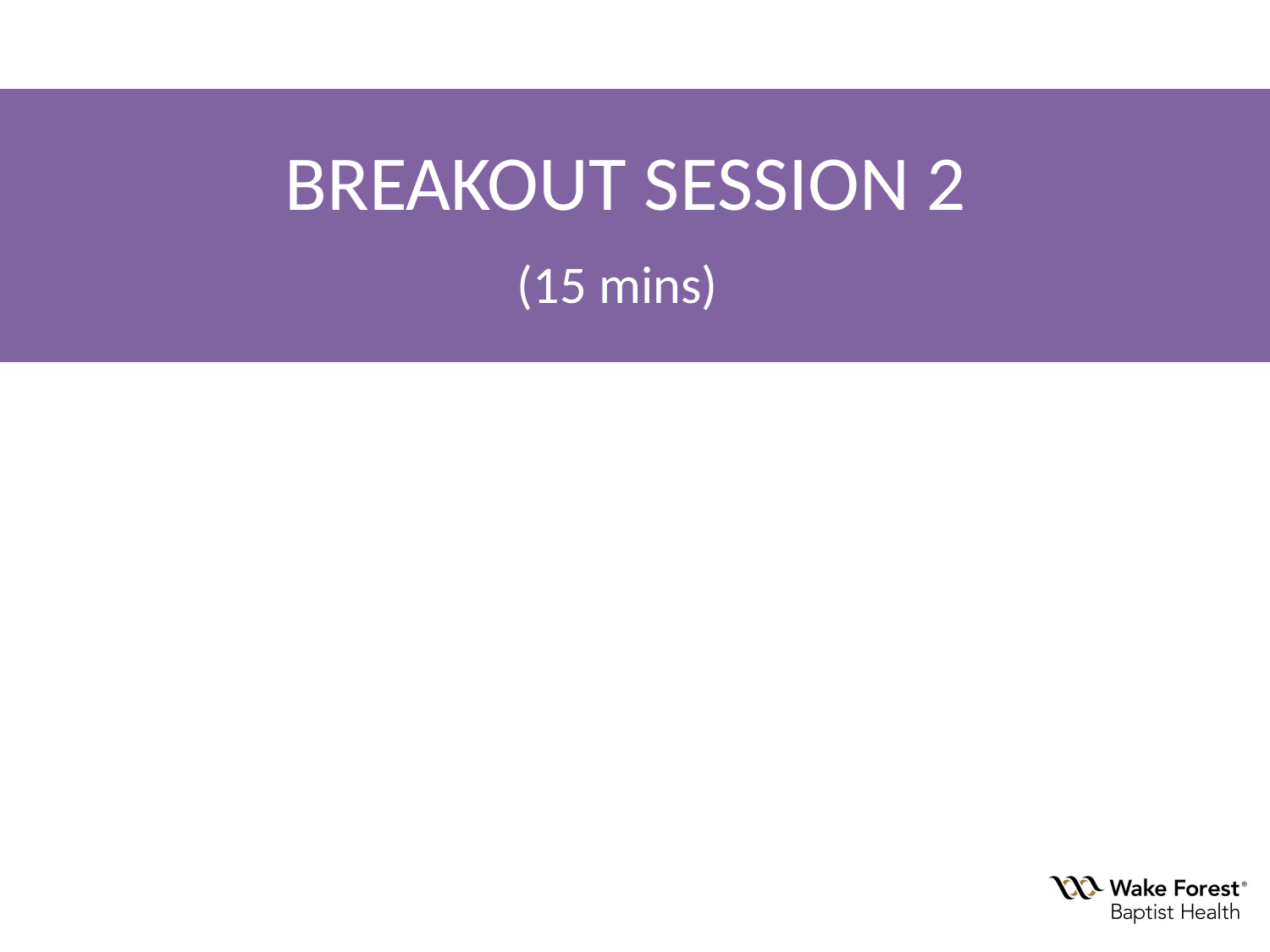

# BREAKOUT SESSION 2 (15 mins)

## Slide 4
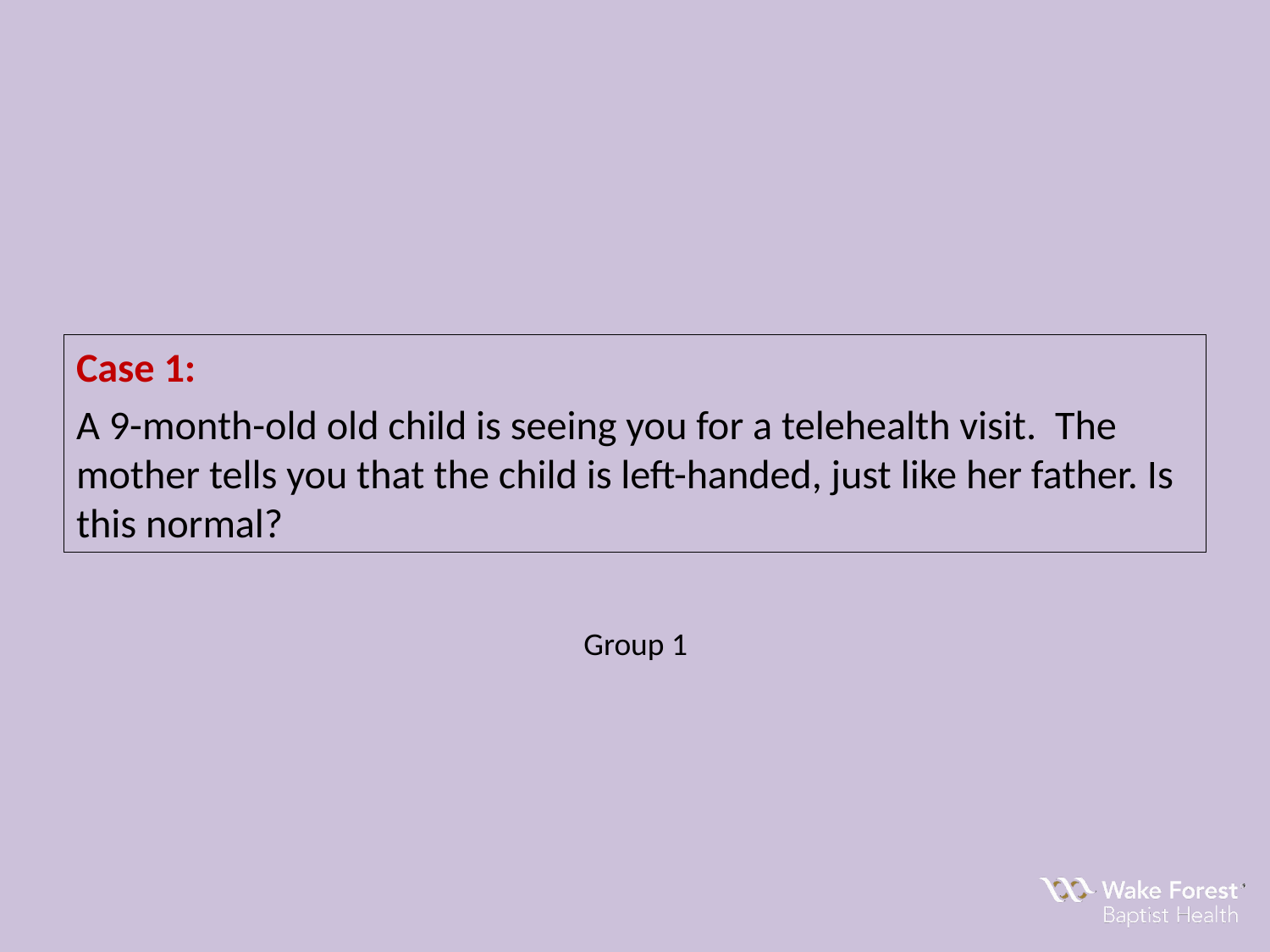

Case 1:
A 9-month-old old child is seeing you for a telehealth visit. The mother tells you that the child is left-handed, just like her father. Is this normal?
Group 1

## Slide 5
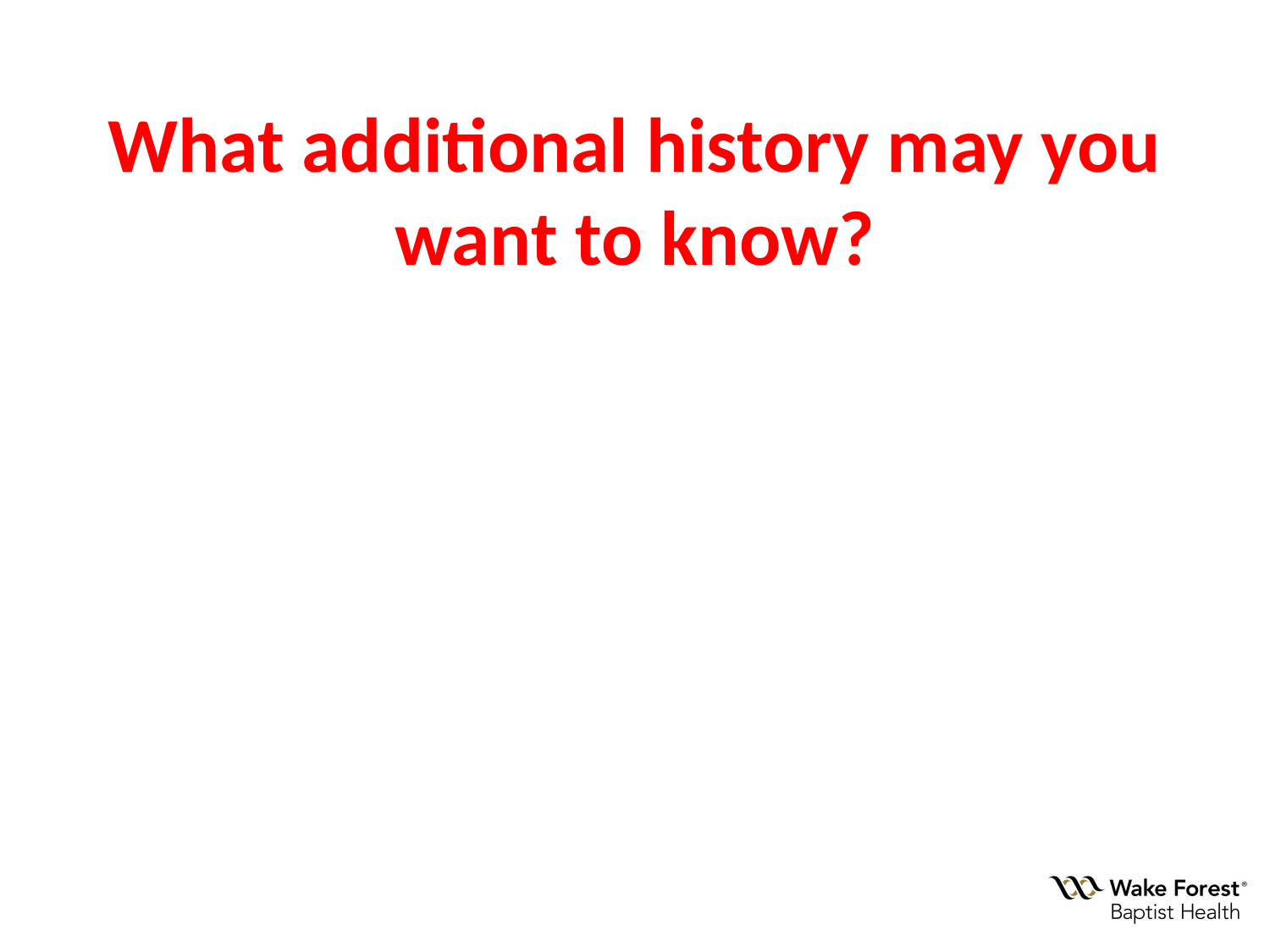

# What additional history may you want to know?

## Slide 6
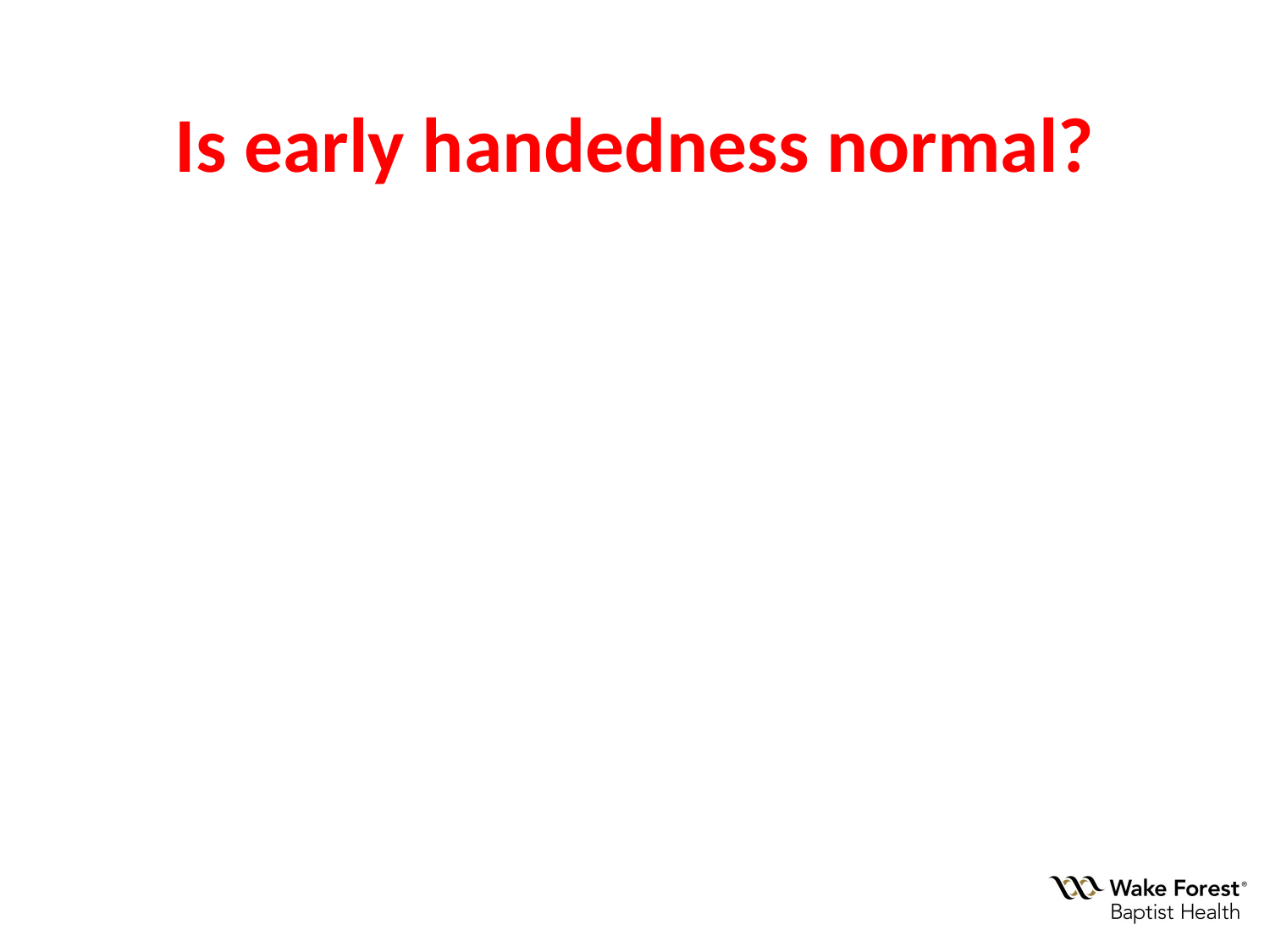

# Is early handedness normal?

## Slide 7
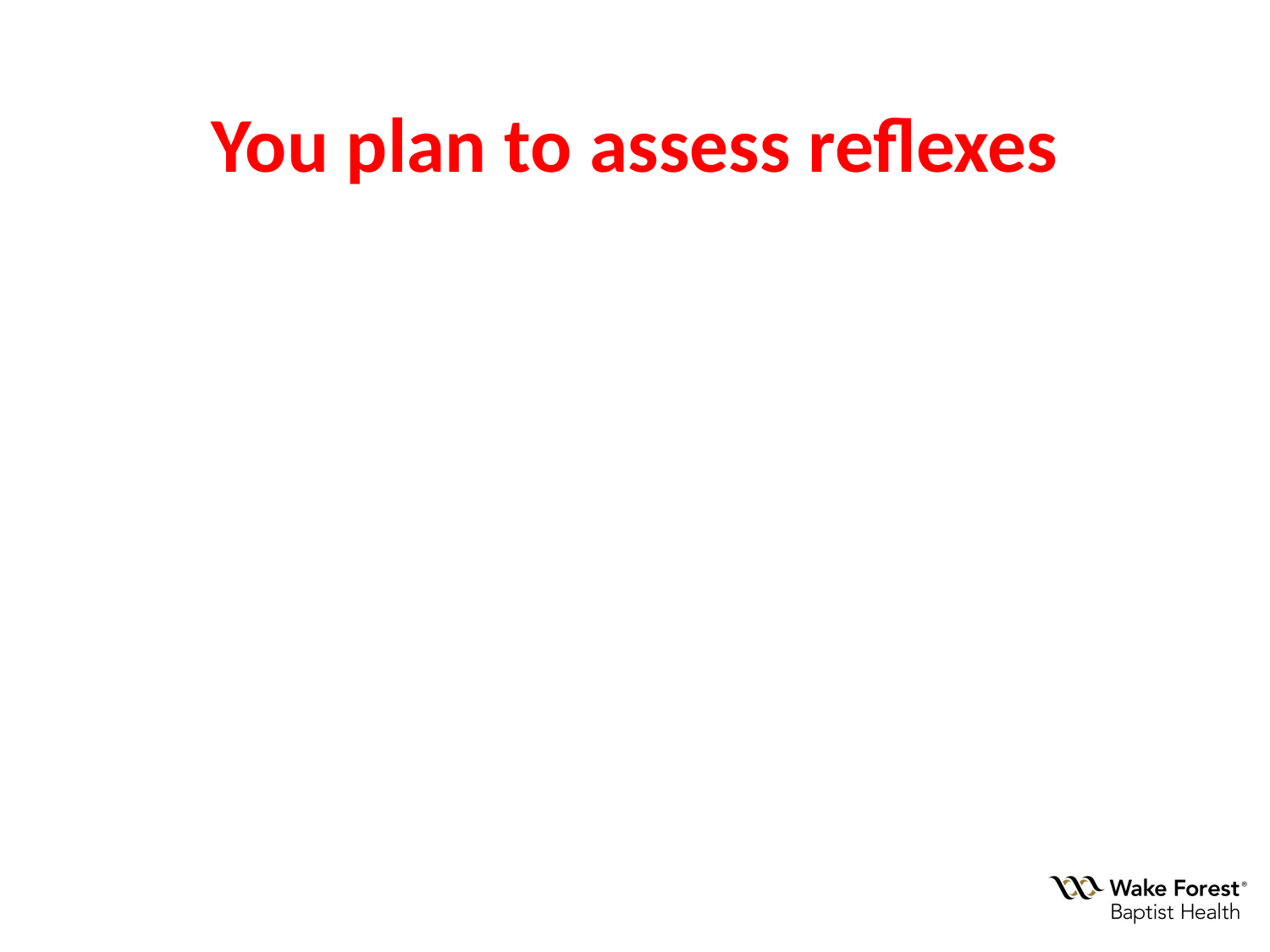

# You plan to assess reflexes

## Slide 8
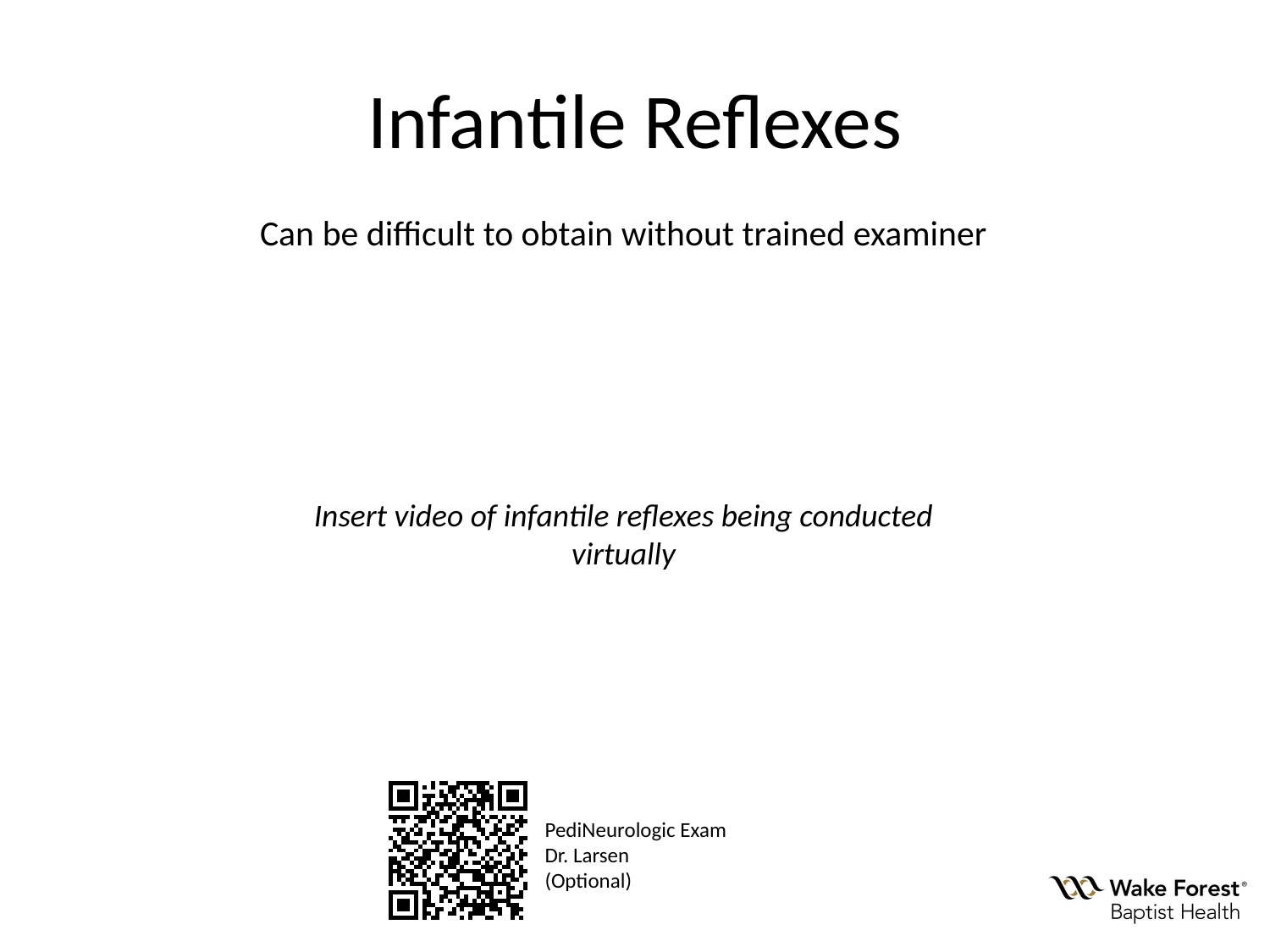

# Infantile Reflexes
Can be difficult to obtain without trained examiner
Insert video of infantile reflexes being conducted virtually
PediNeurologic Exam Dr. Larsen
(Optional)

## Slide 9
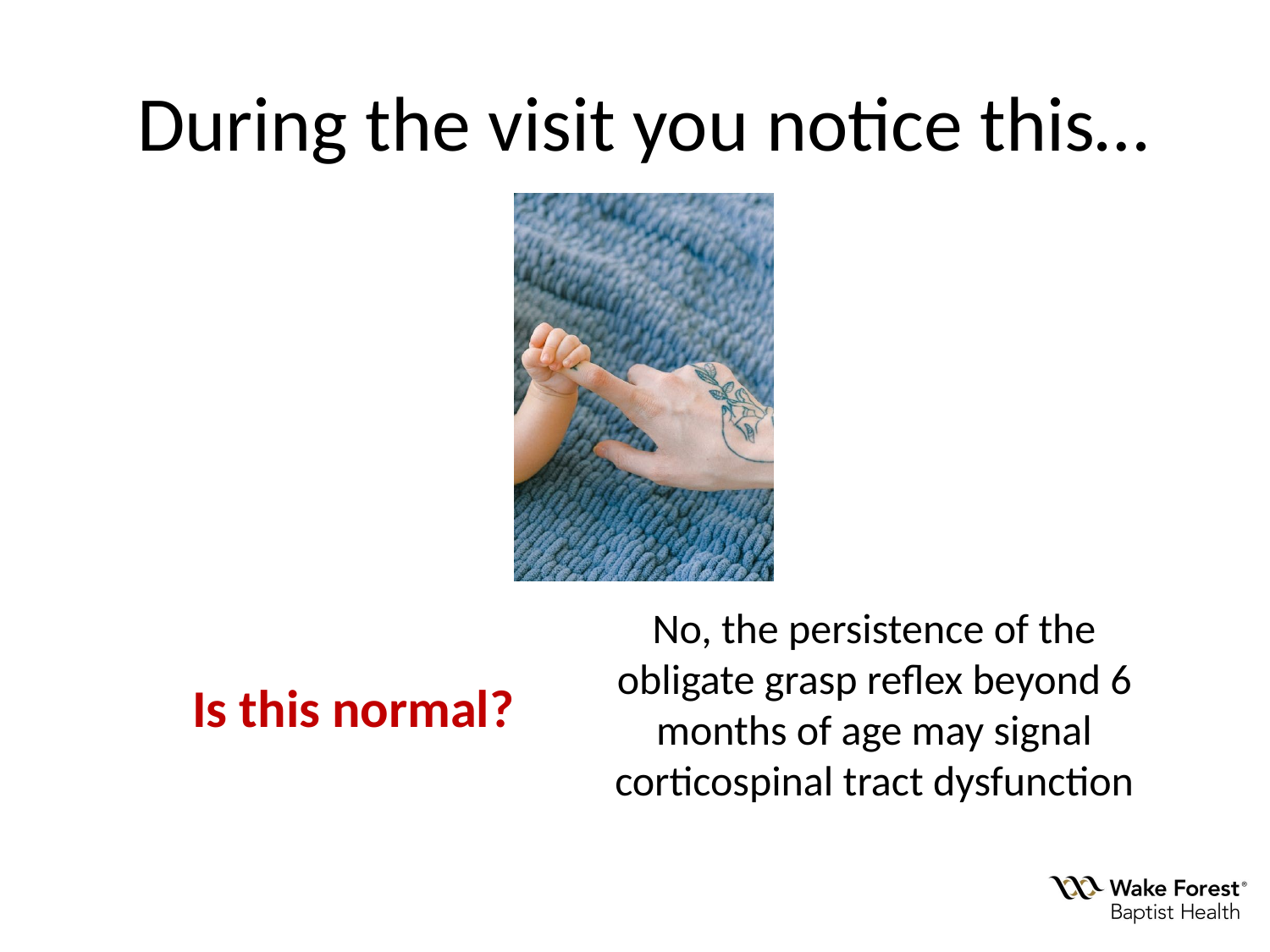

# During the visit you notice this…
No, the persistence of the obligate grasp reflex beyond 6 months of age may signal corticospinal tract dysfunction
Is this normal?

## Slide 10
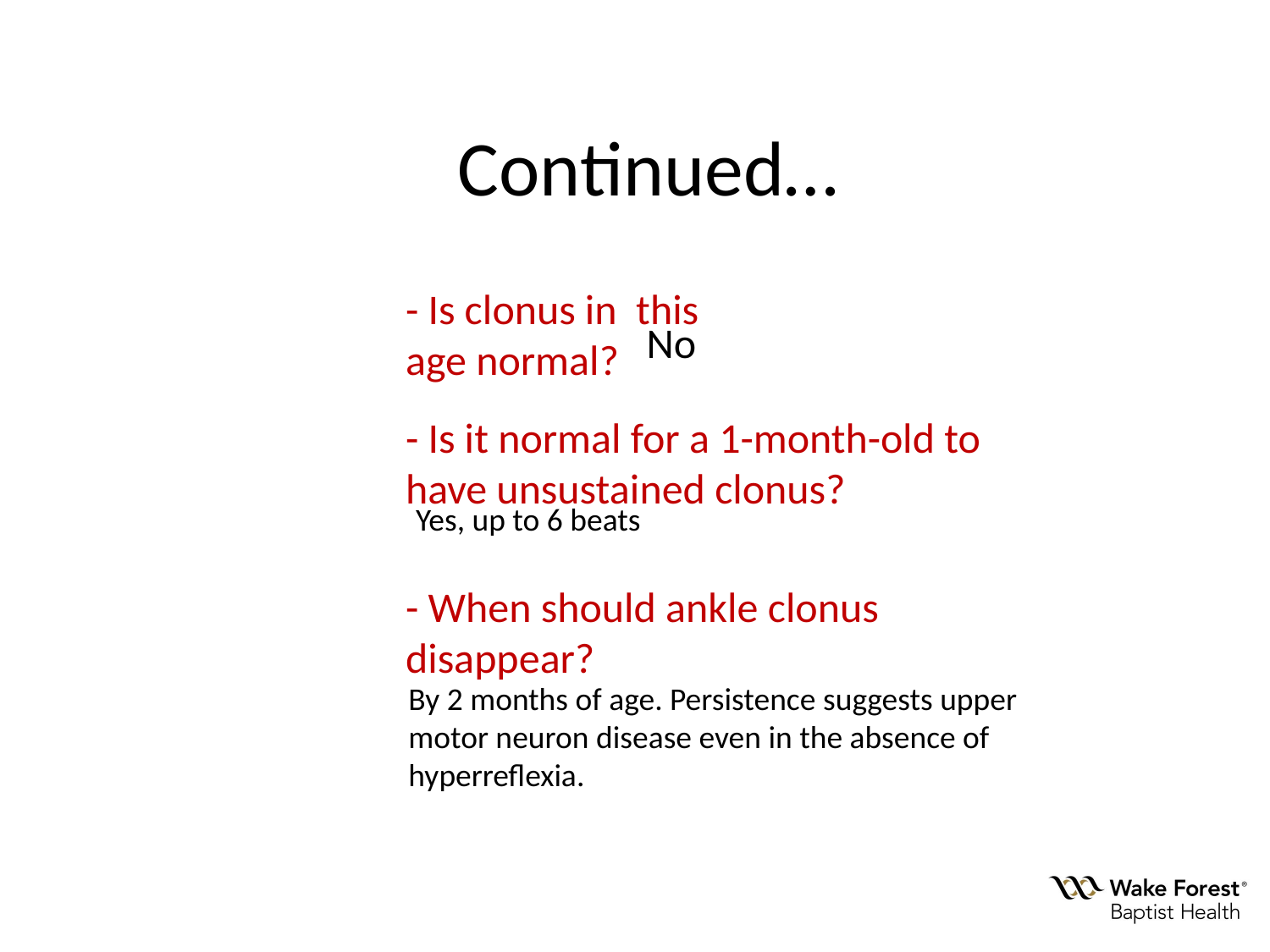

# Continued…
- Is clonus in this age normal?
No
- Is it normal for a 1-month-old to have unsustained clonus?
Yes, up to 6 beats
- When should ankle clonus disappear?
By 2 months of age. Persistence suggests upper motor neuron disease even in the absence of hyperreflexia.

## Slide 11
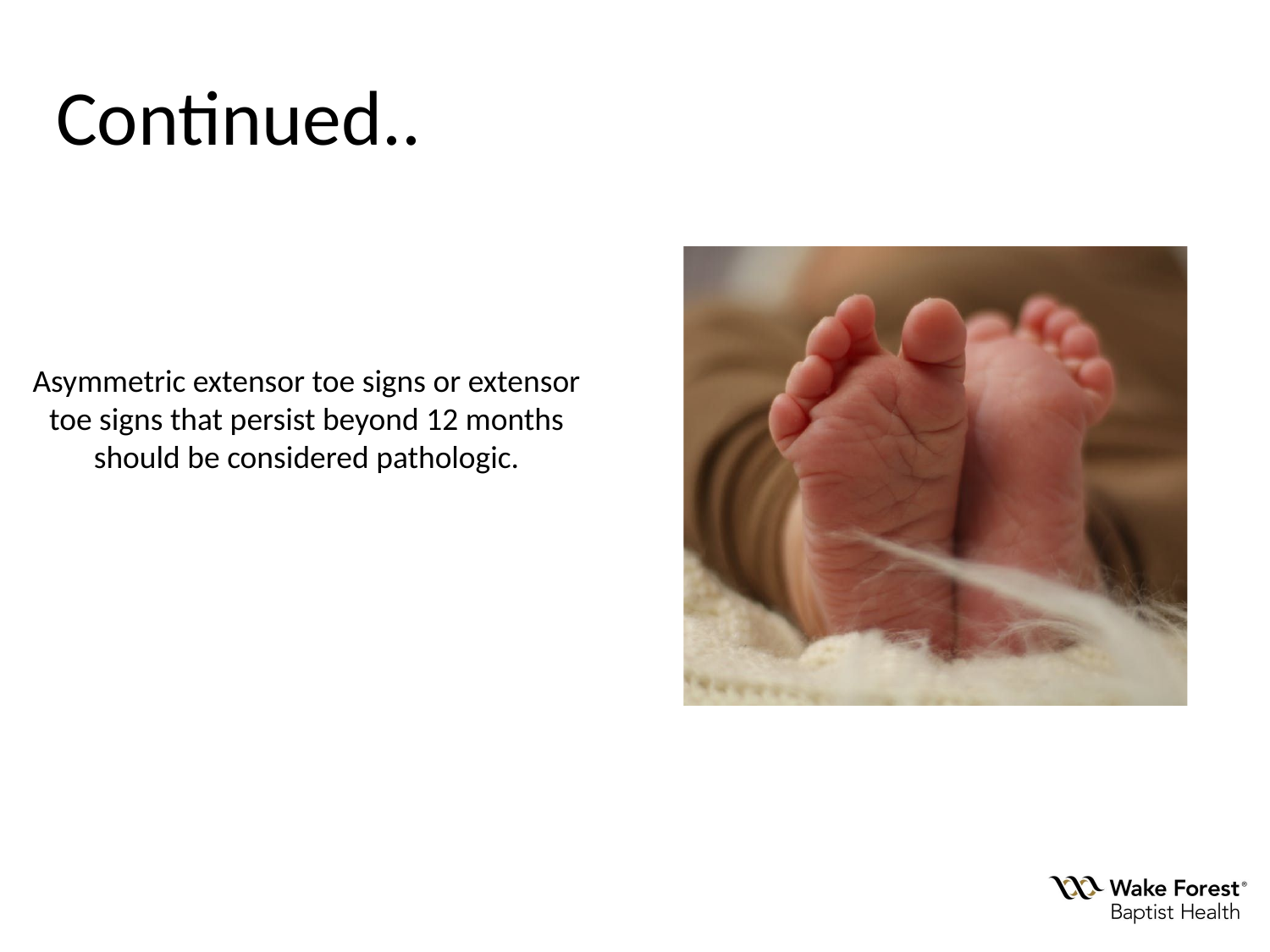

# Continued..
Asymmetric extensor toe signs or extensor toe signs that persist beyond 12 months should be considered pathologic.

## Slide 12
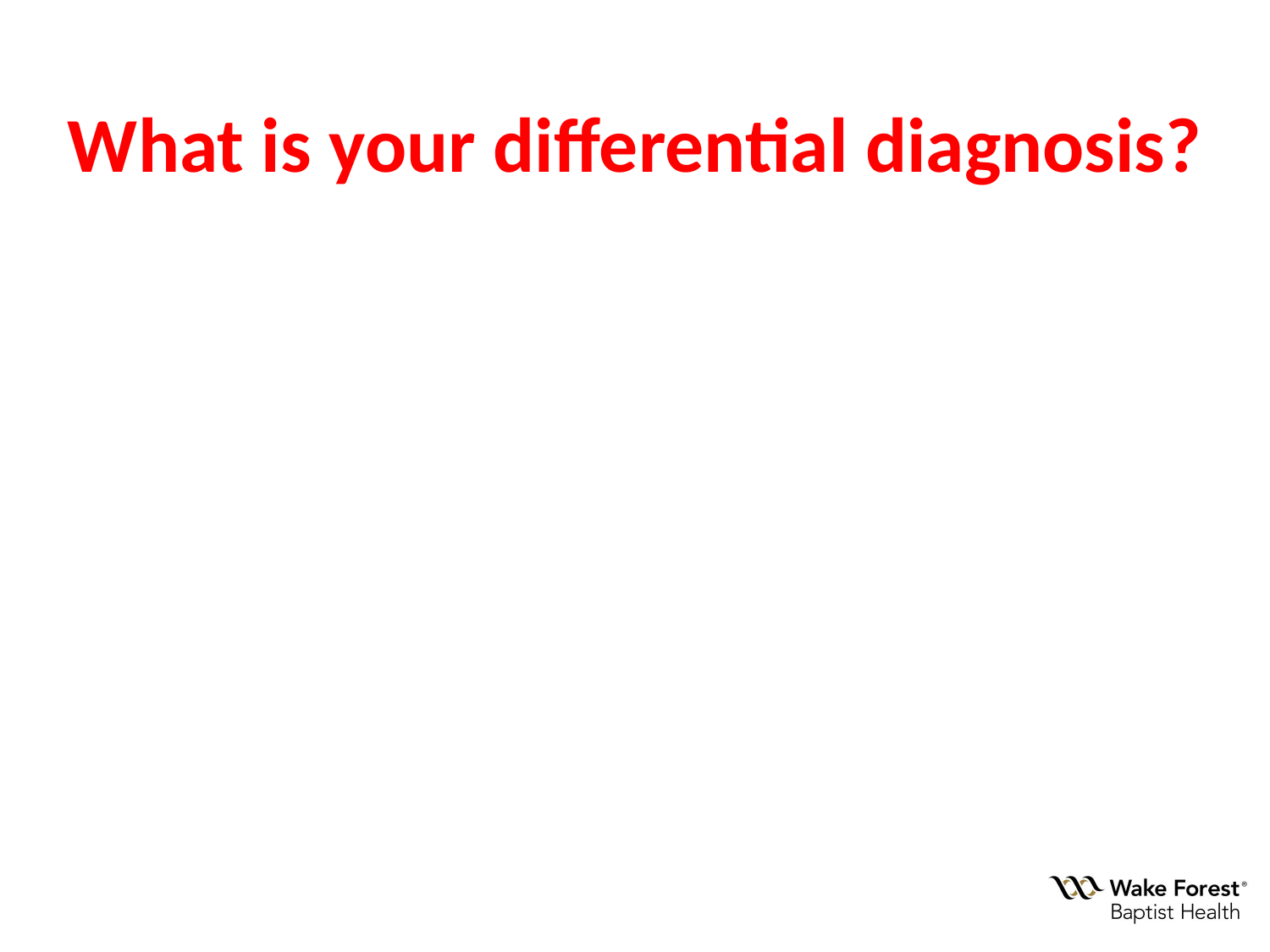

# What is your differential diagnosis?

## Slide 13
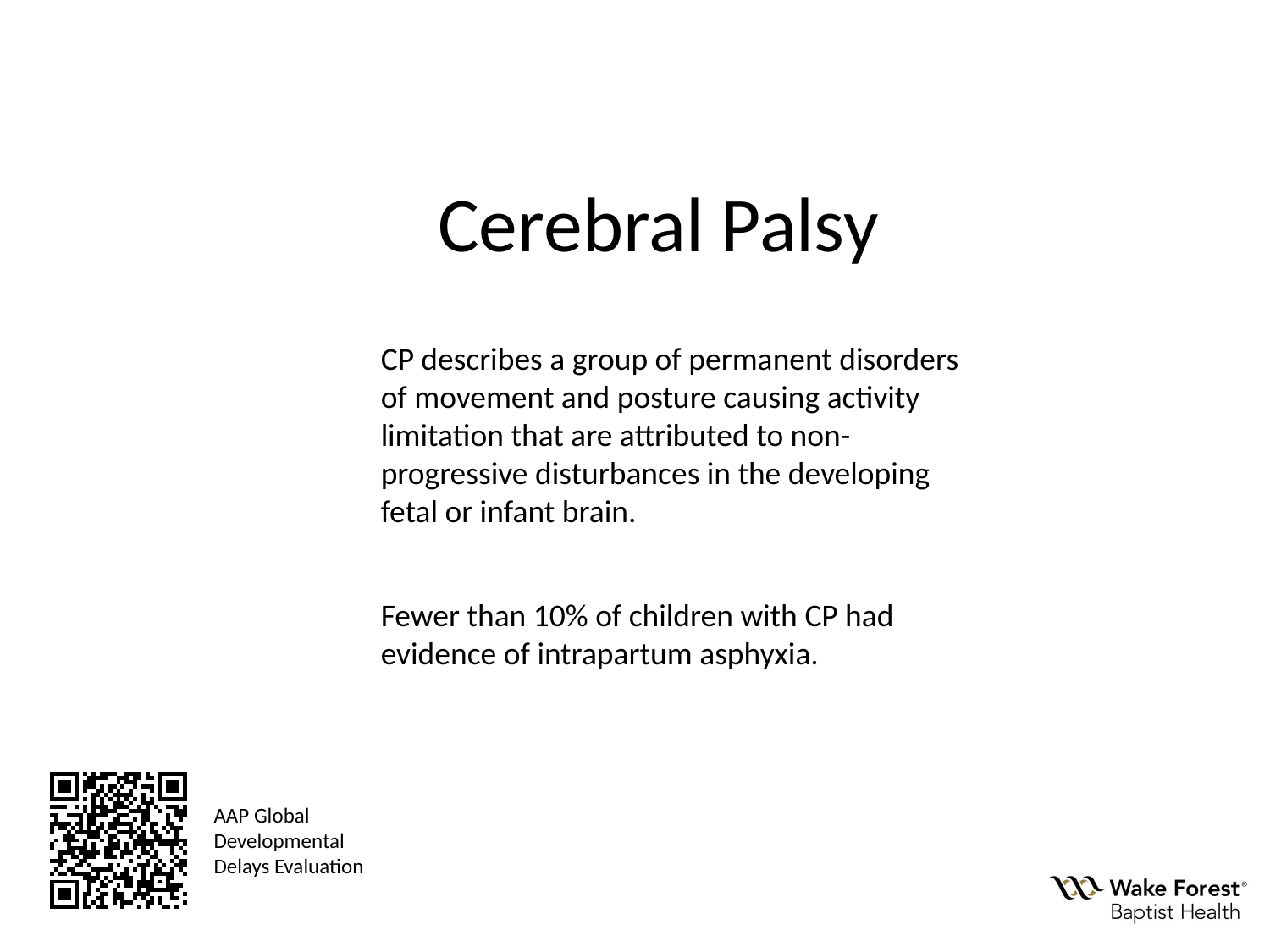

# Cerebral Palsy
CP describes a group of permanent disorders of movement and posture causing activity limitation that are attributed to non-progressive disturbances in the developing fetal or infant brain.
Fewer than 10% of children with CP had evidence of intrapartum asphyxia.
AAP Global Developmental Delays Evaluation

## Slide 14
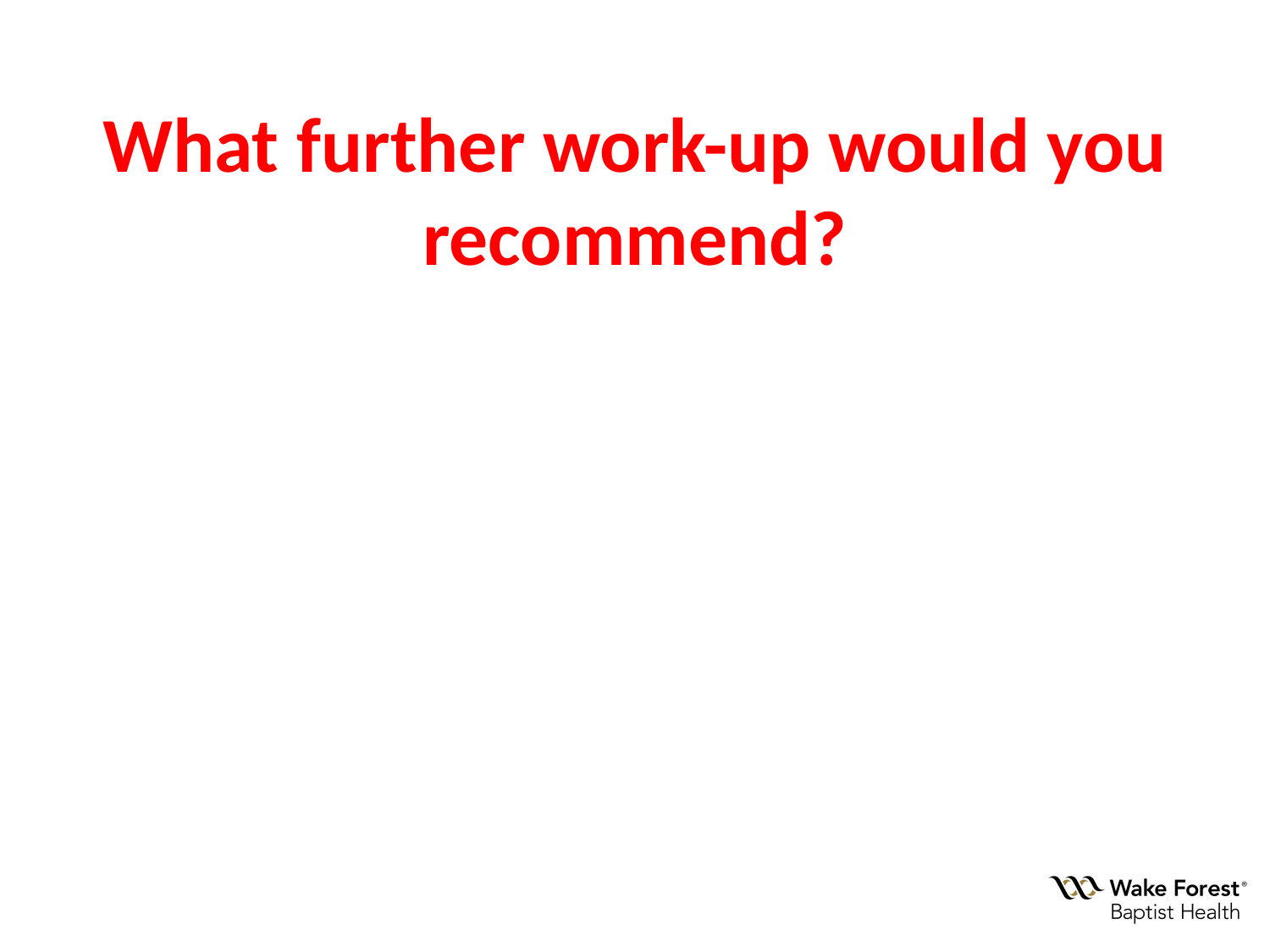

# What further work-up would you recommend?

## Slide 15
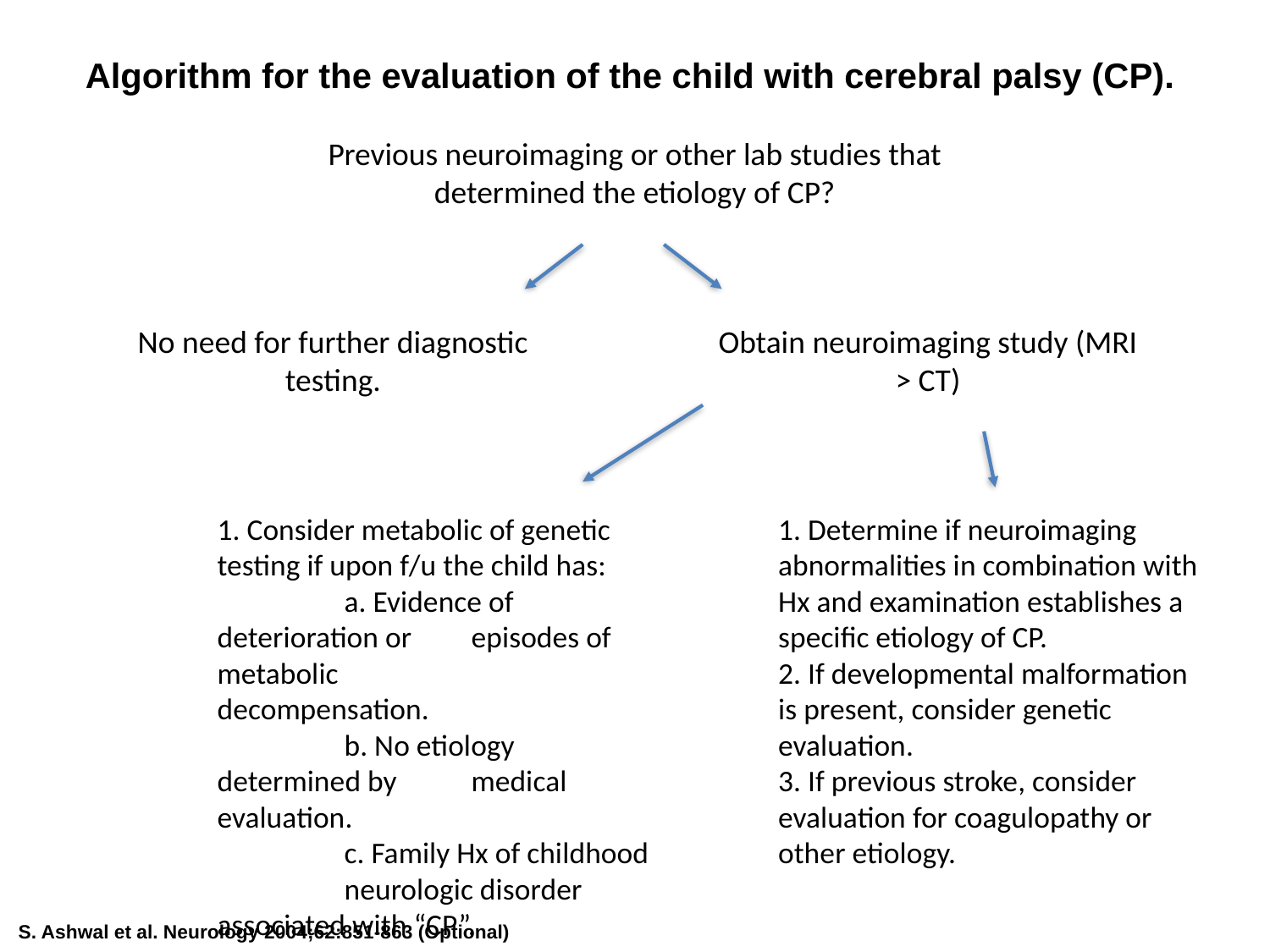

Algorithm for the evaluation of the child with cerebral palsy (CP).
Previous neuroimaging or other lab studies that determined the etiology of CP?
Obtain neuroimaging study (MRI > CT)
No need for further diagnostic testing.
1. Consider metabolic of genetic testing if upon f/u the child has:
	a. Evidence of deterioration or 	episodes of metabolic 	decompensation.
	b. No etiology determined by 	medical evaluation.
	c. Family Hx of childhood 	neurologic disorder 	associated with “CP”.
1. Determine if neuroimaging abnormalities in combination with Hx and examination establishes a specific etiology of CP.
2. If developmental malformation is present, consider genetic evaluation.
3. If previous stroke, consider evaluation for coagulopathy or other etiology.
S. Ashwal et al. Neurology 2004;62:851-863 (Optional)

## Slide 16
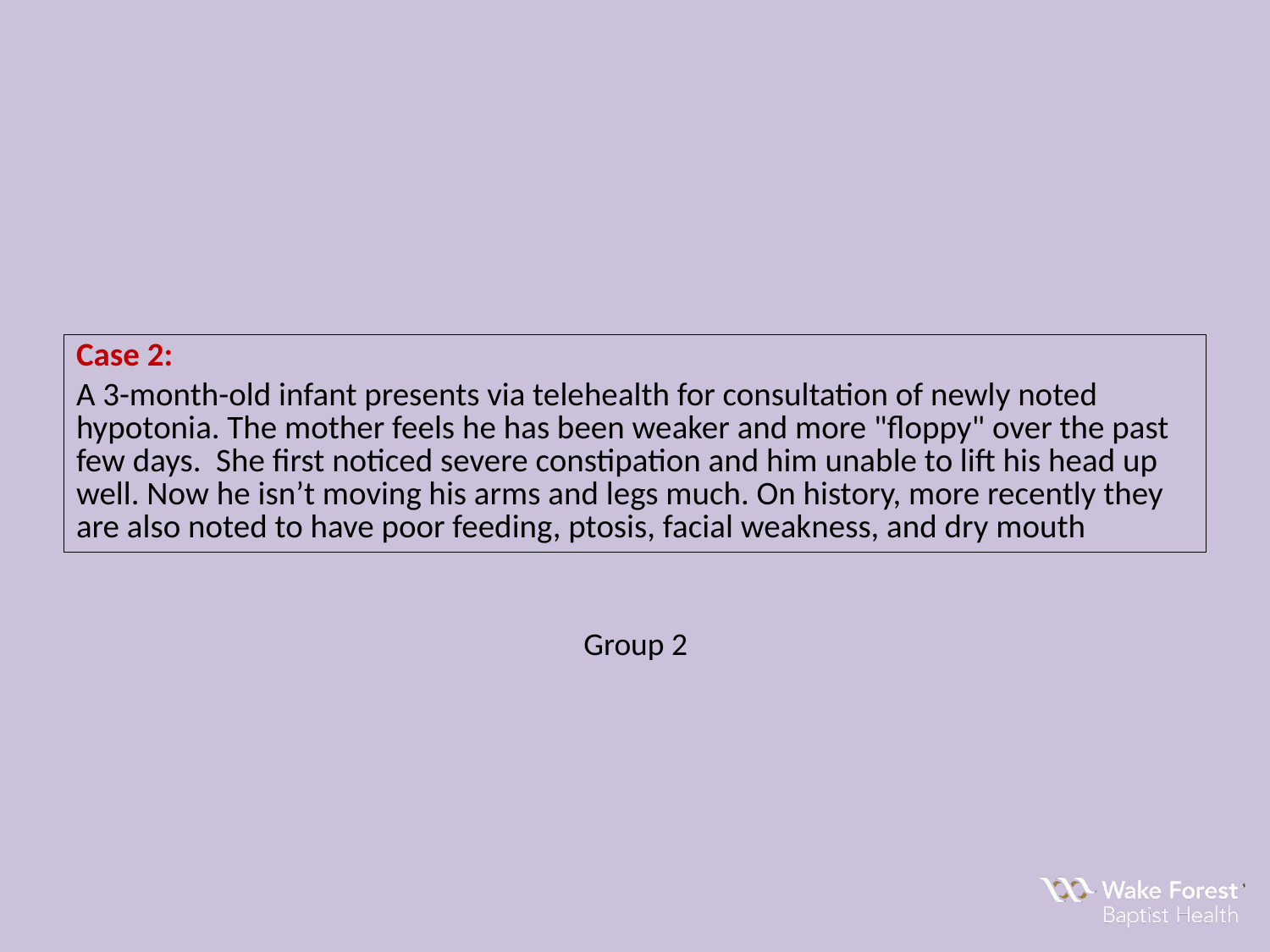

Case 2:
A 3-month-old infant presents via telehealth for consultation of newly noted hypotonia. The mother feels he has been weaker and more "floppy" over the past few days. She first noticed severe constipation and him unable to lift his head up well. Now he isn’t moving his arms and legs much. On history, more recently they are also noted to have poor feeding, ptosis, facial weakness, and dry mouth
Group 2

## Slide 17
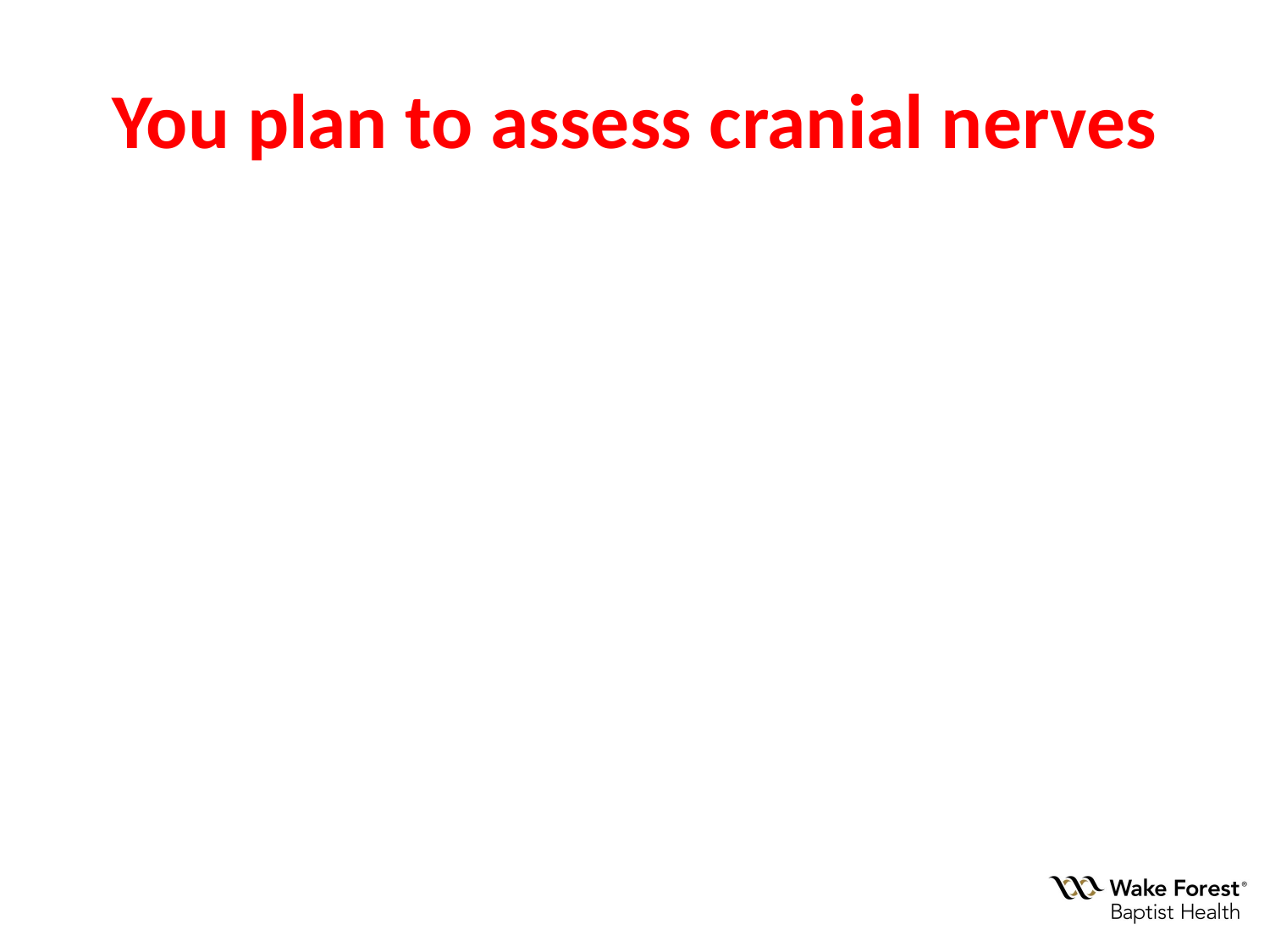

# You plan to assess cranial nerves

## Slide 18
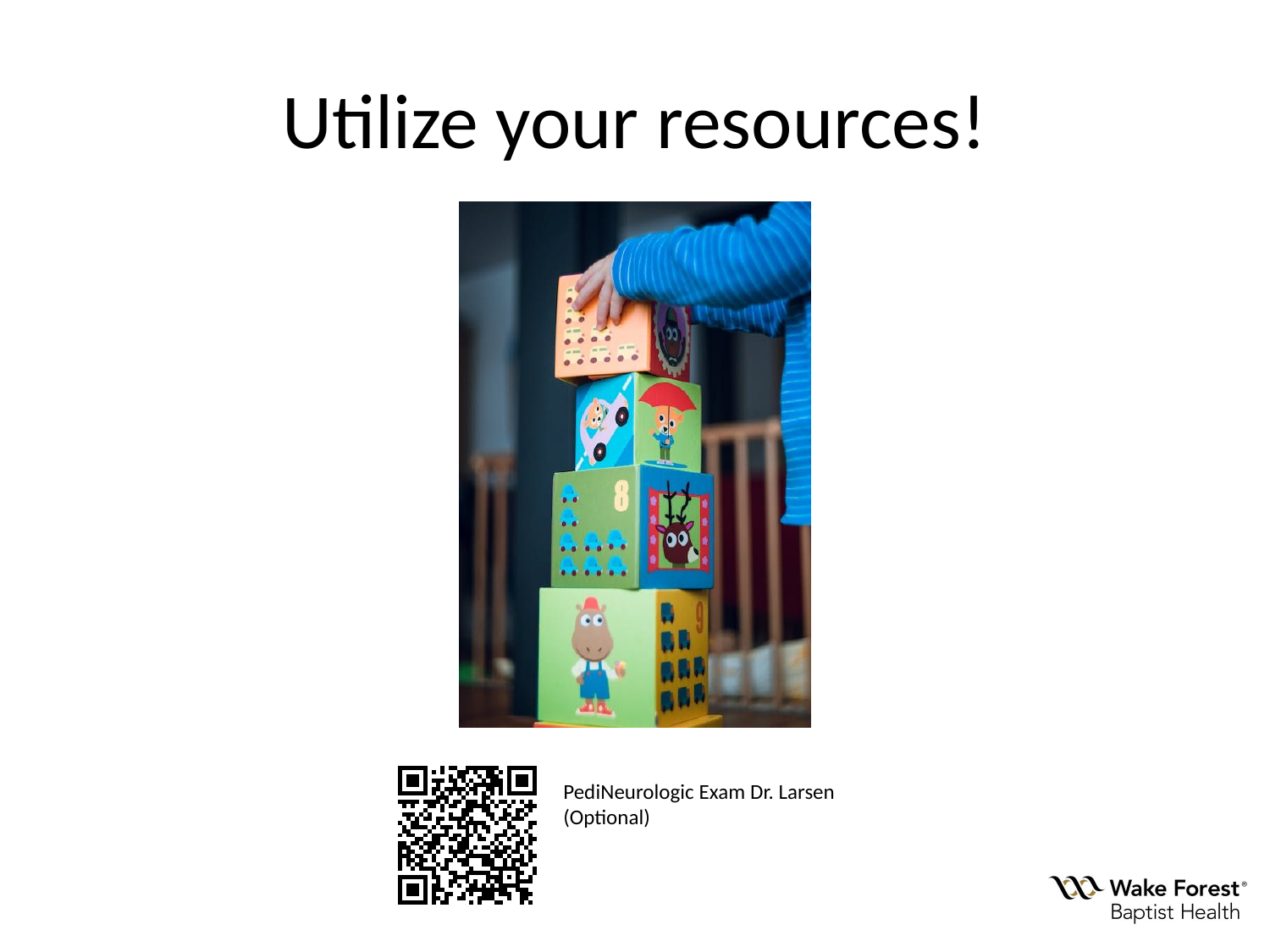

# Utilize your resources!
PediNeurologic Exam Dr. Larsen​
(Optional)

## Slide 19
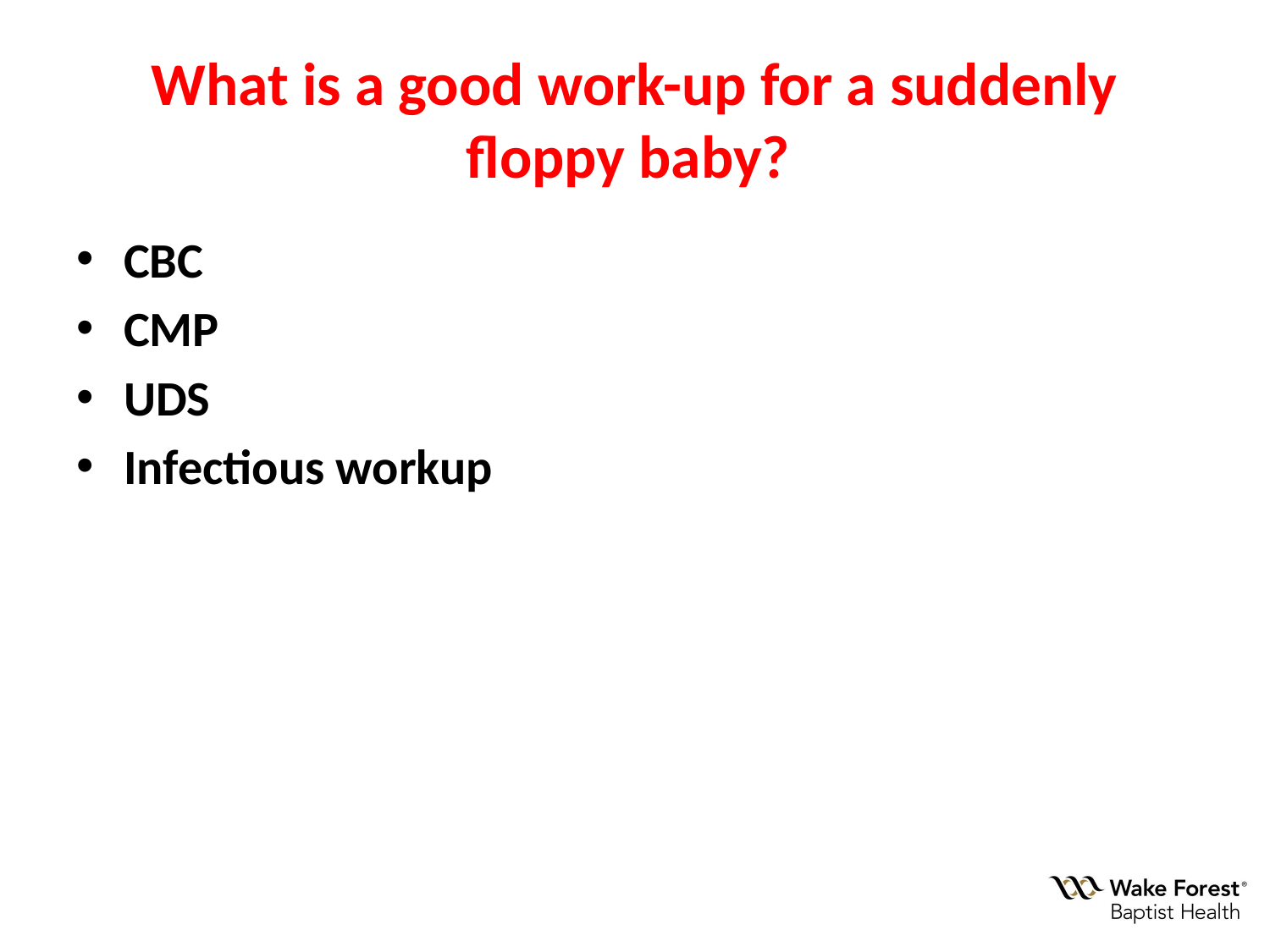

# What is a good work-up for a suddenly floppy baby?
CBC
CMP
UDS
Infectious workup

## Slide 20
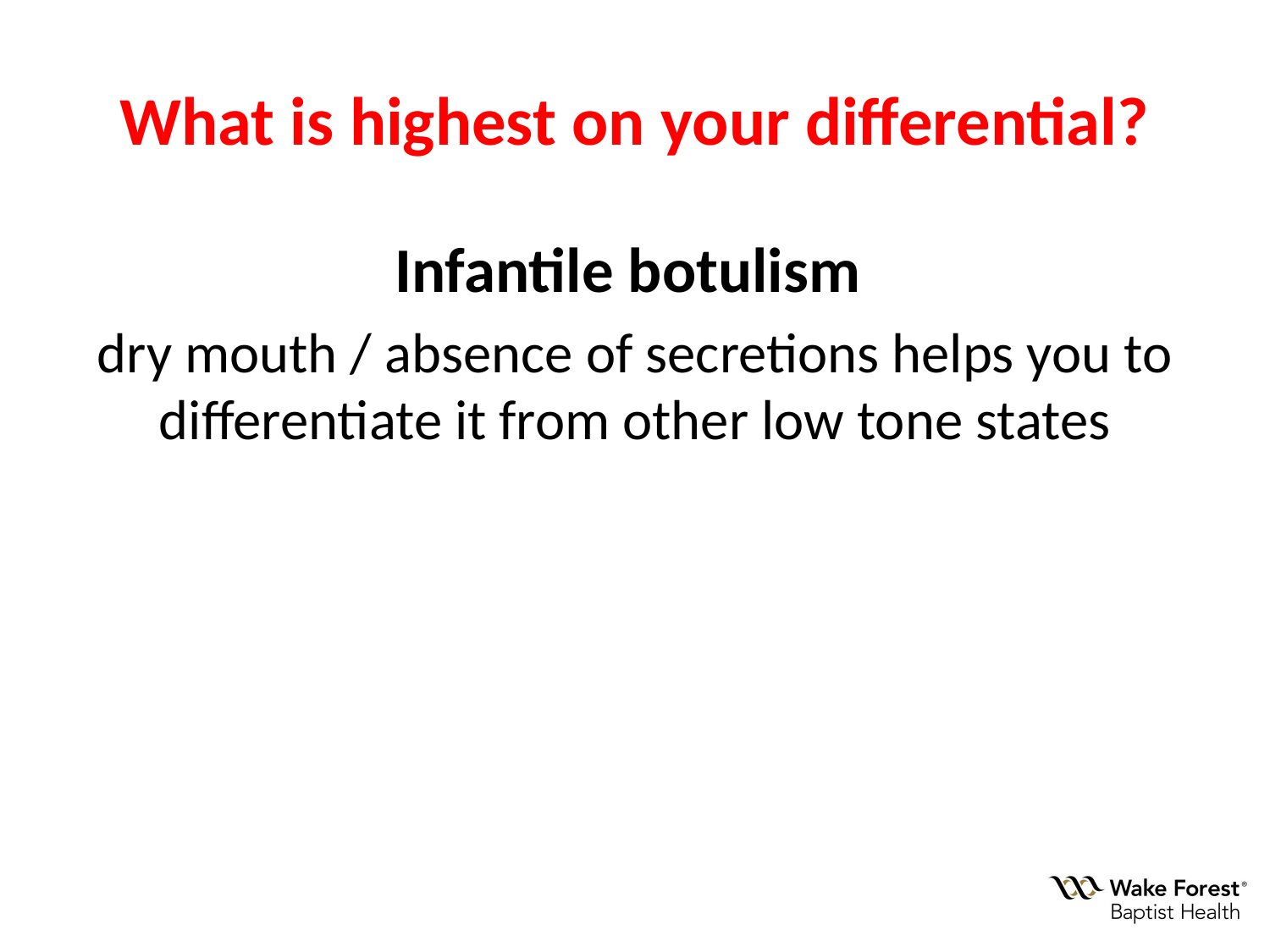

# What is highest on your differential?
Infantile botulism
dry mouth / absence of secretions helps you to differentiate it from other low tone states

## Slide 21
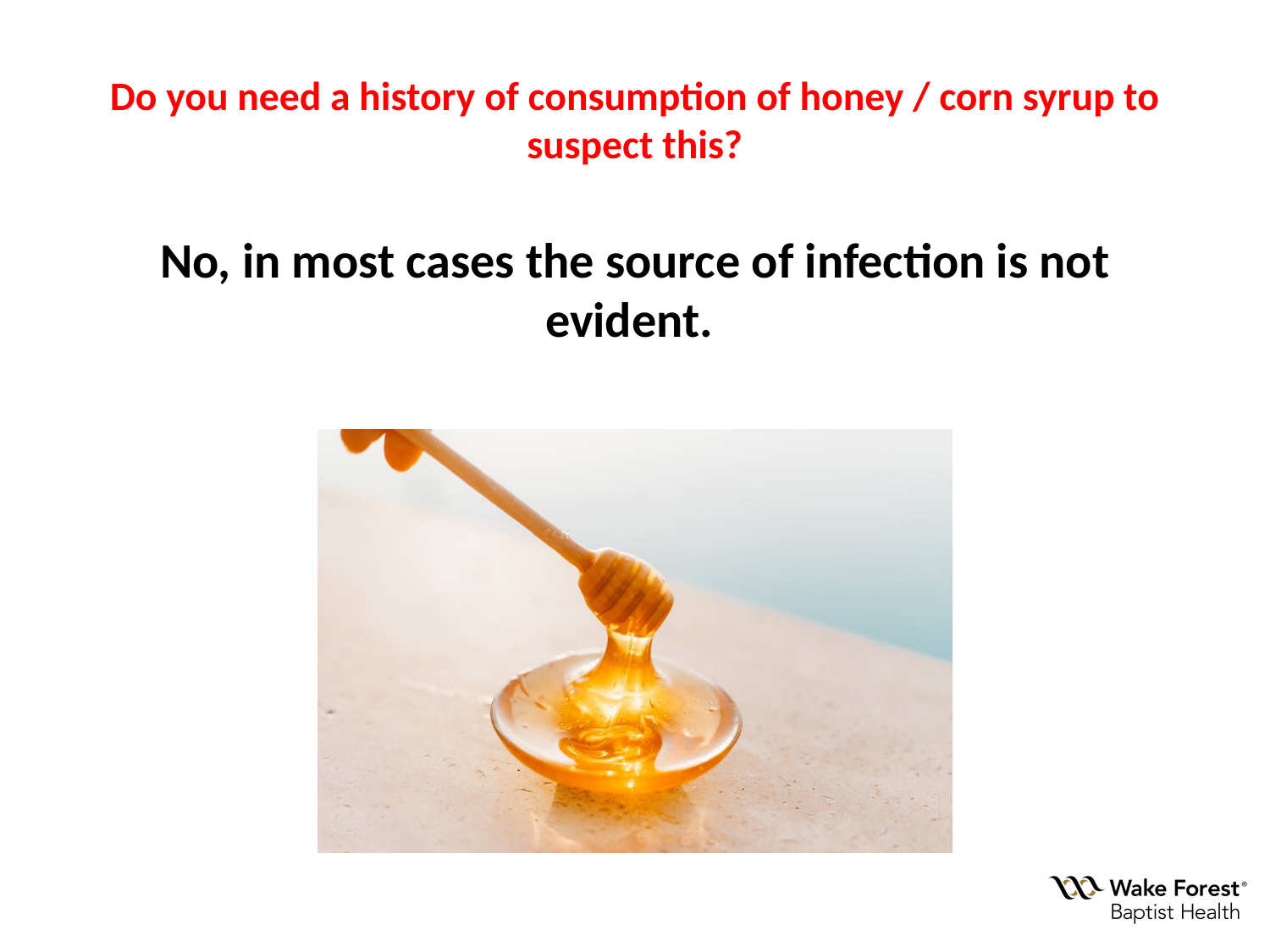

# Do you need a history of consumption of honey / corn syrup to suspect this?
No, in most cases the source of infection is not evident.

## Slide 22
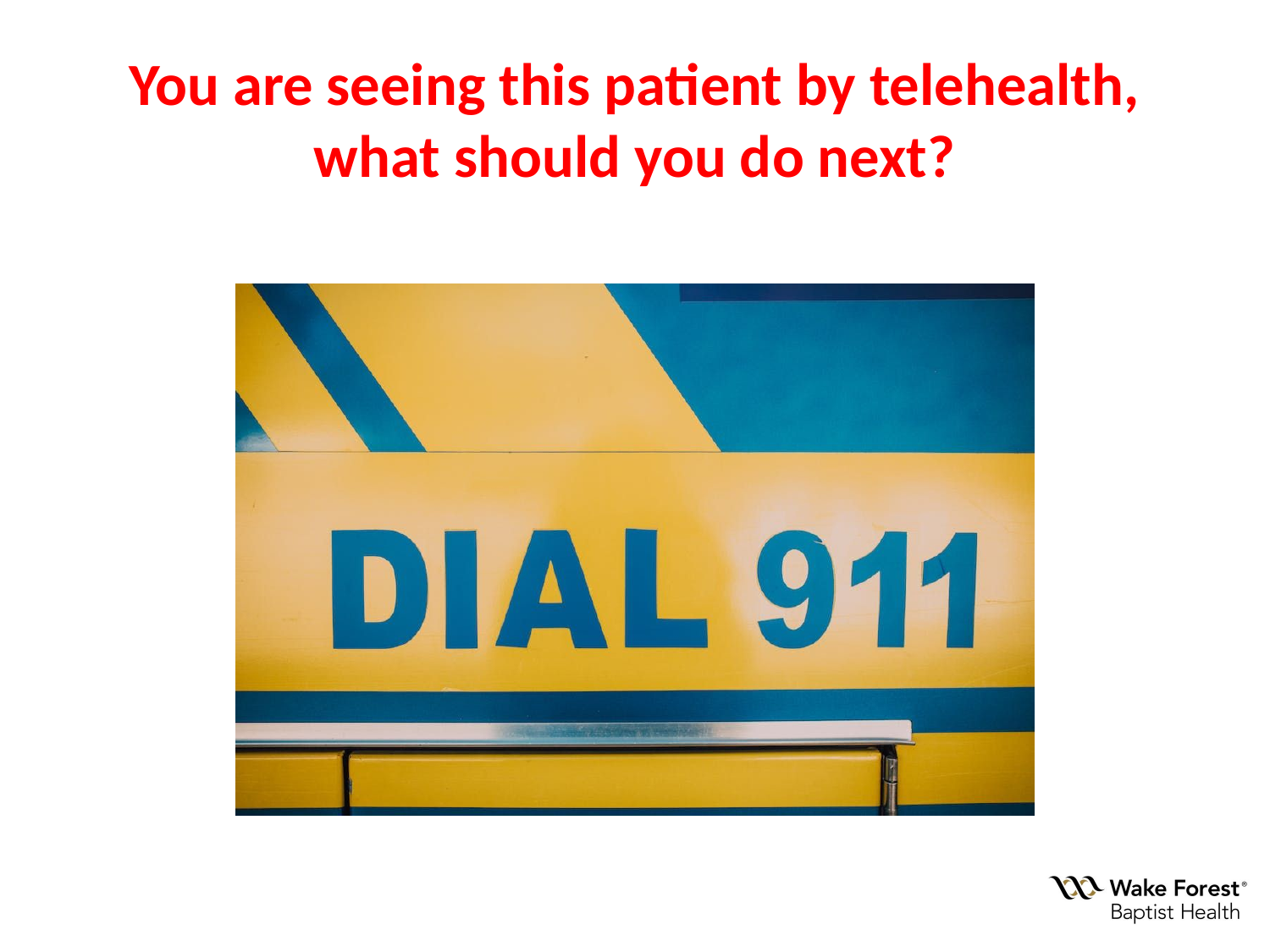

# You are seeing this patient by telehealth, what should you do next?

## Slide 23
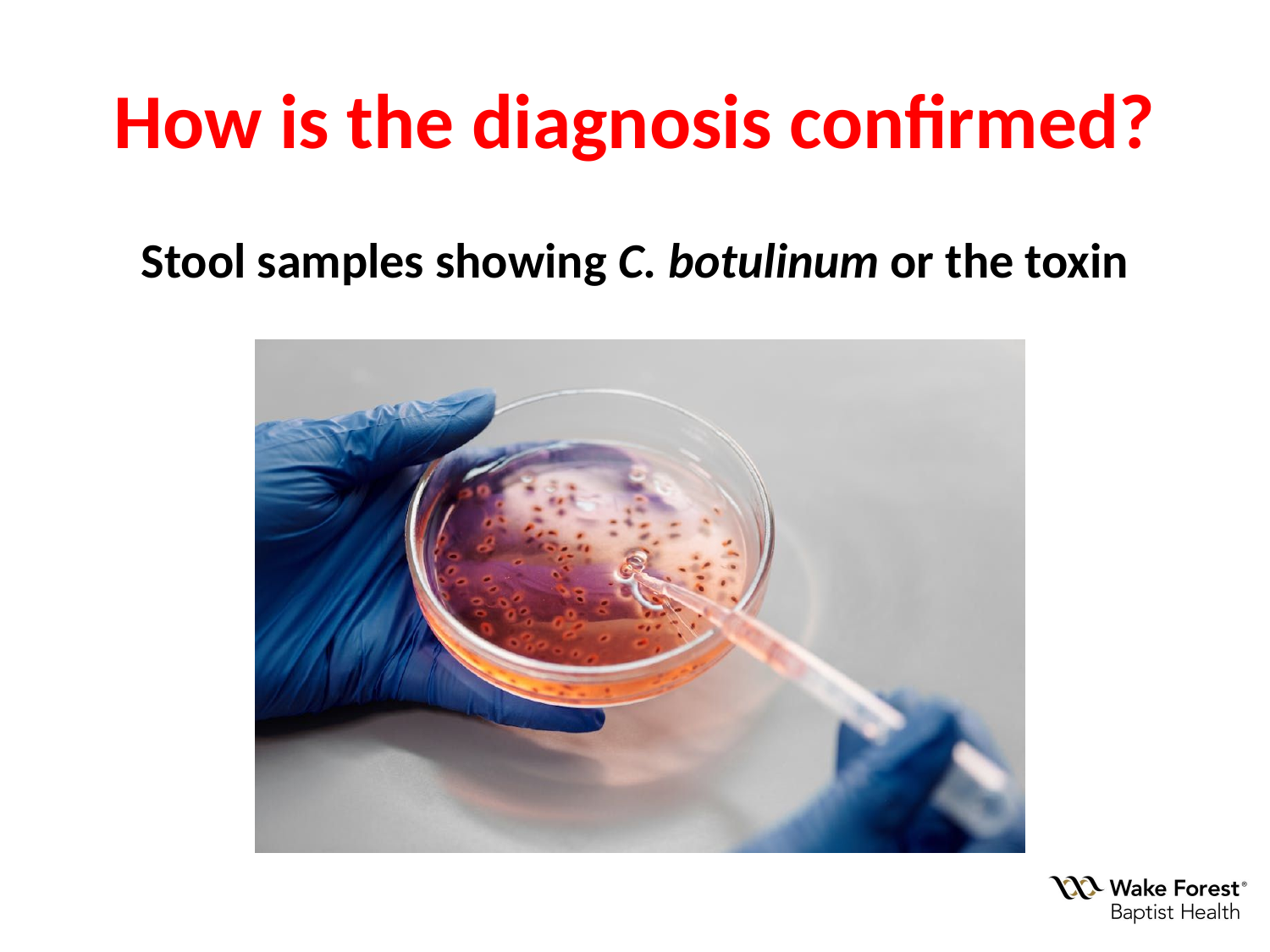

# How is the diagnosis confirmed?
Stool samples showing C. botulinum or the toxin

## Slide 24
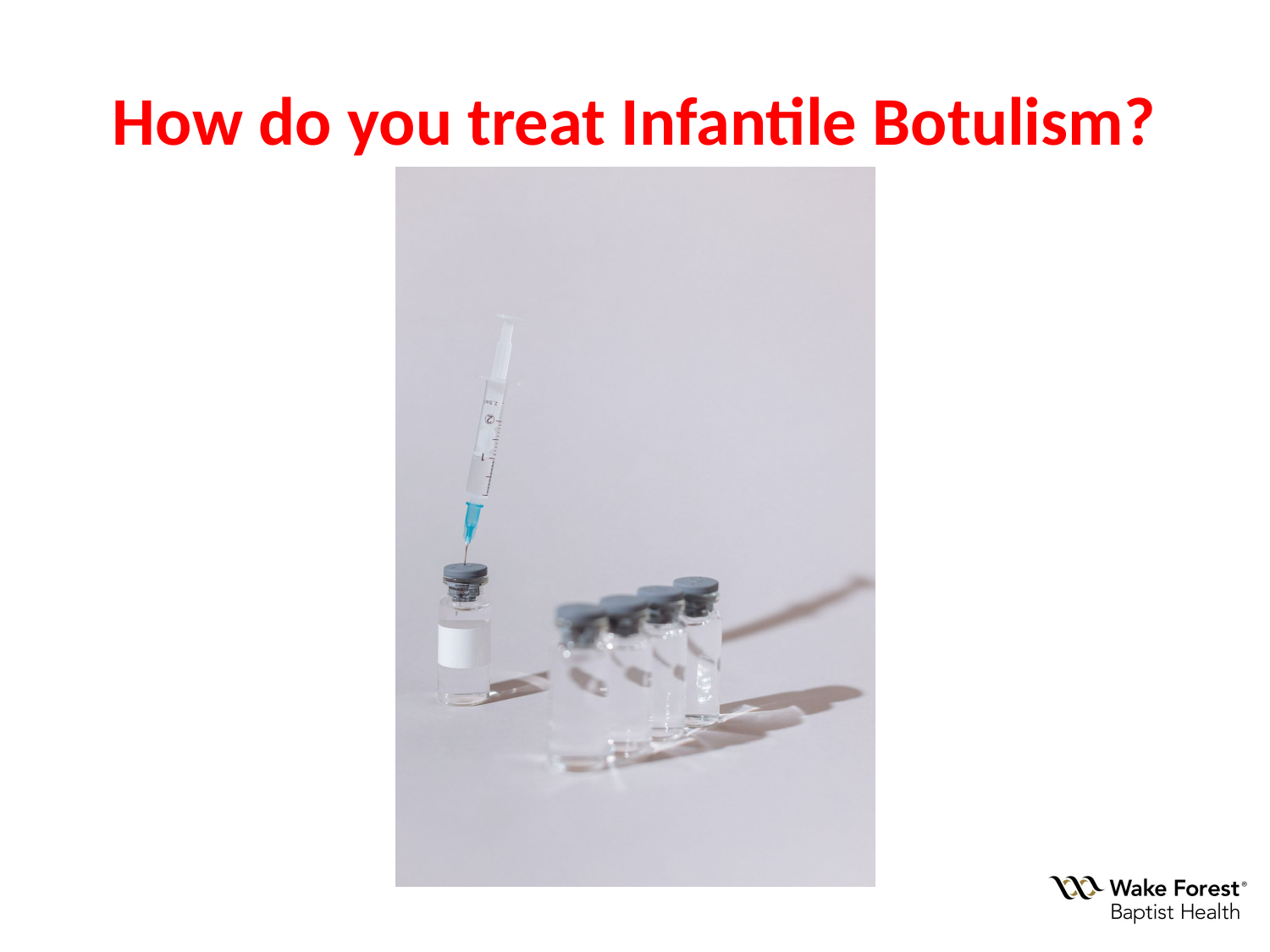

# How do you treat Infantile Botulism?

## Slide 25
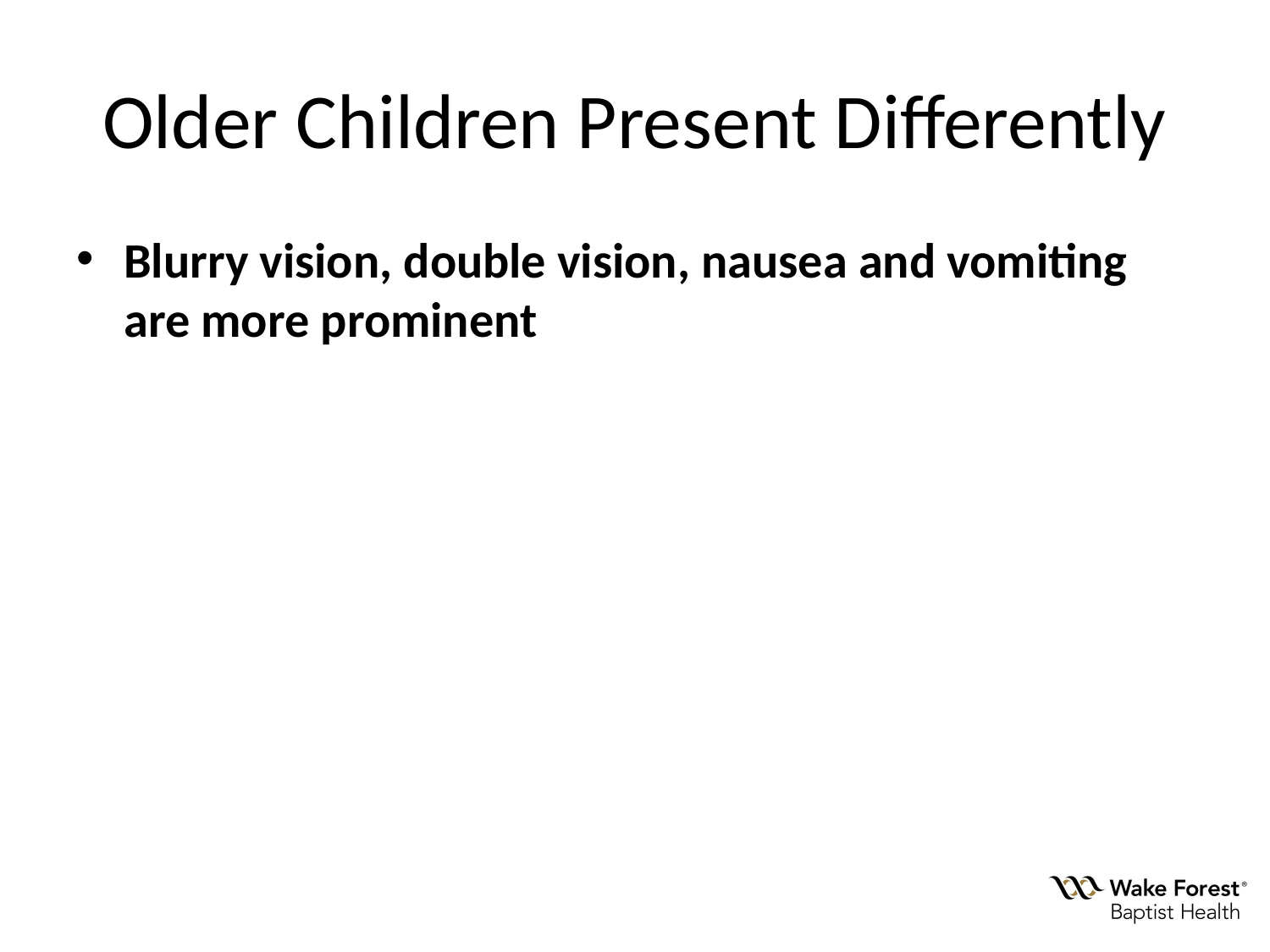

# Older Children Present Differently
Blurry vision, double vision, nausea and vomiting are more prominent

## Slide 26
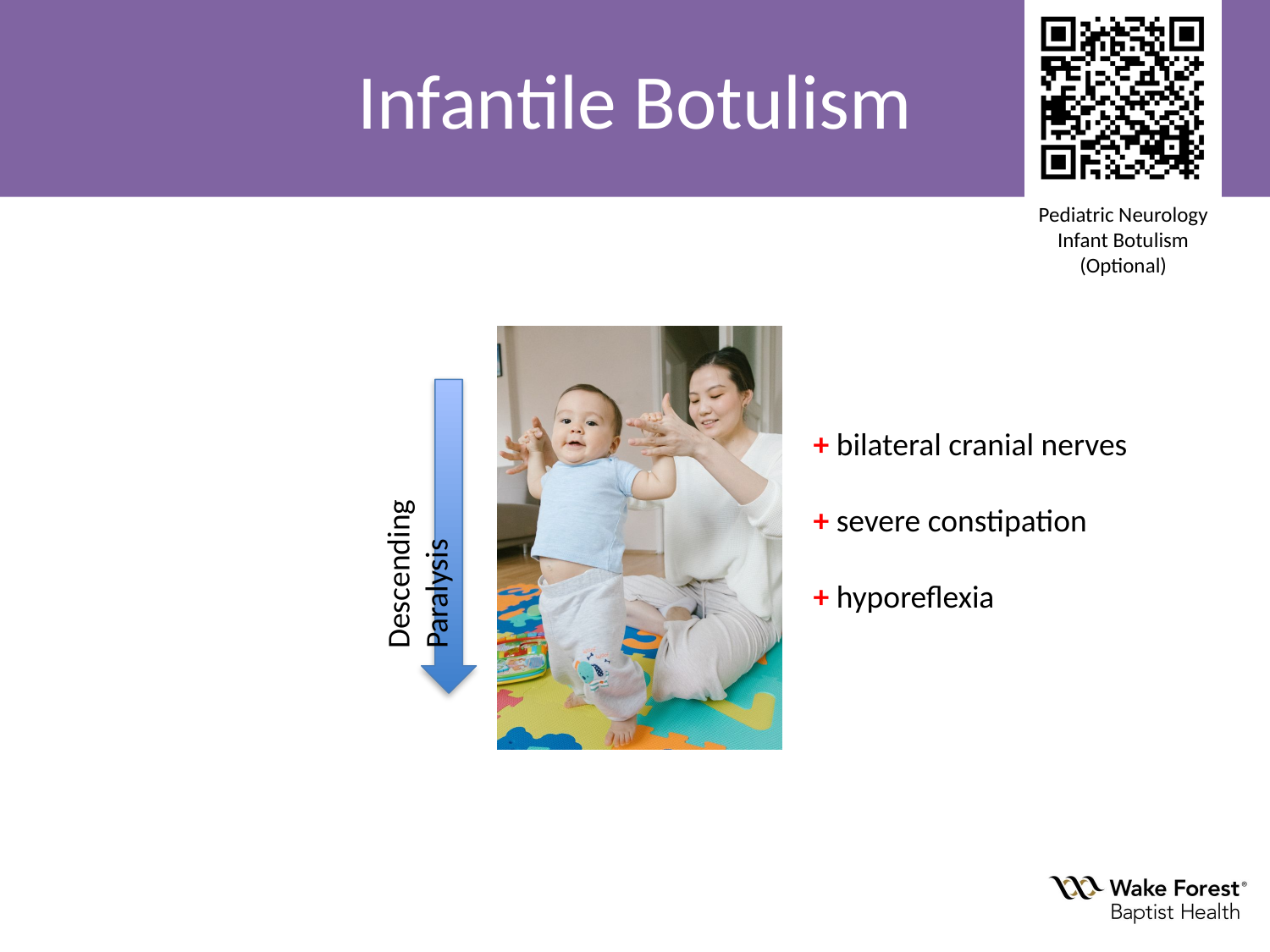

# Infantile Botulism
Pediatric Neurology Infant Botulism (Optional)
Descending Paralysis
+ bilateral cranial nerves
+ severe constipation
+ hyporeflexia

## Slide 27
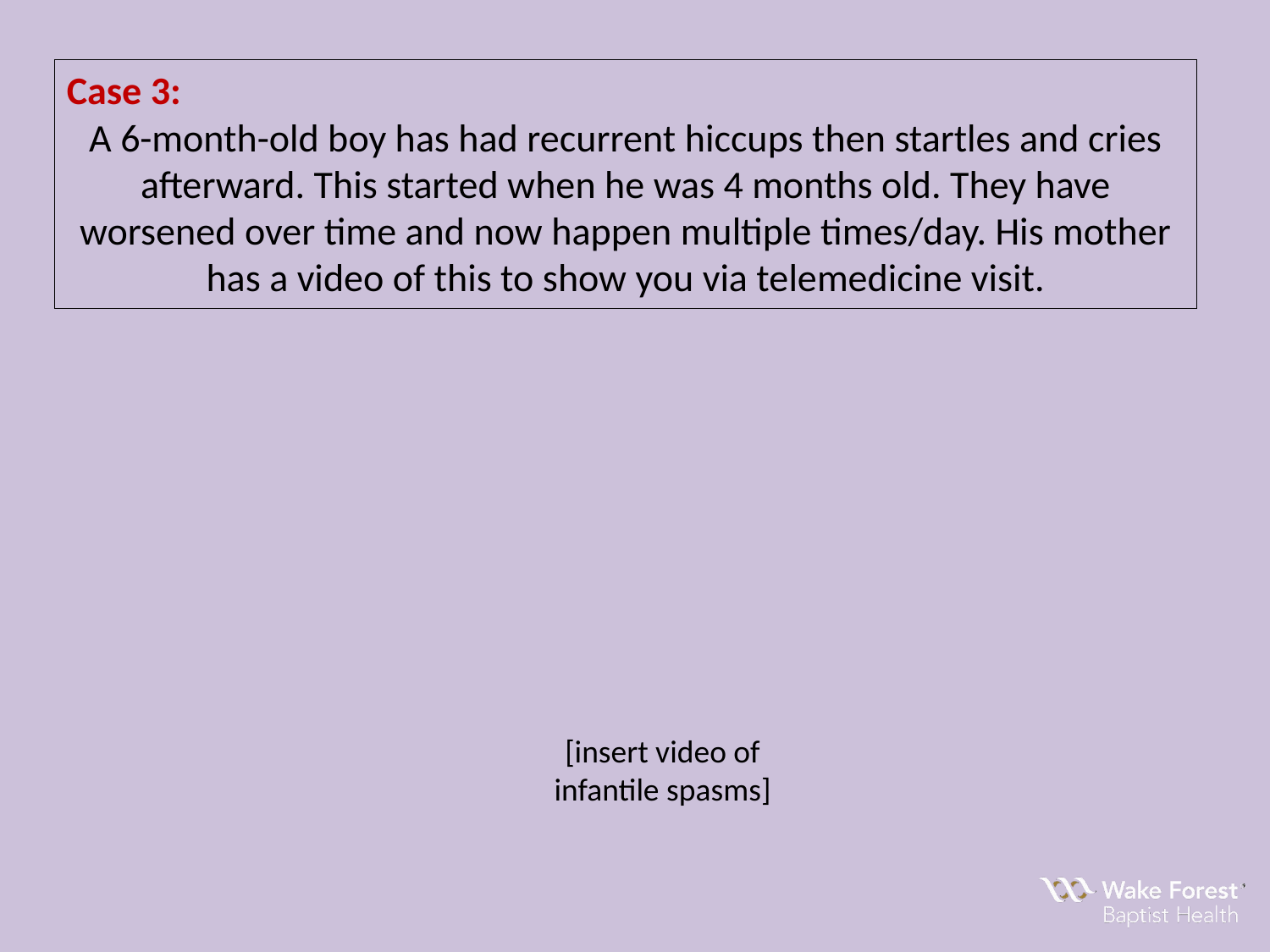

Case 3:
A 6-month-old boy has had recurrent hiccups then startles and cries afterward. This started when he was 4 months old. They have worsened over time and now happen multiple times/day. His mother has a video of this to show you via telemedicine visit.
[insert video of infantile spasms]

## Slide 28
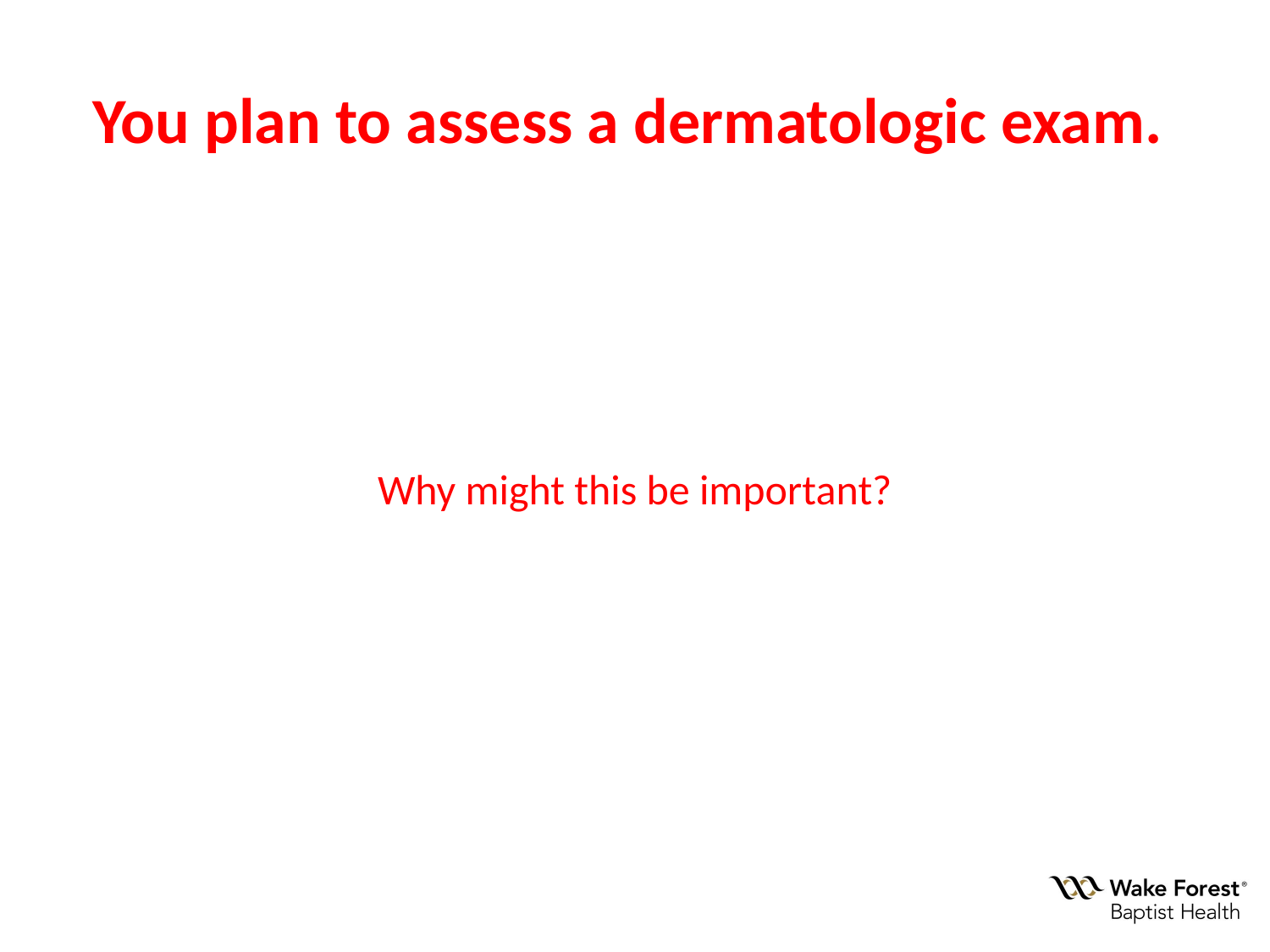

# You plan to assess a dermatologic exam.
Why might this be important?

## Slide 29
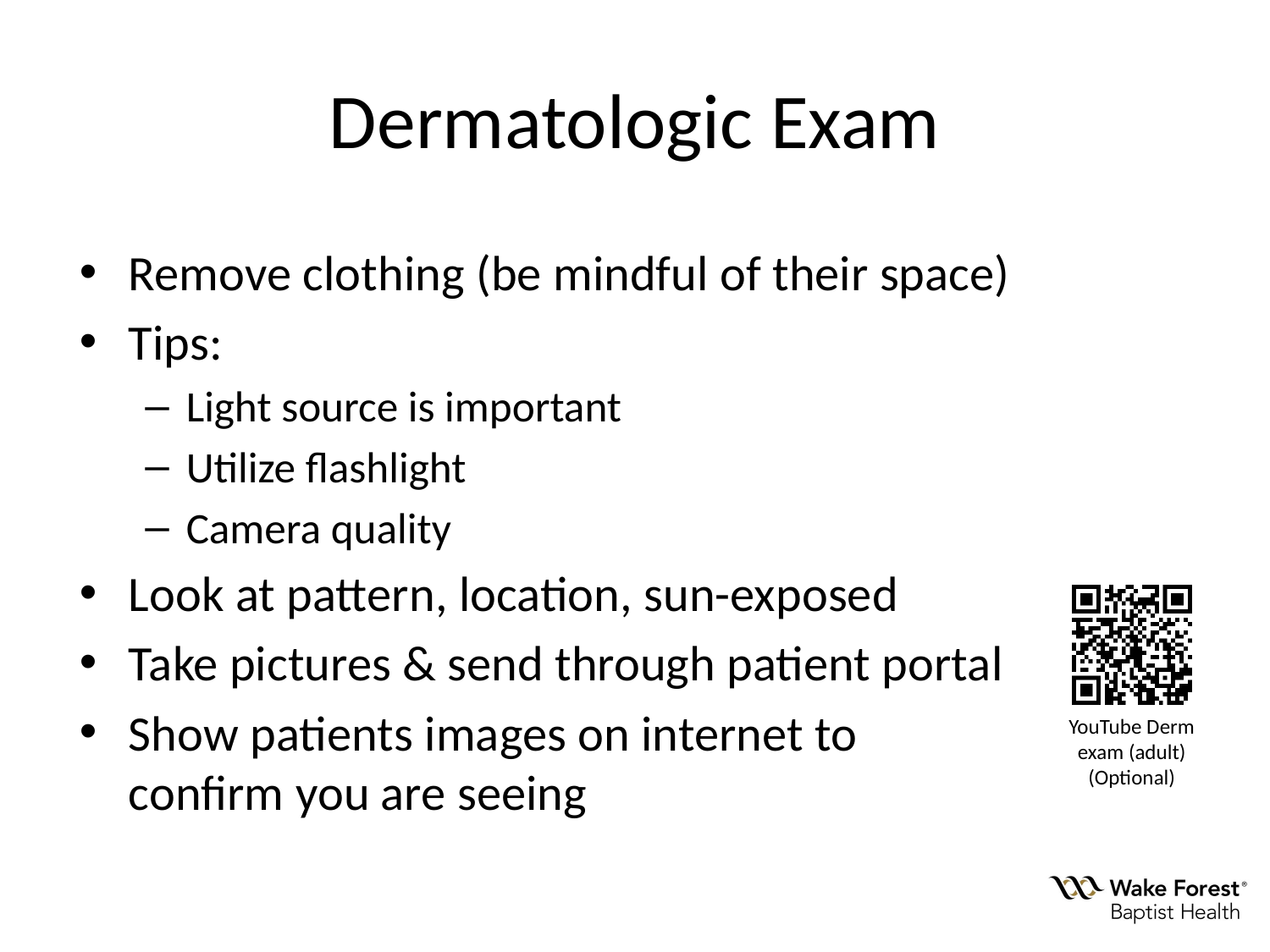

# Dermatologic Exam
Remove clothing (be mindful of their space)
Tips:
Light source is important
Utilize flashlight
Camera quality
Look at pattern, location, sun-exposed
Take pictures & send through patient portal
Show patients images on internet to confirm you are seeing
YouTube Derm exam (adult)
(Optional)

## Slide 30
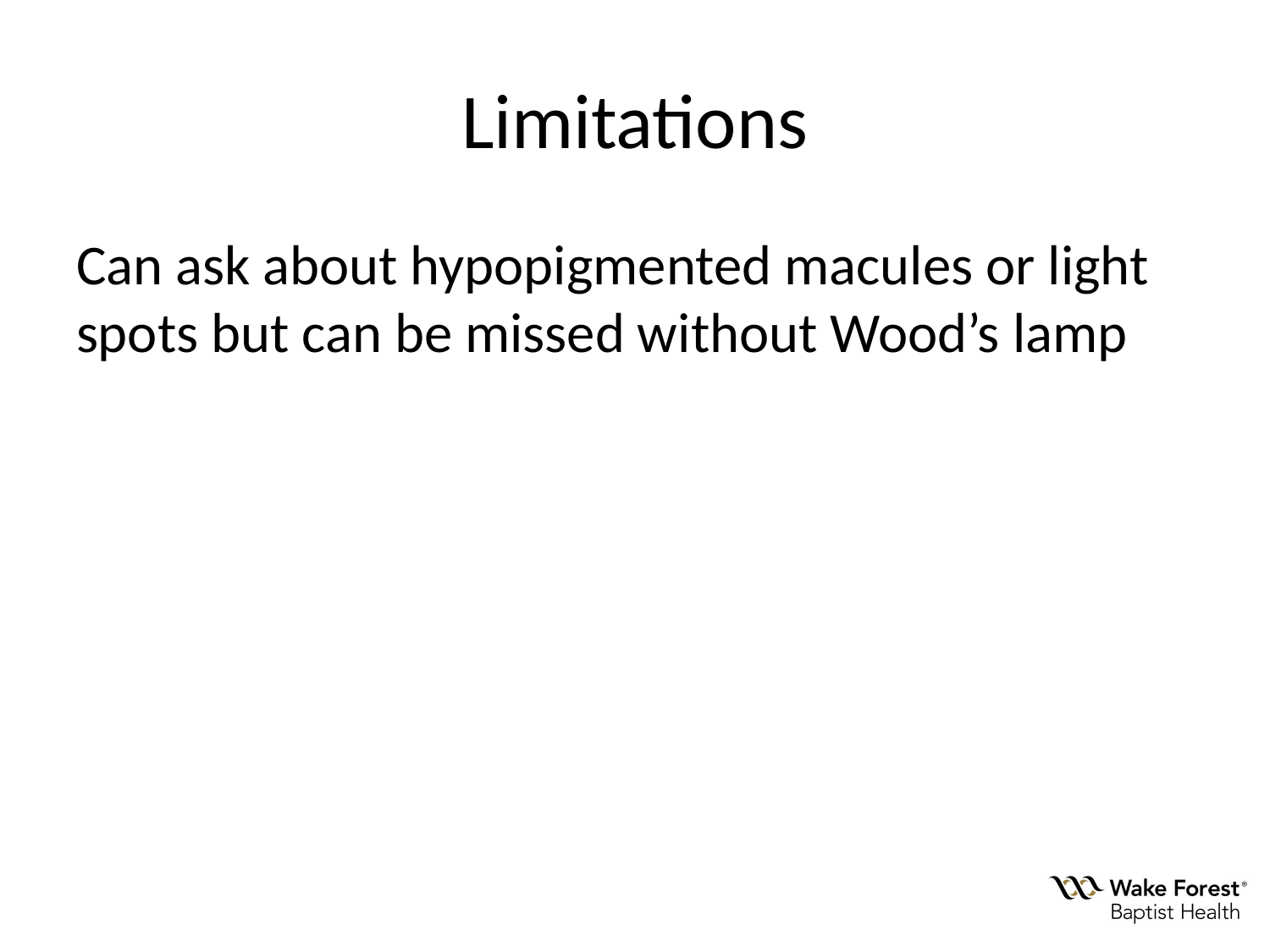

# Limitations
Can ask about hypopigmented macules or light spots but can be missed without Wood’s lamp

## Slide 31
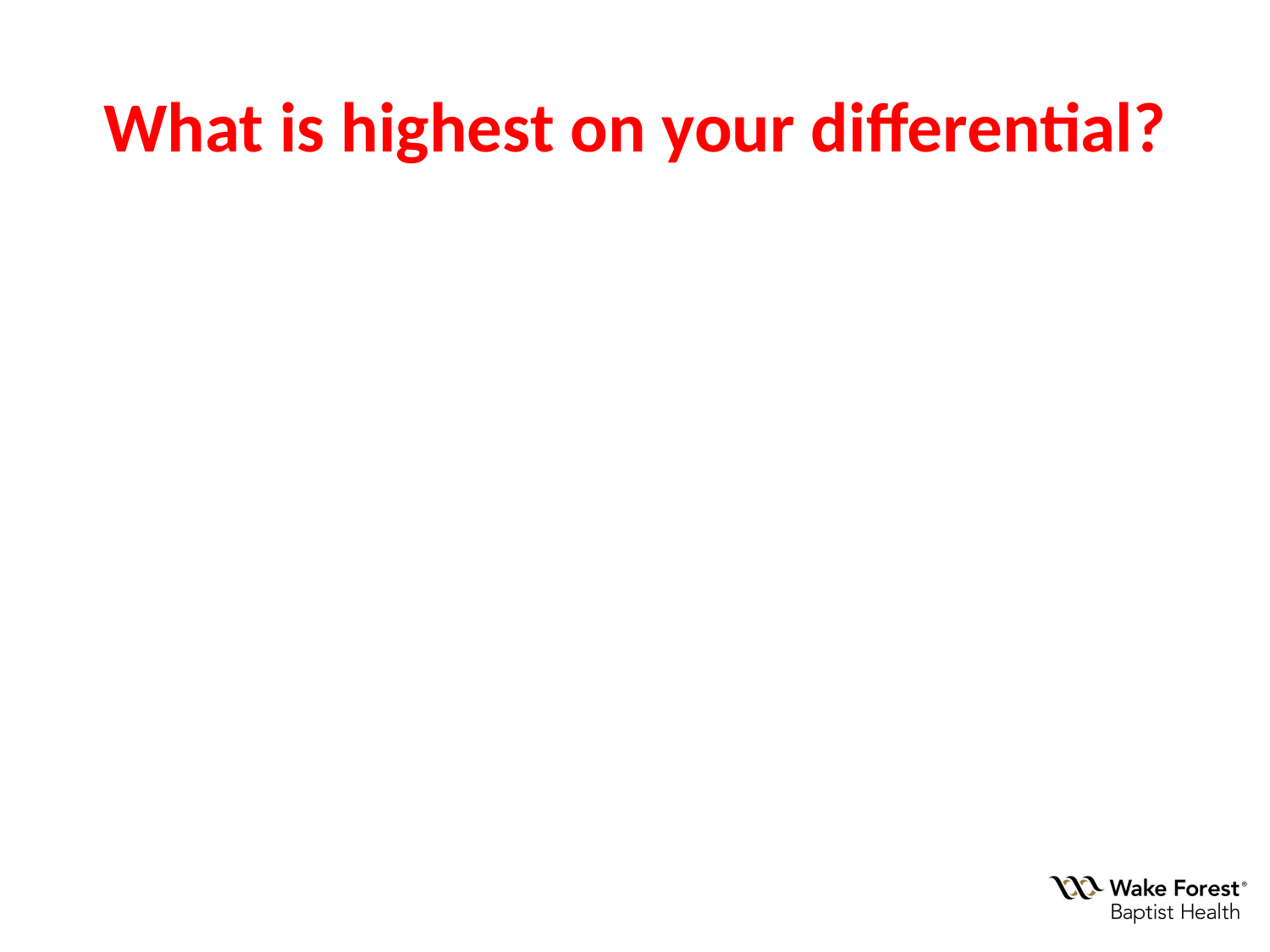

What is highest on your differential?

## Slide 32
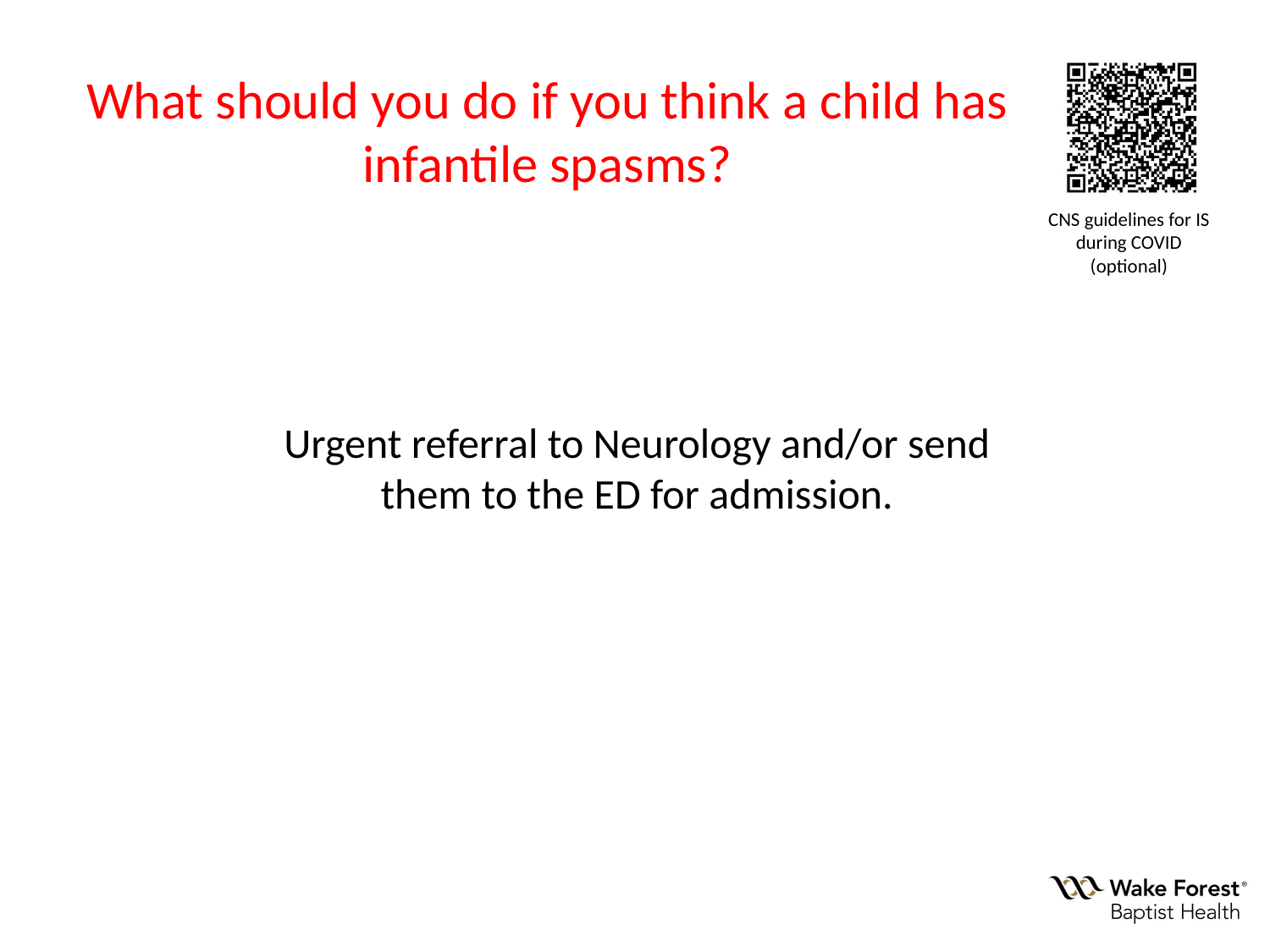

What should you do if you think a child has infantile spasms?
CNS guidelines for IS during COVID
(optional)
Urgent referral to Neurology and/or send them to the ED for admission.

## Slide 33
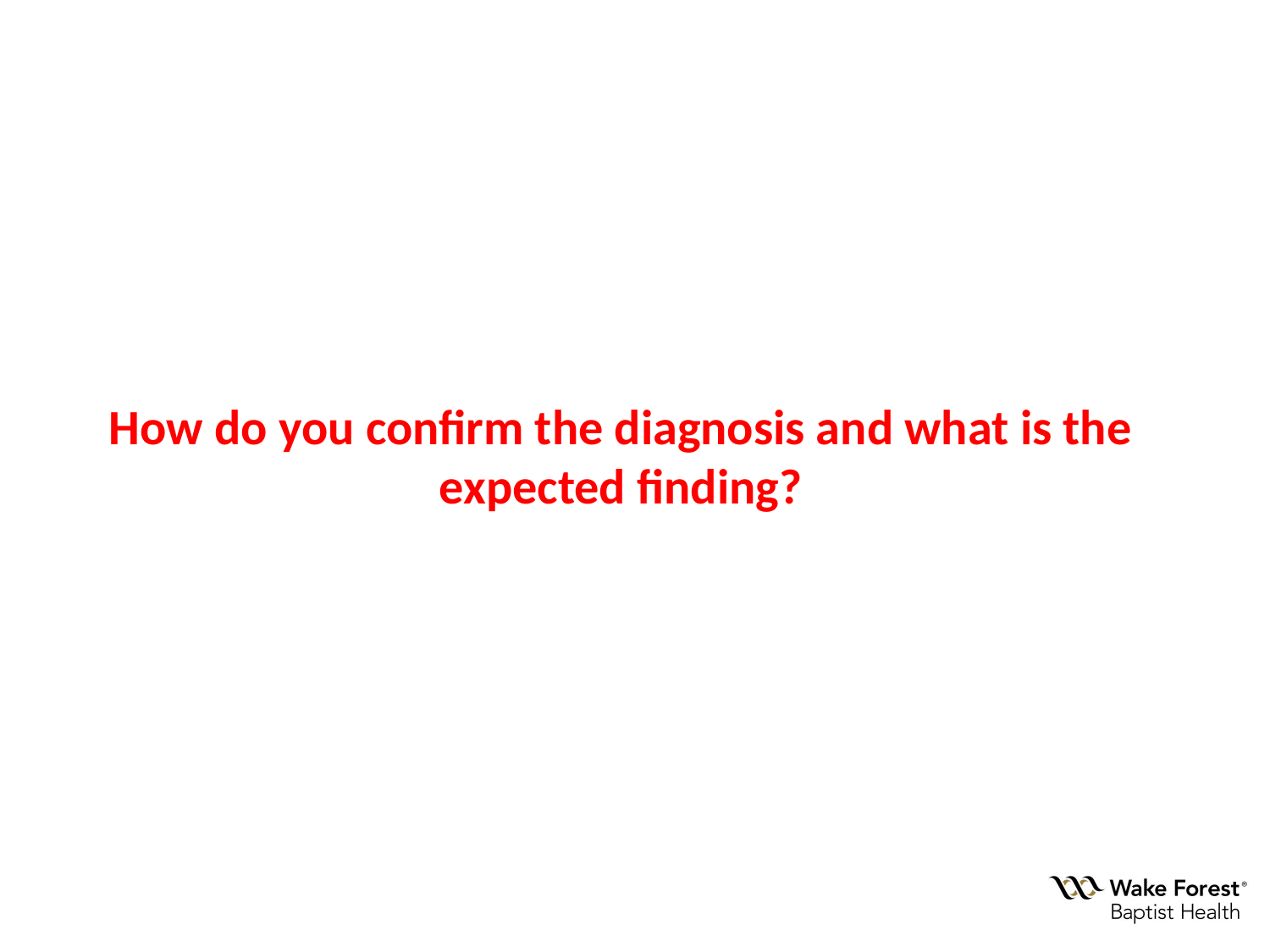

How do you confirm the diagnosis and what is the expected finding?

## Slide 34
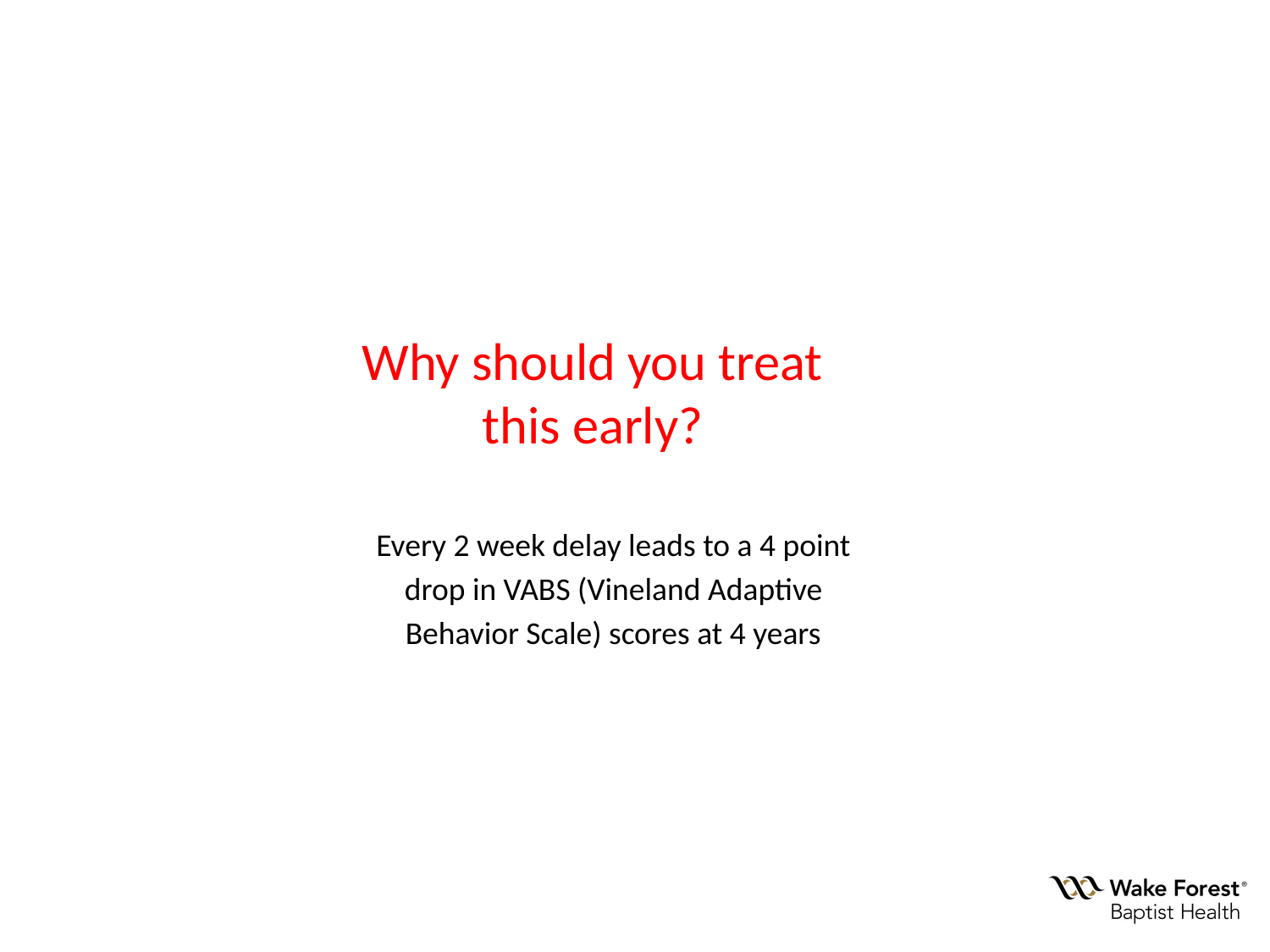

Why should you treat this early?
Every 2 week delay leads to a 4 point drop in VABS (Vineland Adaptive Behavior Scale) scores at 4 years

## Slide 35
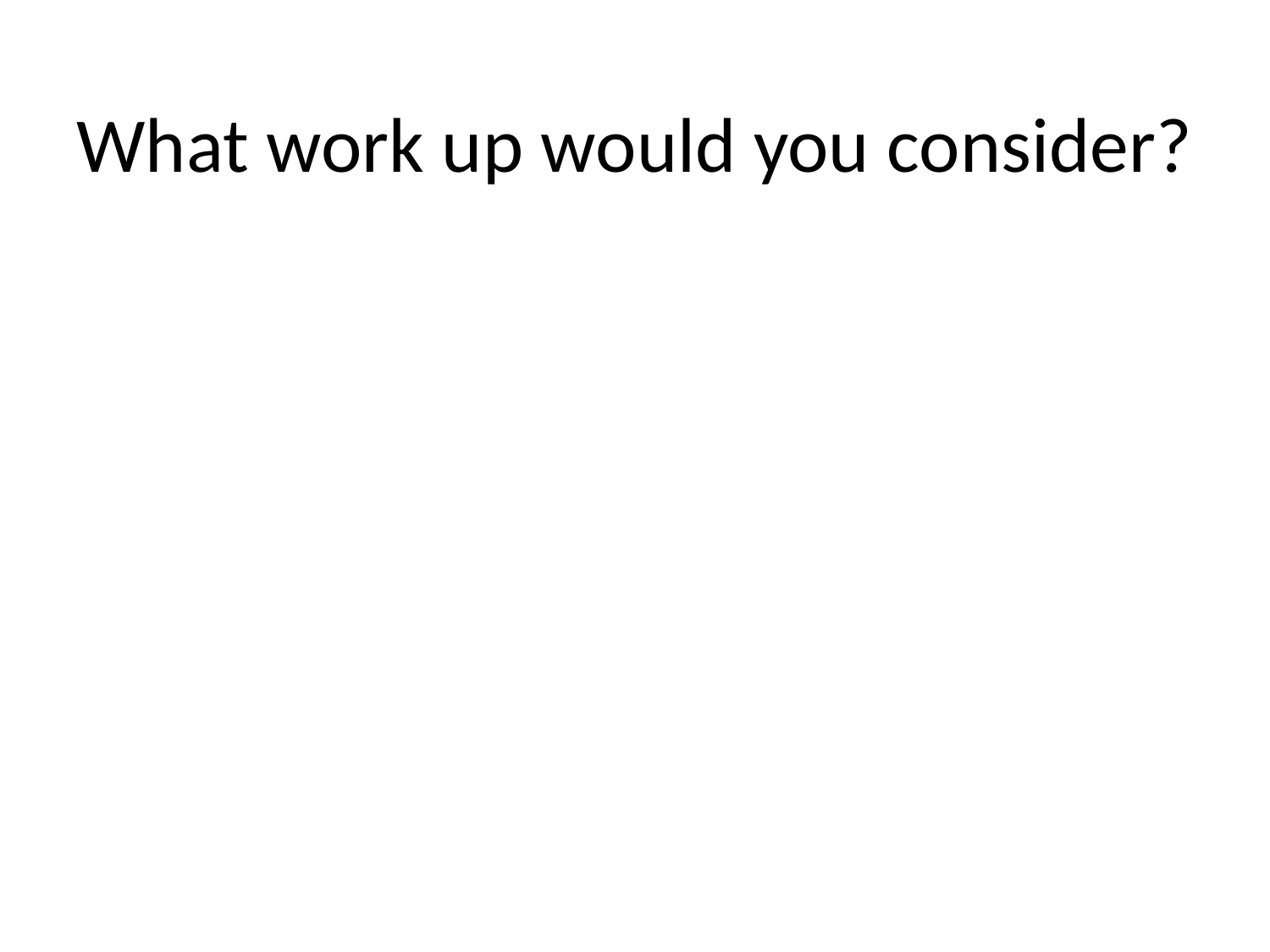

# What work up would you consider?

## Slide 36
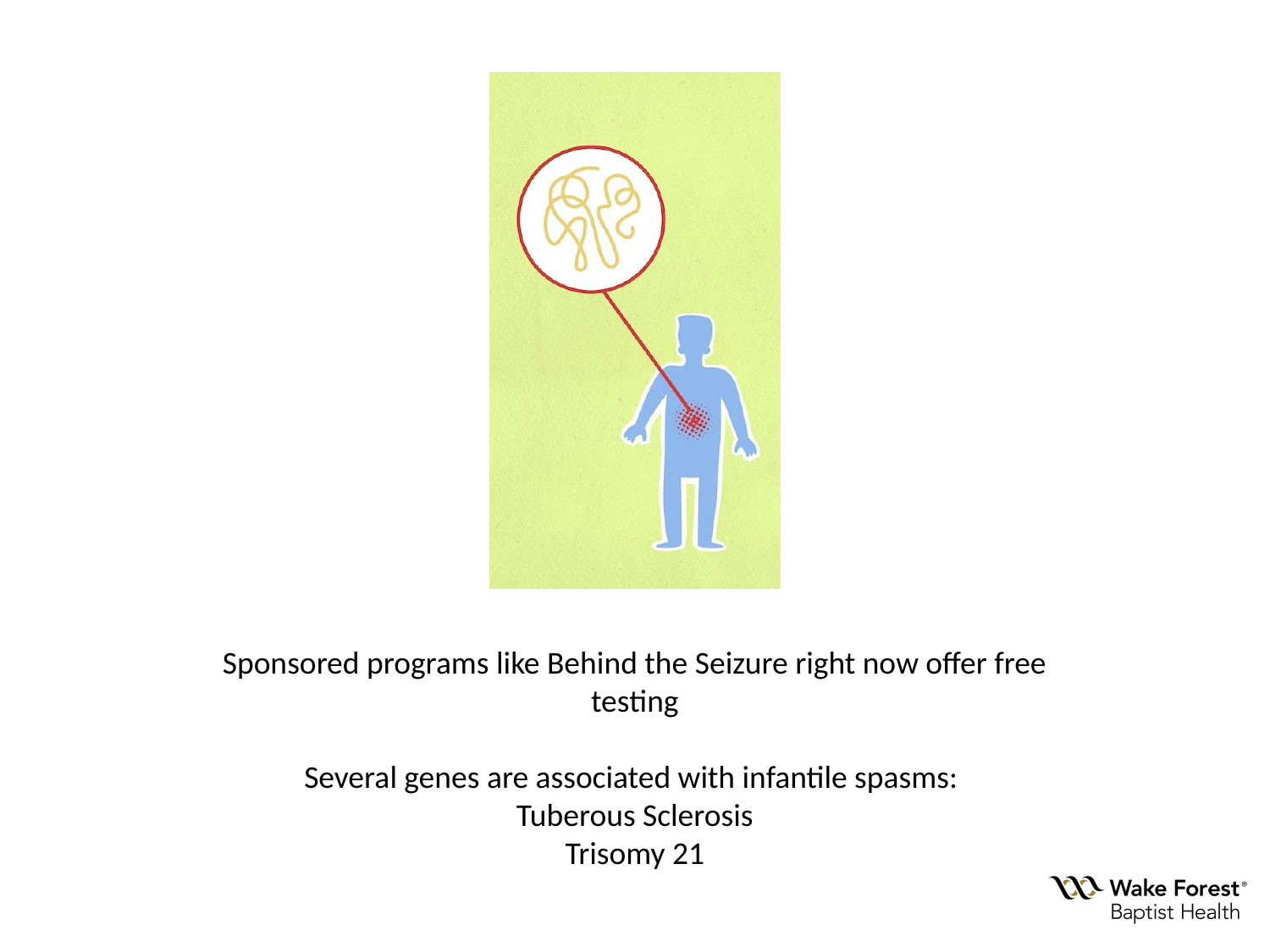

Sponsored programs like Behind the Seizure right now offer free testing
Several genes are associated with infantile spasms:
Tuberous Sclerosis
Trisomy 21

## Slide 37
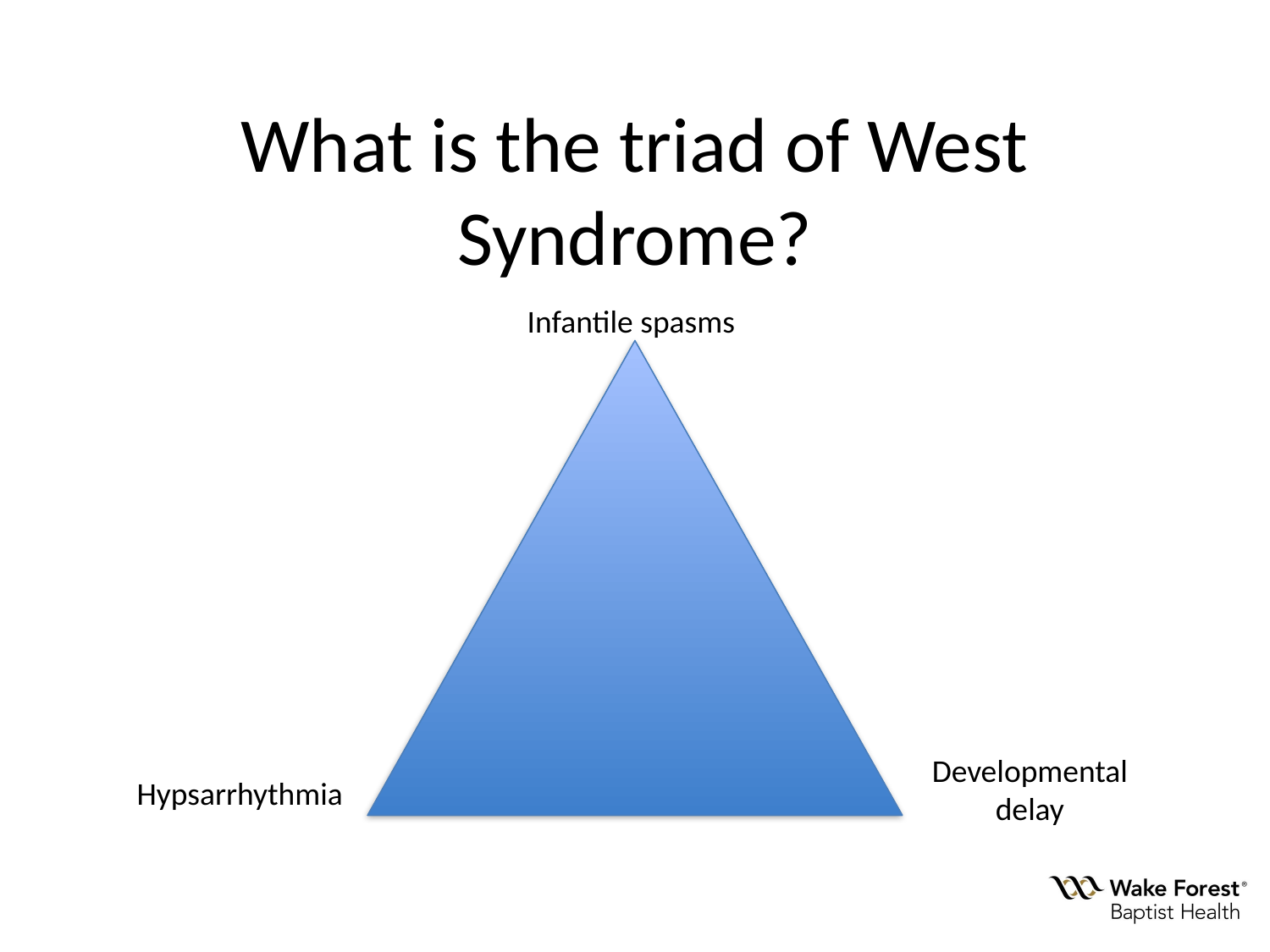

# What is the triad of West Syndrome?
Infantile spasms
Developmental delay
Hypsarrhythmia

## Slide 38
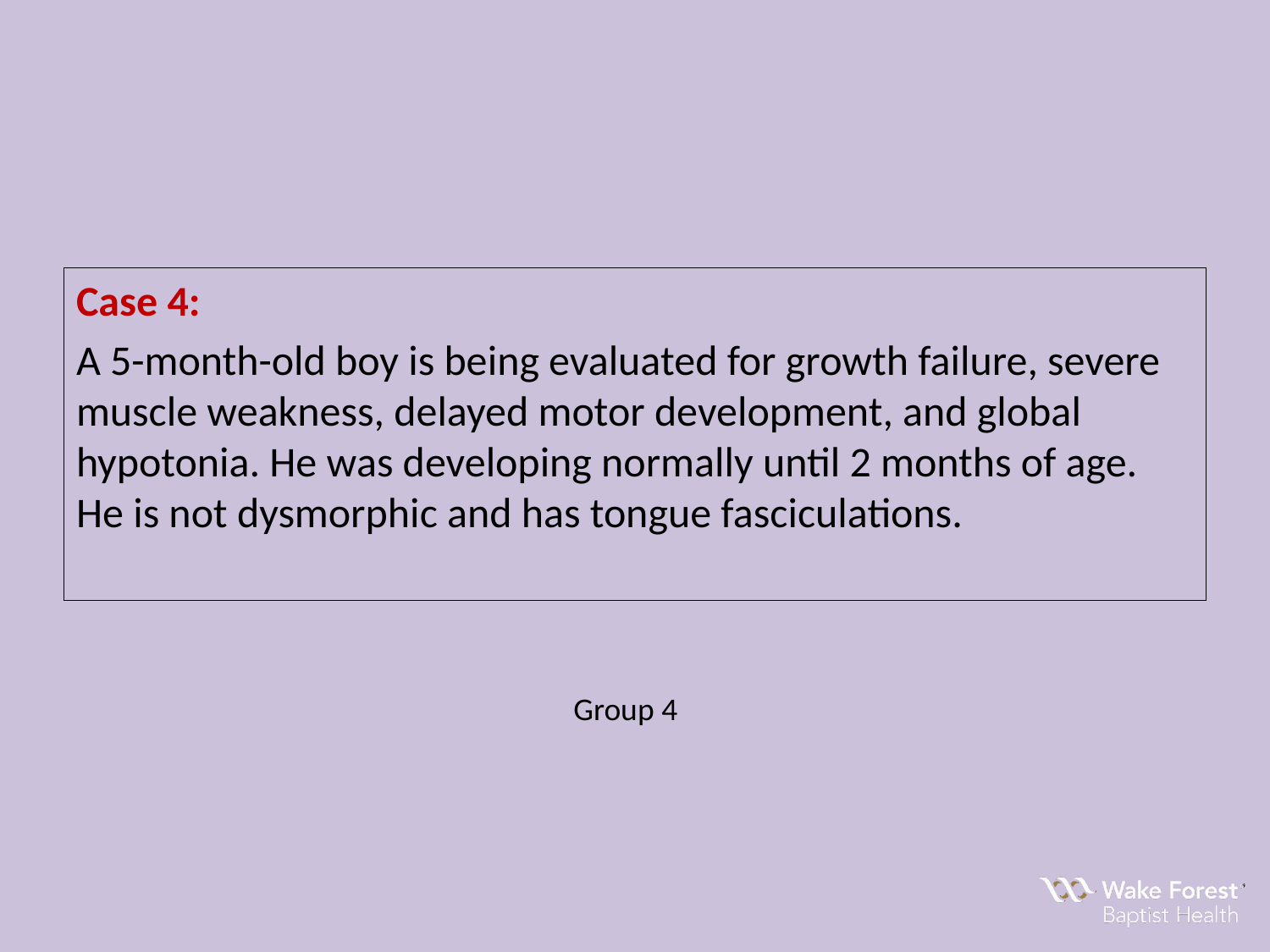

Case 4:
A 5-month-old boy is being evaluated for growth failure, severe muscle weakness, delayed motor development, and global hypotonia. He was developing normally until 2 months of age. He is not dysmorphic and has tongue fasciculations.
Group 4

## Slide 39
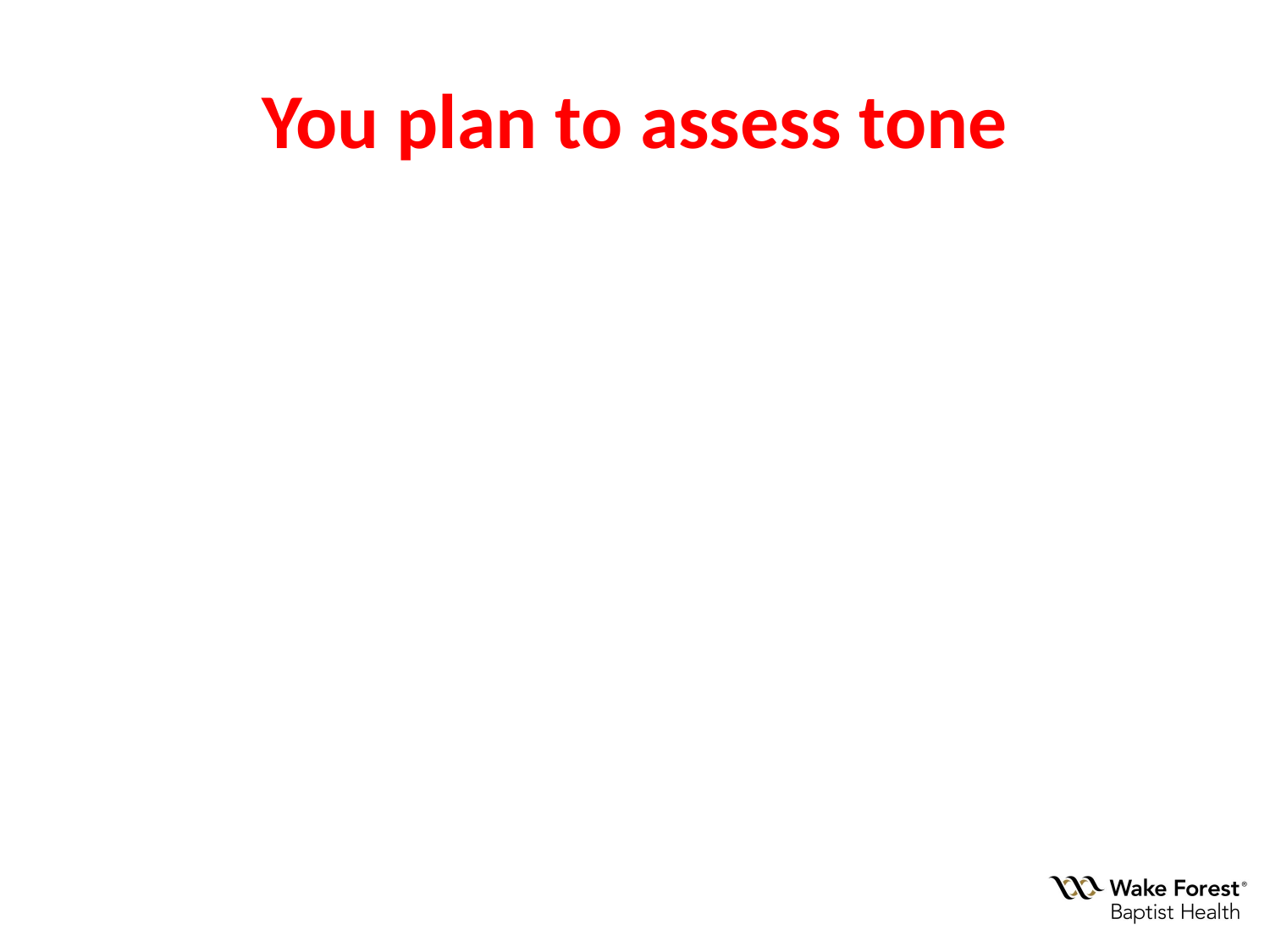

# You plan to assess tone

## Slide 40
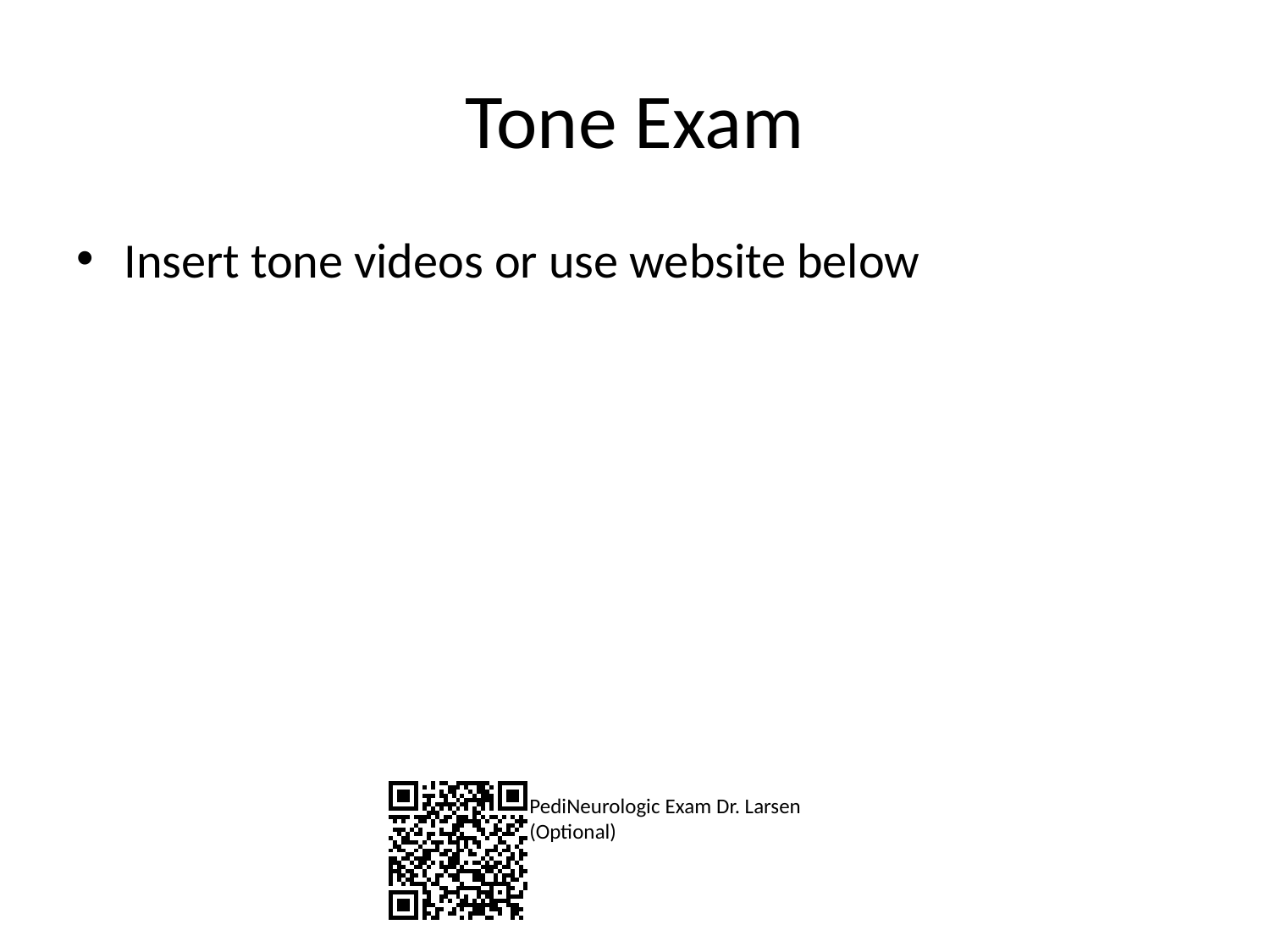

# Tone Exam
Insert tone videos or use website below
PediNeurologic Exam Dr. Larsen​​
(Optional)

## Slide 41
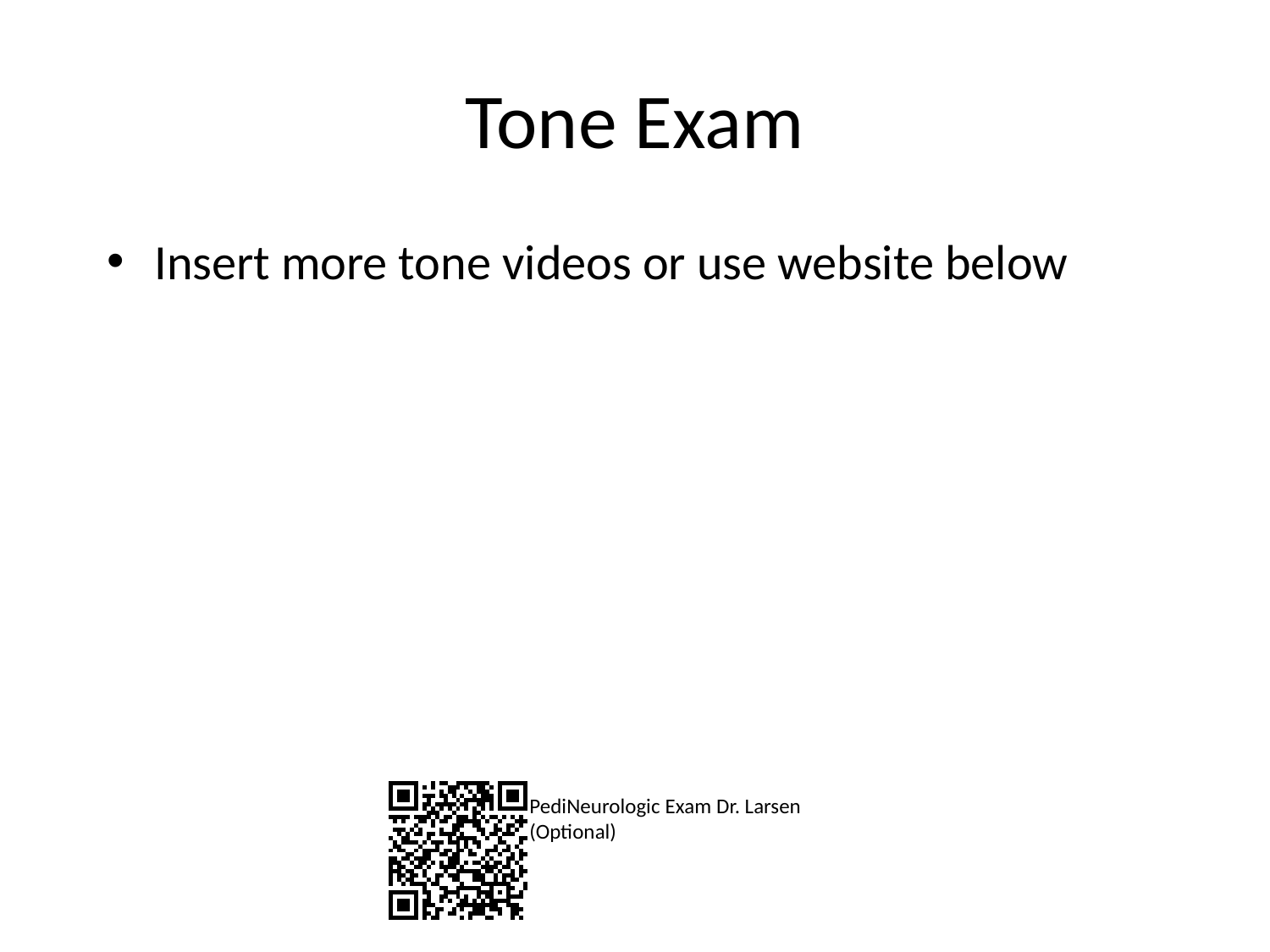

# Tone Exam
Insert more tone videos or use website below
PediNeurologic Exam Dr. Larsen​​​
(Optional)

## Slide 42
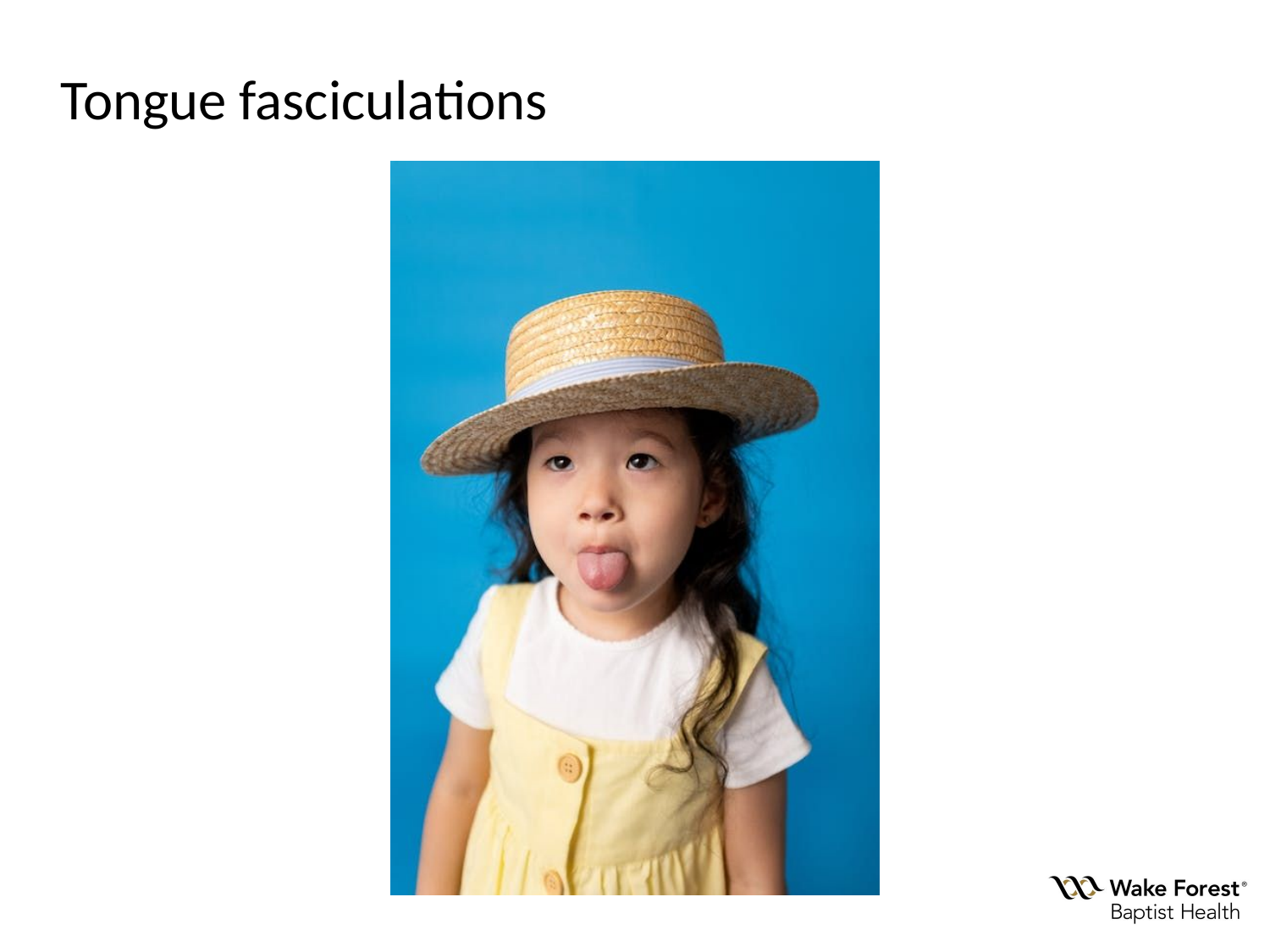

Tongue fasciculations

## Slide 43
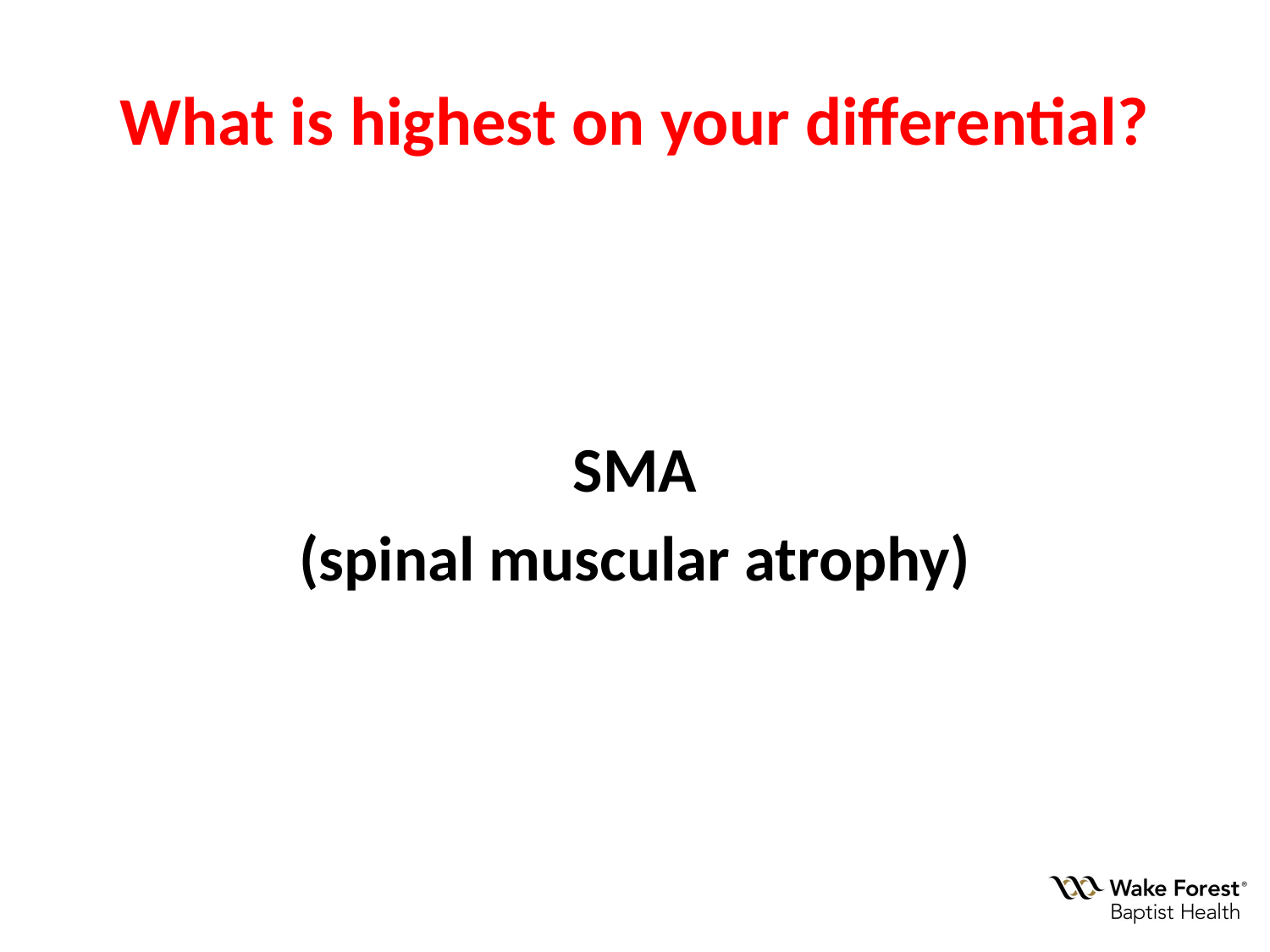

# What is highest on your differential?
SMA
(spinal muscular atrophy)

## Slide 44
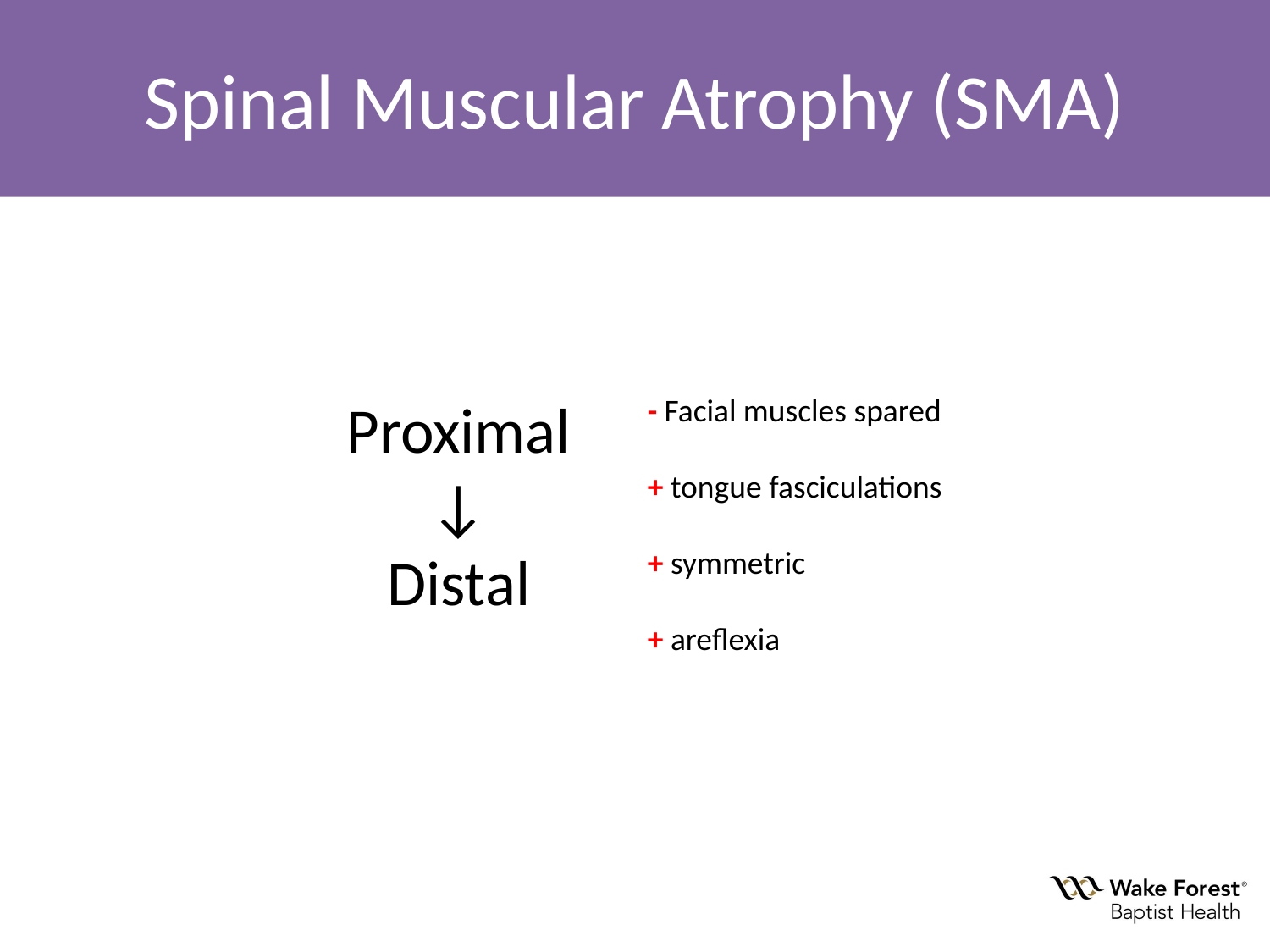

# Spinal Muscular Atrophy (SMA)
Proximal
↓
Distal
- Facial muscles spared
+ tongue fasciculations
+ symmetric
+ areflexia

## Slide 45
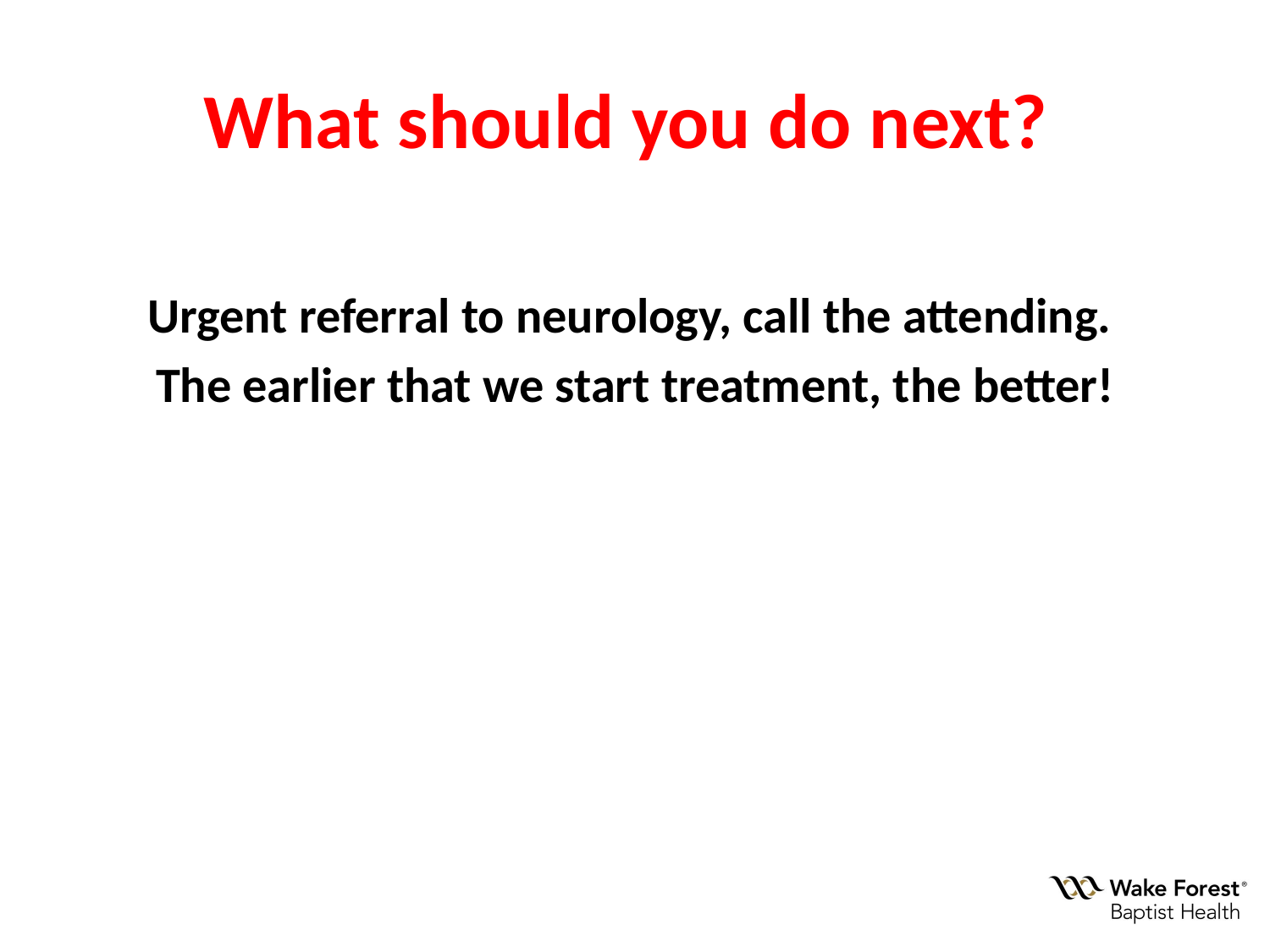

# What should you do next?
Urgent referral to neurology, call the attending.
The earlier that we start treatment, the better!

## Slide 46
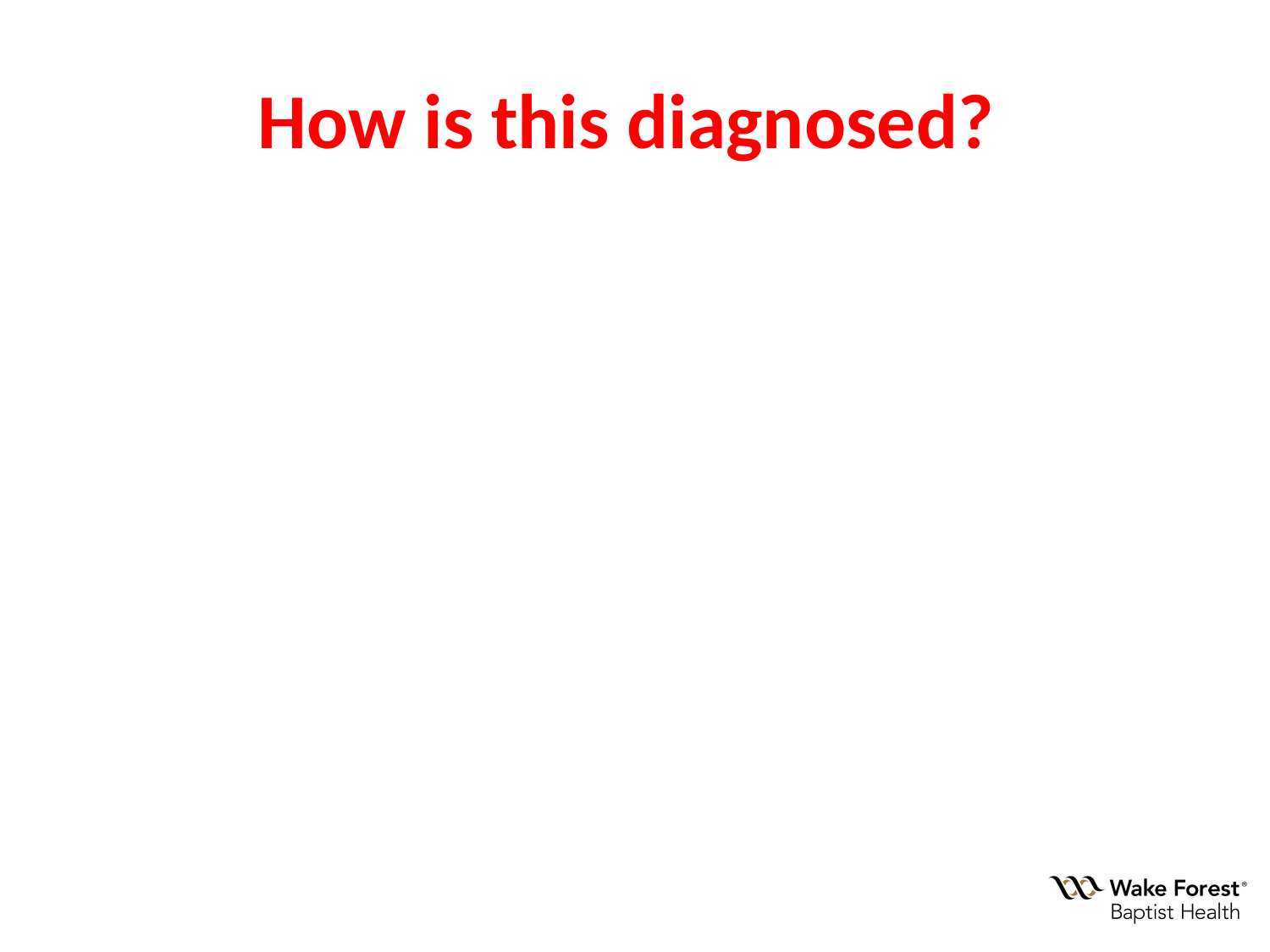

# How is this diagnosed?

## Slide 47
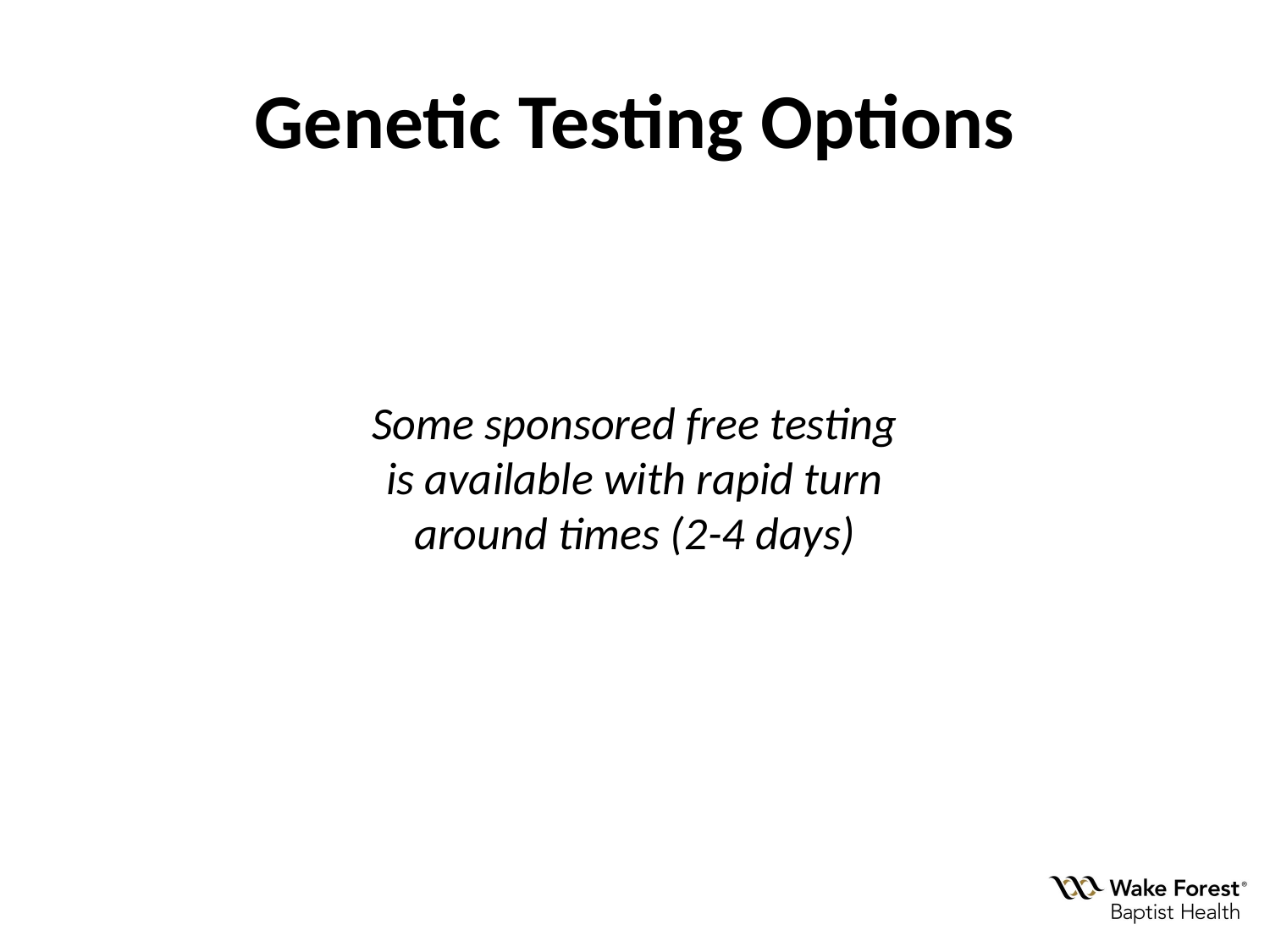

# Genetic Testing Options
Some sponsored free testing is available with rapid turn around times (2-4 days)

## Slide 48
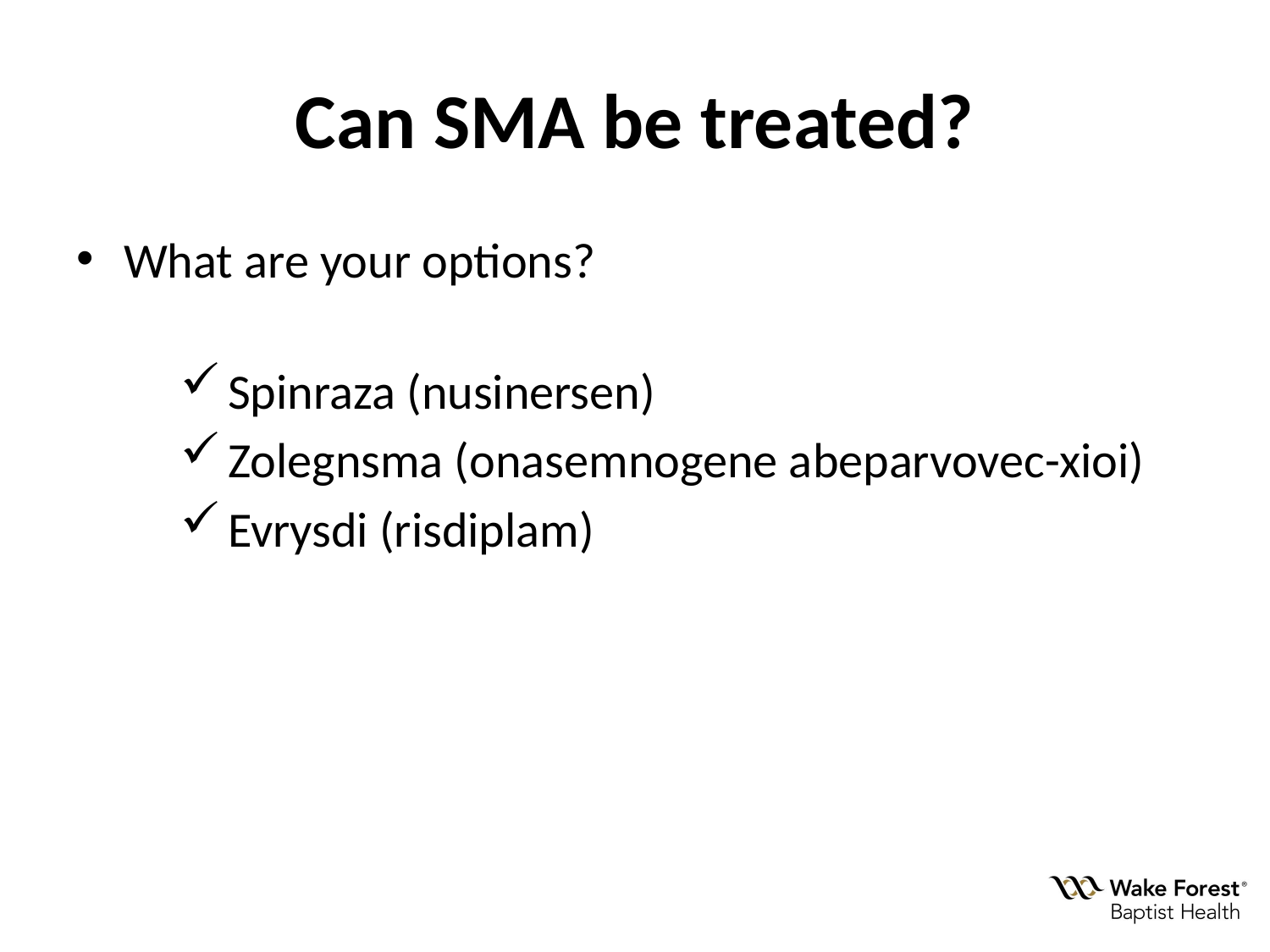

# Can SMA be treated?
What are your options?
Spinraza (nusinersen)
Zolegnsma (onasemnogene abeparvovec-xioi)
Evrysdi (risdiplam)

## Slide 49
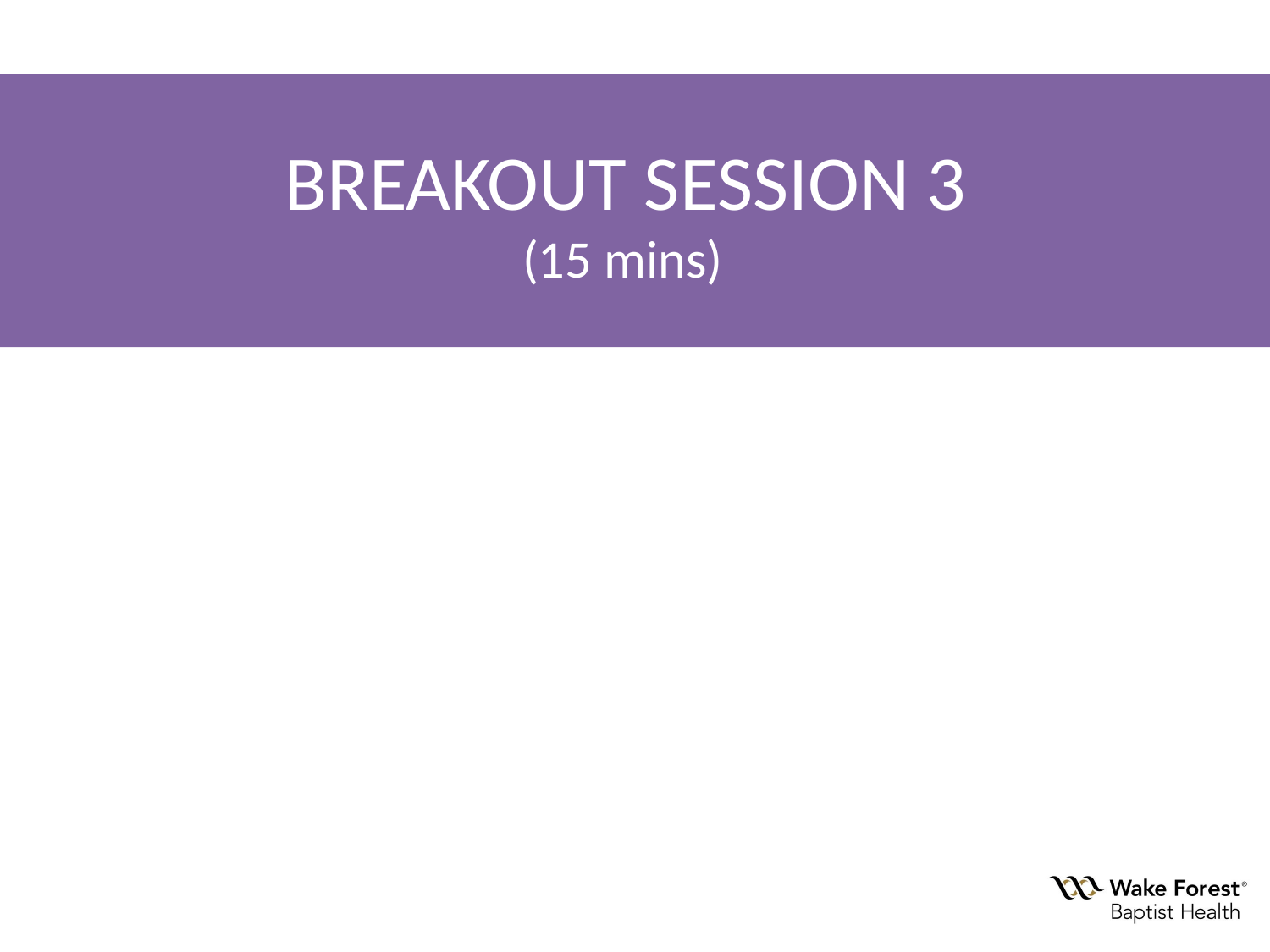

# BREAKOUT SESSION 3 (15 mins)

## Slide 50
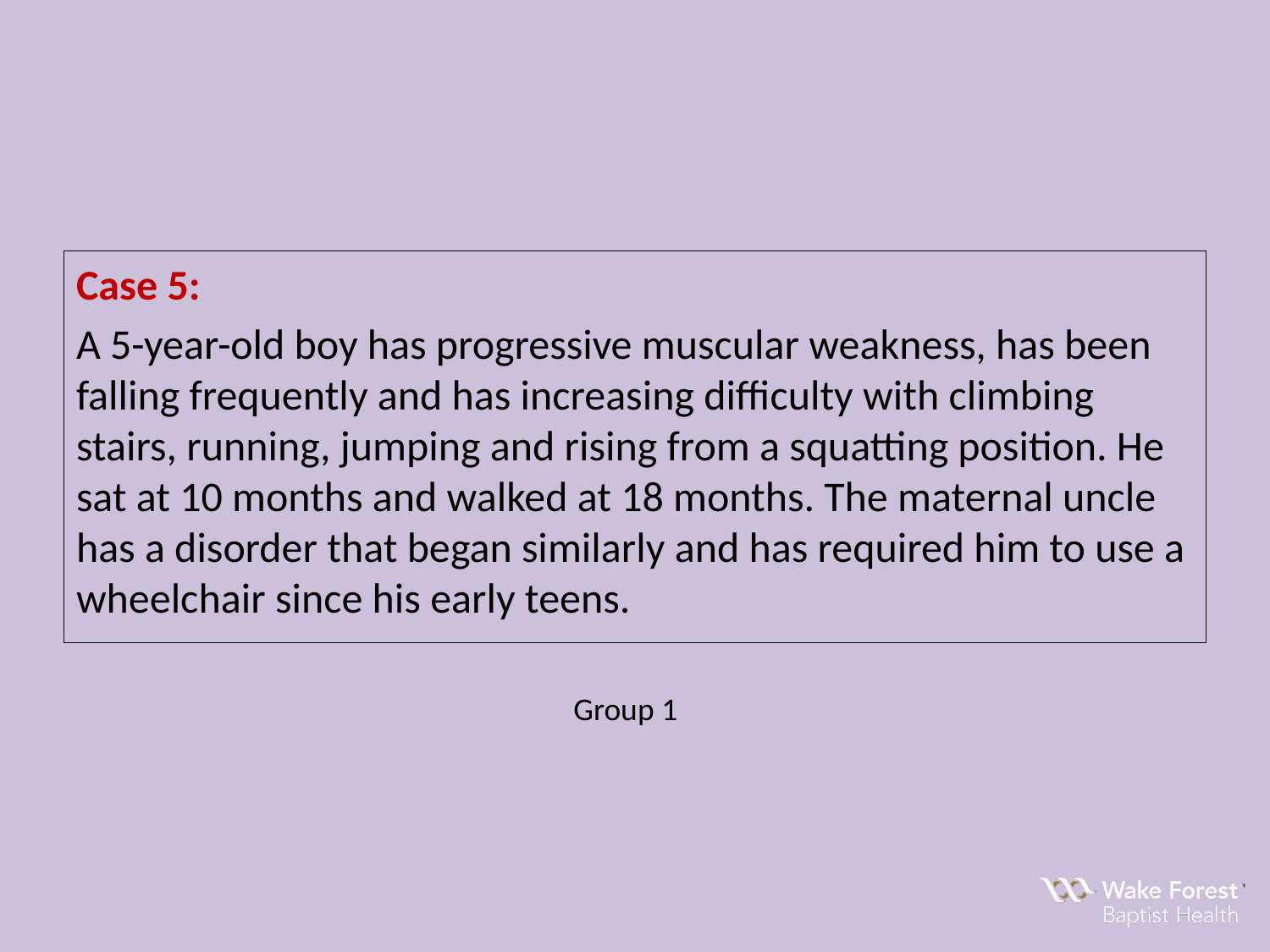

Case 5:
A 5-year-old boy has progressive muscular weakness, has been falling frequently and has increasing difficulty with climbing stairs, running, jumping and rising from a squatting position. He sat at 10 months and walked at 18 months. The maternal uncle has a disorder that began similarly and has required him to use a wheelchair since his early teens.
Group 1

## Slide 51
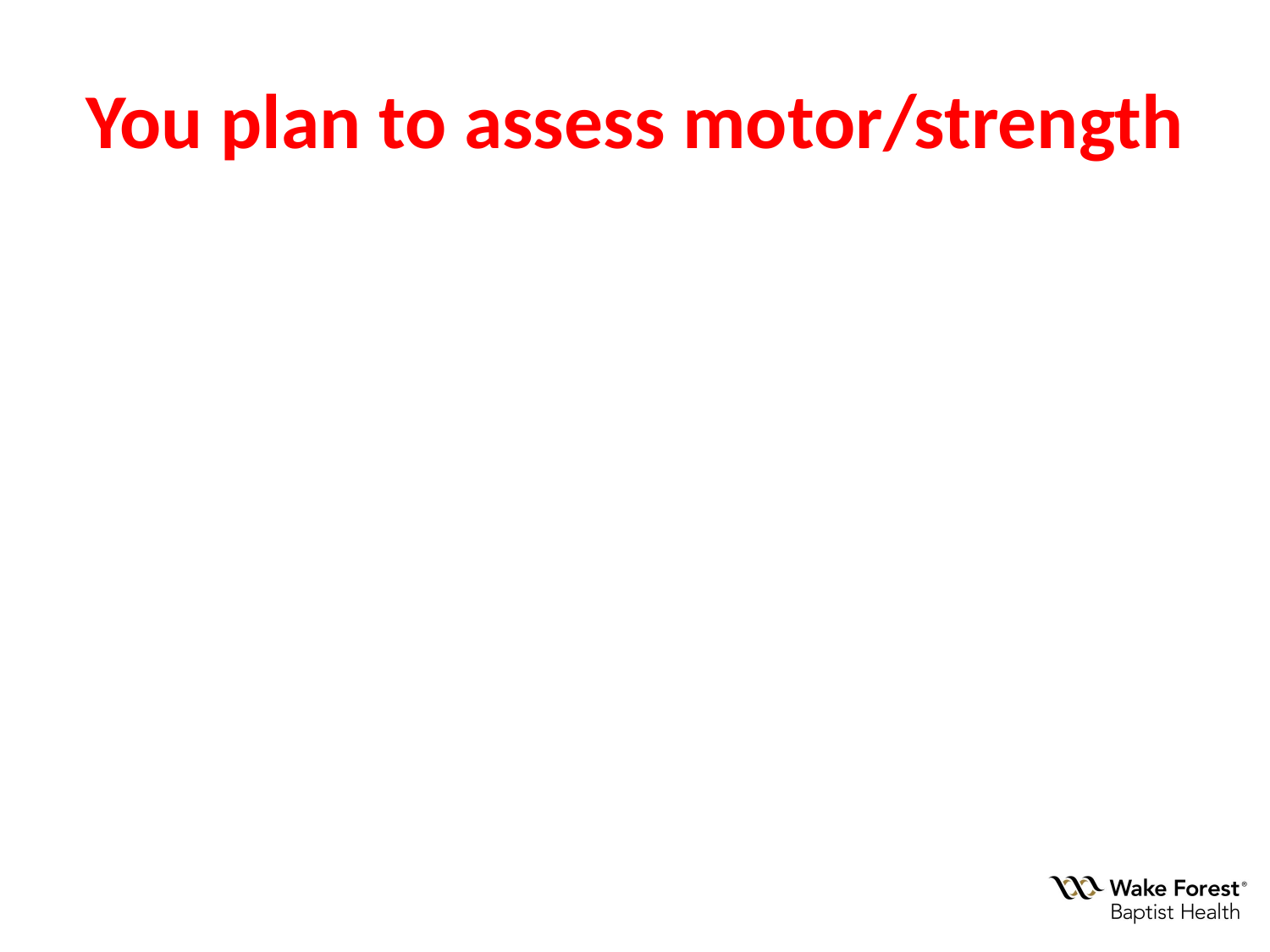

# You plan to assess motor/strength

## Slide 52
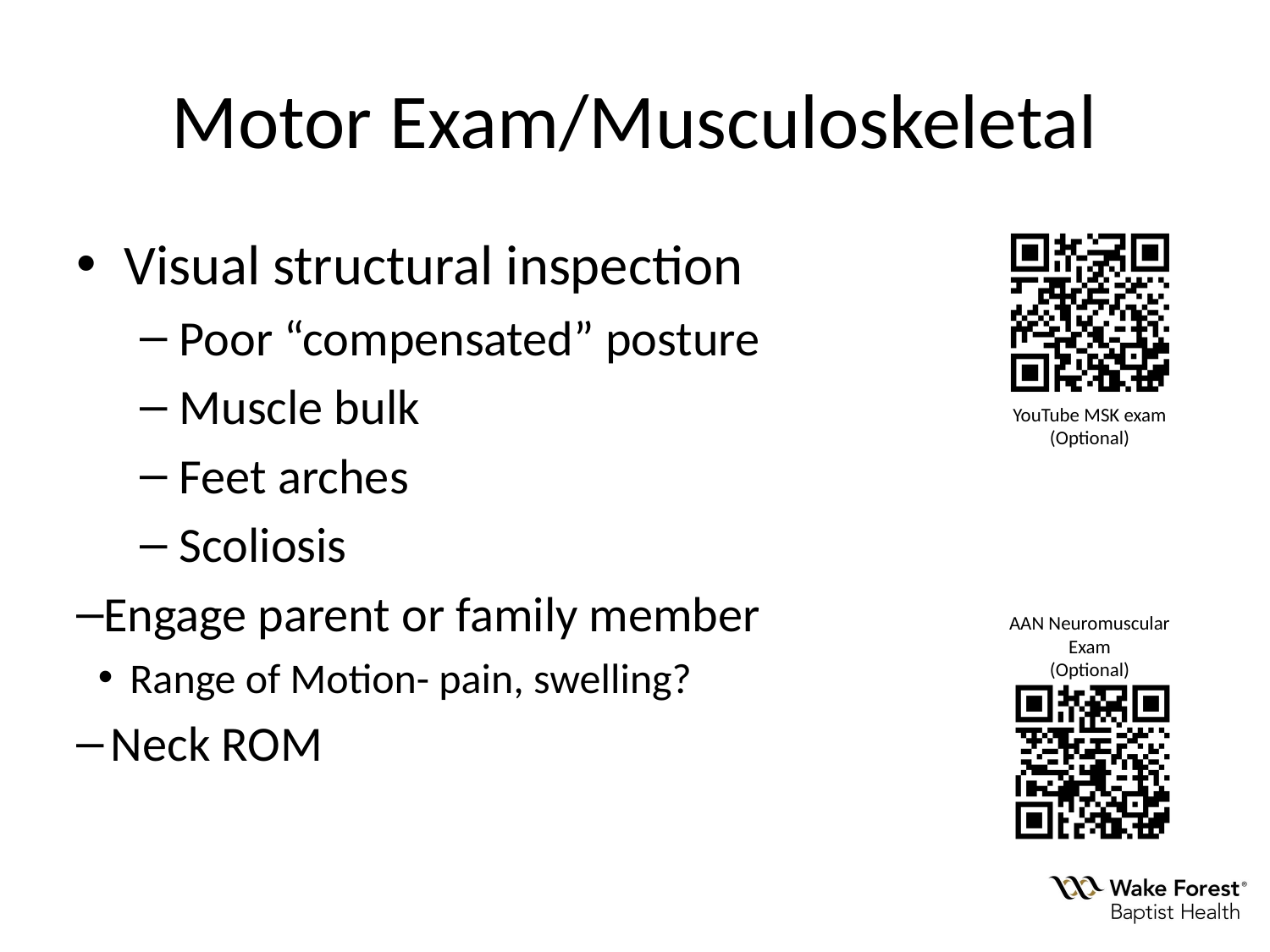

# Motor Exam/Musculoskeletal
Visual structural inspection
Poor “compensated” posture
Muscle bulk
Feet arches
Scoliosis
Engage parent or family member
Range of Motion- pain, swelling?
Neck ROM
YouTube MSK exam
(Optional)
AAN Neuromuscular Exam
(Optional)

## Slide 53
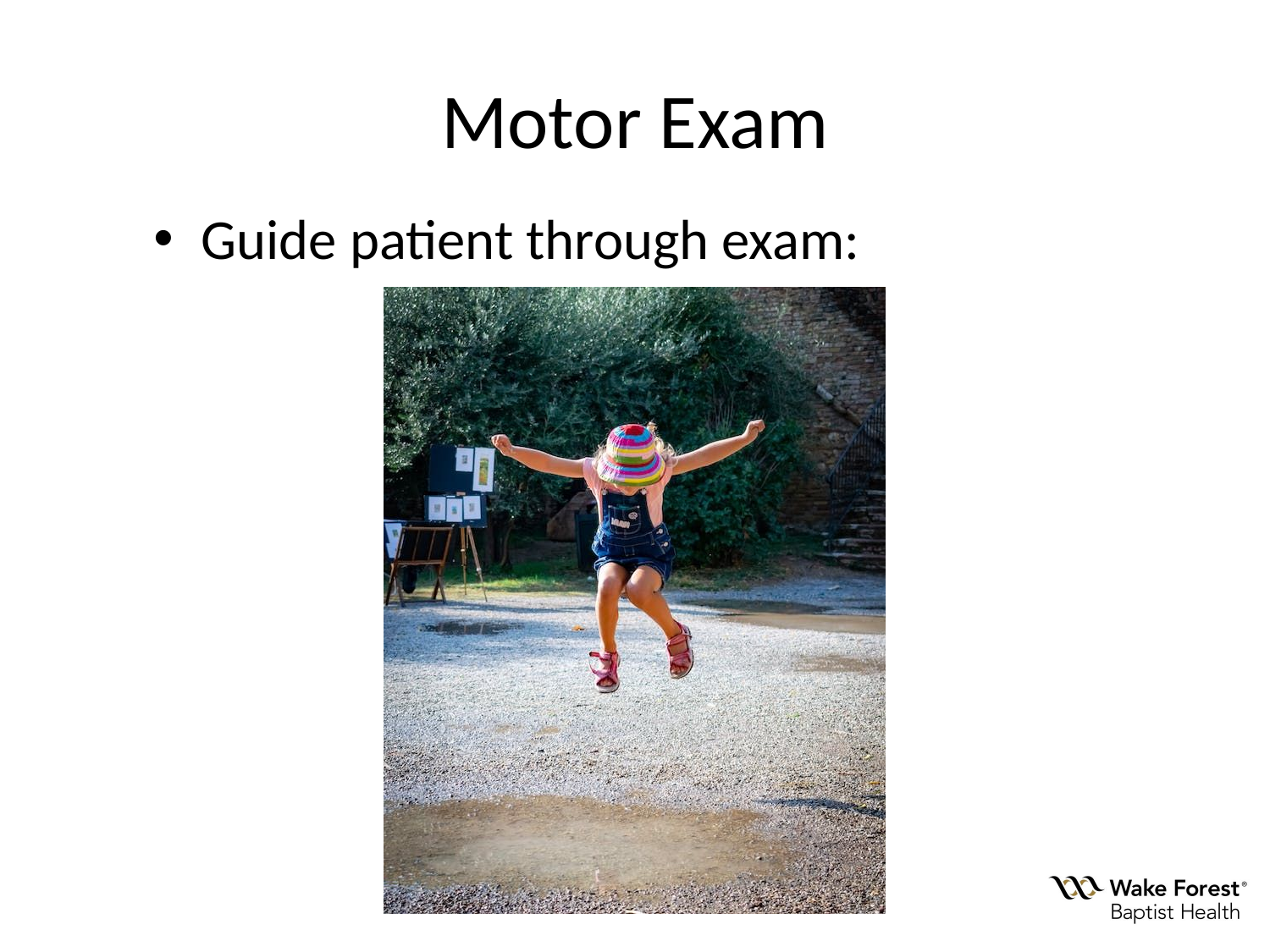

# Motor Exam
Guide patient through exam:

## Slide 54
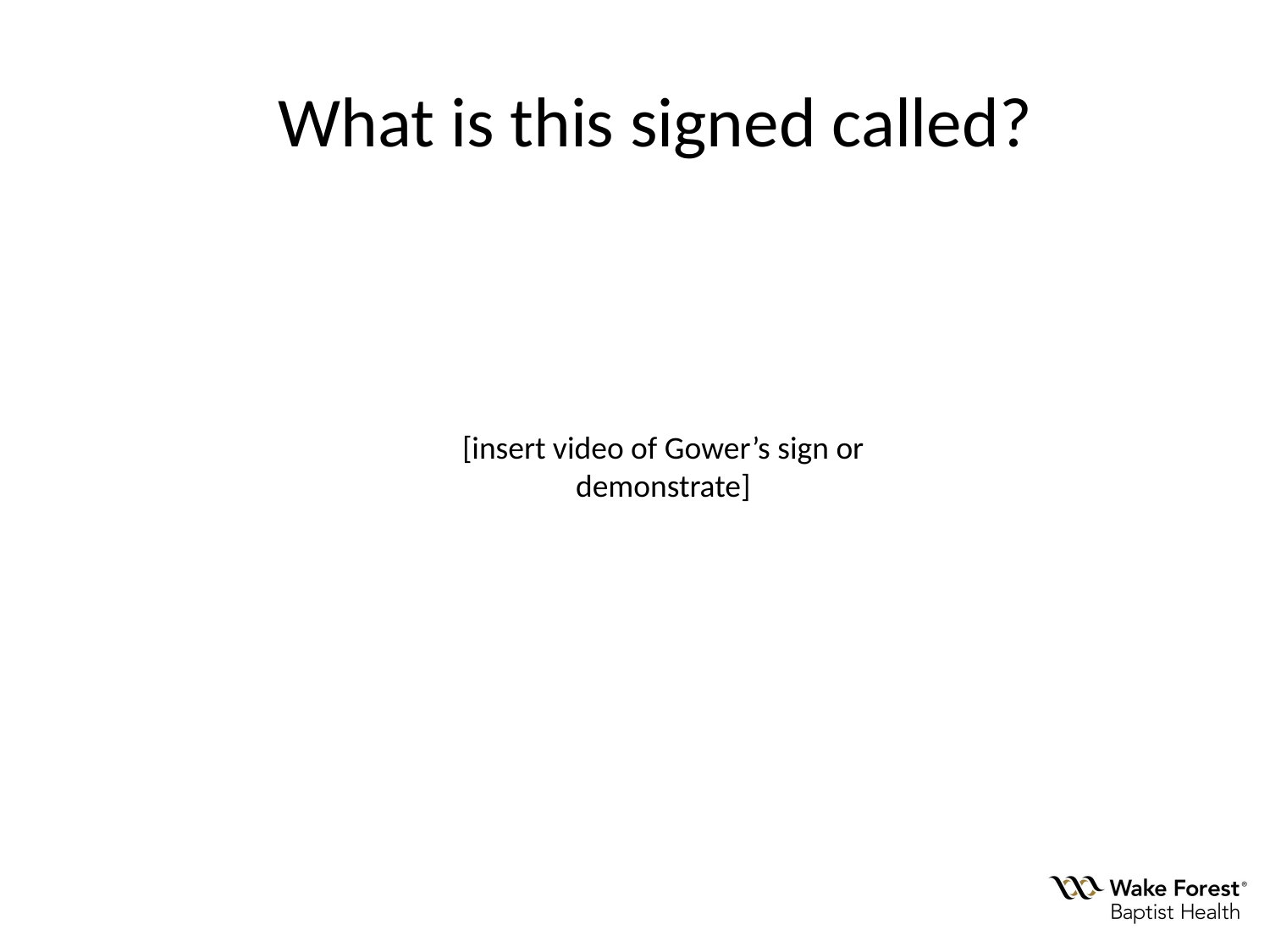

What is this signed called?
[insert video of Gower’s sign or demonstrate]

## Slide 55
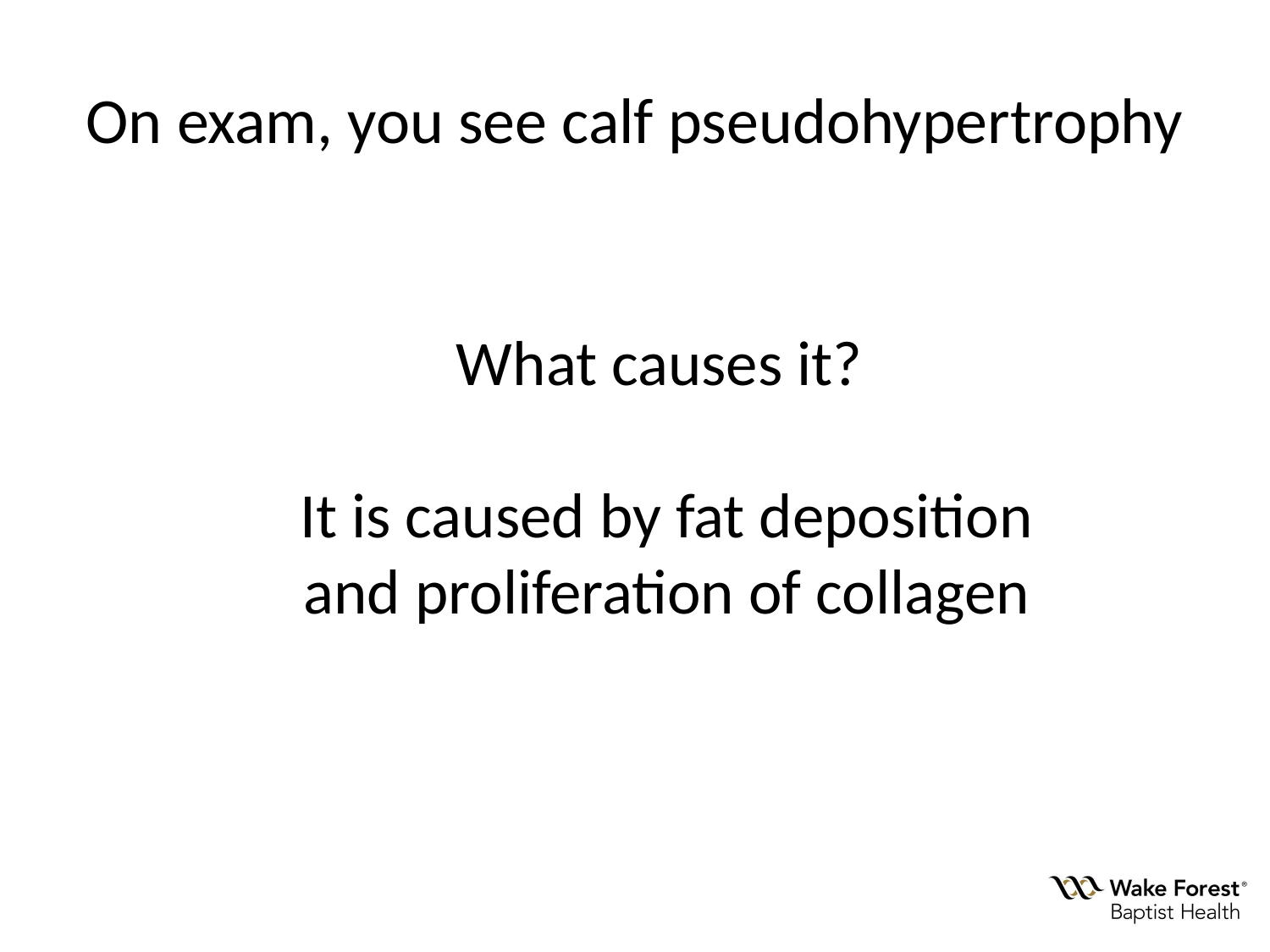

# On exam, you see calf pseudohypertrophy
What causes it?
It is caused by fat deposition and proliferation of collagen

## Slide 56
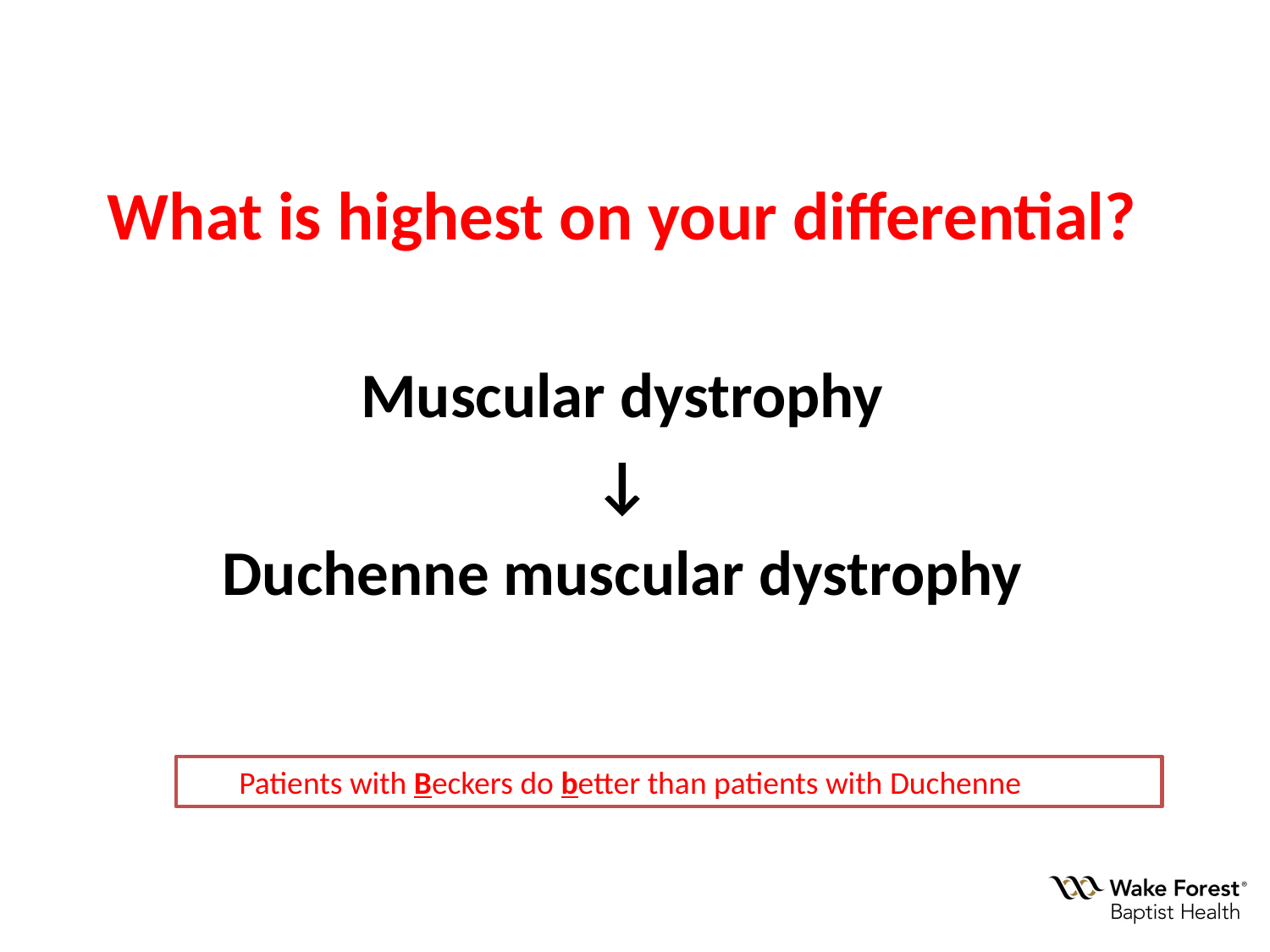

# What is highest on your differential?
Muscular dystrophy
↓
Duchenne muscular dystrophy
 Patients with Beckers do better than patients with Duchenne

## Slide 57
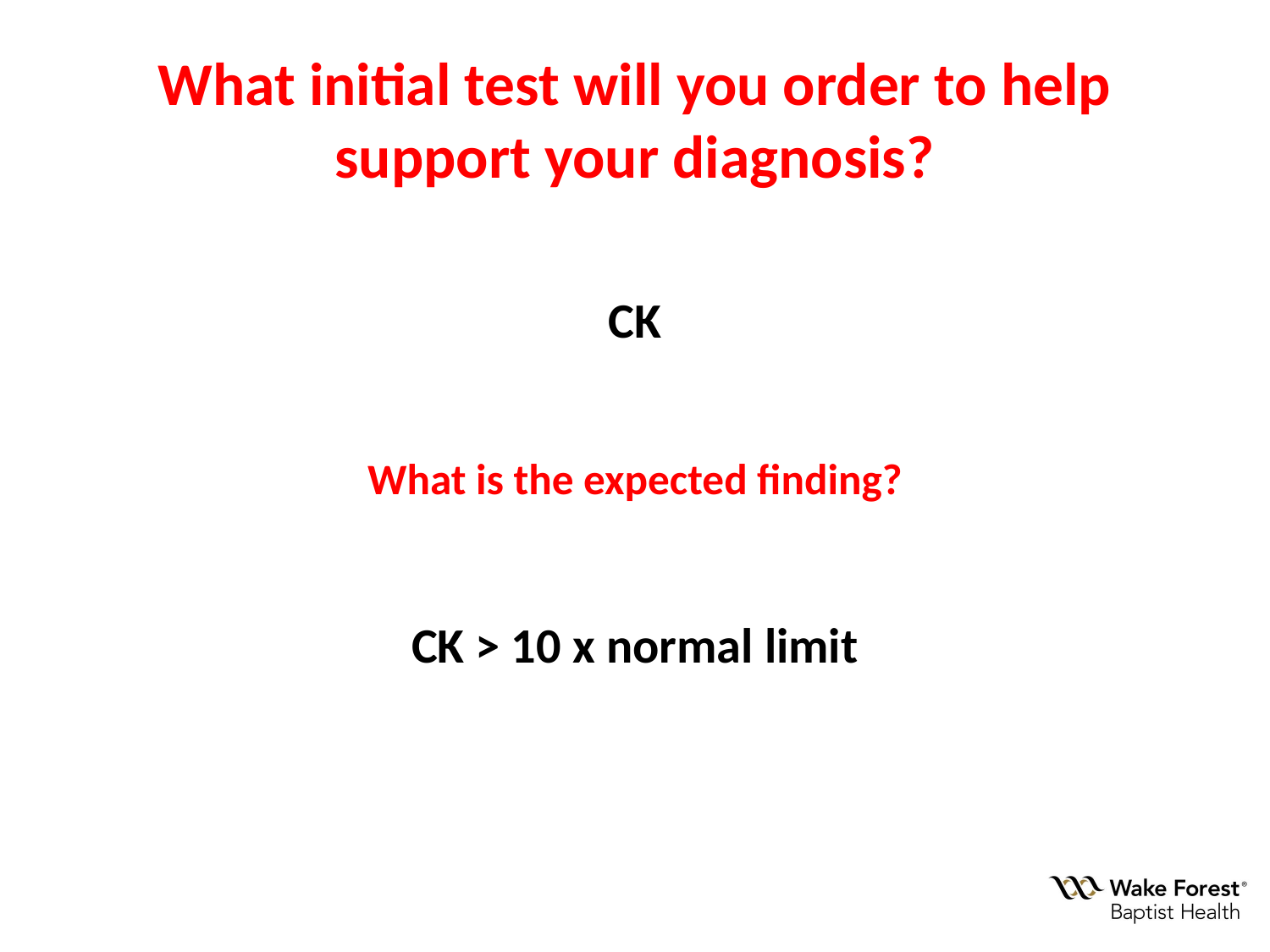

# What initial test will you order to help support your diagnosis?
CK
What is the expected finding?
CK > 10 x normal limit

## Slide 58
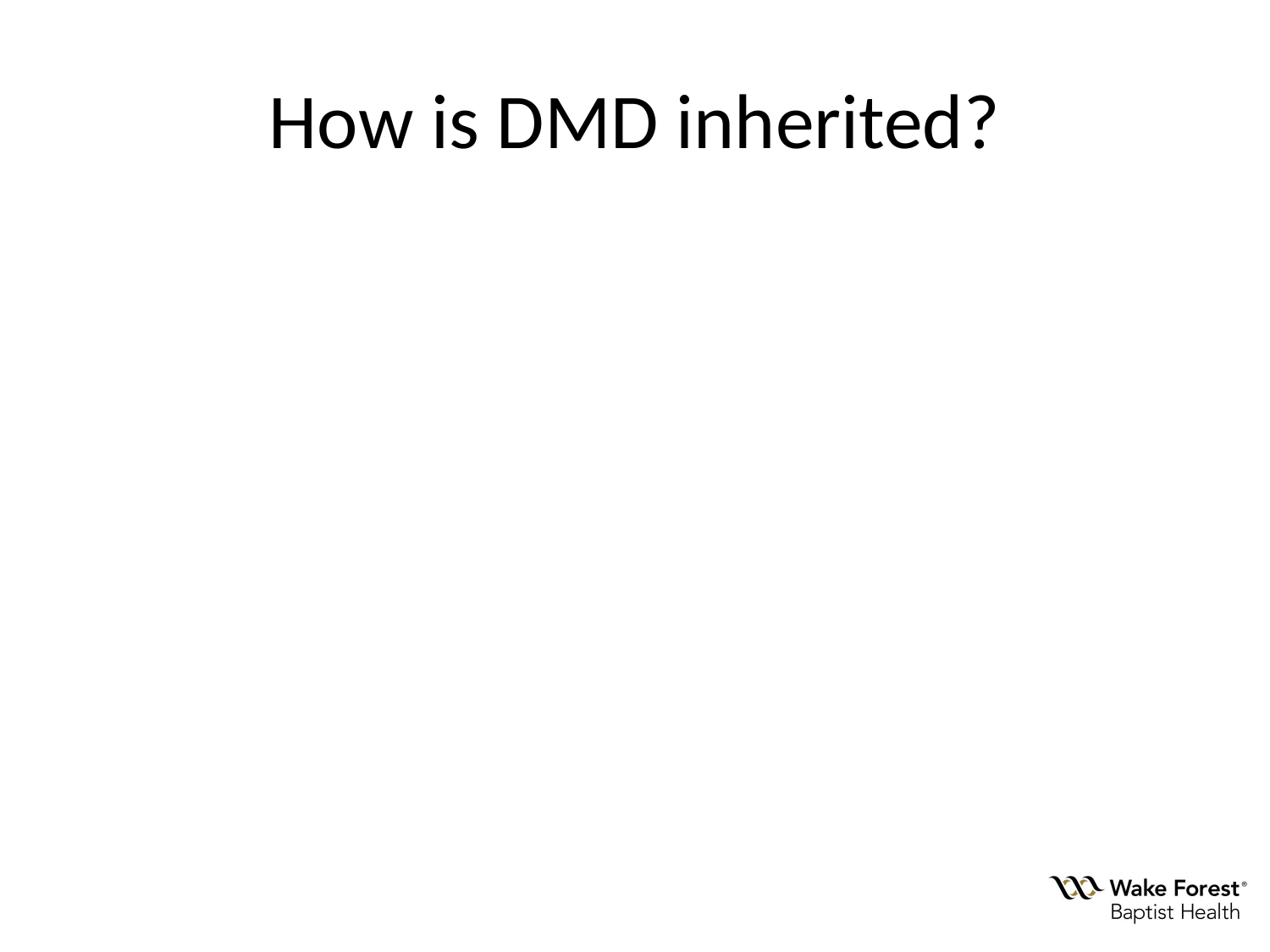

# How is DMD inherited?

## Slide 59
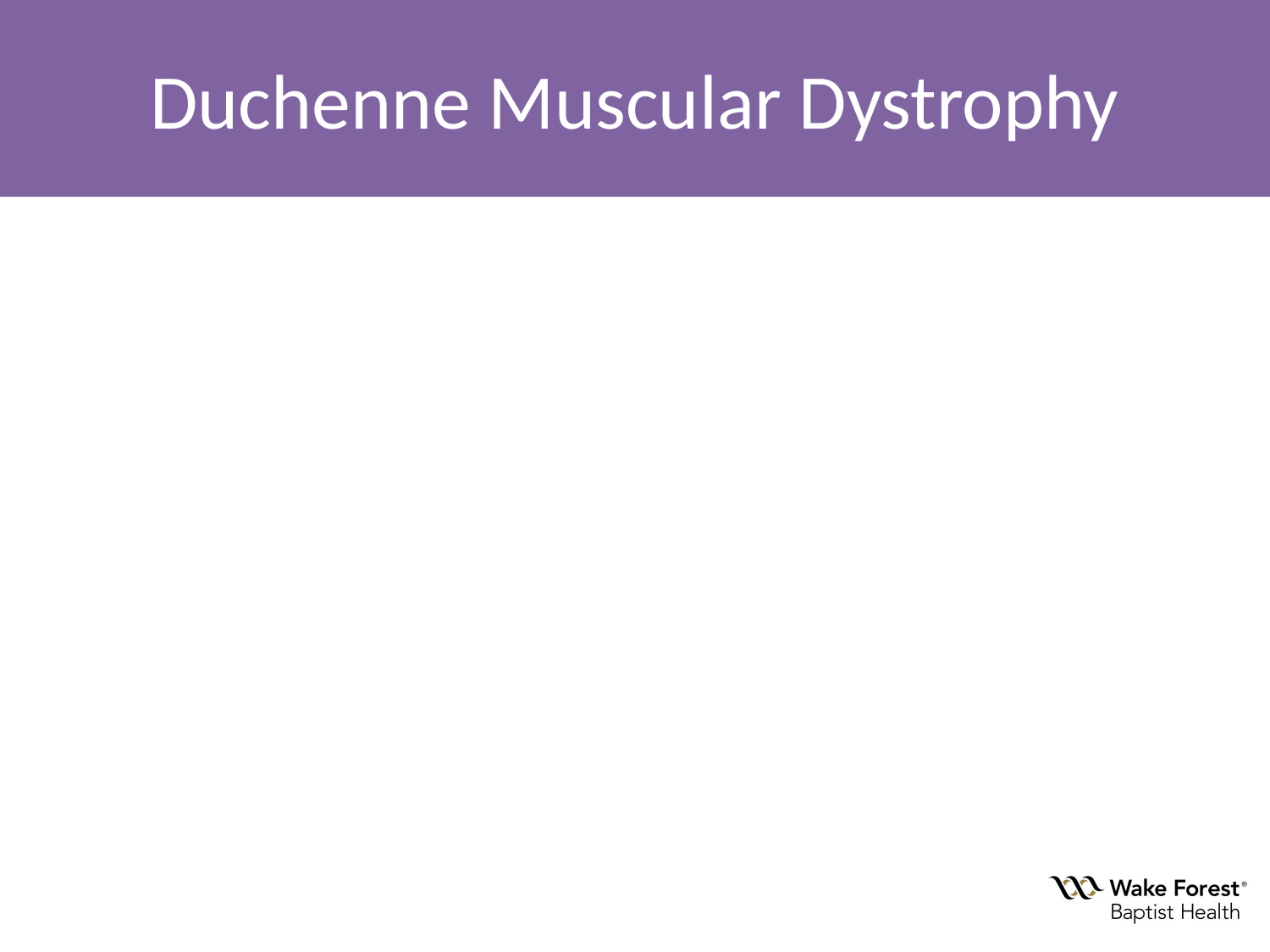

# Duchenne Muscular Dystrophy
,

## Slide 60
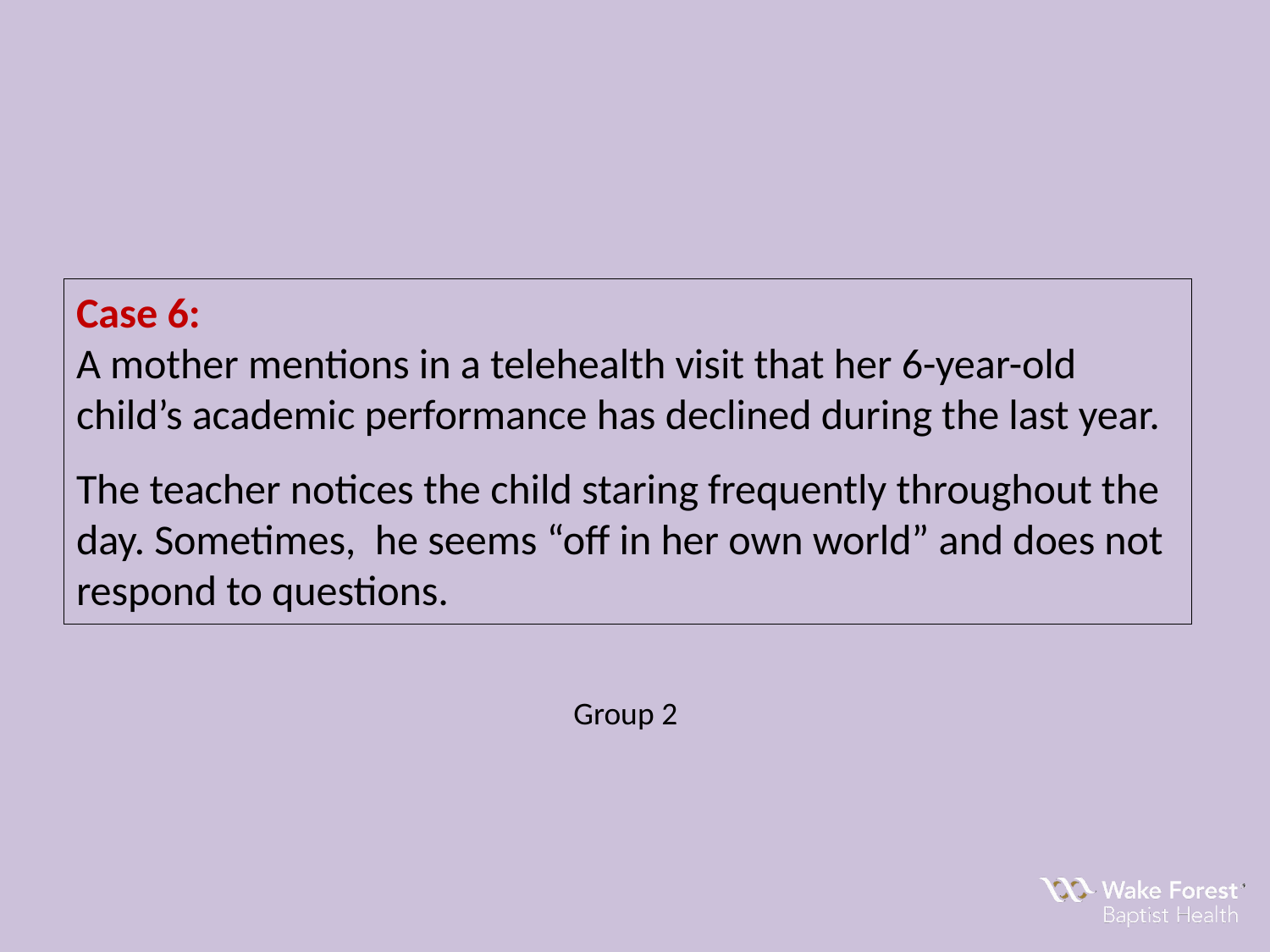

Case 6:
A mother mentions in a telehealth visit that her 6-year-old child’s academic performance has declined during the last year.
The teacher notices the child staring frequently throughout the day. Sometimes, he seems “off in her own world” and does not respond to questions.
Group 2

## Slide 61
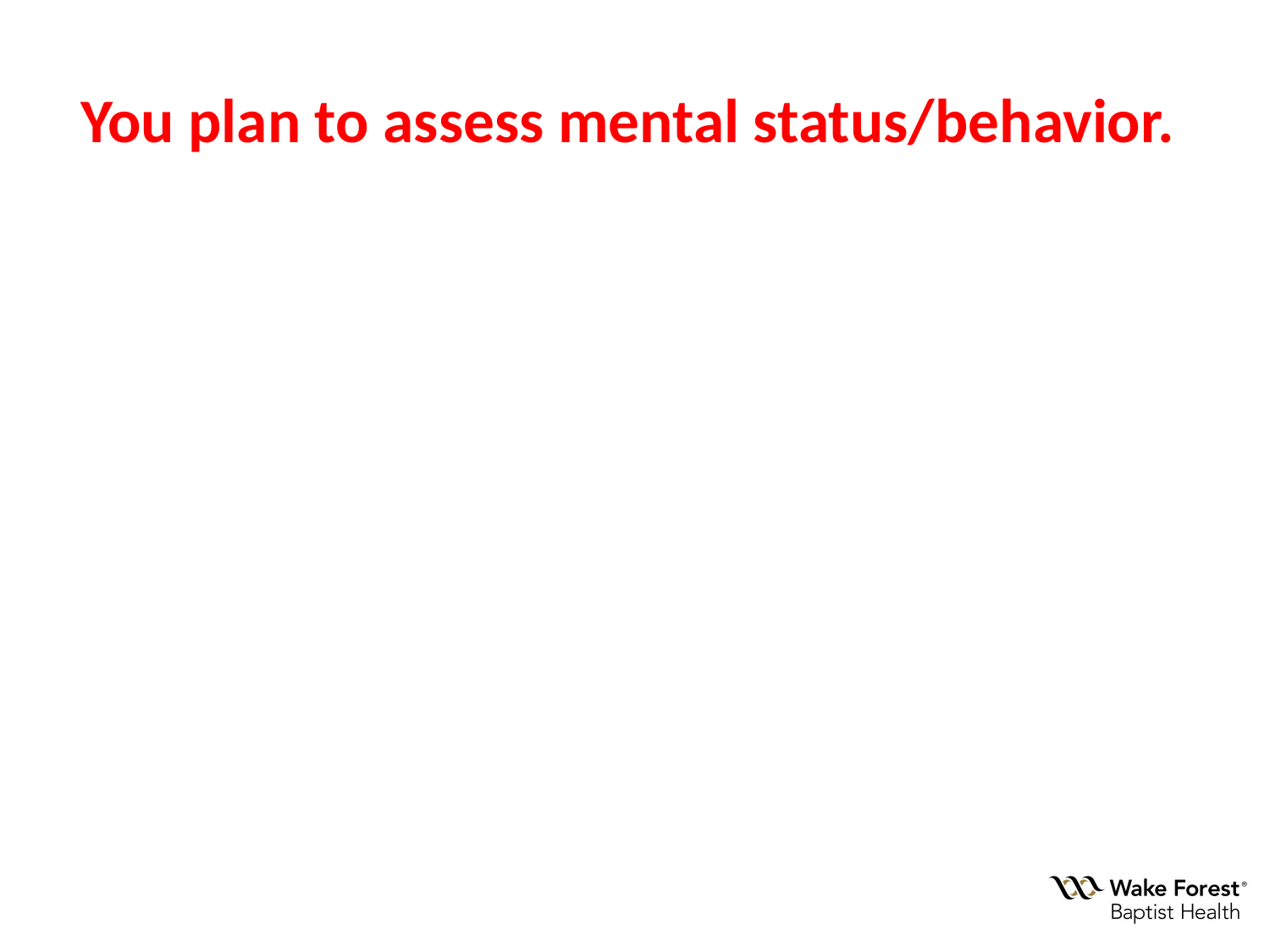

# You plan to assess mental status/behavior.

## Slide 62
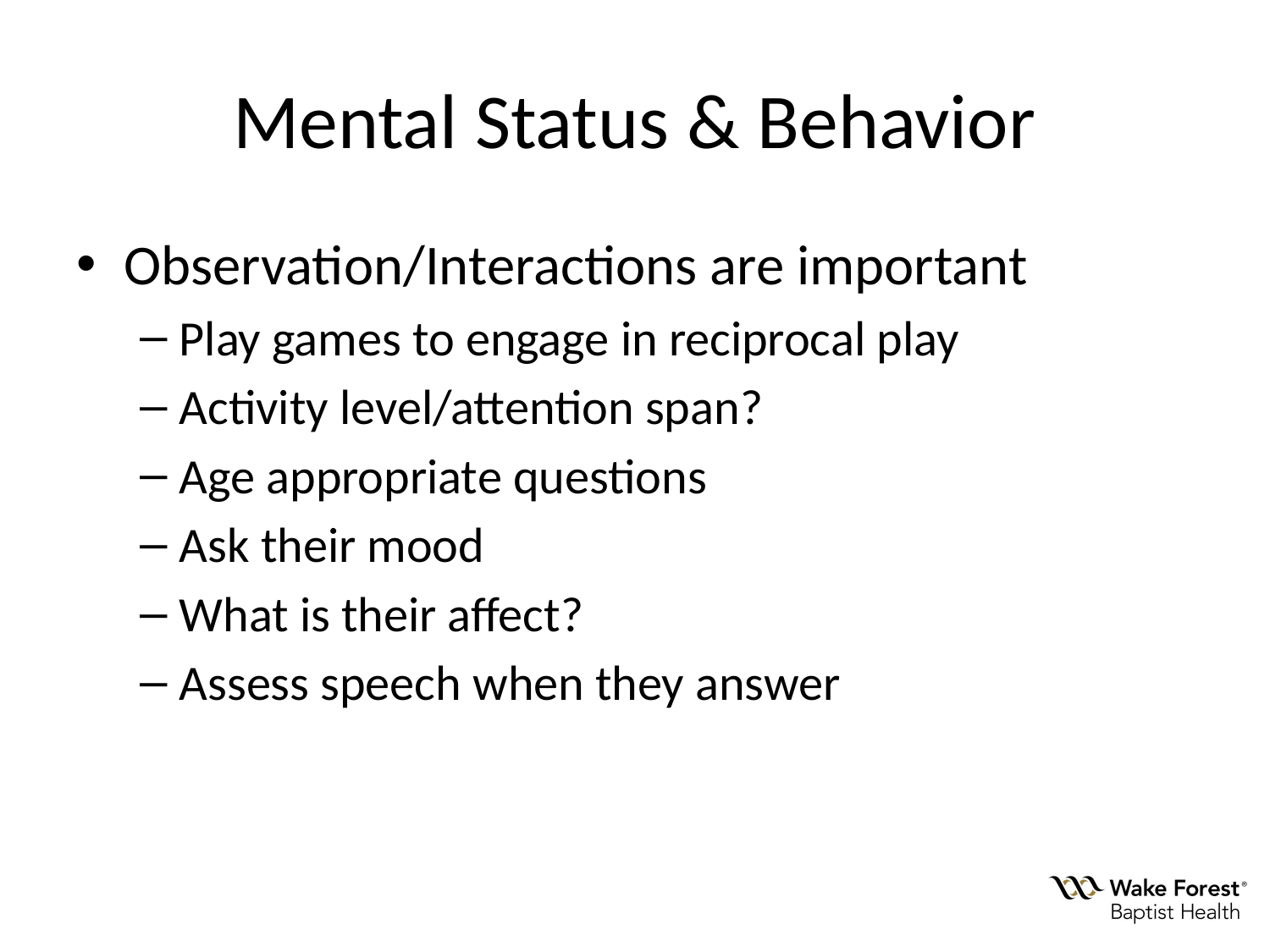

# Mental Status & Behavior
Observation/Interactions are important
Play games to engage in reciprocal play
Activity level/attention span?
Age appropriate questions
Ask their mood
What is their affect?
Assess speech when they answer

## Slide 63
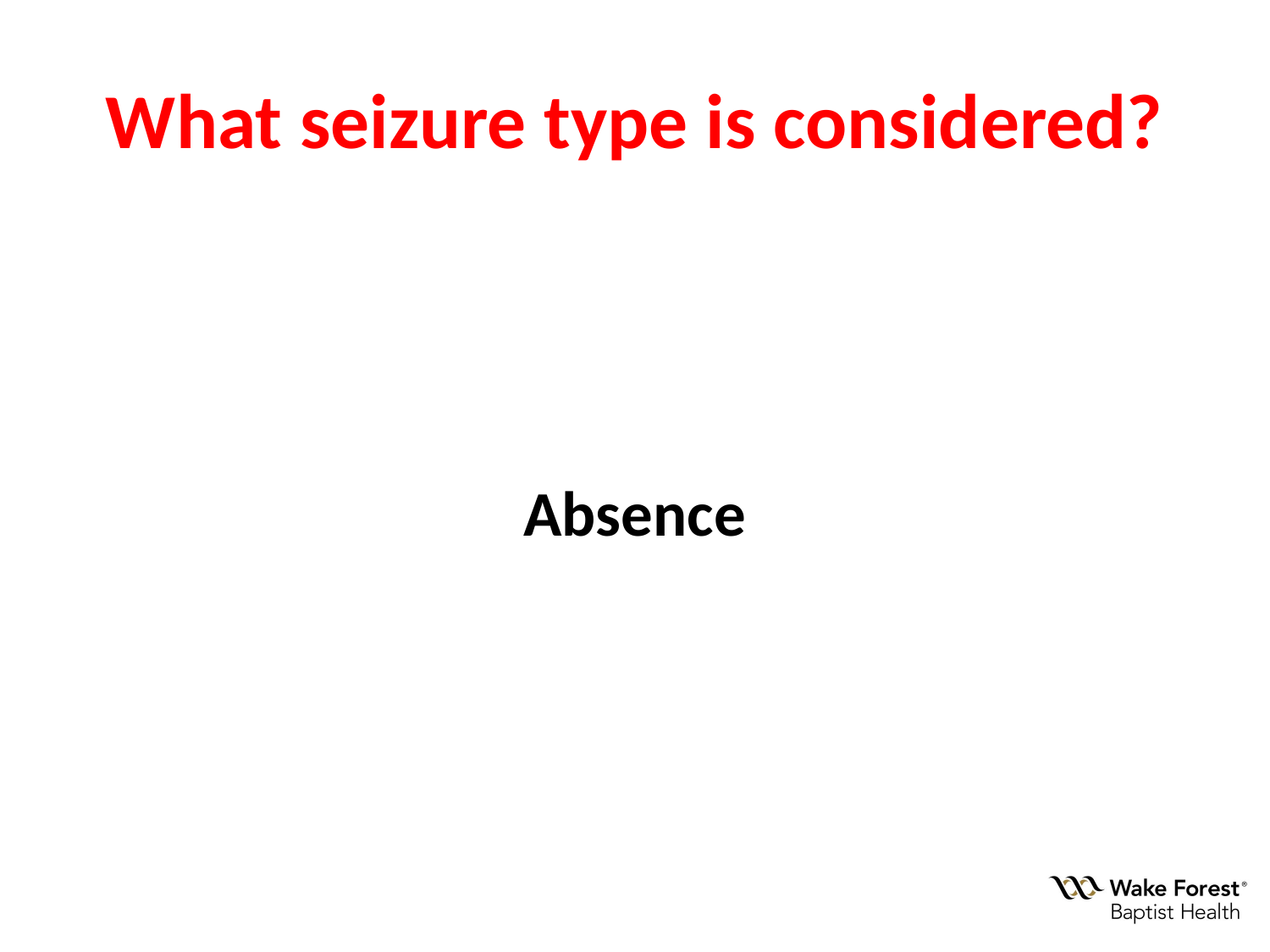

# What seizure type is considered?
Absence

## Slide 64
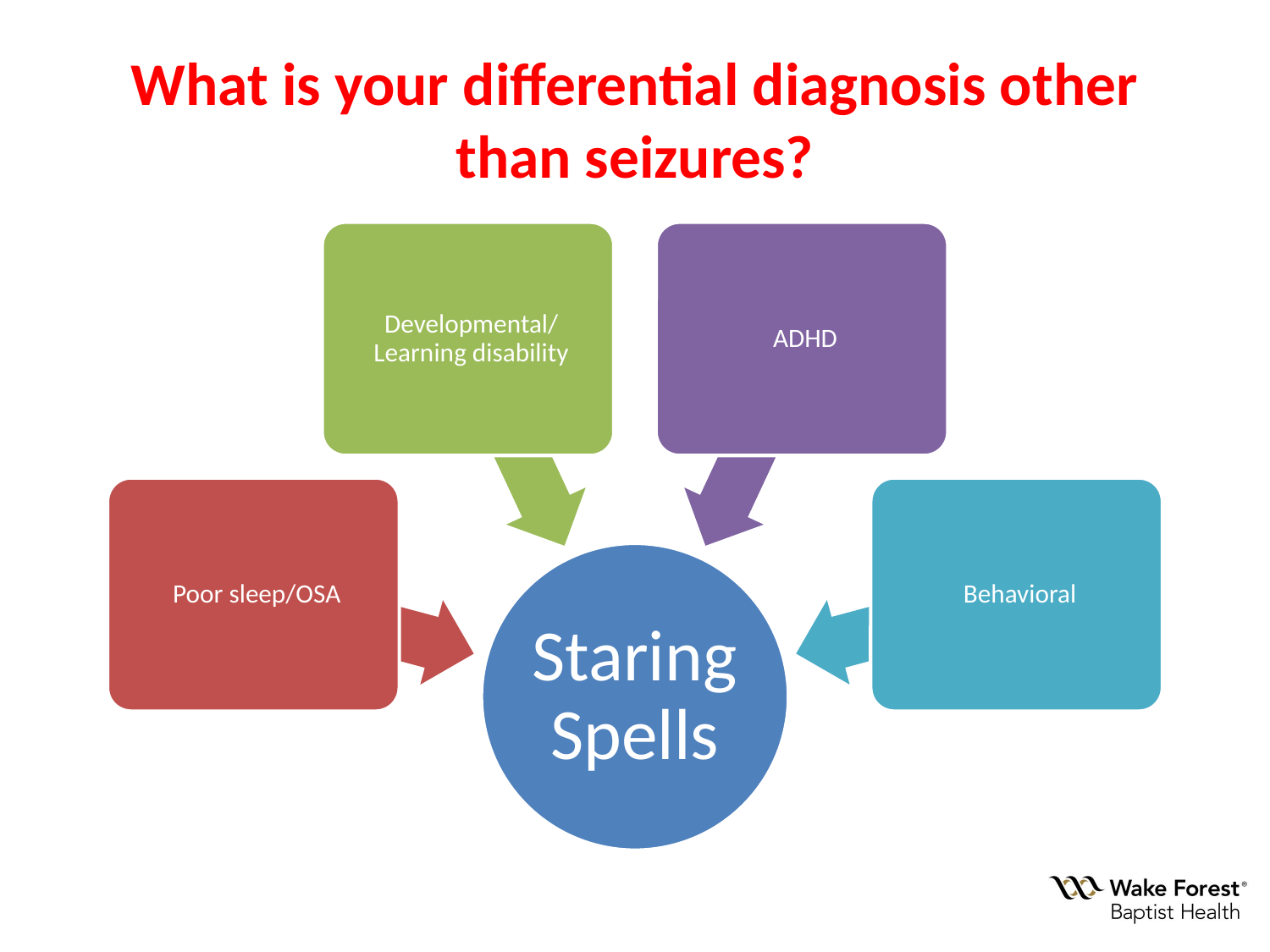

# What is your differential diagnosis other than seizures?

## Slide 65
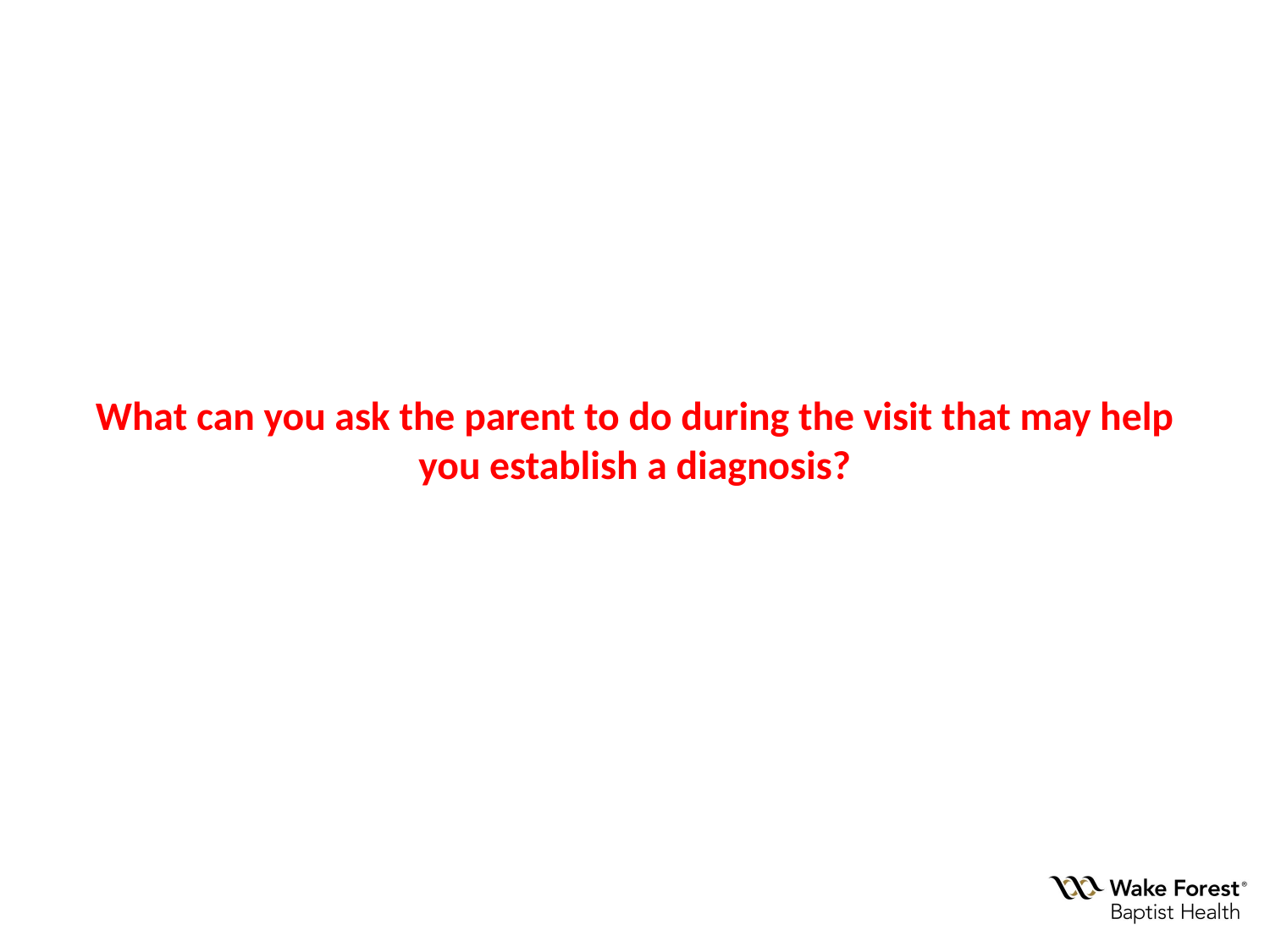

# What can you ask the parent to do during the visit that may help you establish a diagnosis?

## Slide 66
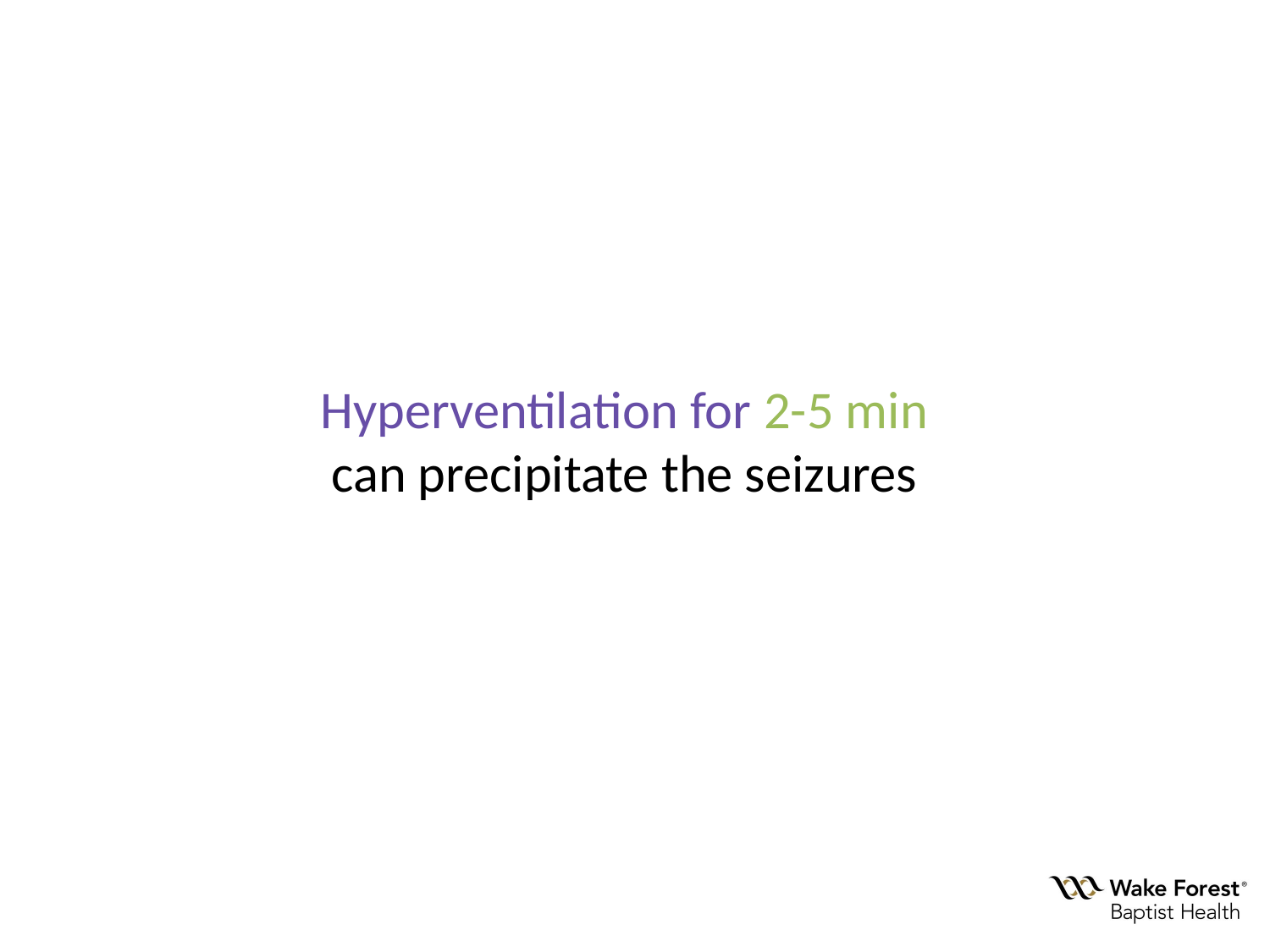

#
Hyperventilation for 2-5 min can precipitate the seizures

## Slide 67
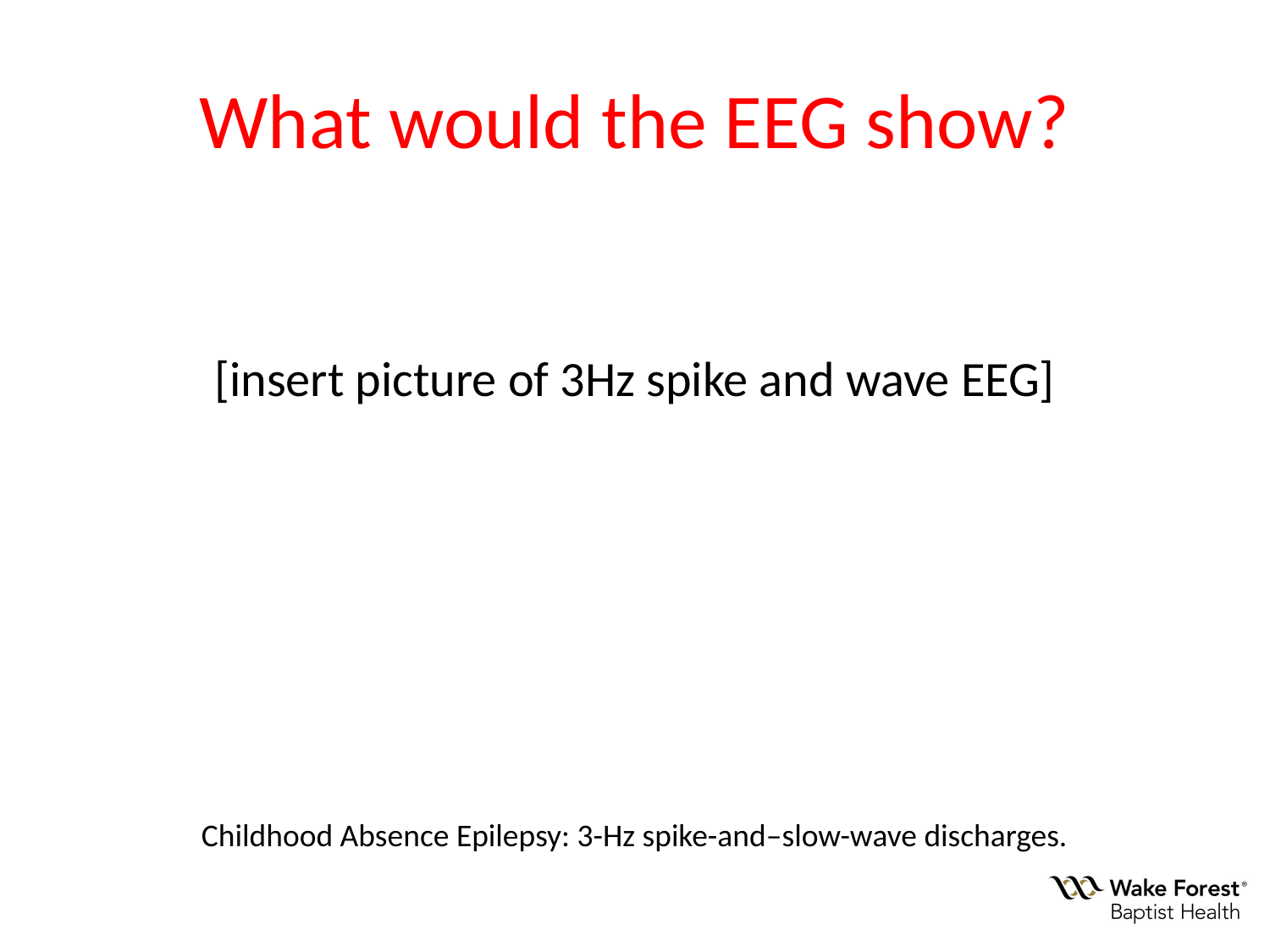

# What would the EEG show?
[insert picture of 3Hz spike and wave EEG]
Childhood Absence Epilepsy: 3-Hz spike-and–slow-wave discharges.

## Slide 68
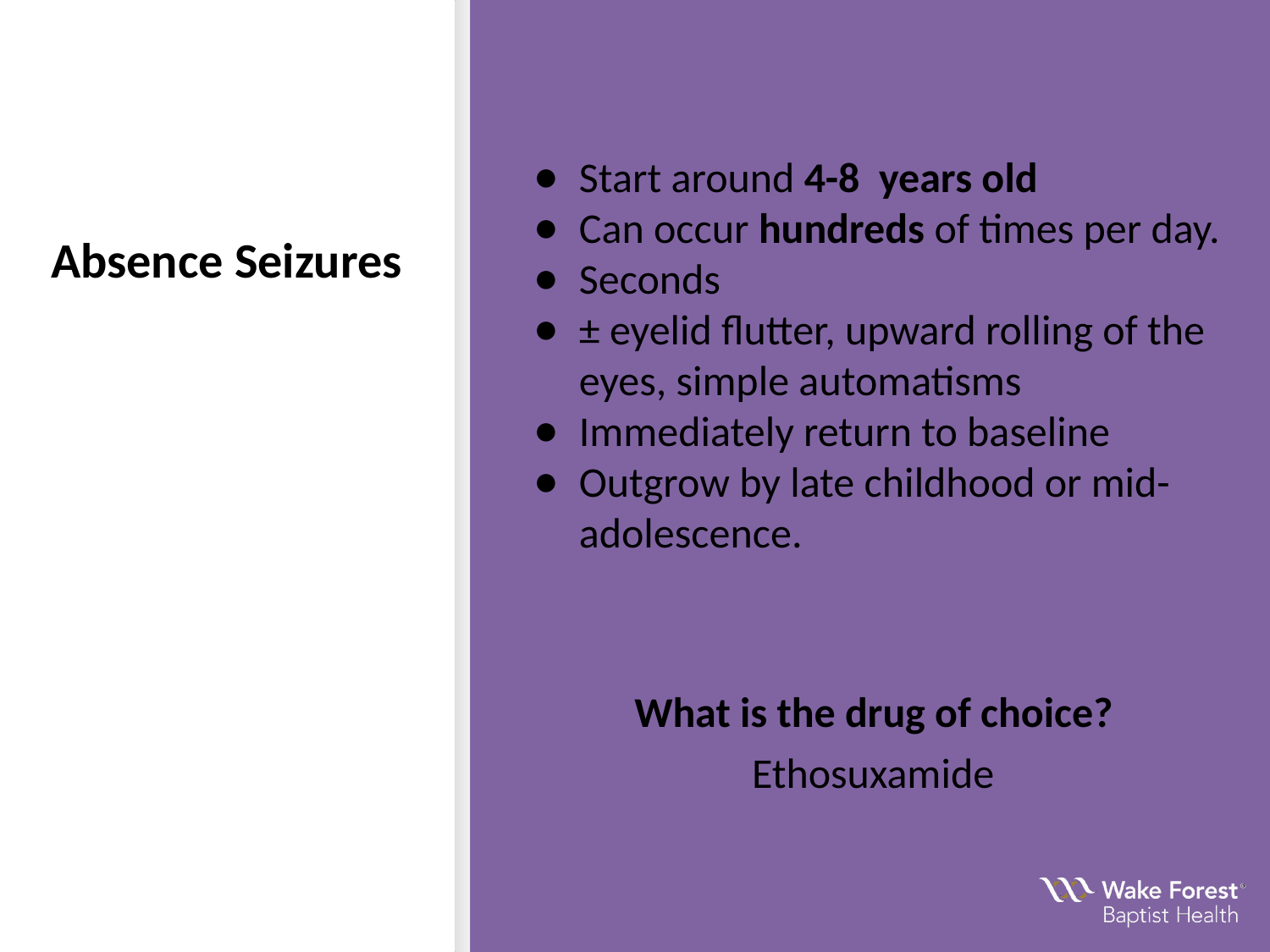

Start around 4-8  years old
Can occur hundreds of times per day.
Seconds
± eyelid flutter, upward rolling of the eyes, simple automatisms
Immediately return to baseline
Outgrow by late childhood or mid-adolescence.
# Absence Seizures
What is the drug of choice?
Ethosuxamide

## Slide 69
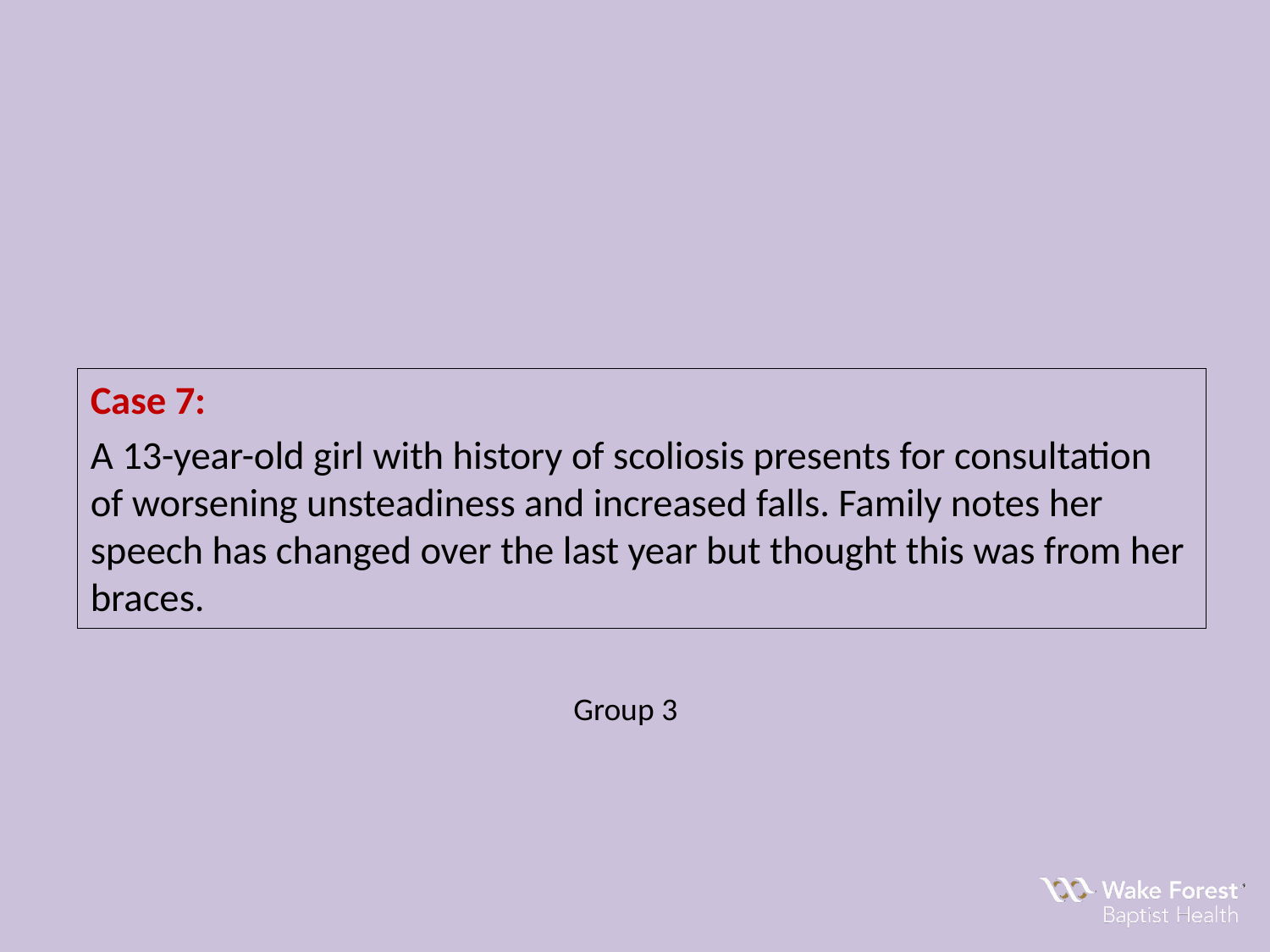

Case 7:
A 13-year-old girl with history of scoliosis presents for consultation of worsening unsteadiness and increased falls. Family notes her speech has changed over the last year but thought this was from her braces.
Group 3

## Slide 70
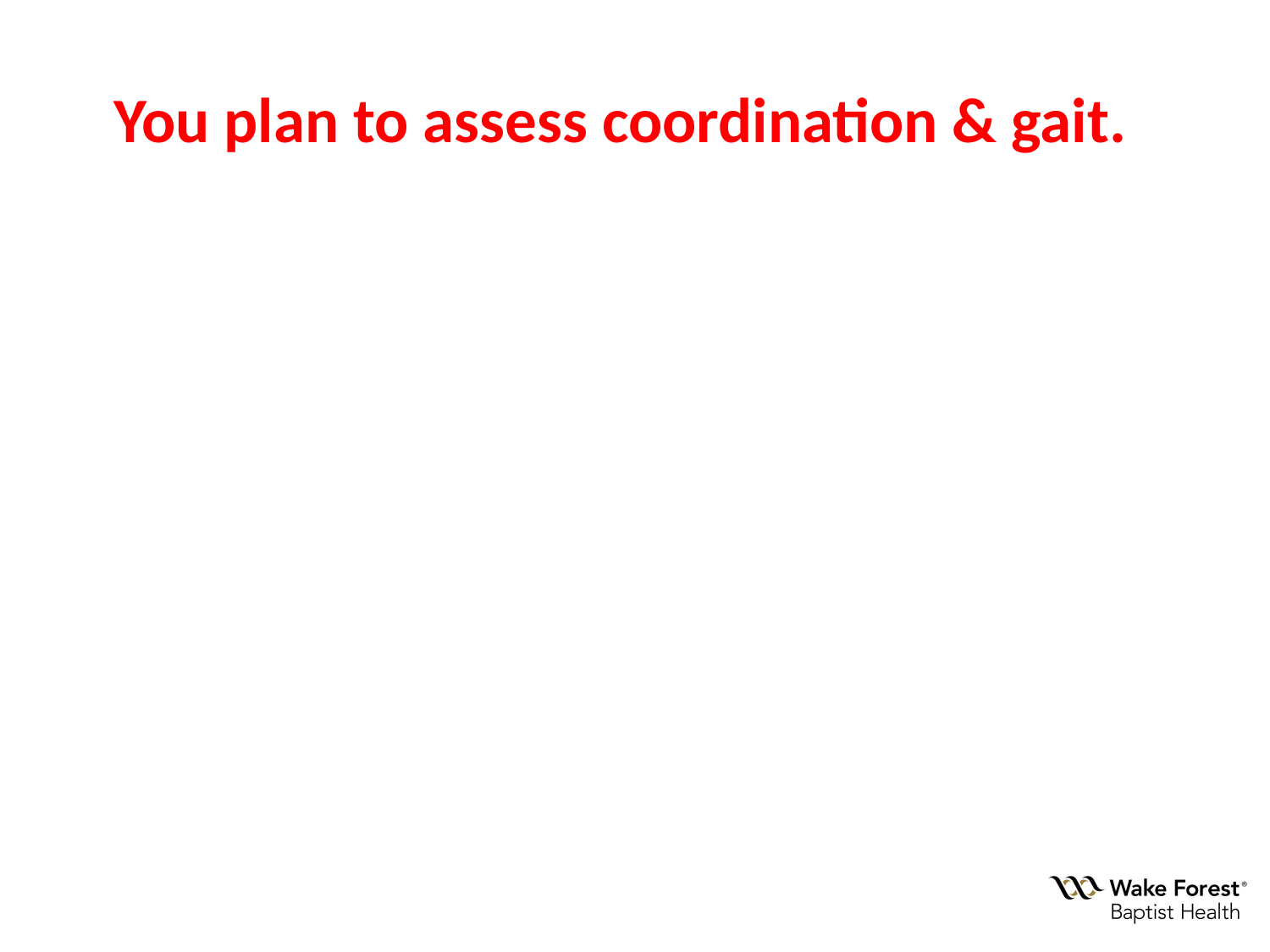

# You plan to assess coordination & gait.

## Slide 71
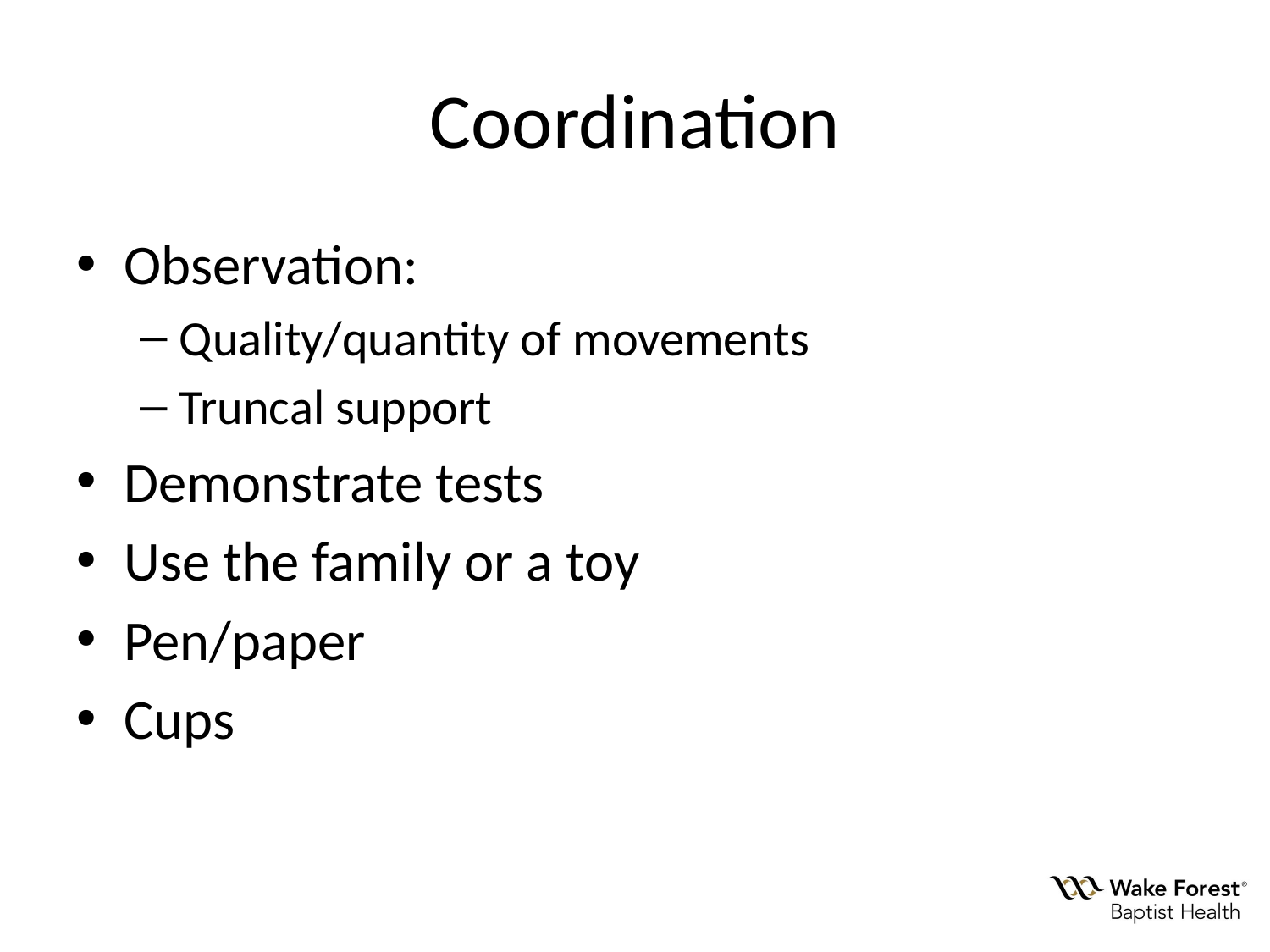

# Coordination
Observation:
Quality/quantity of movements
Truncal support
Demonstrate tests
Use the family or a toy
Pen/paper
Cups

## Slide 72
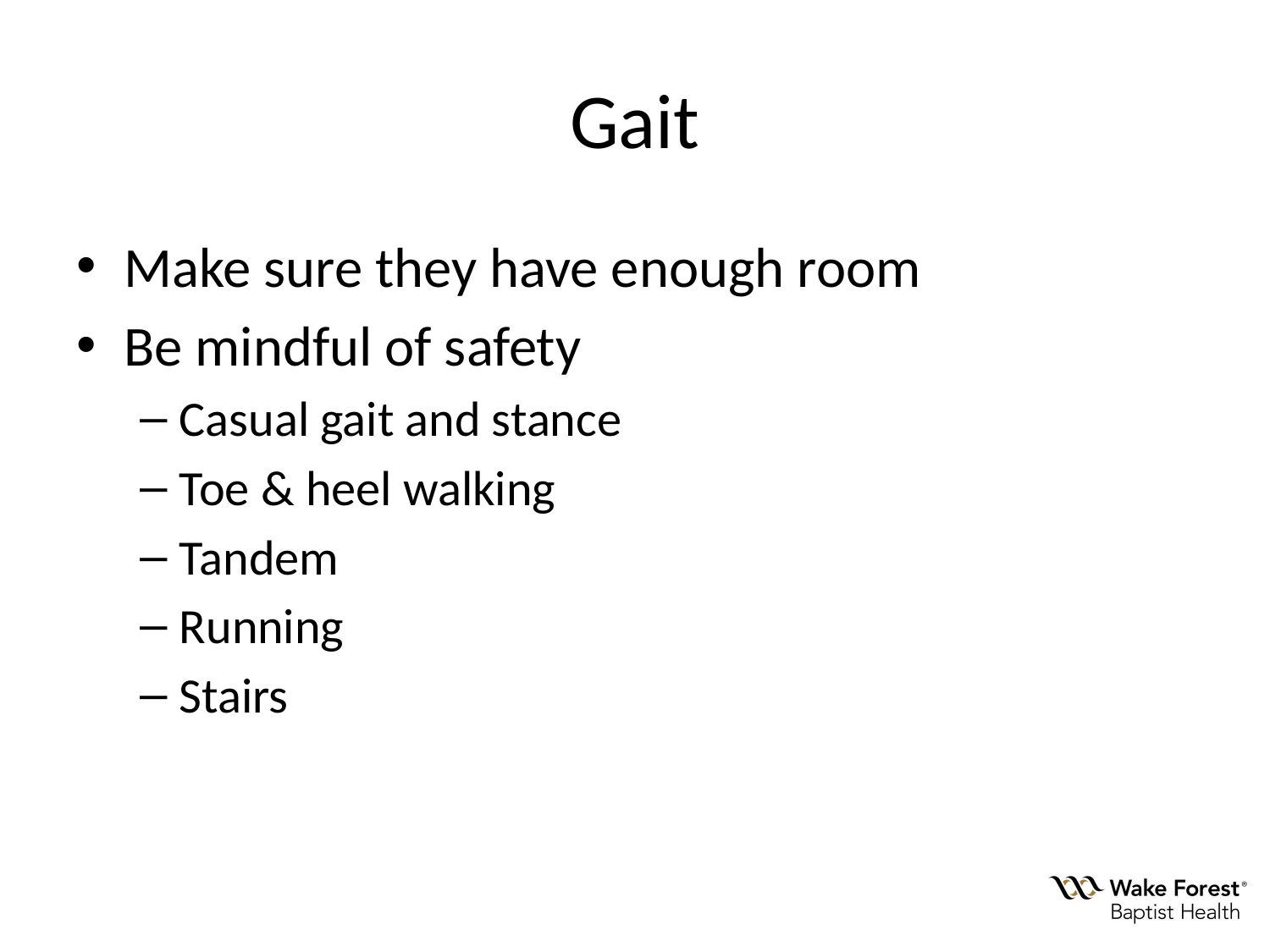

# Gait
Make sure they have enough room
Be mindful of safety
Casual gait and stance
Toe & heel walking
Tandem
Running
Stairs

## Slide 73
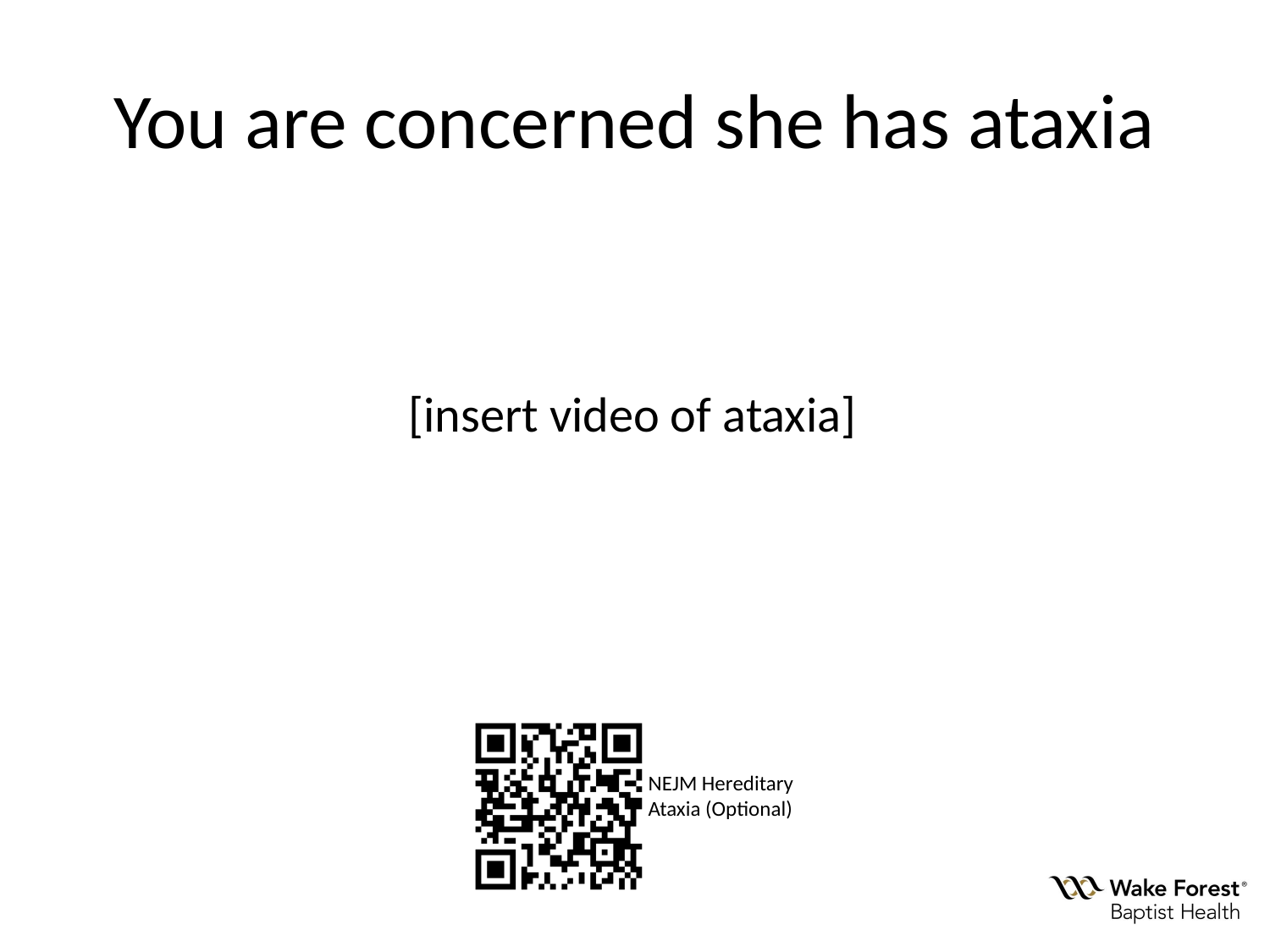

# You are concerned she has ataxia
[insert video of ataxia]
NEJM Hereditary Ataxia (Optional)

## Slide 74
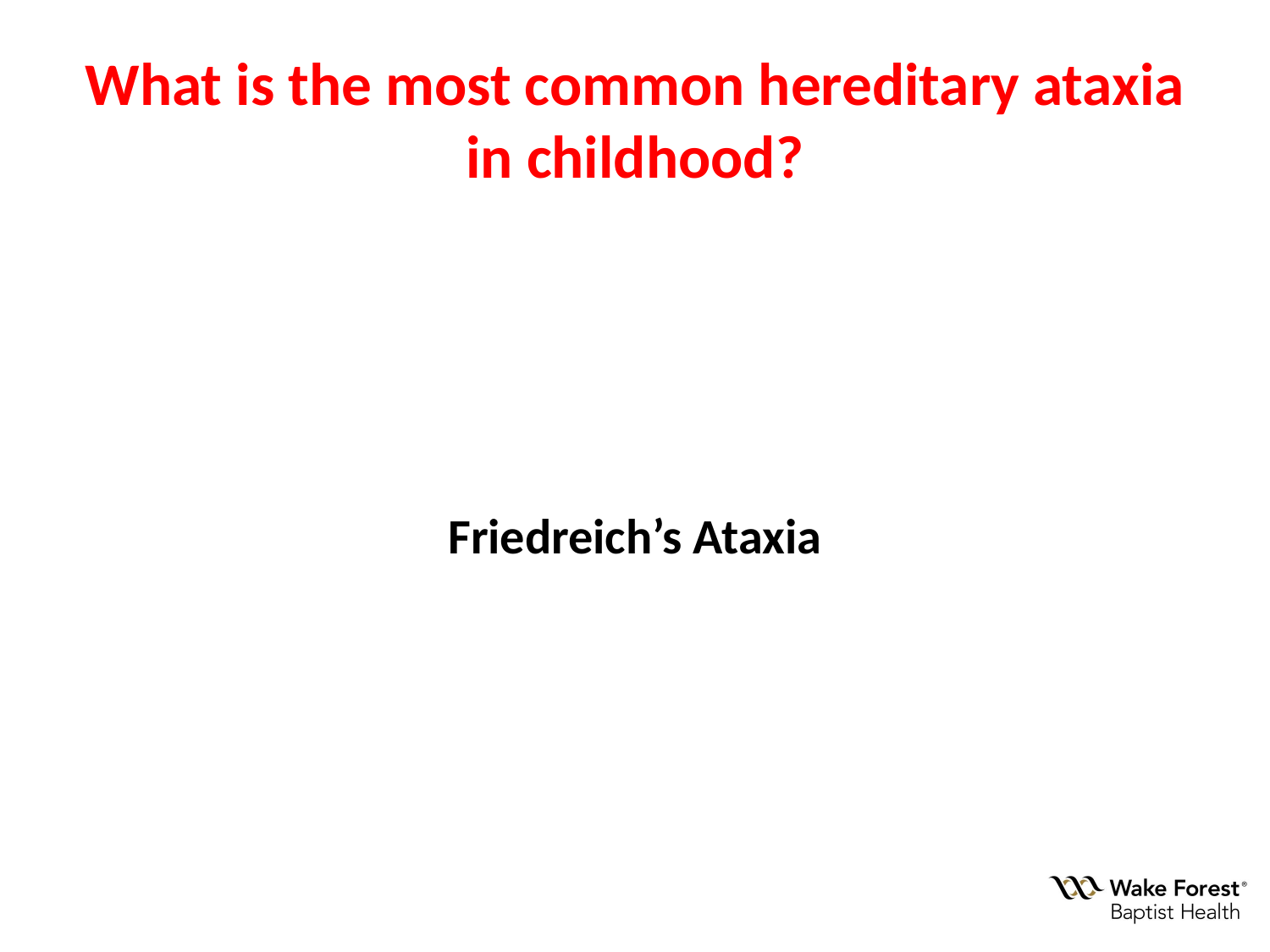

# What is the most common hereditary ataxia in childhood?
Friedreich’s Ataxia

## Slide 75
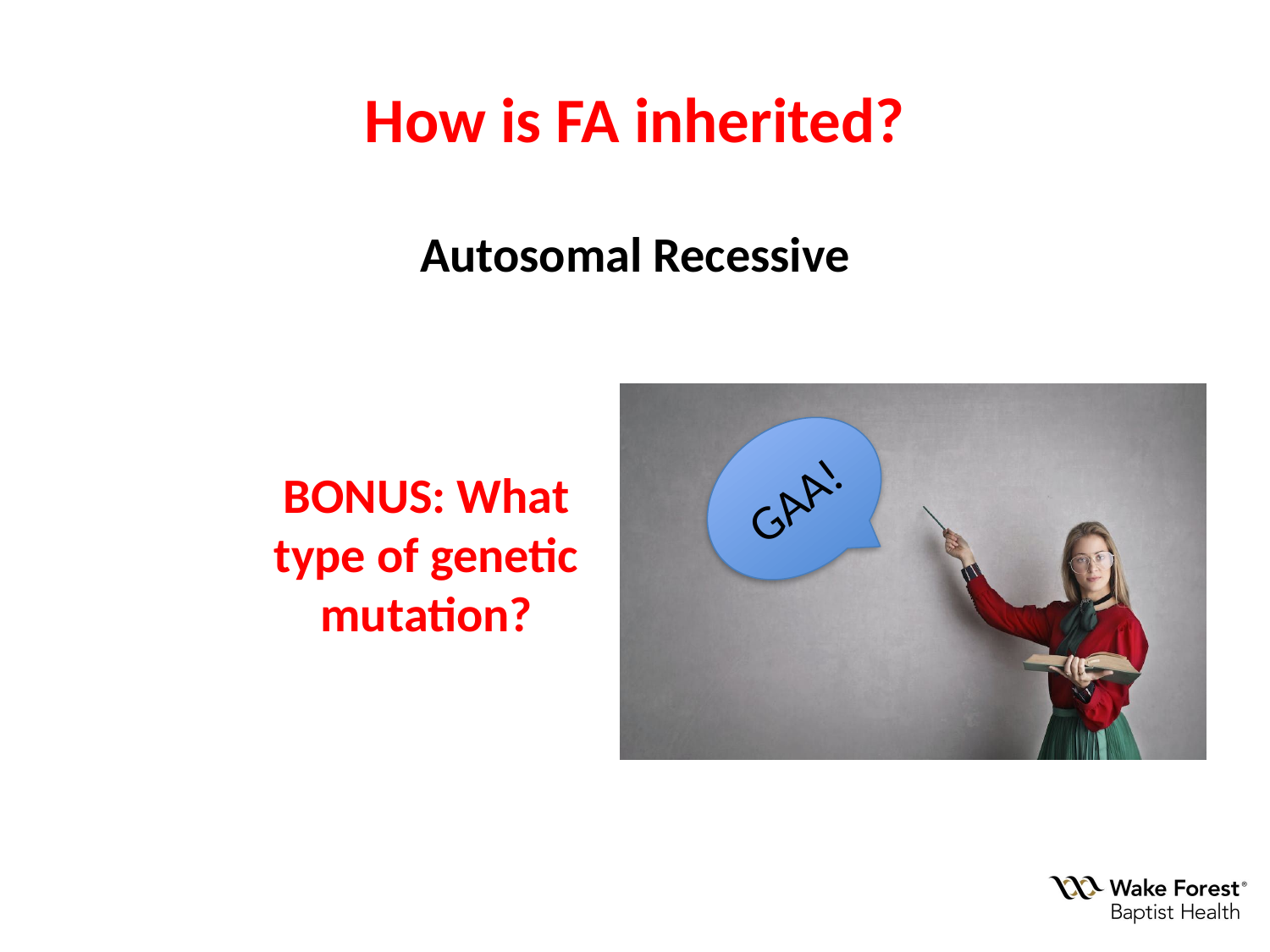

# How is FA inherited?
Autosomal Recessive
BONUS: What type of genetic mutation?
GAA!

## Slide 76
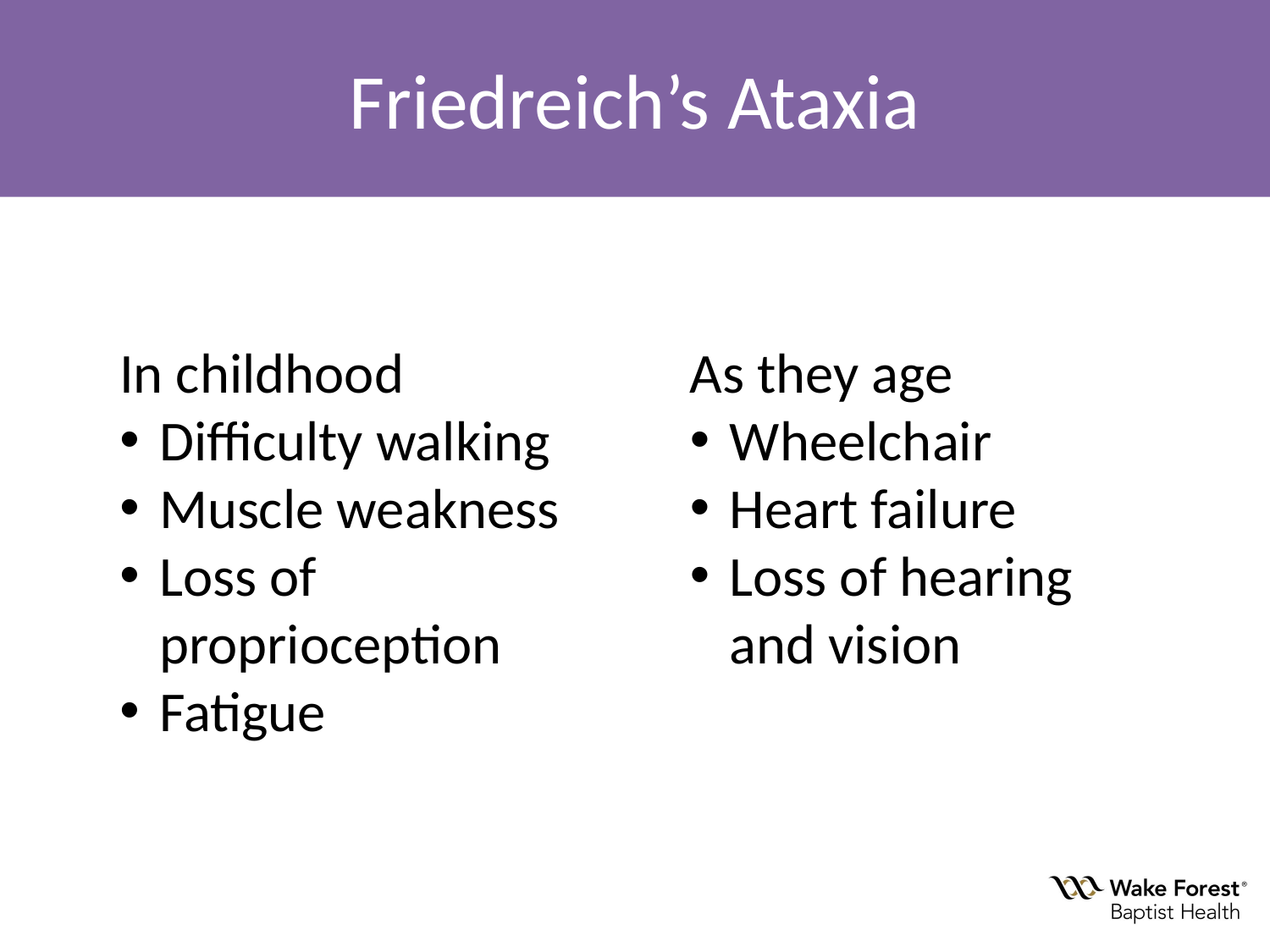

# Friedreich’s Ataxia
In childhood
Difficulty walking
Muscle weakness
Loss of proprioception
Fatigue
As they age
Wheelchair
Heart failure
Loss of hearing and vision

## Slide 77
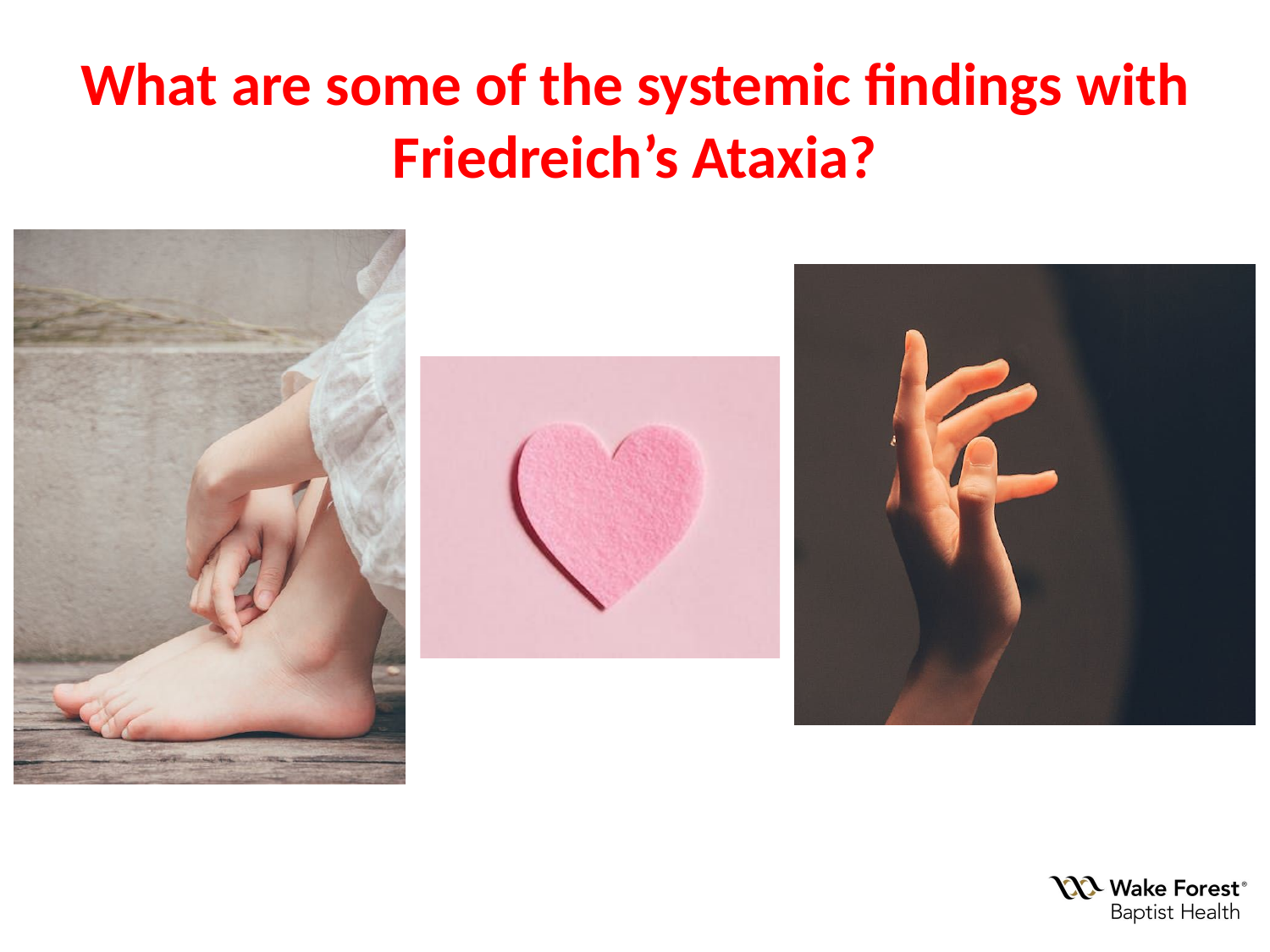

# What are some of the systemic findings with Friedreich’s Ataxia?

## Slide 78
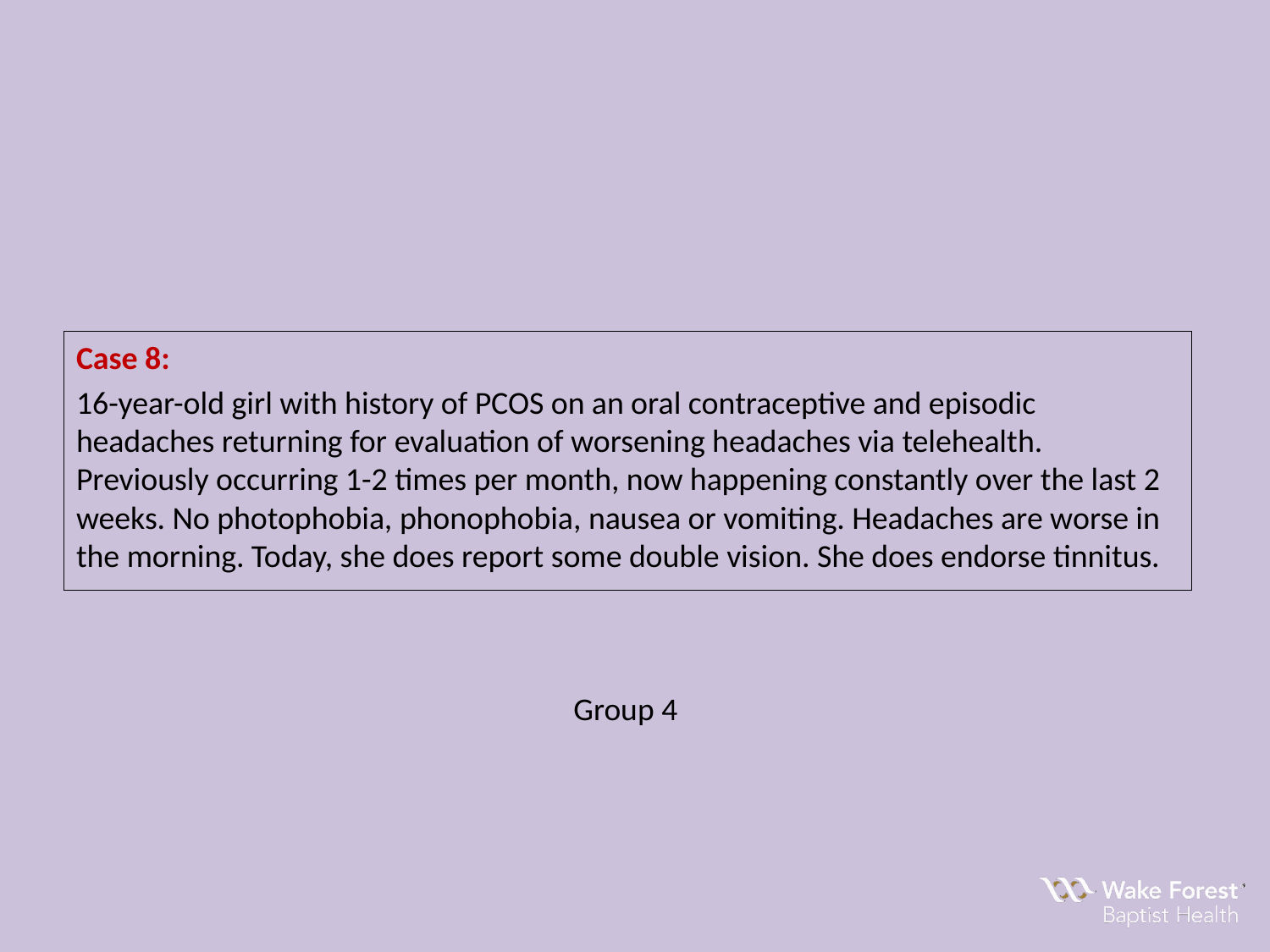

Case 8:
16-year-old girl with history of PCOS on an oral contraceptive and episodic headaches returning for evaluation of worsening headaches via telehealth. Previously occurring 1-2 times per month, now happening constantly over the last 2 weeks. No photophobia, phonophobia, nausea or vomiting. Headaches are worse in the morning. Today, she does report some double vision. She does endorse tinnitus.
Group 4

## Slide 79
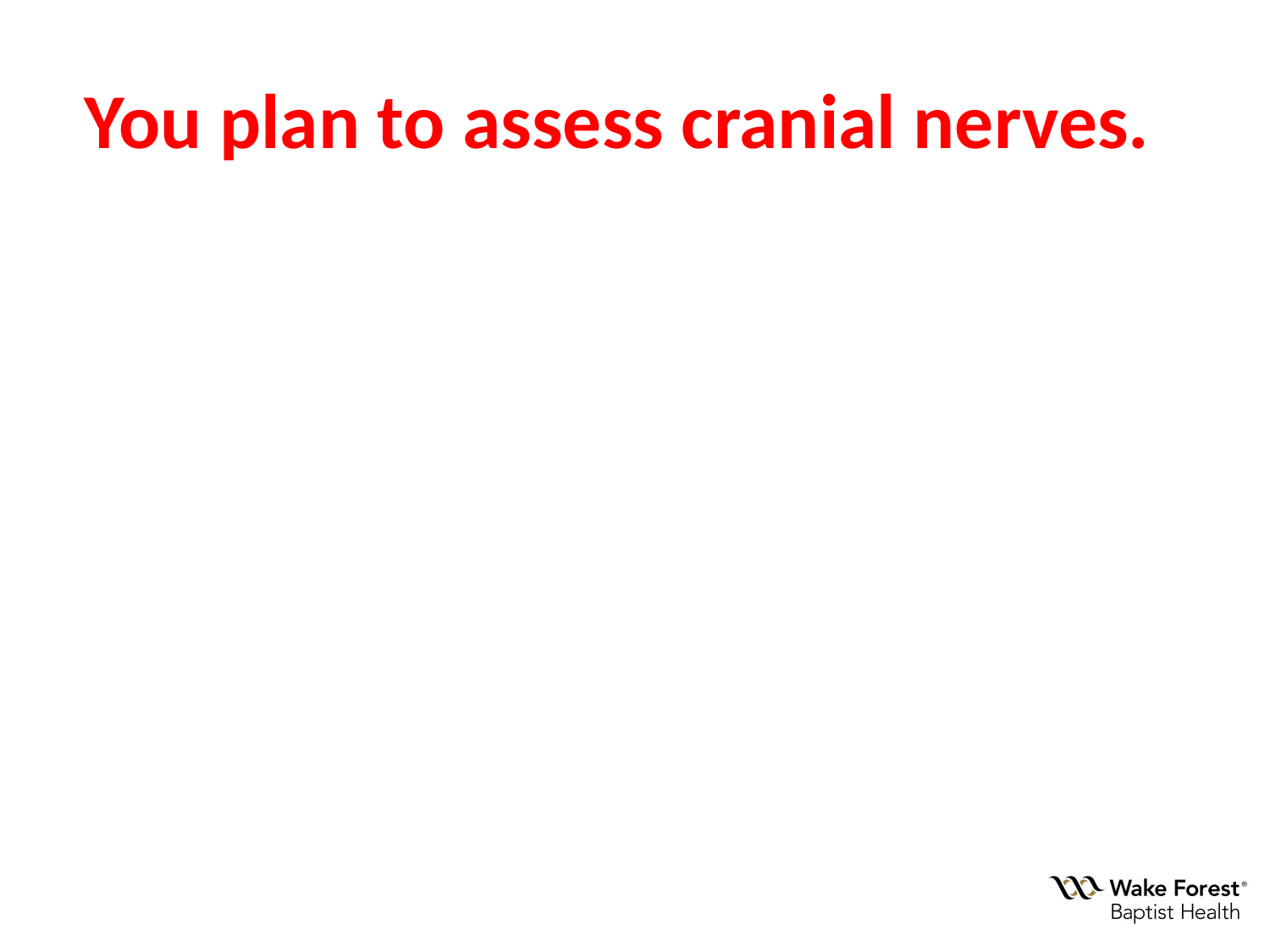

# You plan to assess cranial nerves.

## Slide 80
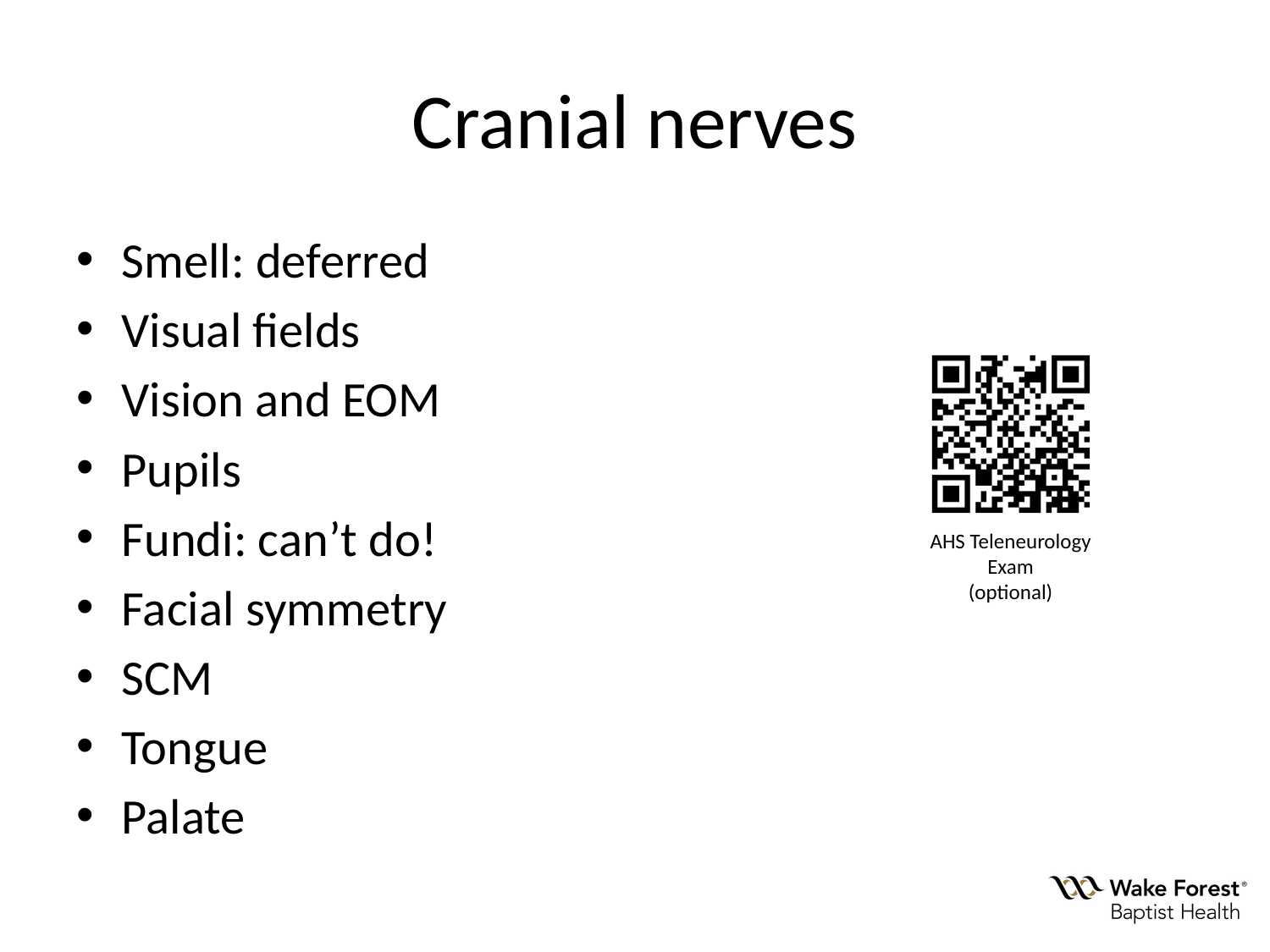

# Cranial nerves
Smell: deferred
Visual fields
Vision and EOM
Pupils
Fundi: can’t do!
Facial symmetry
SCM
Tongue
Palate
AHS Teleneurology Exam
(optional)

## Slide 81
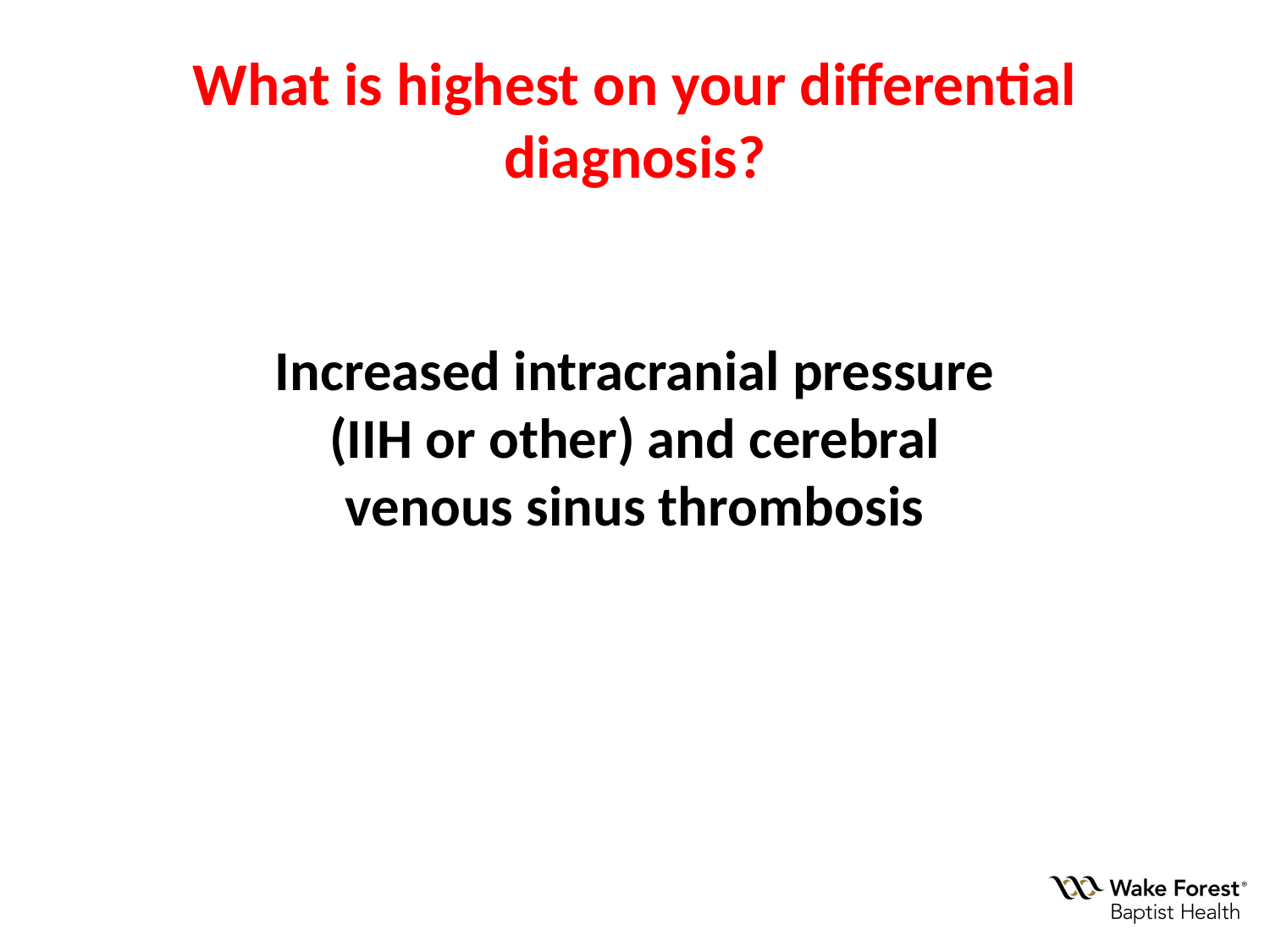

# What is highest on your differential diagnosis?
Increased intracranial pressure (IIH or other) and cerebral venous sinus thrombosis

## Slide 82
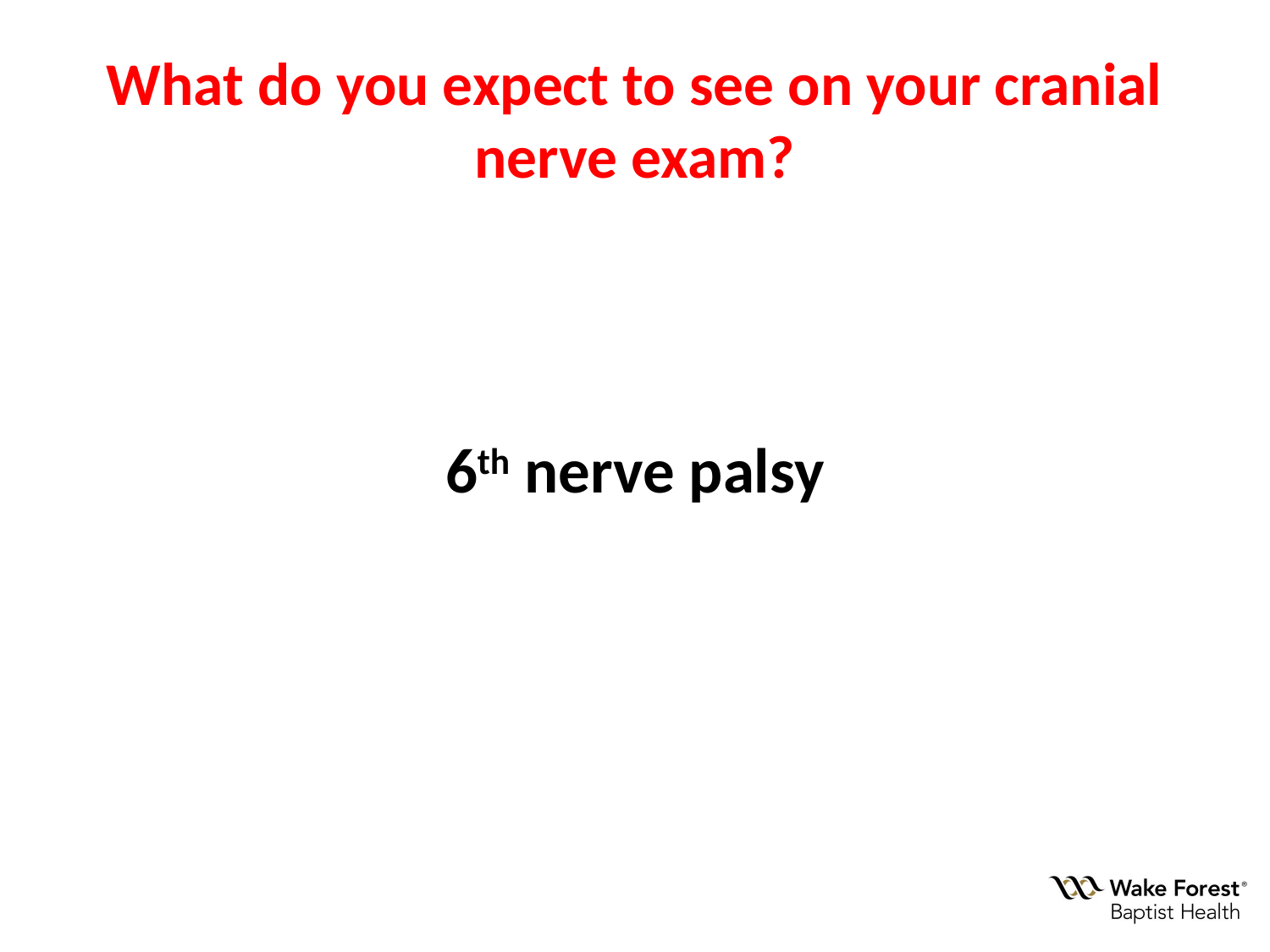

# What do you expect to see on your cranial nerve exam?
6th nerve palsy

## Slide 83
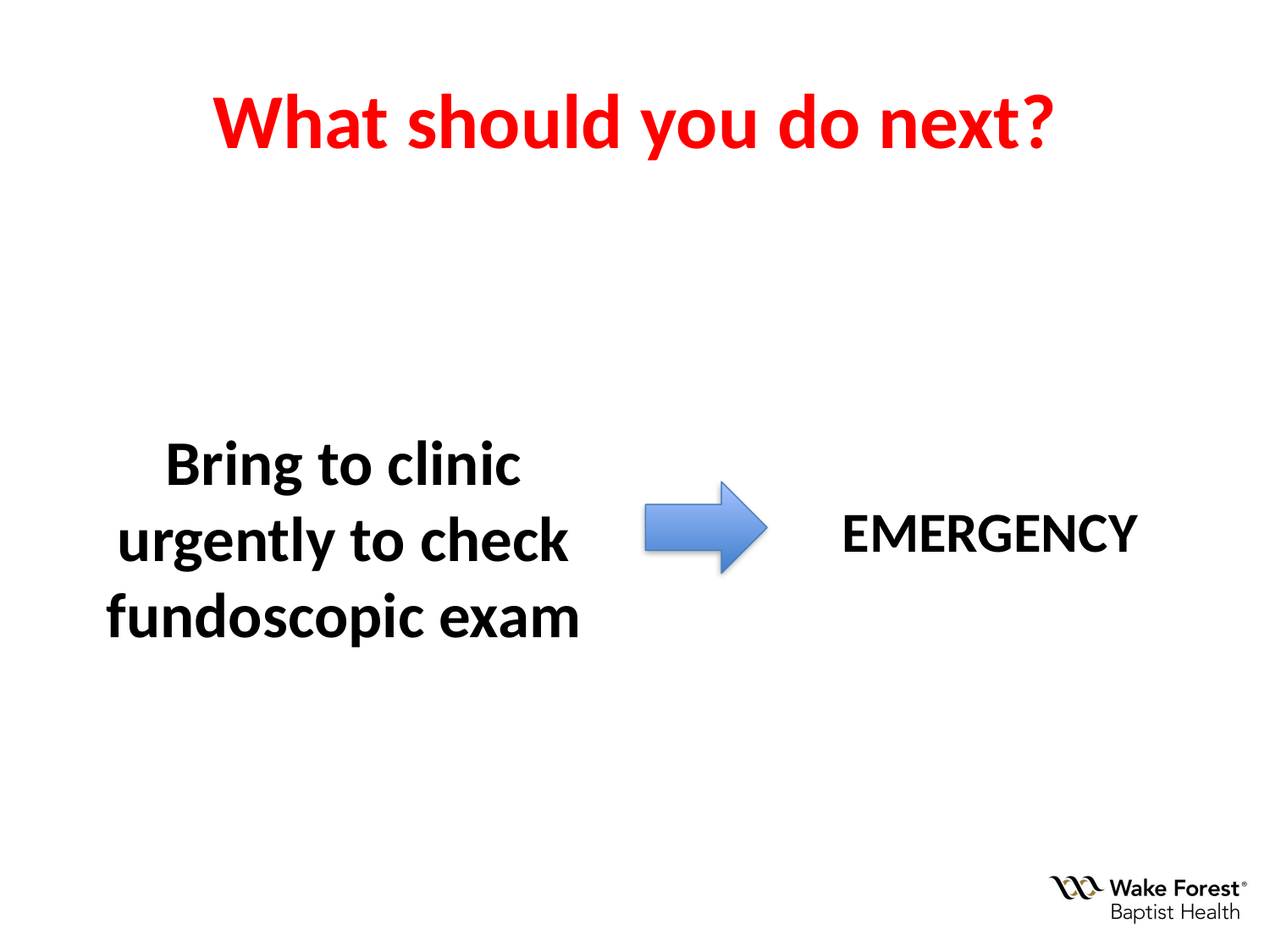

# What should you do next?
Bring to clinic urgently to check fundoscopic exam
EMERGENCY

## Slide 84
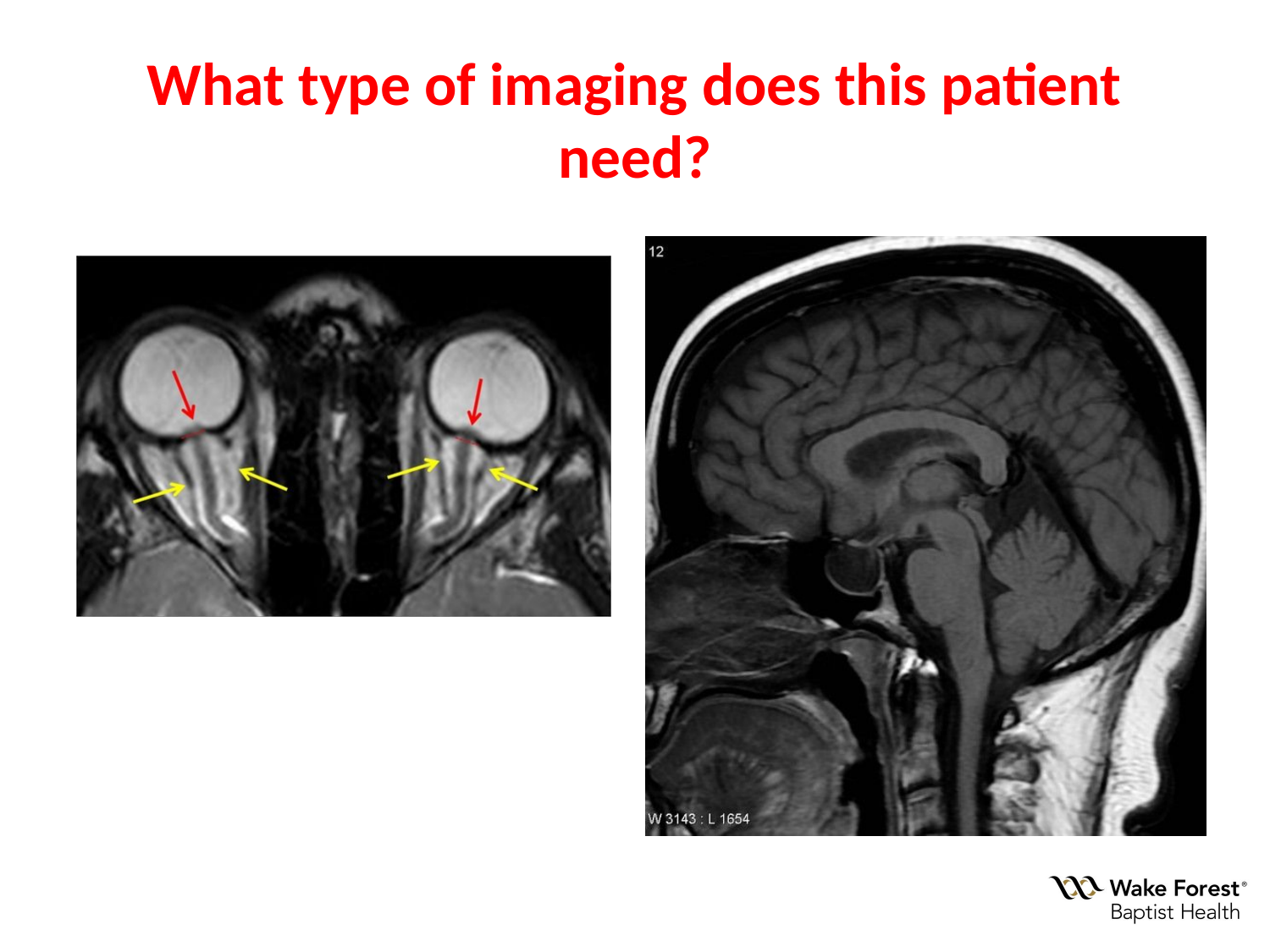

# What type of imaging does this patient need?

## Slide 85
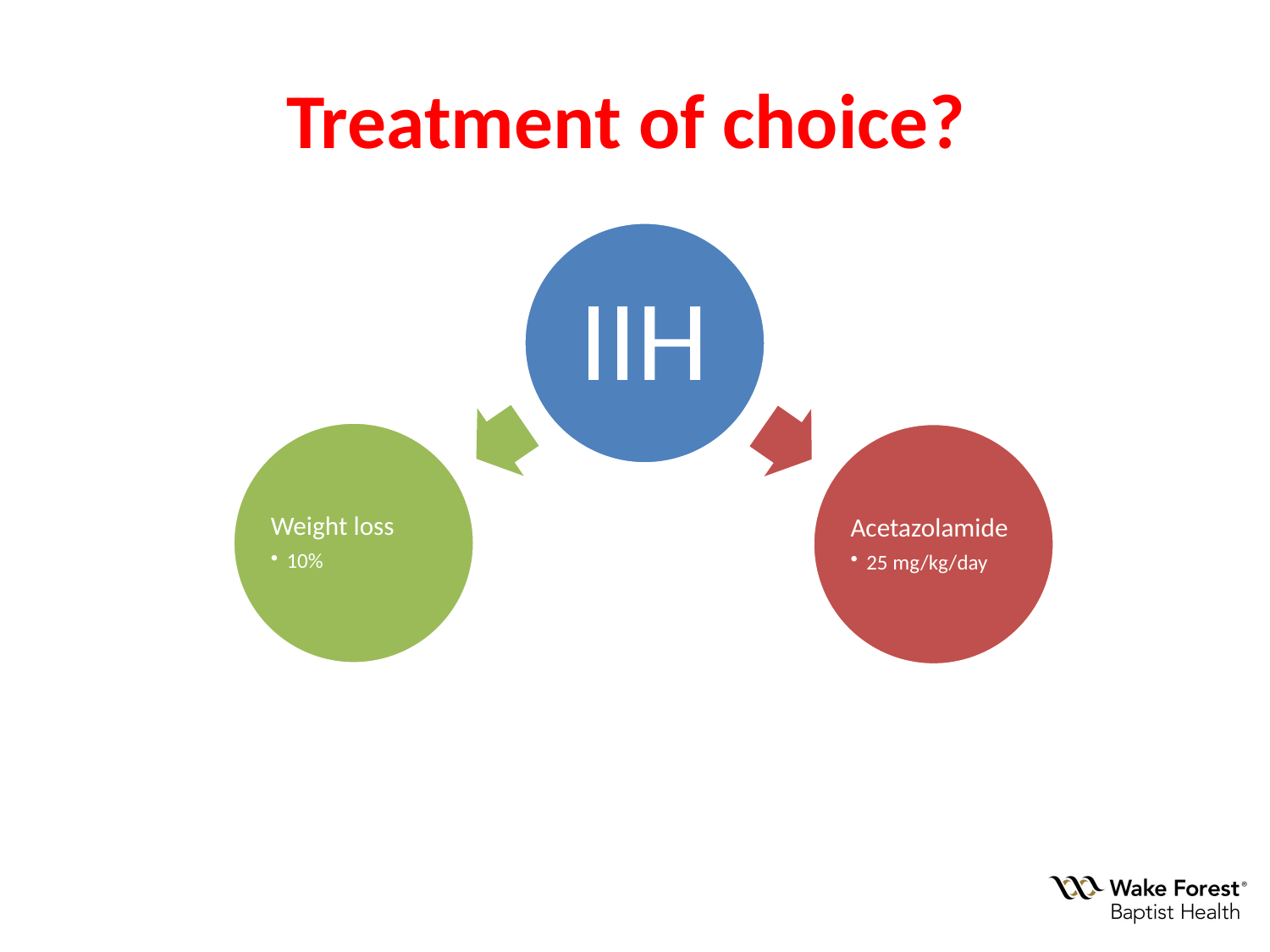

# Treatment of choice?

## Slide 86
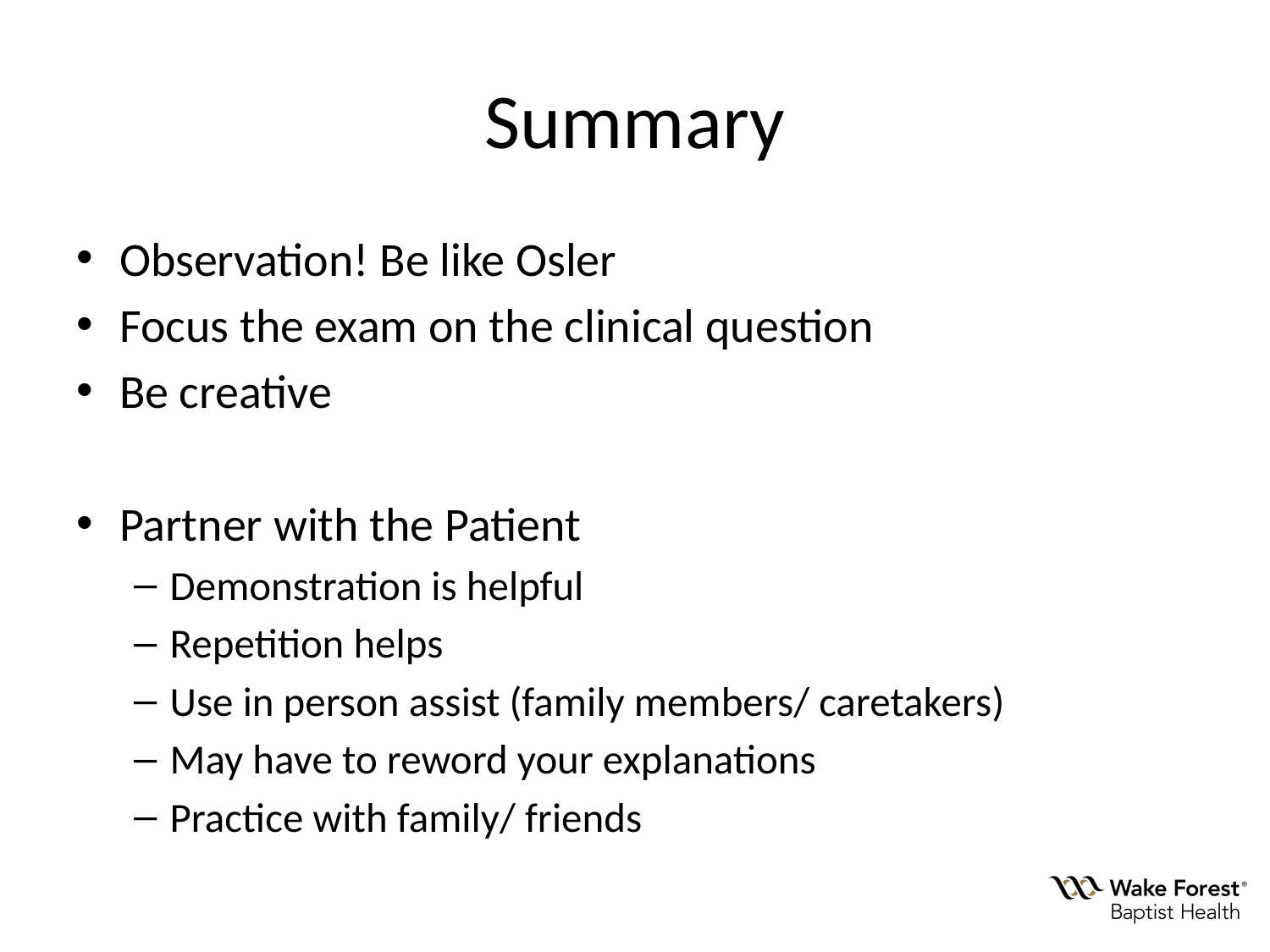

# Summary
Observation! Be like Osler
Focus the exam on the clinical question
Be creative
Partner with the Patient
Demonstration is helpful
Repetition helps
Use in person assist (family members/ caretakers)
May have to reword your explanations
Practice with family/ friends

## Slide 87
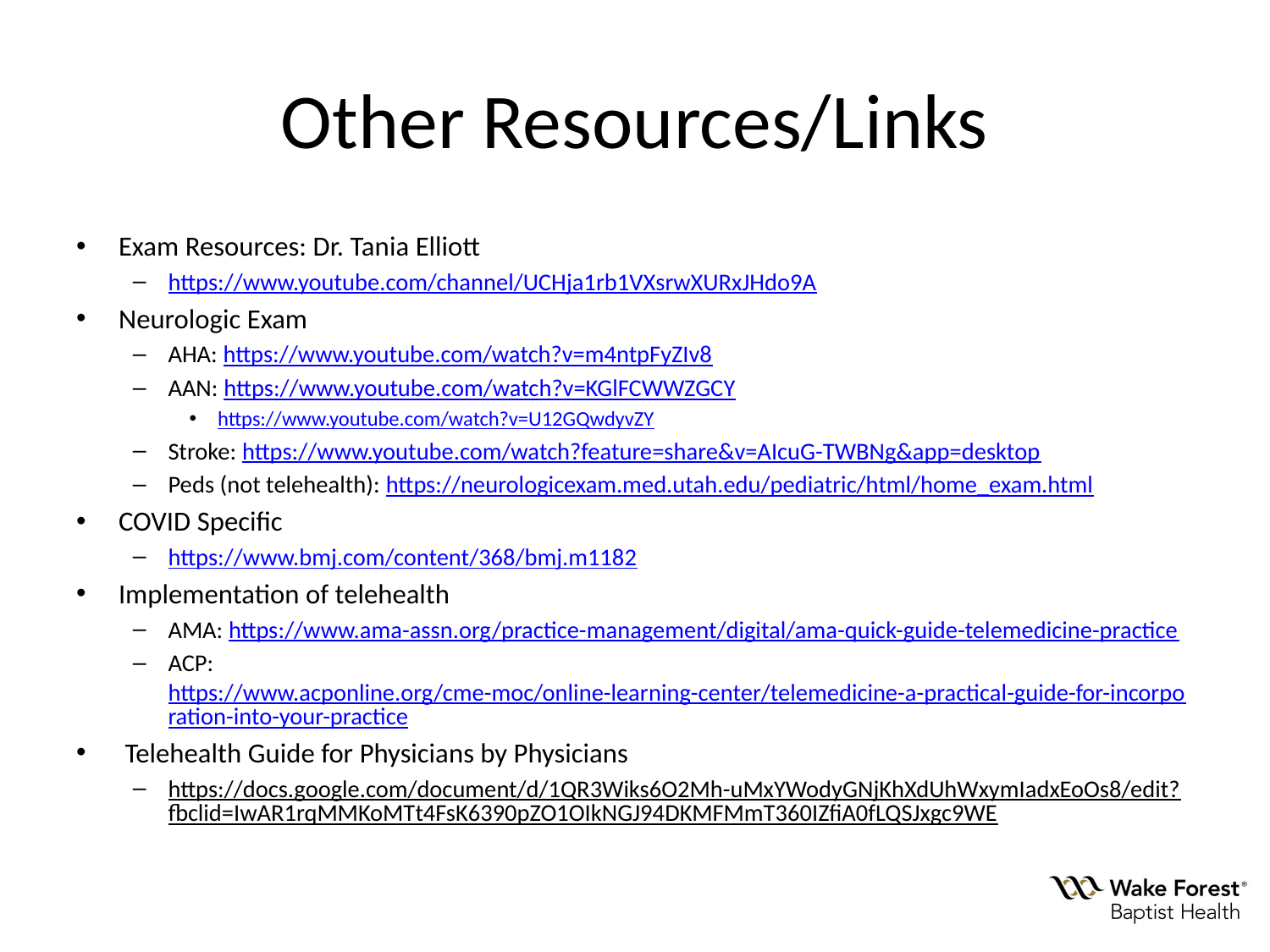

# Other Resources/Links
Exam Resources: Dr. Tania Elliott
https://www.youtube.com/channel/UCHja1rb1VXsrwXURxJHdo9A
Neurologic Exam
AHA: https://www.youtube.com/watch?v=m4ntpFyZIv8
AAN: https://www.youtube.com/watch?v=KGlFCWWZGCY
https://www.youtube.com/watch?v=U12GQwdyvZY
Stroke: https://www.youtube.com/watch?feature=share&v=AIcuG-TWBNg&app=desktop
Peds (not telehealth): https://neurologicexam.med.utah.edu/pediatric/html/home_exam.html
COVID Specific
https://www.bmj.com/content/368/bmj.m1182
Implementation of telehealth
AMA: https://www.ama-assn.org/practice-management/digital/ama-quick-guide-telemedicine-practice
ACP: https://www.acponline.org/cme-moc/online-learning-center/telemedicine-a-practical-guide-for-incorporation-into-your-practice
 Telehealth Guide for Physicians by Physicians
https://docs.google.com/document/d/1QR3Wiks6O2Mh-uMxYWodyGNjKhXdUhWxymIadxEoOs8/edit?fbclid=IwAR1rqMMKoMTt4FsK6390pZO1OIkNGJ94DKMFMmT360IZfiA0fLQSJxgc9WE
